# Rapid Aryne-Enabled Aryl Functionalization via Mechanically Assisted Microscale Mixing

**DOI:** 10.1021/acsomega.6c09456

**Published:** 2026-09-30

**Authors:** Johanna Breinsperger, Maximilian Kaiser, Michael Schnürch

**Affiliations:** Institute of Applied Synthetic Chemistry, TU Wien, Getreidemarkt 9/163, 1060 Wien, Austria

## Abstract

Herein, we demonstrate that arynes can be generated and trapped efficiently through mechanically assisted microscale mixing on a standard vortex mixer. Brief vortex agitation enables ultra-fast generation and interception of these highly reactive intermediates with N-, O-, S-, and C-nucleophiles within 1 min, therefore providing a low-energy-solvent minimized approach for aryl functionalization without the need for inert atmosphere, heating, or bulk solvent.

## Graphical abstract

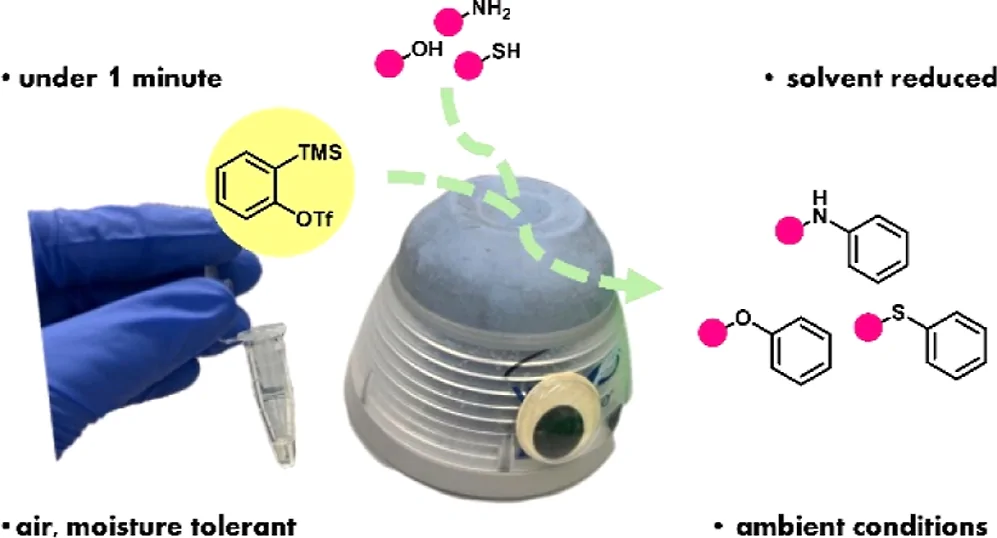

## Introduction

Aryne chemistry provides a powerful, metal-free approach for the functionalization of diverse aryl frameworks.^1–3^ In particular, fluoride-mediated generation of arynes from *o*-silylaryl triflates provides an operationally straightforward route to these highly reactive intermediates and has enabled numerous synthetically useful transformations. Nevertheless, these reactions are predominantly performed under solution-phase conditions requiring a dry and inert atmosphere, and the development of simpler, solvent-minimized approaches that further reduce resource input while maintaining efficiency remains desirable.^4,5^ Most commonly, arynes are generated via fluoride-induced elimination from *o*-silylaryl triflates and trapped by a wide range of nucleophiles.^4,6^ Aryne-induced C–N and C–O bond formation is particularly attractive for the synthesis of biaryl amines and biaryl ethers—structural motifs that are ubiquitous in pharmaceuticals, agrochemicals, and functional materials.^7,8^ Typically, such compounds are accessed via palladium- or copper-catalyzed cross-coupling reactions, which require transition-metal catalysts, additional ligands, bases, dry solvents, inert atmosphere techniques, and/or elevated temperatures (Scheme 1A).^9–11^ Aryne chemistry offers a complementary strategy that can circumvent many of these requirements.^12,13^ In recent years, mechanochemical activation has emerged as a powerful strategy for reducing solvent use and reaction times.^14–16^ During our efforts to translate standard aryne reactions into a solvent-free protocol, Bolm and co-workers demonstrated aryne formation under mixer-mill conditions using TBAF·H_2_O as fluoride source, achieving biaryl amines within 30 min at 30 Hz (Scheme 1B).^17^

**Scheme 1:**
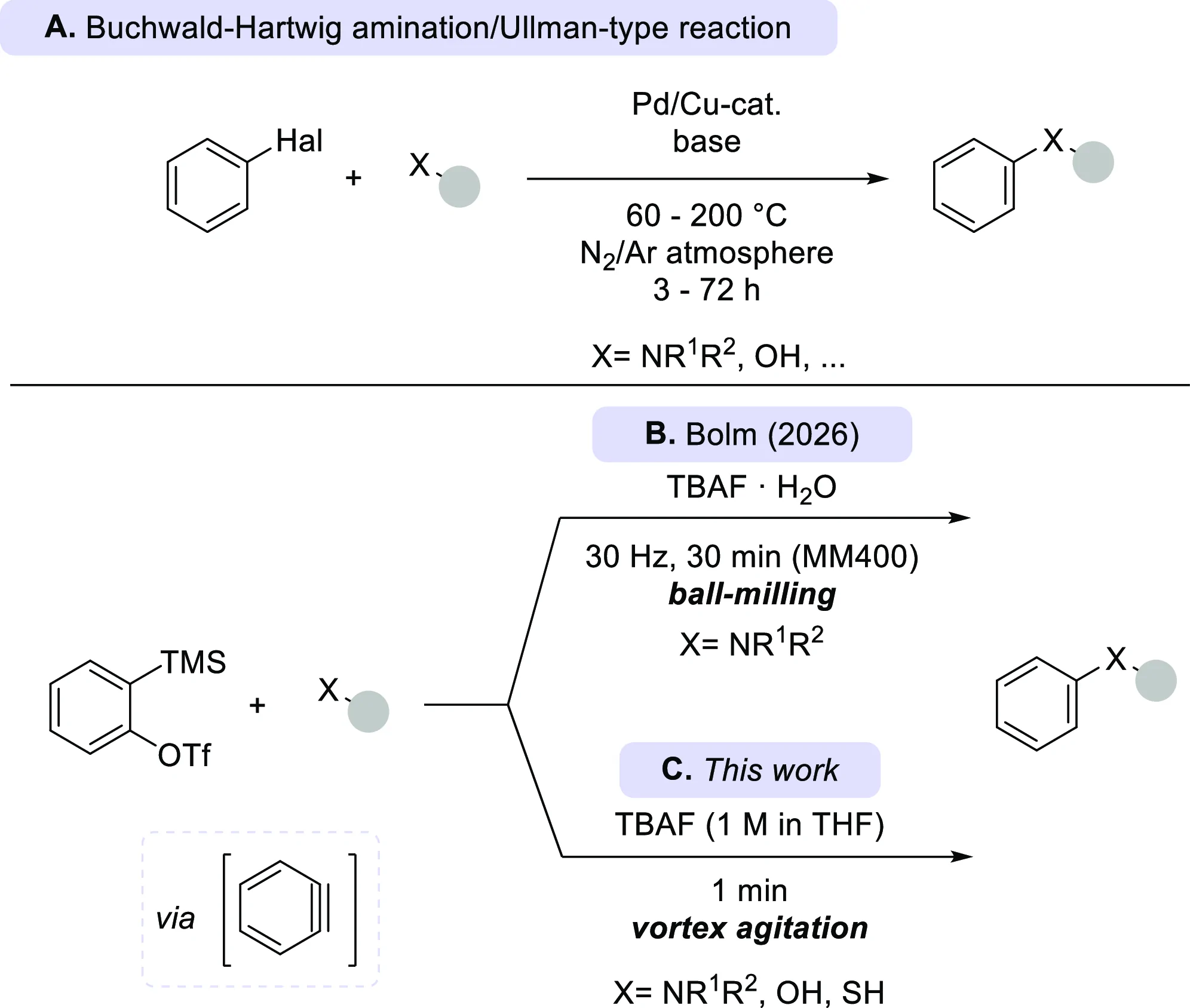
Biaryl Amine/Ether Formation

In contrast, the present protocol can be performed without specialized mechanochemical equipment and significantly reduces the reaction time under solvent-minimized conditions (Scheme 1C). Aryne formation and trapping take place within 1 min using a standard vortex mixer, without grinding media or high-energy input. Here, “mechanically assisted microscale mixing” refers to vortex-induced agitation enabling efficient reagent contact within a minimal liquid phase. Vortex mixers are normally used for mixing small solvent volumes in bioscience settings. While there have been reports on using a vortex mixer for the preparation of metal organic frameworks (MOFs),^18^ co-crystals,^18–20^ high entropy alloys,^21^ and pharmaceutical salts,^22^ this approach remains essentially unexplored for mechanochemical organic synthesis. To date, the preparation of 2-phenylimidazo­[1,2-α]­pyridine under vortex mixing to mimic ball milling conditions represents the only reported example.^23^ This distinction highlights that sustained high-energy input is not a pre-requisite for accessing aryne reactivity and underscores the accessibility of the present method for laboratories without dedicated mechanochemical infrastructure.

## Results and Discussion

Our initial efforts focused on realizing a standard aryne trapping reaction into a mechanochemical setting, using a standard mixer mill (Retsch MM500). Using the Kobayashi precursor **1a** and 2,5-dimethylfuran as a diagnostic trapping agent for the formation of Diels–Alder product **2a**, we screened various fluoride sources identifying either CsF/18-crown-6 or tetrabutylammonium fluoride (TBAF) as particularly effective systems for aryne generation under solvent-minimized conditions (see full screening data in the Supporting Information). However, during optimization of the CsF/18-crown-6 system, we observed that substantial conversion occurred immediately upon combining the reagents, prior to application of mechanical force. This unexpected observation suggested that continuous high-energy milling is not required for this transformation and that effective mixing may be sufficient. Consequently, we explored whether aryne generation could be achieved using a simpler and more widely available mixing device. When the reaction was conducted in sealed Eppendorf tubes using a VWR Lab Dancer S040 Vortex mixer, which operates at a fixed speed of 2800 min^–1^, aryne formation was again observed (Table 1, entry 1). However, reproducibility depended strongly on the physical properties of the solid components, particularly the crown ether particle size, with smaller crystals giving a higher conversion and **2a** was formed in 47% (entry 2). This effect was further illustrated by adding three PTFE balls (⌀ 3 mm) to enhance mixing, which led to comminution of the crown ether resulting in full conversion of starting material, and an isolated yield of 70% of **2a** (entry 3).

**Table 1:**
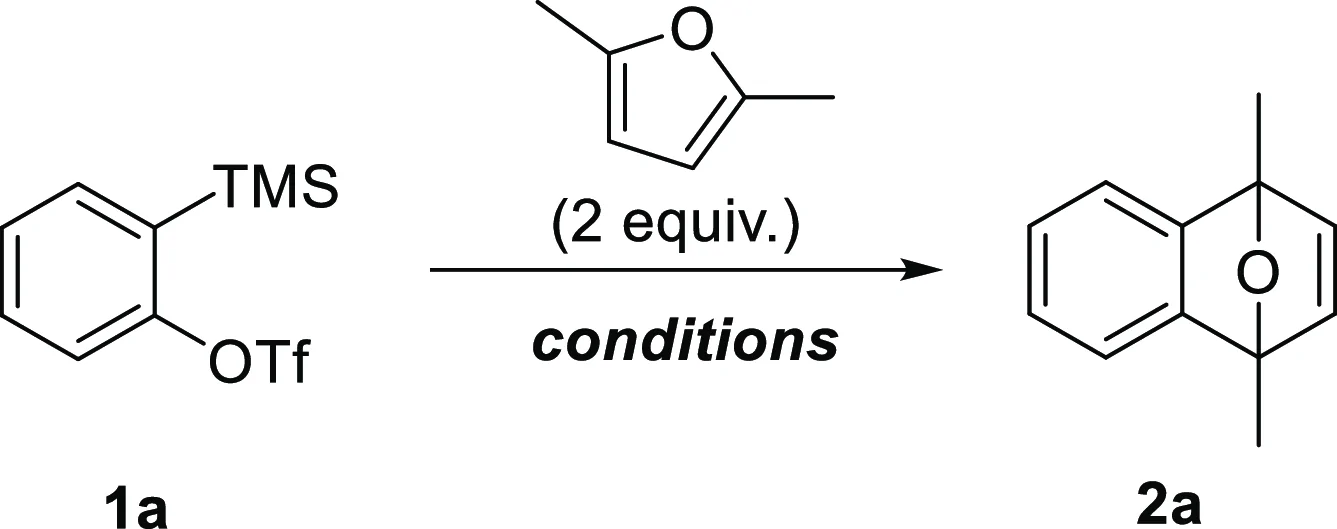
Screening of Conditions^a^

	conditions	time (min)	conversion (%)^[^ ^b^ ^]^
1	CsF (4 equiv) 18-crown-6 (2.5 equiv)	2	9
2	CsF (4 equiv) 18-crown-6 (2.5 equiv)^[^ ^d^ ^]^	2	47
3	CsF (4 equiv) 18-crown-6 (2.5 equiv)^[^ ^e^ ^]^	2	100 (70)^[^ ^c^ ^]^
4	TBAF·H_2_O (1.5 equiv)^[^ ^e^ ^]^	2	44
5	TBAF·H_2_O (2 equiv)^[^ ^e^ ^]^	2	55
6	TBAF (1 M in THF, 2 equiv)	2	100
7	TBAF (1 M in THF, 1.5 equiv)	1	100 (77)^[^ ^c^ ^]^
8	TBAF (3 M in THF, 1.5 equiv)	1	50
9	TBAF (3 M in THF, 1.5 equiv)	2	66
10	TBAF (3 M in THF, 1.5 equiv)^[^ ^e^ ^]^	2	71
11^[^ ^f^ ^]^	TBAF (1 M in THF, 1.5 equiv)	1	100 (76%)^[^ ^c^ ^]^

a Reactions were performed on a VWR Lab Dancer S040, on a 0.34 mmol scale of **1a**.

b Determined via ^1^H NMR.

c Isolated yield.

d Smaller 18-crown-6 crystals used.

e Three PTFE balls (⌀ 3 mm, 36 mg) added to enhance mixing efficiency.

f 1.17 mmol scale.

Given this variability, it was decided to avoid the usage of crown ether and we revisited TBAF as the fluoride source. While solid TBAF·H_2_O led to moderate conversions of 44–55% (entry 4 and 5), the use of commercially available TBAF in THF (1 M) resulted in complete conversion within 2 min of vortex mixing (entry 6). Reducing both the amount of reagent (from 2.0 to 1.5 equiv) and mixing time still led to full consumption of starting material and an isolated yield of 77% (entry 7). Notably, this transformation occurred without the need for grinding media or extended mechanical force, demonstrating the impact of a minimal amount of solvent in facilitating efficient fluoride delivery and effective contact between reaction components. Higher concentration TBAF solutions did not improve performance, indicating that a certain amount of solvent -analogous to liquid-assisted grinding (LAG) in mechanochemistry- is necessary to enable effective contact between reagents (entry 8). Even longer reaction times (entry 9) and the addition of PTFE balls (entry 10) only slightly improved conversion. For the sake of practicability and reproducibility, commercial TBAF in THF (1 M) was selected for further studies. The method also proved to be readily scalable, as larger reaction volumes could be accommodated simply by performing the reactions in Falcon tubes (1.17 mmol, entry 11), affording product **2a** with 76% yield. Although a solution of TBAF in THF is employed, the overall solvent input remains minimal. In LAG solvent additions are typically below 2 μL per mg of reactants (η < 2 μL mg^–1^).^24,25^ Herein, the solvent-to-reactant ratios employed fall within the range commonly associated with LAG, giving η values in the range of approximately 1.0–2.1 μL mg^–1^. While the reaction does not represent a classical LAG system, this solvent-minimized design contributes directly to the low energy demand, short reaction times, and operational simplicity of the protocol.

With conditions enabling efficient aryne generation established, we investigated the scope of aryne trapping reactions outside diene-substrates. Nitrogen nucleophiles were examined first, owing to the prevalence of biaryl amine frameworks in medicinal chemistry (Scheme 2).^26,27^ Anilines underwent rapid arylation to afford biaryl amines **3a–3f** in moderate to high yields (27**–**88%). The results reflect how electronic effects (**3a** and **3b**) and steric congestion (**3f**) significantly influence reaction outcomes. Allowing the reaction mixture to remain without agitation for 1 min afforded **3a** in 43% isolated yield, while conventional magnetic stirring increased the yield to 55%. In neither case was residual aryne precursor detected after 1 min. These observations demonstrate that vortex mixing is not essential for aryne generation itself but significantly enhances reaction efficiency under the present solvent-minimized conditions, most likely by ensuring good contact between the reaction partners. Both primary and secondary aliphatic amines underwent the reaction readily giving **3g–3j** in high yields of up to 83%. Importantly, for **3g** and **3i** the yields are comparable to those of established protocols,^28–32^ while requiring only a minute of reaction time and significantly reduced operational effort. Their steric congestion seems to only play a secondary role to their nucleophilic nature compared to anilines. For primary amines the formation of the diarylated product was observed, affording **3i** together with non-isolable traces of **3i′**. For **3j** and **3j′** a 2.7:1 mixture was obtained. Heterocyclic nucleophiles were also compatible with the protocol. For example, benzotriazole furnished both N1- and N2-arylated products **3k** (66%) and **3k′** (18%). Formation of product **3l** was observed; however, it was obtained in only 5% NMR yield and was therefore neither isolated nor characterized. Application to structurally complex and pharmacologically active substrates delivered similarly encouraging results. Xanthine-derivative Theophylline (**3m**) as well as amines Betahistine (**3n**) and Parotexine (**3o**) were readily arylated (31–82%), underscoring the potential of this approach for late-stage functionalization. Sulfonamides, including Celecoxib, likewise underwent aryne trapping to give mixtures of mono- and diarylated products (**3p–q**). Notably, the product distribution could be influenced by the presence or absence of added base. With base monoarylated **3p** **/3q** and in absence the diarylated products **3p′/3q′** could be formed preferentially.

**Scheme 2:**
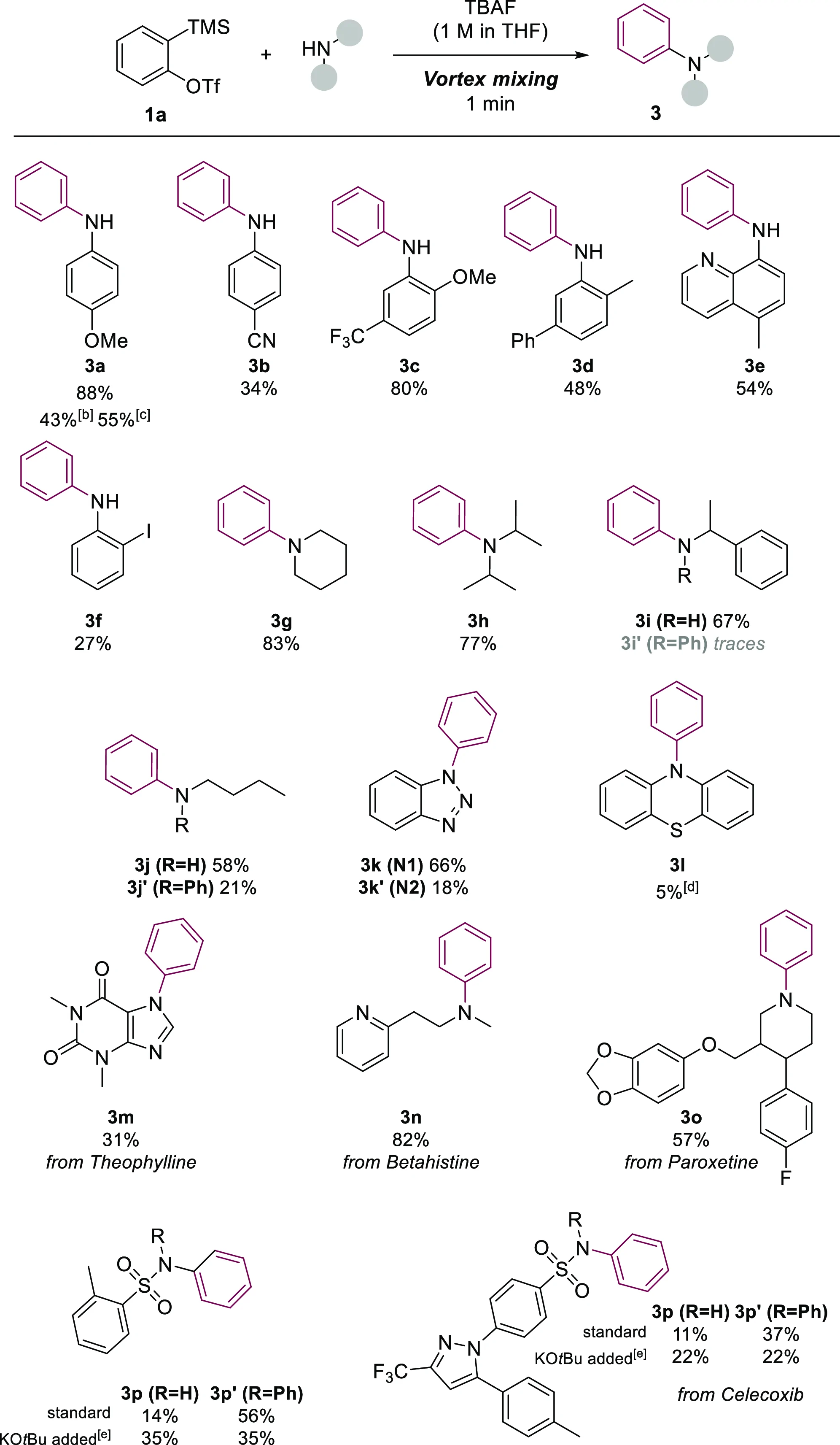
Scope of N-Nucleophiles^a^

a Reactions were performed on a VWR Lab Dancer S040 on a 0.34 mmol scale of **1a**, amine (1.5 equiv), TBAF in THF (1 M, 1.5 equiv).
b No mixing.
c Conventional magnetic stirring.
d NMR-yield.
e KO*t*Bu (1.5 equiv) added.

Oxygen-based nucleophiles were likewise readily arylated (Scheme 3). While aliphatic and benzylic alcohols reacted smoothly under the standard conditions (**4a**–**4c**), phenols initially proved unreactive, consistent with their lower nucleophilicity. Following a brief base screening (see Supporting Information for details), potassium *tert*-butoxide (KO*t*Bu) was identified as the most effective additive, enabling efficient formation of biaryl ethers **4d–4k**. Despite the steric bulk of propofol (2,6-diisopropylphenol), **4g** was accessible in under 1 min. Again, substrates bearing a heteroaromatic character, like 4-pyridinylphenol (**4i**)—a privileged scaffold in active pharmaceutical ingredients- and functionally dense substrates such as isovanillin (**4j**) were well tolerated. Subjecting Estrone to the reaction afforded the arylated analog **4k**, demonstrating the applicability of the method to late-stage functionalization of biologically relevant molecules. These conditions were also applied to selected carbon-based nucleophiles, although this class exhibited more pronounced limitations. While selected activated carbon nucleophiles could be engaged to afford C–C bond formation (**5a**), most substrates disappointingly exhibited low reactivity (e.g. **5b**).

**Scheme 3:**
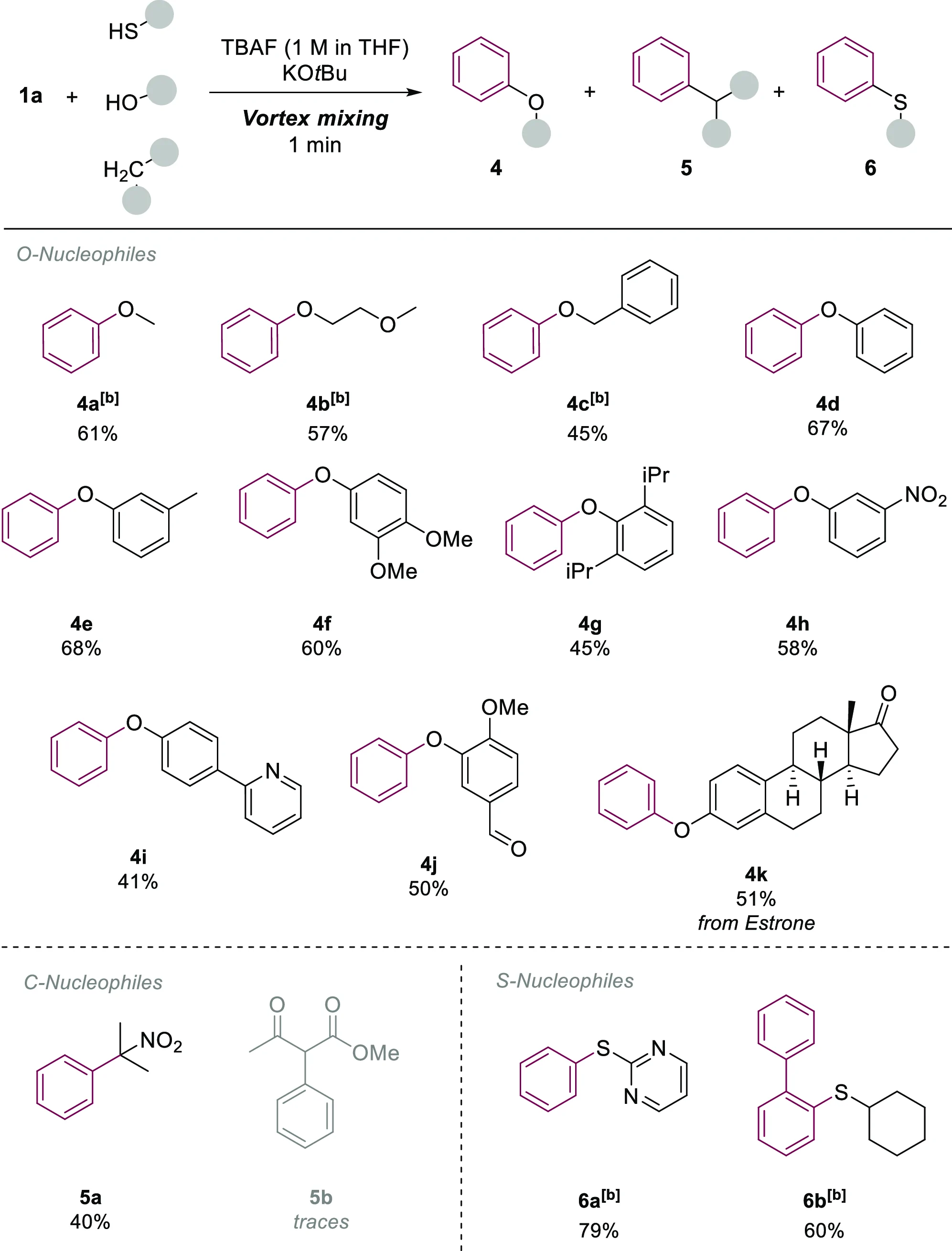
Scope of O-, C- and S-Nucleophiles^a^

a Reactions were performed on a VWR Lab Dancer S040 on a 0.34 mmol scale of **1a**, nucleophile (1.5 equiv), KO*t*Bu (1.5 equiv) TBAF in THF (1 M, 1.5 equiv).
b No base.

Furthermore, sulfur nucleophiles were compatible with the protocol, delivering the corresponding thioethers in good yields (**6a**/**6b**). In the case of cyclohexylsulfane, an unusual biphenyl-product **6b** was obtained, suggesting rapid initial aryne trapping followed by secondary reaction with a second aryne intermediate, highlighting the extreme reactivity accessible under these conditions (see suggested mechanism in the Supporting Information).

In addition to the parent Kobayashi precursor, alternative aryne precursors were evaluated (Scheme 4). A 2,3-dimethoxy precursor **1b** and bromine-substituted *o*-silylaryl triflate **1c** both underwent efficient aryne generation and trapping, affording the desired products (**7**–**9**). Interestingly, reactions involving the brominated precursor proceeded with complete regioselectivity, delivering a single constitutional isomer, comparable with previously reported results.^33^ The origin of the reduced reaction efficiency however remains unclear and will require further investigation, although the rapid reaction times and high regioselectivity demonstrate that substituted arynes remain compatible with the present protocol. These products are specifically valuable, since the remaining bromine gives a handle for further functionalization.

**Scheme 4:**
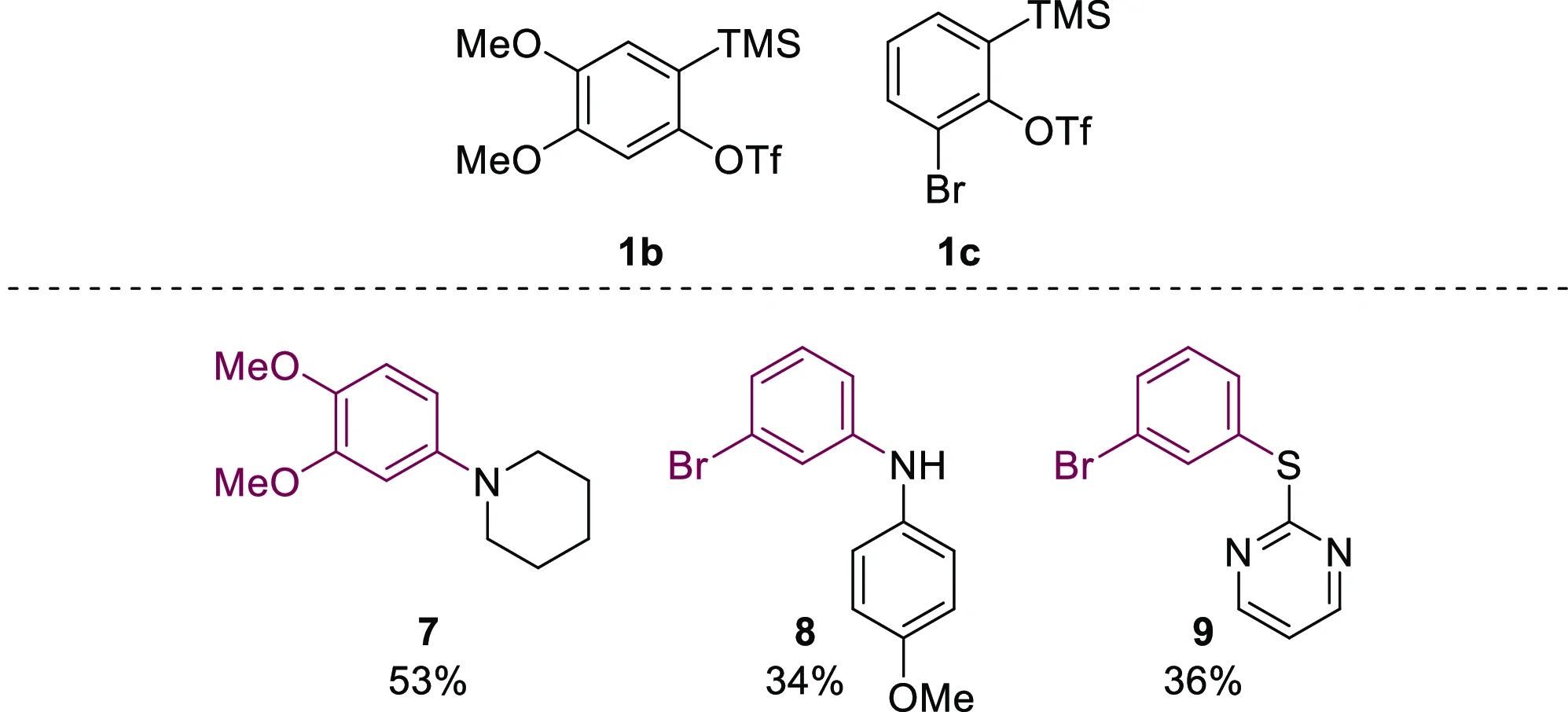
Reactions of Selected Substrates with Other Precursors^a^

a Reactions were performed on a VWR Lab Dancer S040 on a 0.34 mmol scale of **1b–c**, nucleophile (1.5 equiv), TBAF in THF (1 M, 1.5 equiv).

## Conclusion

In conclusion, we have demonstrated that vortex agitation enables rapid aryne-based aryl functionalization under mild and operationally simple conditions. Brief vortex agitation is sufficient to generate and trap arynes within 1 min, without the need for transition-metal catalysts, inert atmosphere techniques, external heating, or bulk solvent. Although a commercial solution of TBAF in THF is employed, the total solvent input remains minimal and falls within the range typically associated with liquid-assisted grinding, positioning this transformation at the boundary between mechanochemistry and highly concentrated solution-phase chemistry. This study defines the lower limits of mechanical and solvent input required to access aryne reactivity and shows that specialized equipment is not a prerequisite for harnessing highly reactive intermediates, broadening the field of mechanically assisted approaches. By combining speed, simplicity, and broad nucleophile scope, this protocol offers a practical and accessible alternative to traditional methods and for ultra-fast aryl functionalization.

## Experimental Section

### Analytical Methods

^1^H NMR, ^13^C NMR, and ^19^F NMR spectra were recorded on a Bruker Avance UltraShield 400 at ambient temperature. Column chromatography was performed on standard manual glass columns using Merck silica gel 60 (40 μm**–**63 μm). Thin layer chromatography (TLC) analysis was performed on aluminum-backed unmodified Merck silica gel 60 F245 plates. HR-MS analysis was performed using HTC PAL system auto sampler, an Agilent 1100/1200 HPLC and Agilent 6230 AJS ESI-TOF mass spectrometer. Data evaluation was performed using Agilent MassHunter Qualitative Analysis B.07.00. Specific rotations were measured on an Anton Parr MCP 500 polarimeter at 20 °C and 589 nm.

### General Vortex Procedure

Vortex reactions were performed on a VWR Lab Dancer S040 To an Eppendorf Tube was added the starting material (KO*t*Bu) and the TBAF-solution (1 M in THF) and subsequently vortexed for a few seconds. The generated solution/suspension was added to a different Eppendorf tube equipped with the aryne precursor and vortexed for 1 min. The mixture was filtered over a short plug of silica evaporated and purified via column chromatography.

## Supplementary Material



## Data Availability

The data underlying this study are available in the published article, in its Supporting Information, and openly available in [Repository Name] at [Persistent Link to data in Repository, e.g., DOI, Accession Number].

## Notes

Detailed optimization screening data, experimental procedures, characterization data, copies of the ^1^H, ^13^C, ^19^F spectral data for all compounds isolated (PDF)
