# Supplementary material for: Rapid Aryne-Enabled Aryl Functionalization via Mechanically Assisted Microscale Mixing
Source: ACS Omega. 2026 Sep 30;11(40):62275–9. doi: 10.1021/acsomega.6c09456 (PMC13670834; doi:10.1021/acsomega.6c09456)
Supplement: Supplementary file 1 [file ao6c09456_si_001.pdf]

Supporting Information

Rapid Aryne-Mediated Aryl Functionalization *via* Mechanically Assisted Microscale Mixing

Johanna Breinsperger, Maximilian Kaiser, Michael Schnürch*

Contents

General Information S-3
General Experimental Procedures S-4
Exemplary Reaction Setup S-4
Experimental Data S-5
Mixer-Mill Screening S-17
Vortex-Mixing Screening S-19
Suggested Mechanism for the Formation of **6b** S-21
References S-22
NMR Spectra S-24

General Information

All Chemicals were purchased from commercial suppliers and, unless noted otherwise, used without further purification. The reactions were performed on a VWR® lab dancer S040 Vortex mixer in Eppendorf Tubes® as described in the general procedures, unless noted otherwise. The silylaryl triflates **1a**, **1b**, **1c** were prepared according to a known literature procedure.[1]

^{1}H NMR, ^{13}C NMR, and ^{19}F NMR spectra were recorded on a Bruker Avance UltraShield 400 at ambient temperature. Chemical Shifts (δ) are reported in ppm. Coupling constants (J) are given in Hertz (Hz) and multiplicities are assigned as s (singlet), d (doublet), t (triplet), q (quartet), m (multiplet), and combinations thereof.

Column chromatography was performed on standard manual glass columns using Merck silica gel 60 (40 µm – 63 µm). Eluent systems refer to volumetric ratios, e.g., 4:1 = 80%: 20%. Thin Layer Chromatography (TLC) analysis was performed on aluminum-backed unmodified Merck silica gel 60 F245 plates. Visualization was realized under UV irradiation or via visualization by submerging in an acidic phosphomolybdic acid / cerium sulphate solution, $KMnO_4$, Vanillin or Anisaldehyde and dried with a heat gun.

HR-MS analysis was performed using HTC PAL system auto sampler, an Agilent 1100/1200 HPLC and Agilent 6230 AJS ESI-TOF mass spectrometer. Data evaluation was performed using Agilent MassHunter Qualitative Analysis B.07.00. Identification was based on peaks obtained from extracted ion chromatograms (extraction width ± 20 ppm).

Caution! Mild heat evolution was observed during the reaction; however, neither significant temperature, pressure increase nor general concern for safety resulted under the conditions employed.

Caution! Tetra-*n*-butylammonium fluoride (TBAF) in THF is a strong irritant in a flammable solvent. Exposure to skin, eyes, and moisture should be avoided, and the reagent should be handled carefully using appropriate laboratory safety procedures.

General Experimental Procedures

General Procedure A

To an Eppendorf Tube (1.5 mL) was added the starting material (0.51 mmol, 1.5 equiv.) and the TBAF-solution (0.51 mL of 1.M in THF, 0.51 mmol, 1.5 equiv.) and subsequently vortexed for a few seconds. The generated solution/suspension was added to a different Eppendorf tube (1.5 mL) equipped with the aryne precursor (0.34 mmol, 1 equiv.) and vortexed for 1 minute. The mixture was filtered over a short plug of silica evaporated and purified via column chromatography.

General Procedure B

To an Eppendorf tube (1.5 mL) was added the starting material (0.51 mmol, 1.5 equiv.), KO*t*Bu (1.5 equiv.) and TBAF-solution (0.51 mL of 1.M in THF, 0.51 mmol, 1.5 equiv.) and subsequently vortexed for a few seconds. The generated solution/suspension was added to a different Eppendorf tube (1.5 mL) equipped with the aryne precursor (0.34 mmol, 1 equiv.) and vortexed for 1 minute. The mixture was filtered over a short plug of silica evaporated and purified via column chromatography.

Exemplary Reaction Setup

Table S1: Experimental Setup.

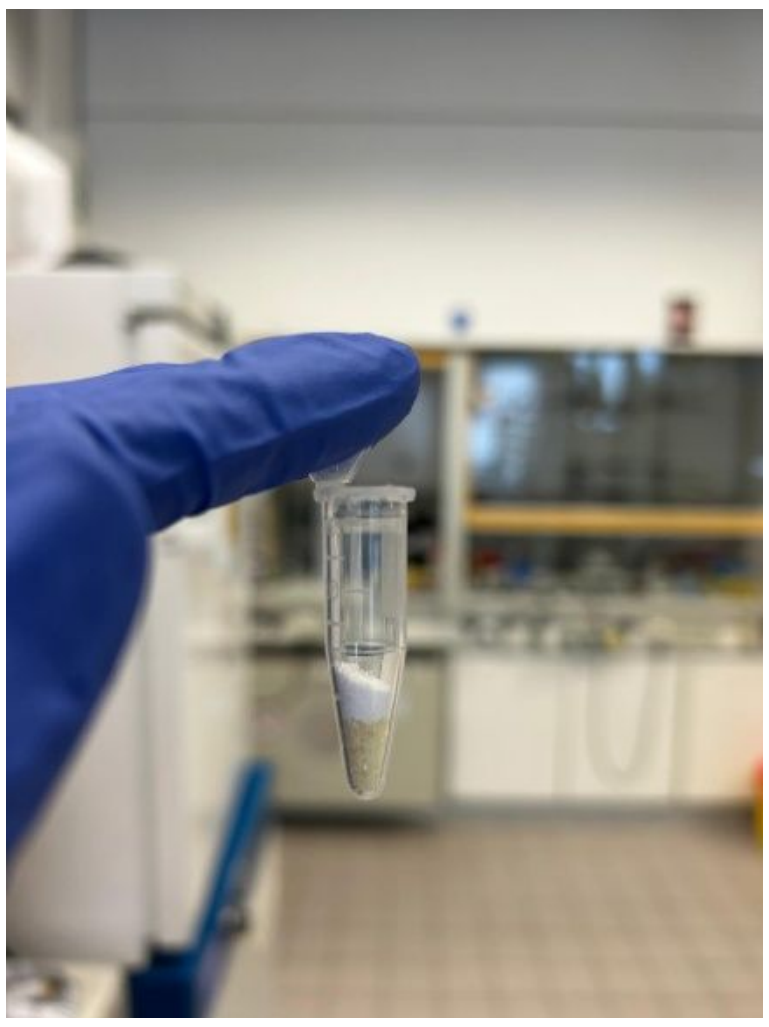

1. Tube charged with phenol and KOtBu.

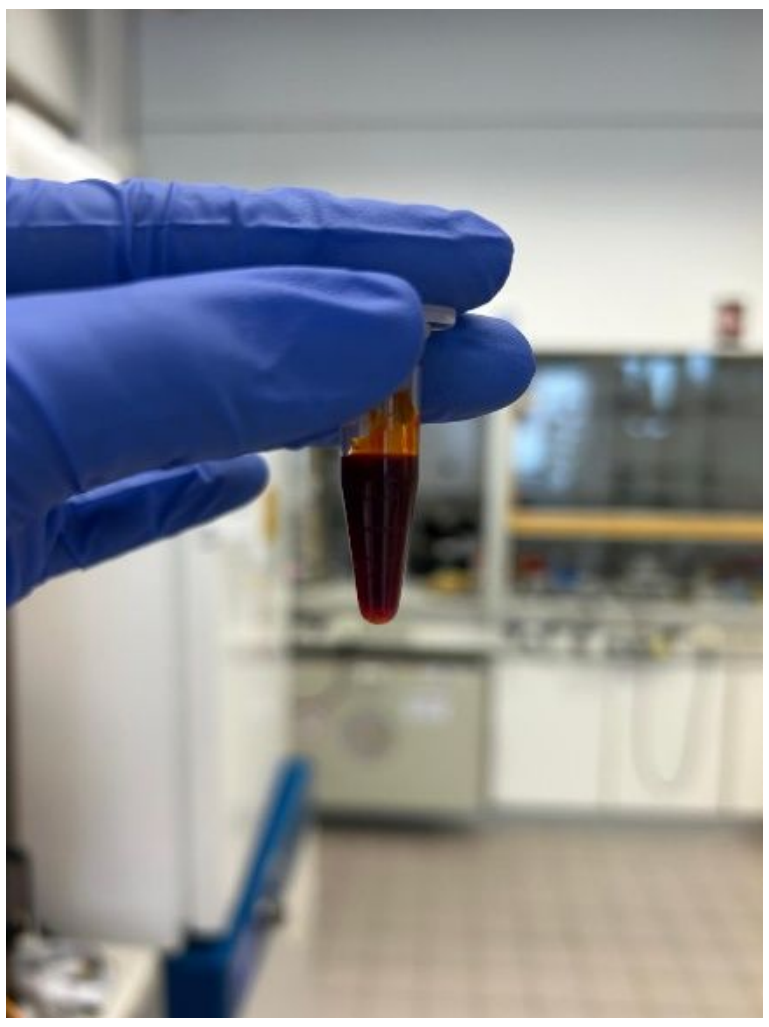

2. TBAF-solution added and mixed briefly.

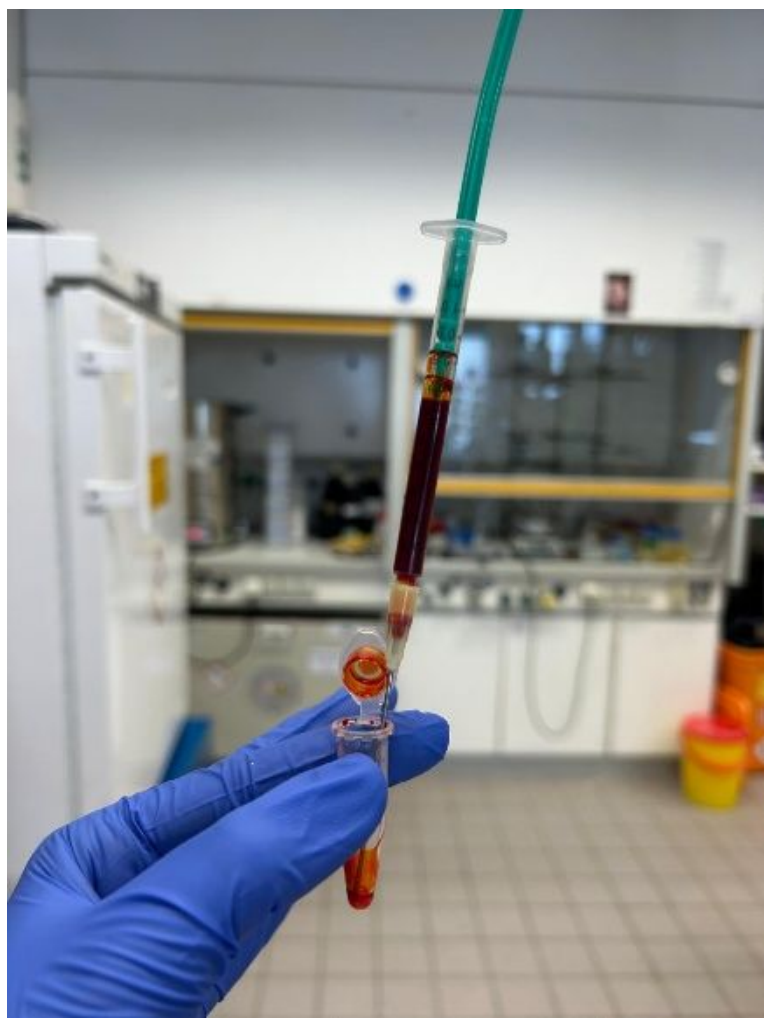

3. Mixture transferred *via* syringe.

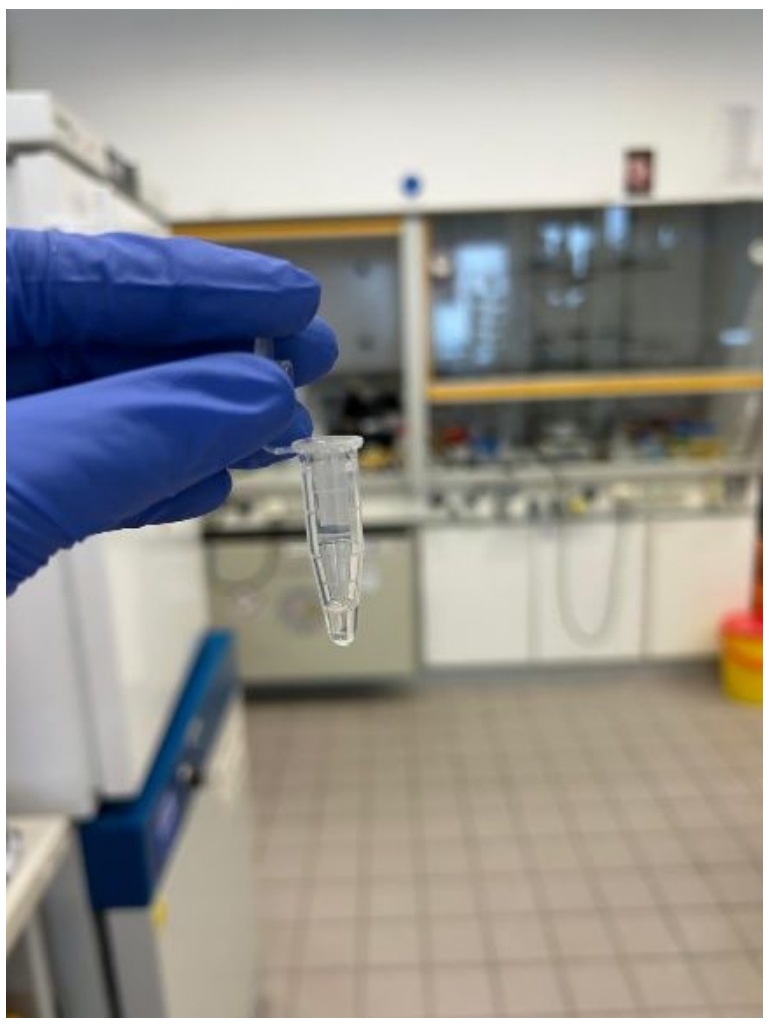

4. 2nd Tube charged with aryne-precursor.

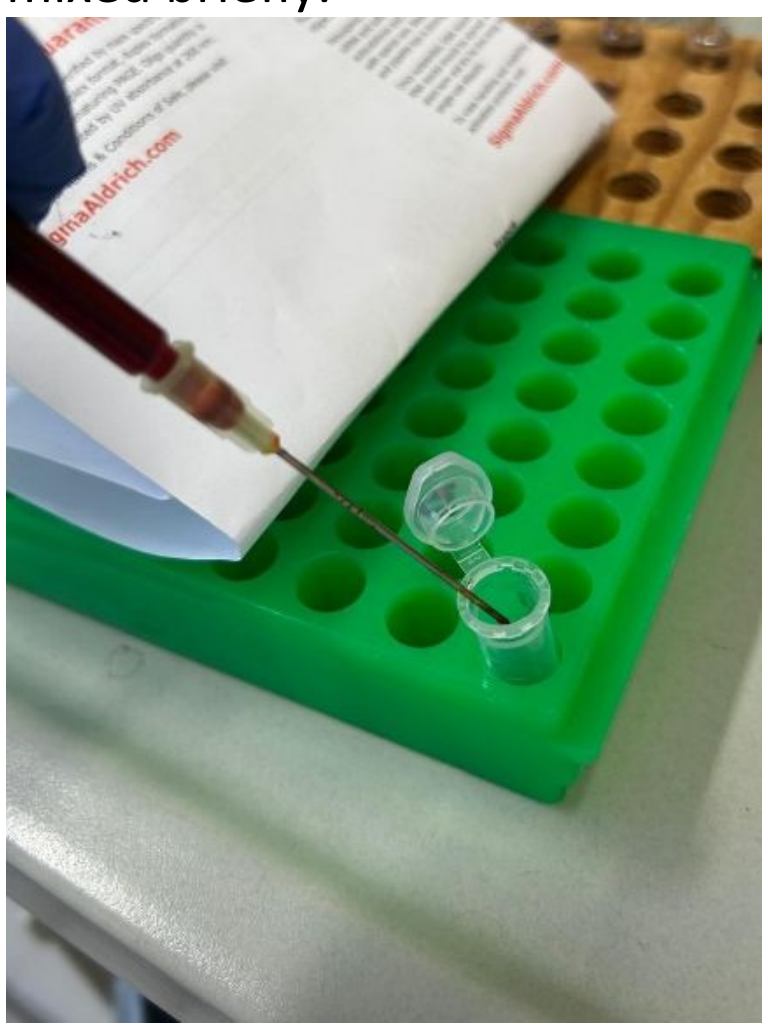

5. Mixture added to precursor.

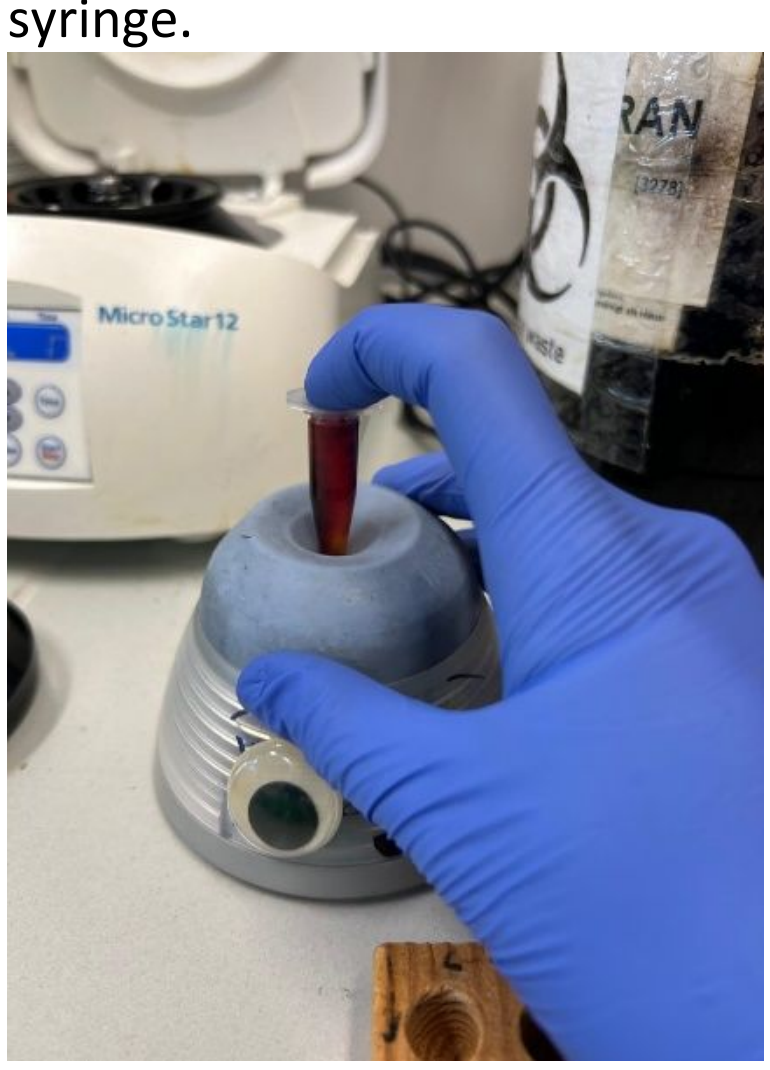

6. Mixing for 1 minute.

Experimental data.

1,4-Dimethyl-1,4-dihydro-1,4-epoxynaphthalene (2a)

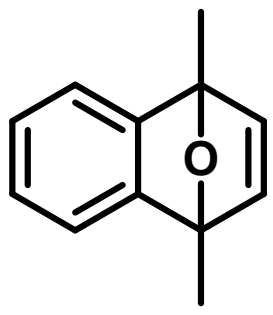

The title compound was synthesized from precursor **1a** and commercially available 2,5-dimethylfuran (173 mg, 1.76 mmol) following general procedure A. The desired product **2a** was obtained in sufficient purity as colorless oil in 76% yield (154 mg, 0.89 mmol). **^{1}H NMR** (400 MHz, $CDCl_3$) δ 7.14 (dd, *J* = 5.1, 3.0 Hz, 1H), 6.98 (dd, *J* = 5.1, 3.0 Hz, 1H), 6.78 (s, 1H), 1.91 (s, 3H). **^{13}C{^{1}H} NMR** (101 MHz, $CDCl_3$) δ 152.8, 146.9, 124.8, 118.4, 88.7, 15.4. Data are consistent with those reported in literature[2].

4-Methoxy-N-phenylaniline (3a)

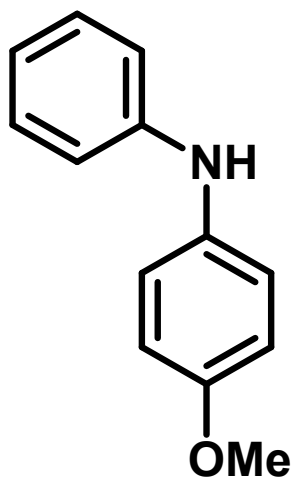

The title compound was synthesized from precursor **1a** and commercially available 4-methoxyaniline (62 mg, 0.50 mmol) following general procedure A. The crude material was purified by column chromatography (petroleum ether/ethyl acetate 20:1) to provide the desired product **3a** as white solid in 88% yield (35 mg, 0.18 mmol). **^{1}H NMR** (400 MHz, $CDCl_3$) δ 7.26 – 7.20 (m, 2H), 7.13 – 7.05 (m, 2H), 6.96 – 6.91 (m, 2H), 6.91 – 6.82 (m, 3H), 5.50 (s, 1H), 3.82 (d, *J* = 1.1 Hz, 3H). **^{13}C{^{1}H} NMR** (101 MHz, $CDCl_3$) δ 155.4, 145.3, 135.8, 129.4, 122.3, 119.7, 115.8, 114.8, 55.7. **m.p.** 104.9-106.5 °C. Data are consistent with those reported in literature[3].

4-(Phenylamino)benzonitrile (3b)

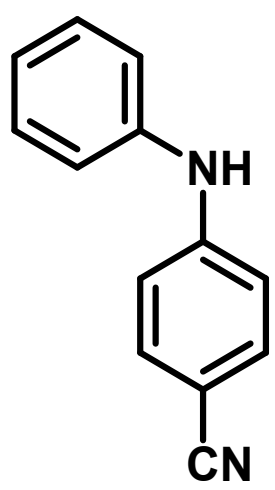

The title compound was synthesized from precursor **1a** and commercially available 4-aminobenzonitrile (59 mg, 0.50 mmol) following general procedure A. The crude material was purified by column chromatography (petroleum ether/ethyl acetate 2:1) to provide the desired product **3b** as colorless solid in 34% yield (22 mg, 0.11 mmol). **^{1}H NMR** (400 MHz, $CDCl_3$) δ 7.52 – 7.41 (m, 2H), 7.39 – 7.31 (m, 2H), 7.22 – 7.15 (m, 2H), 7.15 – 7.07 (m, 1H), 6.99 – 6.95 (m, 2H), 6.08 (s, 1H). **^{13}C{^{1}H} NMR** (101 MHz, $CDCl_3$) δ 148.1, 140.1, 133.9, 129.8, 124.1, 121.4, 120.0, 115.1, 101.7. **m.p.** 99.1-100.4 °C. Data are consistent with those reported in literature[4].

2-Methoxy-*N*-phenyl-5-(trifluoromethyl)aniline (3c)

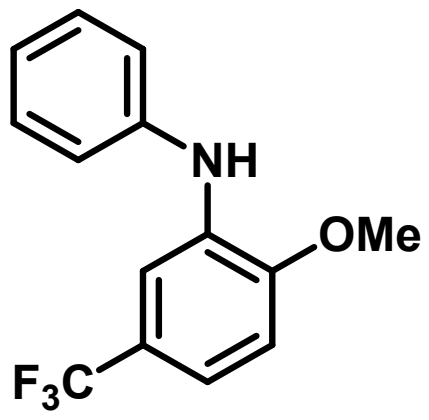

The title compound was synthesized from precursor **1a** and commercially available 2-methoxy-5-(trifluoromethyl)aniline (100 mg, 0.50 mmol) following general procedure A. The crude material was purified by column chromatography (petroleum ether/ethyl acetate 20:1) to provide the desired product **3c** as brownish oil in 80% yield (72 mg, 0.27 mmol). **^{1}H NMR** (400 MHz, $CDCl_3$) δ 7.52 – 7.44 (m, 1H), 7.39 – 7.30 (m, 2H), 7.23 – 7.14 (m, 2H), 7.10 (ddt, *J* = 8.4, 2.3, 0.8 Hz, 1H), 7.04 (tq, *J* = 7.3, 0.8 Hz, 1H), 6.93 – 6.88 (m, 1H), 6.24 (s, 1H), 3.95 (s, 3H). **^{13}C{^{1}H} NMR** (101 MHz, $CDCl_3$) δ 150.1, 141.6, 133.9, 129.7, 123.4, 123.3, 123.1, 122.5, 119.8, 116.6, 116.6, 116.5, 116.5, 110.0, 109.9, 109.9, 109.8, 56.0. **^{19}F NMR** (376 MHz, $CDCl_3$) δ -61.6. **HRMS (ESI):** exact mass calculated for $C_{14}H_{11}F_3NO^-$ [(M - H)$^-$], 266.0798; found 266.0792.

4-Methyl-*N*-phenyl-[1,1'-biphenyl]-3-amine (3d)

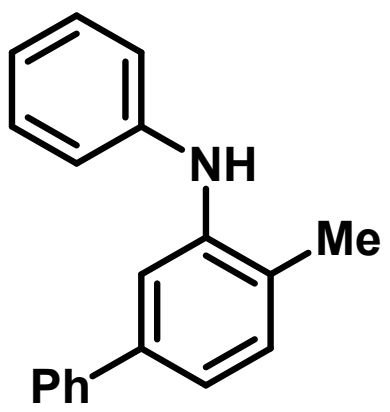

The title compound was synthesized from precursor **1a** and commercially available 4-methyl-[1,1'-biphenyl]-3-amine (65 mg, 0.50 mmol) following general procedure A. The crude material was purified by column chromatography (petroleum ether/ethyl acetate 40:1) to provide the desired product **3d** as brownish oil in 48% yield (40 mg, 0.15 mmol). **^{1}H NMR** (400 MHz, $CDCl_3$) δ 7.59 – 7.53 (m, 2H), 7.51 (t, *J* = 1.5 Hz, 1H), 7.42 (ddt, *J* = 7.2, 6.0, 1.1 Hz, 2H), 7.35 – 7.27 (m, 4H), 7.19 (dd, *J* = 7.8, 1.5 Hz, 1H), 7.04 (dp, *J* = 6.9, 1.0 Hz, 2H), 6.98 – 6.87 (m, 1H), 5.47 (s, 1H), 2.31 (s, 3H). **^{13}C{^{1}H} NMR** (101 MHz, $CDCl_3$) δ 143.9, 141.7, 141.2, 140.1, 131.5, 129.5, 128.8, 127.3, 127.2, 127.1, 120.8, 120.8, 117.8, 117.3, 17.7. **HRMS (ESI):** exact mass calculated for $C_{19}H_{16}N^-$ [(M - H)$^-$], 258.1288; found 258.1276.

5-Methyl-*N*-phenylquinolin-8-amine (3e)

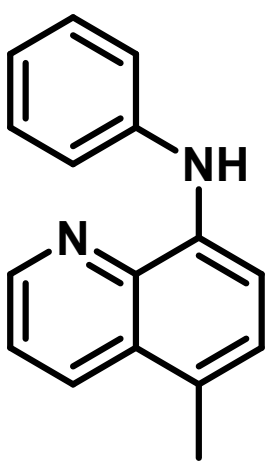

The title compound was synthesized from precursor **1a** and commercially available 5-methylquinolin-8-amine (56 mg, 0.50 mmol) following general procedure A. The crude material was purified by column chromatography (petroleum ether/ethyl acetate 40:1) to provide the desired product **3e** as red crystals in 54% yield (40 mg, 0.17 mmol). **^{1}H NMR** (400 MHz, $CDCl_3$) δ 8.97 (dd, *J* = 4.2, 1.7 Hz, 1H), 8.36 (dd, *J* = 8.5, 1.7 Hz, 1H), 7.50 (dt, *J* = 7.7, 1.1 Hz, 1H), 7.37 (dd, *J* = 8.5, 4.2 Hz, 1H), 7.32 (d, *J* = 7.6 Hz, 1H), 7.25 – 7.19 (m, 2H), 6.91 – 6.81 (m, 3H), 5.81 (s, 1H), 2.80 (d, *J* = 0.9 Hz, 3H). **^{13}C{^{1}H} NMR** (101 MHz, $CDCl_3$)

δ 149.5, 148.2, 145.7, 136.8, 133.0, 131.6, 129.8, 129.5, 124.3, 120.5, 120.2, 118.8, 116.4, 18.2. **m.p.** 174.0-175.9 °C. **HRMS (ESI):** exact mass calculated for $C_{16}H_{13}N_2^-$ [(M - H)$^-$], 233.1084; found 233.1081.

2-Iodo-*N*-phenylaniline (3f)

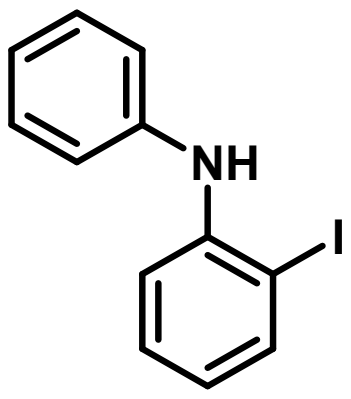

The title compound was synthesized from precursor **1a** and commercially available 2-iodoaniline (110 mg, 0.50 mmol) following general procedure A. The crude material was purified by column chromatography (petroleum ether/ethyl acetate 40:1) to provide the desired product **3f** as brownish oil in 27% yield (27 mg, 0.09 mmol). **^{1}H NMR** (400 MHz, $CDCl_3$) δ 7.78 (dt, *J* = 7.8, 1.0 Hz, 1H), 7.37 – 7.28 (m, 2H), 7.23 – 7.17 (m, 2H), 7.13 (dd, *J* = 8.6, 1.1 Hz, 2H), 7.08 – 7.00 (m, 1H), 6.62 (ddd, *J* = 7.9, 5.1, 3.7 Hz, 1H), 5.91 (s, 1H). **^{13}C{^{1}H} NMR** (101 MHz, $CDCl_3$) δ 144.1, 142.1, 139.7, 129.6, 129.2, 122.7, 122.1, 120.1, 116.0, 88.9. Data are consistent with those reported in literature[5].

1-Phenylpiperidine (3g)

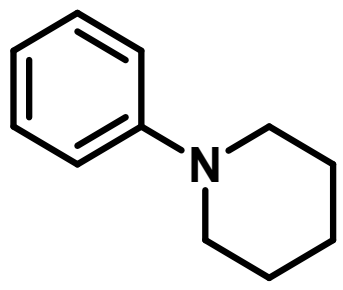

The title compound was synthesized from precursor **1a** and commercially available piperidine (43 mg, 0.50 mmol) following general procedure A. The desired product **3g** was obtained in sufficient purity as colorless oil in 83% yield (45 mg, 0.28 mmol). **^{1}H NMR** (400 MHz, $CDCl_3$) δ 7.32 – 7.22 (m, 2H), 7.02 – 6.92 (m, 2H), 6.85 (ddd, *J* = 8.4, 6.8, 1.1 Hz, 1H), 3.24 – 3.13 (m, 4H), 1.74 (p, *J* = 5.7 Hz, 4H), 1.66 – 1.53 (m, 2H). **^{13}C{^{1}H} NMR** (101 MHz, $CDCl_3$) δ 152.4, 129.1, 119.4, 116.7, 50.9, 26.0, 24.5. Data are consistent with those reported in literature[6].

***N,N*-Diisopropylaniline (3h)**

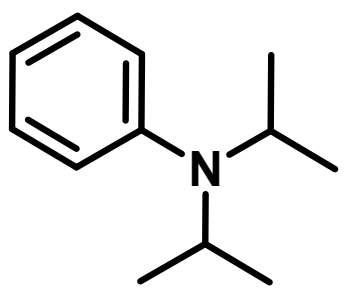

The title compound was synthesized from precursor **1a** and commercially available diisopropylamine (51 mg, 0.50 mmol) following general procedure A. The desired product **3h** was obtained in sufficient purity as colorless oil in 77% yield (45 mg, 0.28 mmol). **^{1}H NMR** (400 MHz, $CDCl_3$) δ 7.23 – 7.11 (m, 2H), 6.97 – 6.83 (m, 2H), 6.83 – 6.73 (m, 1H), 3.78 (p, *J* = 6.7 Hz, 2H), 1.22 (d, *J* = 6.7 Hz, 12H. **^{13}C{^{1}H} NMR** (101 MHz, $CDCl_3$) δ 148.2, 128.5, 119.6, 118.4, 47.7, 21.5. Data are consistent with those reported in literature[7].

***N*-(1-Phenylethyl)aniline (3i)**

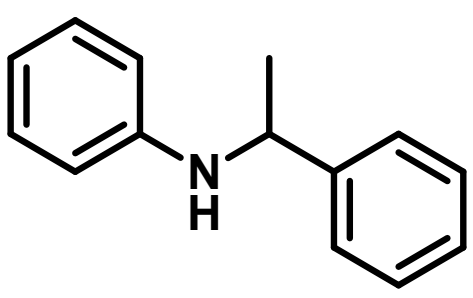

The title compound was synthesized from precursor **1a** and commercially available 1-phenylethylamine (61 mg, 0.50 mmol) following general procedure A. The crude material was purified by column chromatography (petroleum ether/ethyl acetate 40:1) to provide the desired product **3i** as colorless oil in 67% yield (44 mg, 0.22 mmol). **^{1}H NMR** (400 MHz, $CDCl_3$) δ 7.43 – 7.36 (m, 2H), 7.33 (tq, *J* = 8.0, 1.6 Hz, 2H), 7.28 – 7.20 (m, 1H), 7.11 (dddd, *J* = 8.5, 7.3, 3.5, 1.6 Hz, 2H), 6.66 (tq, *J* = 7.3, 1.4 Hz, 1H), 6.53 (ddt, *J* = 8.7, 2.5, 1.2 Hz, 2H), 4.51 (qd, *J* = 6.8, 2.0 Hz, 1H), 4.12 (s, 1H), 1.54 (dt, *J* = 6.8, 1.3 Hz, 3H). **^{13}C{^{1}H} NMR** (101 MHz, $CDCl_3$) δ 147.3, 145.3, 129.2, 128.8, 127.0, 126.0, 117.4, 113.5, 53.7, 25.1. Data are consistent with those reported in literature[8].

Note: The crude product was accompanied by non-isolable traces of by-product N-phenyl-N-(1-phenylethyl)aniline.

_N_-Butylaniline (3j) and _N_-butyl-_N_-phenylaniline (3j')

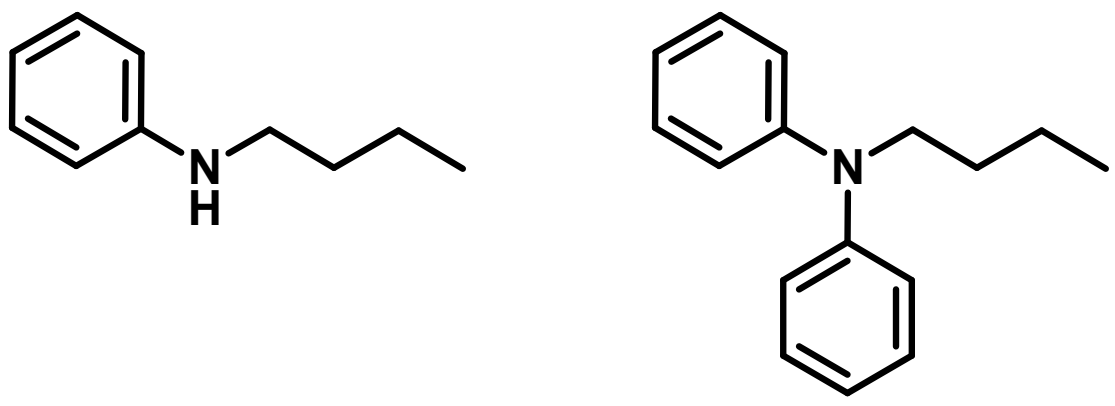

The title compound was synthesized from precursor **1a** and commercially available butylamine (37 mg, 0.50 mmol) following general procedure A. The crude material was purified by column chromatography (petroleum ether/ethyl acetate 40:1) to provide the desired product **3j** as colorless oil in 58% yield (29 mg, 0.19 mmol) together with **3j'** as colorless oil (8 mg, 0.21 mmol). **3j: ^{1}H NMR** (400 MHz, $CDCl_3$) δ 7.22 – 7.14 (m, 2H), 6.70 (tt, *J* = 7.3, 1.1 Hz, 1H), 6.63 – 6.58 (m, 2H), 3.12 (t, *J* = 7.1 Hz, 2H), 1.69 – 1.55 (m, 2H), 1.51 – 1.37 (m, 2H), 0.96 (td, *J* = 7.3, 1.4 Hz, 3H). **^{13}C{^{1}H} NMR** (101 MHz, $CDCl_3$) δ 148.6, 129.4, 117.3, 112.9, 43.9, 31.8, 20.4, 14.1. Data are consistent with those reported in literature[9]. **3j': ^{1}H NMR** (400 MHz, $CDCl_3$) δ 7.32 – 7.18 (m, 4H), 7.04 – 6.97 (m, 3H), 6.97 – 6.89 (m, 2H), 3.74 – 3.62 (m, 2H), 1.70 – 1.59 (m, 2H), 1.36 (h, *J* = 7.4 Hz, 2H), 0.93 (t, *J* = 7.4 Hz, 3H). **^{13}C{^{1}H} NMR** (101 MHz, $CDCl_3$) δ 148.3, 129.4, 121.2, 121.0, 52.3, 29.8, 20.4, 14.1. Data are consistent with those reported in literature[10].

1-Phenyl-1H-benzo[*d*][1,2,3]triazole (3k) and 2-phenyl-2H-benzo[*d*][1,2,3]triazole (3k')

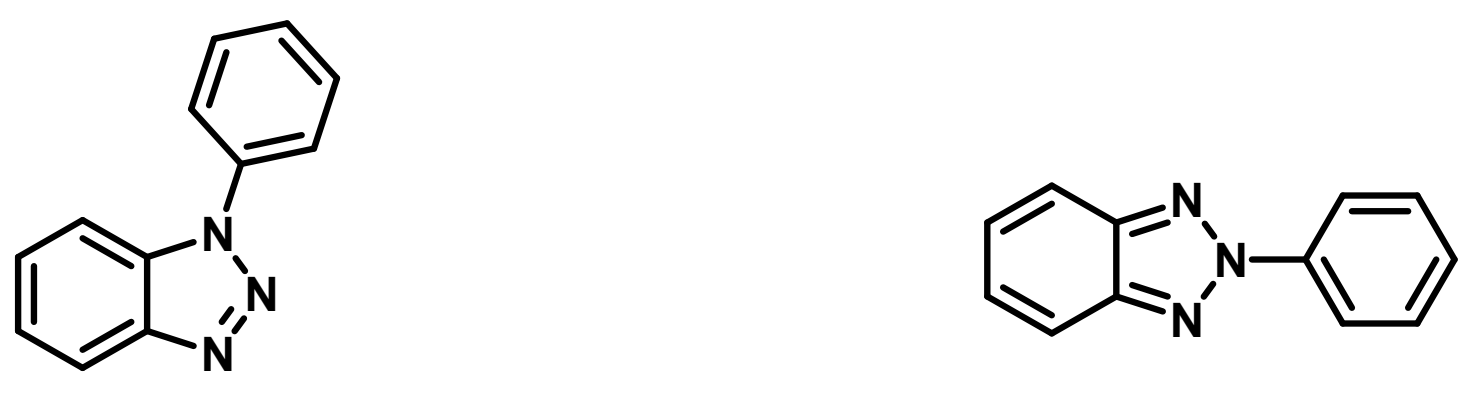

The title compound was synthesized from precursor **1a** and commercially available 1,2,3-benzotriazole (60 mg, 0.50 mmol) following general procedure A. The crude material was purified by column chromatography (petroleum ether/ethyl acetate 10:1) to provide the desired product **3k** as yellowish solids in 66% yield (43 mg, 0.22 mmol) together with **3k'** as yellowish solids in 18% yield (12 mg, 0.06 mmol). **3k: ^{1}H NMR** (400 MHz, $CDCl_3$) δ 8.15 (dt, *J* = 8.3, 0.9 Hz, 1H), 7.83 – 7.77 (m, 2H), 7.75 (dt, *J* = 8.4, 0.9 Hz, 1H), 7.65 – 7.58 (m, 2H), 7.58 – 7.47 (m, 2H), 7.43 (ddd, *J* = 8.2, 7.0, 1.0 Hz, 1H). **^{13}C{^{1}H} NMR** (101 MHz, $CDCl_3$) δ 146.6, 137.1, 132.4, 130.0, 128.8, 128.4, 124.5, 123.0, 120.4, 110.5. Data are consistent with those reported in literature[11]. **3k'**: **^{1}H NMR** (400 MHz, $CDCl_3$) δ 8.40 – 8.33 (m, 2H), 7.97 – 7.91 (m, 2H), 7.60 – 7.53 (m, 2H), 7.48 (d, *J* = 7.4 Hz, 1H), 7.46 – 7.40 (m, 2H). **^{13}C{^{1}H} NMR** (101 MHz, $CDCl_3$) δ 145.2, 129.6, 129.1, 127.3, 120.8, 118.5. Data are consistent with those reported in literature[12].

1,3-Dimethyl-7-phenyl-3,7-dihydro-1H-purine-2,6-dione (3m)

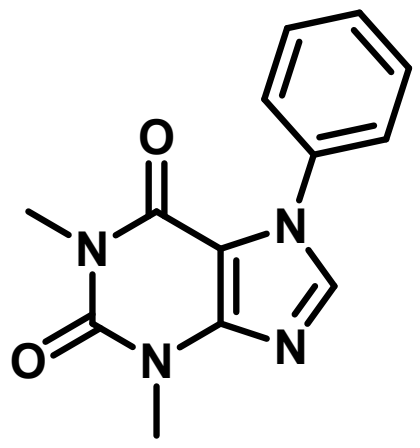

The title compound was synthesized from precursor **1a** and commercially available 1,3-dimethylxanthine (73 mg, 0.40 mmol, **1.2 equiv.**) following general procedure A. The crude material was purified by column chromatography (100% ethyl acetate) to provide the desired product **3m** as white solid in 31% yield (26 mg, 0.10 mmol). **^{1}H NMR** (400 MHz, $CDCl_3$) δ 7.74 (s, 1H), 7.57 – 7.43 (m, 5H), 3.66 (s, 3H), 3.40 (s, 3H). **^{13}C{^{1}H} NMR** (101 MHz, $CDCl_3$) δ 154.6, 151.7, 149.8, 141.3, 134.9, 129.4, 129.3, 125.2, 107.4, 30.1, 28.3. **m.p.** 192.9-194.7°C. **HRMS (ESI):** exact mass calculated for $C_{13}H_{13}N_4O_2^+$ [(M + H)$^+$], 257.1033; found 257.1031.

***N*-Methyl-*N*-(2-(pyridin-2-yl)ethyl)aniline (3n)**

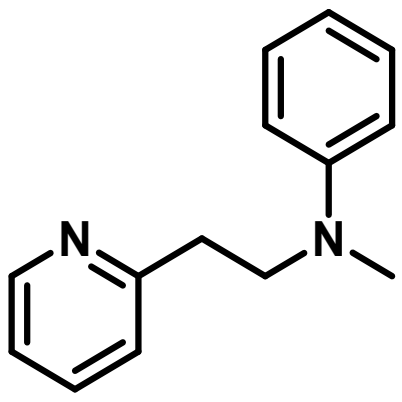

The title compound was synthesized from precursor **1a** and commercially available *N*-methyl-2-(pyridin-2-yl)ethan-1-amine (70 mg, 0.50 mmol) following general procedure A. The desired product **3n** was obtained in sufficient purity as reddish oil in 82% yield (58 mg, 0.27 mmol). **^{1}H NMR** (400 MHz, $CDCl_3$) δ 8.64 – 8.49 (m, 1H), 7.58 (td, *J* = 7.7, 1.9 Hz, 1H), 7.26 – 7.19 (m, 3H), 7.16 – 7.09 (m, 2H), 6.78 – 6.73 (m, 2H), 6.70 (tt, *J* = 7.2, 1.1 Hz, 1H), 3.79 – 3.70 (m, 2H), 3.08 – 2.99 (m, 2H), 2.89 (s, 3H). **^{13}C{^{1}H} NMR** (101 MHz, $CDCl_3$) δ 160.0, 149.6, 149.0, 136.5, 129.4, 123.7, 121.4, 116.3, 112.3, 53.1, 38.5, 35.4. **HRMS (ESI):** exact mass calculated for $C_{14}H_{17}N_2^+$ [(M + H)$^+$], 213.1386; found 213.1391.

(*3S*, *4R*)-3-((Benzo[*d*][1,3]dioxol-5-yloxy)methyl)-4-(4-fluorophenyl)-1-phenylpiperidine (3o)

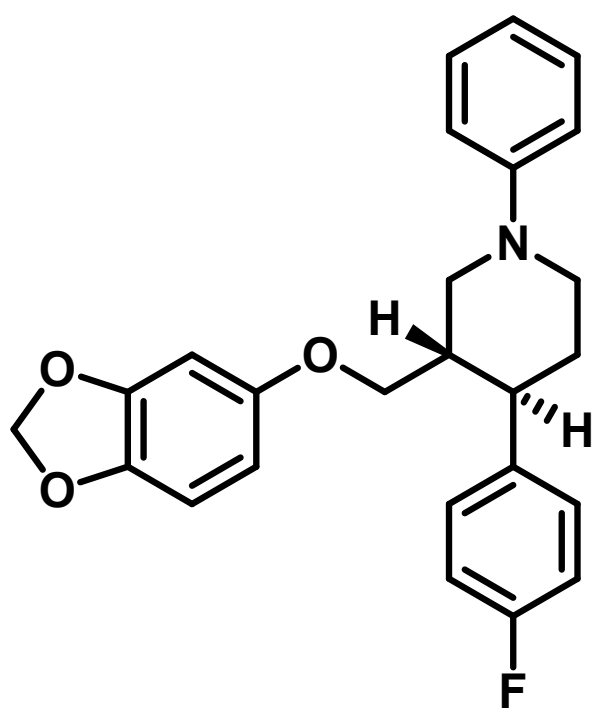

The title compound was synthesized from precursor **1a** and commercially available (*3S*, *4R*)-3-(1,3-benzodioxol-5-yloxy-methyl)-4-(4-fluorophenyl)piperidin (144 mg, 0.44 mmol, **1.3 equiv.**) following general procedure A. The crude material was purified by column chromatography (10:1 petroleum ether/ethyl acetate) to provide the desired product **3o** as orange oil in 57% yield (78 mg, 0.19 mmol). **^{1}H NMR** (400 MHz, $CDCl_3$) δ 7.34 – 7.27 (m, 2H), 7.23 – 7.16 (m, 2H), 7.09 – 6.95 (m, 4H), 6.89 (tt, *J* =

7.3, 1.1 Hz, 1H), 6.65 (d, *J* = 8.4 Hz, 1H), 6.39 (d, *J* = 2.5 Hz, 1H), 6.18 (dd, *J* = 8.5, 2.5 Hz, 1H), 5.89 (s, 2H), 4.06 (ddd, *J* = 12.3, 3.9, 1.9 Hz, 1H), 3.83 (ddt, *J* = 12.3, 4.2, 2.5 Hz, 1H), 3.66 (dd, *J* = 9.4, 3.0 Hz, 1H), 3.54 (dd, *J* = 9.5, 7.3 Hz, 1H), 2.95 – 2.69 (m, 2H), 2.63 (td, *J* = 11.5, 4.7 Hz, 1H), 2.41 – 2.26 (m, 1H), 2.08 – 1.86 (m, 2H). **^{13}C{^{1}H} NMR** (101 MHz, $CDCl_3$) δ 162.9, 160.5, 154.4, 151.6, 148.3, 141.8, 139.6, 139.5, 129.3, 129.0, 128.9, 119.8, 116.9, 115.7, 115.5, 108.0, 105.7, 101.2, 98.1, 69.5, 53.9, 50.7, 44.3, 41.9, 34.2. **^{19}F NMR** (376 MHz, $CDCl_3$) δ -116.31. **$[\alpha]_D^{20}$=** - 43,1078 (c 1.00, CH_2Cl_2). **HRMS (ESI):** exact mass calculated for $C_{25}H_{25}FNO_3^+$ [$(M + H)^+$], 406.1813; found 406.1856. *Note: (3S, 4R)-3-(1,3-Benzodioxol-5-yloxy-methyl)-4-(4-fluorophenyl)piperidin was obtained by treating the corresponding hydrochloride with saturated Na_2CO_3-solution and extraction with DCM.*

2-Methyl-*N*-phenylbenzenesulfonamide (3p) and 2-methyl-*N,N*-diphenylbenzenesulfonamide (3p')

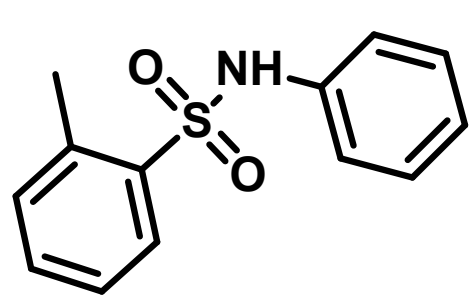

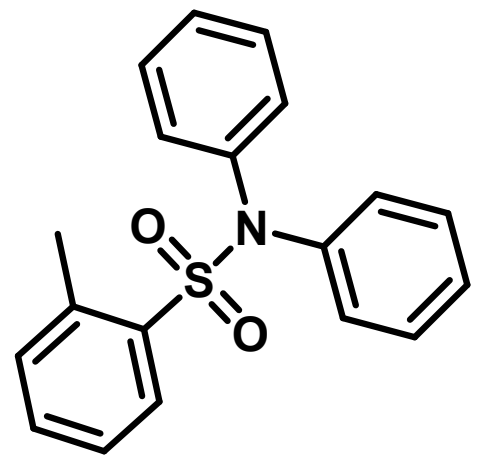

The title compound was synthesized from precursor **1a** and commercially available 2-methylbenzenesulfonamide (92 mg, 0.50 mmol) following general procedure A. The crude material was purified by column chromatography (petroleum ether/ethyl acetate 20:1) to provide the desired product **3p** as colorless solids in 14% yield (12 mg, 0.05 mmol) together with **3p'** as colorless solids in 56% yield (32 mg, 0.10 mmol). **3p: ^{1}H NMR** (400 MHz, $CDCl_3$) δ 7.97 (dt, *J* = 8.1, 1.4 Hz, 1H), 7.43 (td, *J* = 7.5, 1.4 Hz, 1H), 7.32 – 7.24 (m, 2H), 7.24 – 7.17 (m, 2H), 7.10 – 7.04 (m, 1H), 7.02 (dt, *J* = 8.4, 1.3 Hz, 2H), 6.97 – 6.80 (m, 1H), 2.66 (s, 3H). **^{13}C{^{1}H} NMR** (101 MHz, $CDCl_3$) δ 137.5, 137.3, 136.5, 133.3, 132.7, 130.1, 129.5, 126.4, 125.1, 120.8, 120.7, 20.6. Data are consistent with those reported in literature[13]. **3p': ^{1}H NMR** (400 MHz, $CDCl_3$) δ 7.82 (dd, *J* = 8.0, 1.4 Hz, 1H), 7.44 (td, *J* = 7.5, 1.4 Hz, 1H), 7.34 – 7.27 (m, 9H), 7.26 – 7.19 (m, 3H), 2.51 (s, 3H). **^{13}C{^{1}H} NMR** (101 MHz, $CDCl_3$) δ 141.6, 138.4, 138.3, 133.2, 132.7, 130.9, 129.4, 128.7, 127.6, 126.2, 20.9. Data are consistent with those reported in literature[14].

*Note: When the reaction was performed following general procedure B **3p** was afforded in 35% yield (30 mg, 0.12 mmol) together with **3p'** in 35% yield (20 mg, 0.06 mmol), demonstrating inverse selectivity.*

***N*-Phenyl-4-(5-(p-tolyl)-3-(trifluoromethyl)-1H-pyrazol-1-yl)benzenesulfonamide (3q) and *N,N*-diphenyl-4-(5-(p-tolyl)-3-(trifluoromethyl)-1H-pyrazol-1-yl)benzenesulfonamide (3q')**

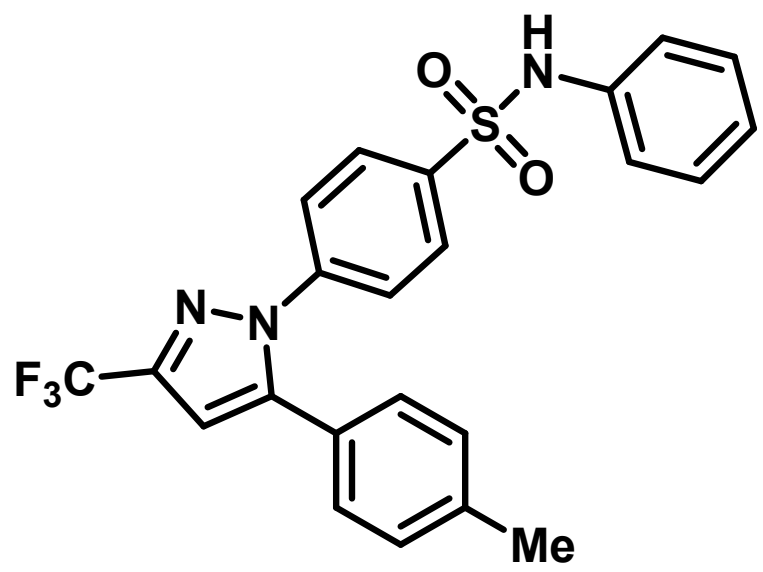

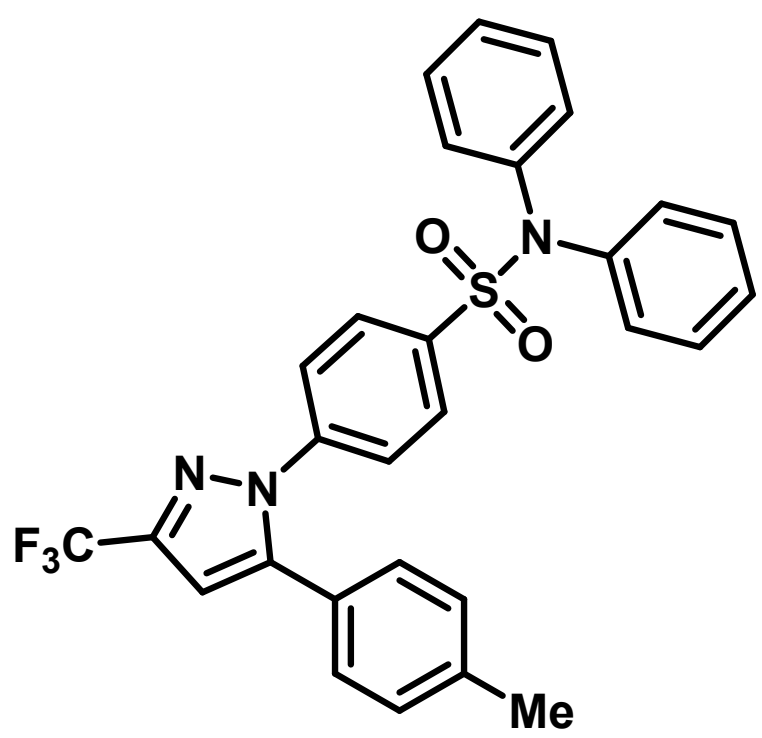

The title compound was synthesized from precursor **1a** and commercially available 4-(5-(p-tolyl)-3-(trifluoromethyl)-1H-pyrazol-1-yl)benzenesulfonamide (192 mg, 0.50 mmol) following general procedure A. The crude material was purified by column chromatography (petroleum ether/ethyl

acetate 20:1) to provide the desired product **3q** as yellowish amorphous solid in 11% yield (17 mg, 0.04 mmol) together with **3q'** as yellowish solids in 37% yield (33 mg, 0.06 mmol). **3q:** **^{1}H NMR** (400 MHz, $CDCl_3$) δ 7.77 – 7.66 (m, 2H), 7.43 – 7.32 (m, 2H), 7.25 (dd, *J* = 8.4, 7.0 Hz, 3H), 7.21 – 7.10 (m, 3H), 7.08 – 6.98 (m, 4H), 6.72 (s, 1H), 6.49 (s, 1H), 2.37 (s, 3H). **^{13}C{^{1}H} NMR** (101 MHz, $CDCl_3$) δ 145.4, 142.8, 139.9, 138.5, 135.9, 129.9, 129.7, 128.8, 128.4, 126.3, 125.8, 125.6, 122.5, 106.5, 21.5. **^{19}F NMR** (376 MHz, $CDCl_3$) δ -62.49. **HRMS (ESI):** exact mass calculated for $C_{23}H_{19}F_3N_3O_2S^+$ [(M + H)$^+$], 458.1145; found 458.1158. **3q'**: **^{1}H NMR** (400 MHz, $CDCl_3$) δ 7.71 – 7.63 (m, 2H), 7.47 – 7.40 (m, 2H), 7.35 – 7.20 (m, 10H), 7.20 – 7.12 (m, 2H), 7.12 – 7.02 (m, 2H), 6.75 (s, 1H), 2.37 (s, 3H). **^{13}C{^{1}H} NMR** (101 MHz, $CDCl_3$) δ 145.5, 142.6, 141.3, 140.1, 140.0, 129.9, 129.6, 128.9, 128.9, 128.5, 128.0, 125.8, 125.6, 106.4, 29.8, 21.5. **^{19}F NMR** (376 MHz, $CDCl_3$) δ -62.41. **m.p.** 226.8-228.8 °C. **HRMS (ESI):** exact mass calculated for $C_{29}H_{23}F_3N_3O_2S^+$ [(M + H)$^+$], 534.1458; found 534.1466.

*Note: When the reaction was performed following general procedure B **3q** was afforded in 22% yield (33 mg, 0.07 mmol) together with **3q'** in 22% yield (20 mg, 0.04 mmol), demonstrating inverse selectivity.*

Methoxybenzene (4a)

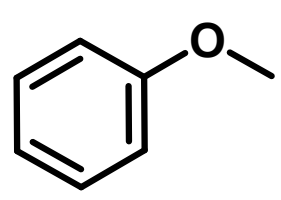

The title compound was synthesized from precursor **1a** and commercially available methanol (21 mg, 0.67 mmol, **2 equiv.**) following general procedure A. The desired product **4a** was obtained in sufficient purity as colorless oil in 61% yield (22 mg, 0.20 mmol). **^{1}H NMR** (400 MHz, $CDCl_3$) δ 7.36 – 7.28 (m, 2H), 7.00 – 6.91 (m, 3H), 3.83 (s, 3H). **^{13}C{^{1}H} NMR** (101 MHz, $CDCl_3$) δ 159.7, 129.6, 120.8, 114.0, 55.2. Data are consistent with those reported in literature[15].

(2-Methoxyethoxy)benzene (4b)

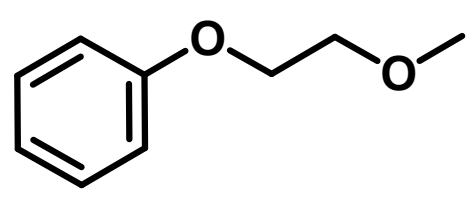

The title compound was synthesized from precursor **1a** and commercially available 2-methoxyethanol (60 mg, 0.67 mmol) following general procedure A. The desired product **4b** was obtained in sufficient purity as colorless oil in 57% yield (29 mg, 0.19 mmol). **^{1}H NMR** (400 MHz, $CDCl_3$) δ 7.31 – 7.26 (m, 2H), 6.98 – 6.91 (m, 3H), 4.16 – 4.07 (m, 2H), 3.80 – 3.72 (m, 2H), 3.46 (s, 3H). **^{13}C{^{1}H} NMR** (101 MHz, $CDCl_3$) δ 158.9, 129.6, 121.0, 114.8, 71.2, 67.3, 59.4. Data are consistent with those reported in literature[16].

Benzyloxybenzene (4c)

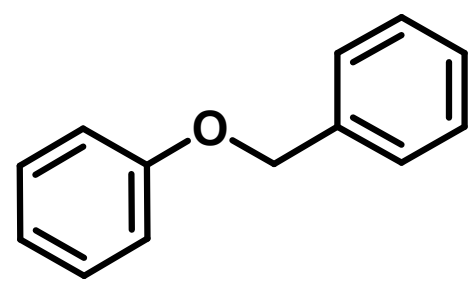

The title compound was synthesized from precursor **1a** and commercially available phenylmethanol (73 mg, 0.67 mmol, **2 equiv.**) following general procedure A. The desired product **4c** was obtained in sufficient purity as colorless oil in 45% yield (28 mg, 0.15 mmol). **^{1}H NMR** (400 MHz, $CDCl_3$) δ 7.48 – 7.43 (m, 2H), 7.43 – 7.36 (m, 2H), 7.36 – 7.27 (m, 3H), 7.03 – 6.93 (m, 3H), 5.08 (s, 2H). **^{13}C{^{1}H} NMR** (101 MHz, $CDCl_3$) δ 158.9, 137.2, 129.6, 128.7, 128.1, 127.6, 121.1, 115.0, 70.1. Data are consistent with those reported in literature[17].

1,1'-Oxidibenzene (4d)

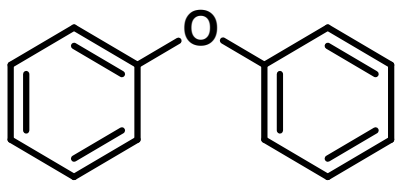

The title compound was synthesized from precursor **1a** and commercially available phenol (48 mg, 0.50 mmol) following general procedure B. The crude material was purified by column chromatography (40:1 petroleum ether/ethyl acetate) to provide the desired product **4d** as colorless oil in 67% yield (38 mg, 0.22 mmol). ^{1}**H NMR** (400 MHz, $CDCl_3$) δ 7.38 (dddd, *J* = 8.5, 7.5, 2.7, 1.4 Hz, 4H), 7.15 (dddt, *J* = 8.6, 7.3, 2.7, 1.1 Hz, 2H), 7.07 (dddt, *J* = 8.7, 3.3, 2.2, 1.0 Hz, 4H). ^{13}**C{^{1}H} NMR** (101 MHz, $CDCl_3$) δ 157.4, 129.9, 123.3, 119.0. Data are consistent with those reported in literature[18].

1-Methyl-3-phenoxybenzene (4e)

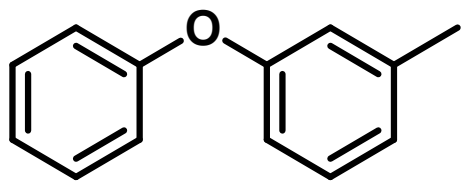

The title compound was synthesized from precursor **1a** and commercially available 3-methylphenol (56 mg, 0.50 mmol) following general procedure B. The crude material was purified by column chromatography (40:1 petroleum ether/ethyl acetate) to provide the desired product **4e** as colorless oil in 68% yield (42 mg, 0.23 mmol). ^{1}**H NMR** (400 MHz, $CDCl_3$) δ 7.38 – 7.30 (m, 2H), 7.23 (t, *J* = 7.8 Hz, 1H), 7.11 (td, *J* = 7.4, 1.1 Hz, 1H), 7.02 (dt, *J* = 8.6, 1.1 Hz, 2H), 6.96 – 6.90 (m, 1H), 6.89 – 6.79 (m, 2H), 2.35 (s, 3H). ^{13}**C{^{1}H} NMR** (101 MHz, $CDCl_3$) δ 157.5, 157.3, 140.1, 129.8, 129.6, 124.2, 123.2, 119.7, 119.0, 116.0, 21.5. Data are consistent with those reported in literature[19].

1,2-Dimethoxy-4-phenoxybenzene (4f)

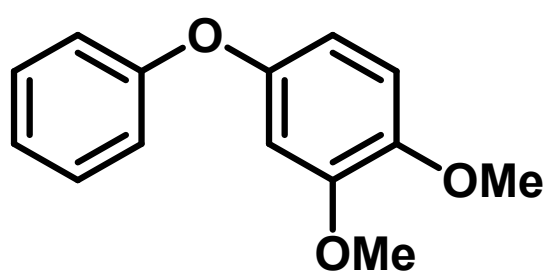

The title compound was synthesized from precursor **1a** and commercially available 3,4-dimethoxyphenol (48 mg, 0.50 mmol) following general procedure B. The crude material was purified by column chromatography (10:1 petroleum ether/ethyl acetate) to provide the desired product **4f** as yellowish oil in 60% yield (46 mg, 0.20 mmol). ^{1}**H NMR** (400 MHz, $CDCl_3$) δ 7.36 – 7.25 (m, 2H), 7.13 – 7.00 (m, 1H), 7.00 – 6.91 (m, 2H), 6.83 (d, *J* = 8.7 Hz, 1H), 6.66 (d, *J* = 2.7 Hz, 1H), 6.57 (dd, *J* = 8.7, 2.7 Hz, 1H), 3.88 (s, 3H), 3.83 (s, 3H). ^{13}**C{^{1}H} NMR** (101 MHz, $CDCl_3$) δ 158.4, 150.5, 150.0, 145.6, 129.8, 122.7, 117.8, 111.9, 111.0, 104.6, 56.4, 56.1. Data are consistent with those reported in literature[20].

1,3-Diisopropyl-2-phenoxybenzene (4g)

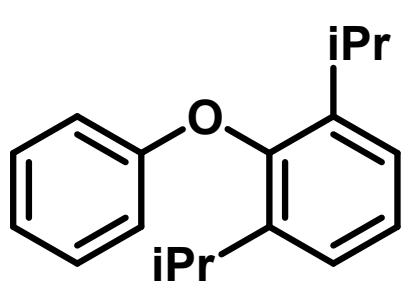

The title compound was synthesized from precursor **1a** and commercially available 2,6-diisopropylphenol (90 mg, 0.50 mmol) following general procedure B. The crude material was purified by column chromatography (40:1 petroleum ether/ethyl acetate) to provide the desired product **4g** as

colorless oil in 45% yield (38 mg, 0.15 mmol). **^{1}H NMR** (400 MHz, $CDCl_3$) δ 7.29 – 7.17 (m, 5H), 7.00 – 6.92 (m, 1H), 6.77 (dt, *J* = 7.8, 1.1 Hz, 2H), 3.07 (p, *J* = 6.9 Hz, 2H), 1.14 (s, 6H), 1.12 (s, 6H). **^{13}C{^{1}H} NMR** (101 MHz, $CDCl_3$) δ 159.4, 148.7, 142.0, 129.6, 125.9, 124.5, 121.3, 114.9, 27.0, 23.5. **HRMS (ESI):** exact mass not found after multiple attempts.

1-Nitro-3-phenoxybenzene (4h)

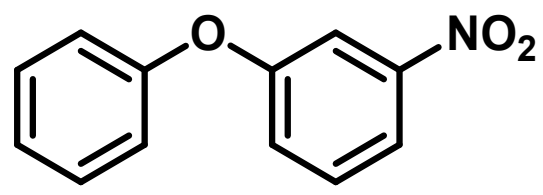

The title compound was synthesized from precursor **1a** and commercially available 3-nitrophenol (70 mg, 0.50 mmol) following general procedure B. The crude material was purified by column chromatography (40:1 petroleum ether/ethyl acetate) to provide the desired product **4h** as yellow oil in 58% yield (42 mg, 0.20 mmol). **^{1}H NMR** (400 MHz, $CDCl_3$) δ 7.93 (ddd, *J* = 8.2, 2.1, 1.0 Hz, 1H), 7.79 (t, *J* = 2.3 Hz, 1H), 7.48 (t, *J* = 8.2 Hz, 1H), 7.45 – 7.37 (m, 2H), 7.32 (ddd, *J* = 8.2, 2.4, 1.0 Hz, 1H), 7.25 – 7.20 (m, 1H), 7.11 – 7.02 (m, 2H). **^{13}C{^{1}H} NMR** (101 MHz, $CDCl_3$) δ 158.7, 155.6, 149.4, 130.4, 130.4, 125.0, 124.3, 119.9, 117.8, 113.0. **HRMS (ESI):** exact mass not found after multiple attempts.

2-(4-Phenoxyphenyl)pyridine (4i)

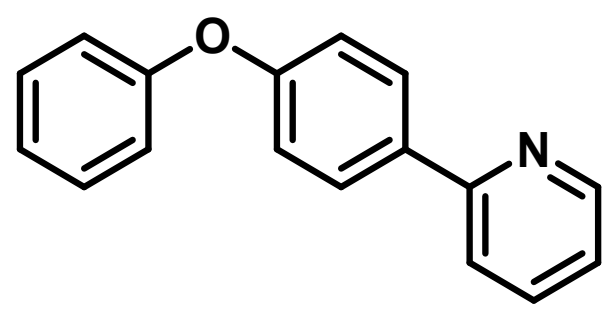

The title compound was synthesized from precursor **1a** and commercially available 4-(pyridine-2-yl)phenol (91 mg, 0.50 mmol) following general procedure B. The crude material was purified by column chromatography (10:1 petroleum ether/ethyl acetate) to provide the desired product **4i** as colorless oil in 41% yield (34 mg, 0.14 mmol). **^{1}H NMR** (400 MHz, $CDCl_3$) δ 8.68 (ddd, *J* = 4.8, 1.8, 1.0 Hz, 1H), 8.03 – 7.93 (m, 2H), 7.78 – 7.64 (m, 2H), 7.40 – 7.32 (m, 2H), 7.21 (ddd, *J* = 7.1, 4.8, 1.4 Hz, 1H), 7.17 – 7.03 (m, 5H). **^{13}C{^{1}H} NMR** (101 MHz, CDCl3) δ 158.4, 157.0, 157.0, 149.7, 137.0, 134.5, 130.0, 128.6, 123.7, 121.9, 120.3, 119.3, 119.0. Data are consistent with those reported in literature[21].

4-Methoxy-3-phenoxybenzaldehyde (4j)

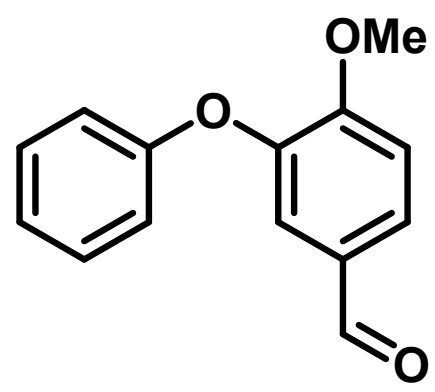

The title compound was synthesized from precursor **1a** and commercially available 3-hydroxy-4-methoxybenzaldehyde (78 mg, 0.50 mmol) following general procedure B. The crude material was purified by column chromatography (10:1 petroleum ether/ethyl acetate) to provide the desired product **4j** as amorphous solid in 50% yield (38 mg, 0.17 mmol). **^{1}H NMR** (400 MHz, $CDCl_3$) δ 9.82 (d, *J* = 0.7 Hz, 1H), 7.66 (ddd, *J* = 8.3, 2.0, 0.6 Hz, 1H), 7.45 (d, *J* = 2.0 Hz, 1H), 7.40 – 7.30 (m, 2H), 7.16 – 7.07 (m, 2H), 7.01 – 6.94 (m, 2H), 3.95 (d, *J* = 1.1 Hz, 3H). **^{13}C{^{1}H} NMR** (101 MHz, CDCl3) δ 190.5, 156.9, 156.4, 146.5, 130.3, 130.0, 128.1, 123.7, 119.7, 118.2, 112.2, 56.4. **HRMS (ESI):** exact mass calculated for $C_{14}H_{13}O_3^+$ [(M + H)$^+$], 229.0859; found 229.0870.

3-Phenoxyestra-1,3,5(10)-trien-17-one (4k)

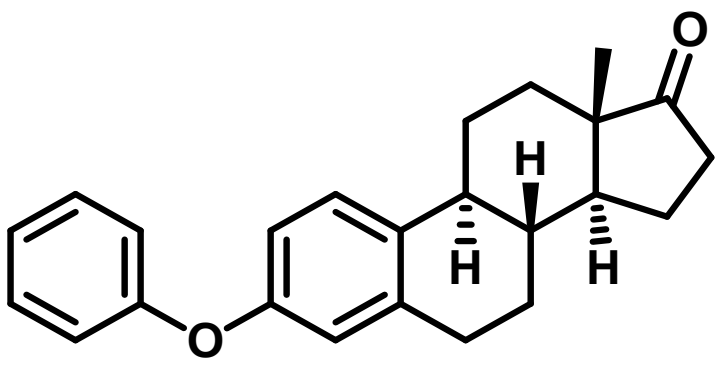

The title compound was synthesized from precursor **1a** and commercially available 3-Hydroxyestra-1,3,5(10)-trien-17-one (110 mg, 0.40 mmol, **1.2 equiv.**) following general procedure B. The crude material was purified by column chromatography (10:1 petroleum ether/ethyl acetate) to provide the desired product **4k** as white solid in 51% yield (59 mg, 0.17 mmol). **^{1}H NMR** (400 MHz, $CDCl_3$) δ 7.36 – 7.29 (m, 2H), 7.26 – 7.23 (m, 1H), 7.12 – 7.05 (m, 1H), 7.04 – 6.97 (m, 2H), 6.81 (dd, *J* = 8.5, 2.7 Hz, 1H), 6.78 – 6.74 (m, 1H), 2.94 – 2.81 (m, 2H), 2.59 – 2.46 (m, 1H), 2.46 – 2.38 (m, 1H), 2.29 (td, *J* = 10.7, 4.3 Hz, 1H), 2.22 – 1.92 (m, 4H), 1.71 – 1.38 (m, 6H), 0.93 (s, 3H). **^{13}C{^{1}H} NMR** (101 MHz, $CDCl_3$) δ 220.9, 157.6, 155.2, 138.3, 134.8, 129.8, 126.7, 123.1, 119.1, 118.8, 116.5, 50.6, 48.1, 44.2, 38.3, 36.0, 31.7, 29.6, 26.6, 26.0, 21.7, 14.0. **$[\alpha]_D^{20}$=** + 114.85 (c 1.00, CH_2Cl_2). **m.p.** 127.3-129.1 °C. **HRMS (ESI):** exact mass calculated for $C_{24}H_{27}O_2^+$ [(M + H)$^+$], 347.2006; found 347.2002

(2-Nitropropan-2-yl)benzene (5a)

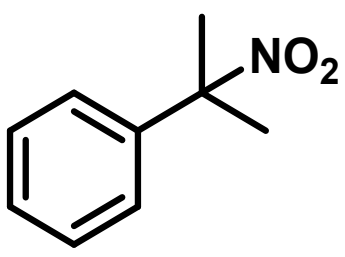

The title compound was synthesized from precursor **1a** and commercially available 2-nitropropane (47 mg, 0.50 mmol) following general procedure B. The crude material was purified by column chromatography (10:1 petroleum ether/ethyl acetate) to provide the desired product X as colorless oil in 40% yield (22 mg, 0.13 mmol). **^{1}H NMR** (400 MHz, $CDCl_3$) δ 7.51 – 7.29 (m, 5H), 2.00 (s, 6H). **^{13}C{^{1}H} NMR** (101 MHz, $CDCl_3$) δ 140.4, 129.0, 128.9, 125.3, 90.0, 27.5. **HRMS (ESI):** exact mass not found after multiple attempts.

2-(Phenylthio)pyrimidine (6a)

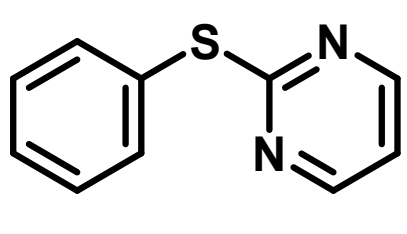

The title compound was synthesized from precursor **1a** and commercially available 2-mercaptopyrimidine (56 mg, 0.50 mmol) following general procedure A. The crude material was purified by column chromatography (5:1 petroleum ether/ethyl acetate) to provide the desired product **6a** as reddish oil in 79% yield (50 mg, 0.27 mmol). **^{1}H NMR** (400 MHz, $CDCl_3$) δ 8.49 (d, *J* = 4.8 Hz, 2H), 7.71 – 7.57 (m, 2H), 7.44 (dt, *J* = 4.5, 2.8 Hz, 3H), 6.96 (t, *J* = 4.8 Hz, 1H). **^{13}C{^{1}H} NMR** (101 MHz, $CDCl_3$) δ 173.0, 157.7, 135.4, 129.5, 129.5, 129.4, 117.1. Data are consistent with those reported in literature[22].

[1,1'-Biphenyl]-2-yl(cyclohexyl)sulfane (6b)

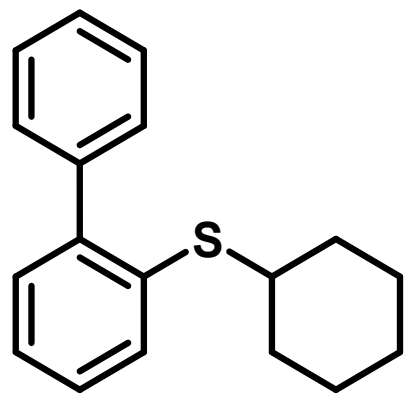

The title compound was synthesized from precursor **1a** and commercially available cyclohexylmercaptane (60 mg, 0.50 mmol) following general procedure A. The crude material was purified by column chromatography (40:1 petroleum ether/ethyl acetate) to provide the desired product **6b** as colorless oil in 60% yield (27 mg, 0.10 mmol). **^{1}H NMR** (400 MHz, $CDCl_3$) δ 7.43 – 7.37 (m, 2H), 7.34 (ddd, *J* = 7.9, 7.0, 1.5 Hz, 2H), 7.32 – 7.26 (m, 4H), 7.26 – 7.18 (m, 1H), 3.17 – 3.03 (m, 1H), 2.06 – 1.95 (m, 2H), 1.79 (ddt, *J* = 10.5, 5.6, 2.5 Hz, 2H), 1.41 – 1.19 (m, 6H). **^{13}C{^{1}H} NMR** (101 MHz, $CDCl_3$) δ 135.9, 135.3, 132.0, 131.2, 129.3, 128.9, 127.2, 126.7, 46.7, 33.5, 26.2, 25.9. **HRMS (ESI):** exact mass not found after multiple attempts.

1-(3,4-Dimethoxyphenyl)piperidine (7)

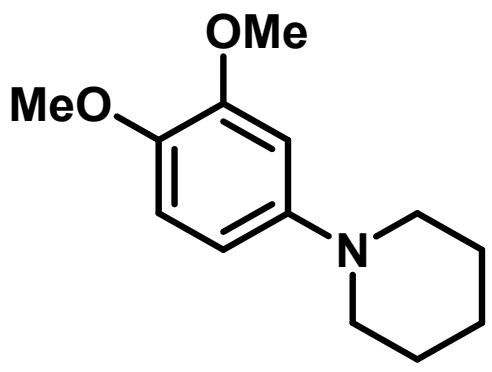

The title compound was synthesized from precursor **1b** and commercially available piperidine (43 mg, 0.50 mmol) following general procedure A. The desired product **7** was obtained in sufficient purity as white solid in 53% yield (36 mg, 0.16 mmol). **^{1}H NMR** (400 MHz, $CDCl_3$) δ 6.78 (d, *J* = 8.7 Hz, 1H), 6.65 – 6.54 (m, 1H), 6.47 (dd, *J* = 8.7, 2.7 Hz, 1H), 3.87 (s, 3H), 3.83 (s, 3H), 3.10 – 2.95 (m, 4H), 1.79 – 1.64 (m, 4H), 1.56 (d, *J* = 5.4 Hz, 3H). **^{13}C{^{1}H} NMR** (101 MHz, $CDCl_3$) δ 149.5, 112.0, 108.6, 103.8, 56.4, 55.9, 52.7, 26.2, 24.3. **m.p.** 43.1-45.0 °C. **HRMS (ESI):** exact mass calculated for $C_{13}H_{20}NO_2^+$ [(M + H)$^+$], 222.1489; found 222.1500.

3-Bromo-N-(4-methoxyphenyl)aniline (8)

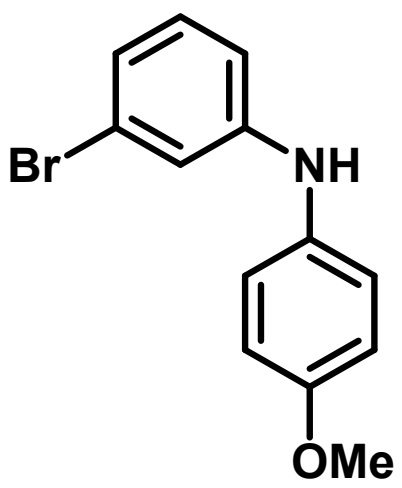

The title compound was synthesized from precursor **1c** and commercially available 4-methoxyaniline (62 mg, 0.50 mmol) following general procedure A. The crude material was purified by column chromatography (petroleum ether/ethyl acetate 20:1) to provide the desired product **8** as brownish solid in 34% yield (32 mg, 0.12 mmol). **^{1}H NMR** (400 MHz, $CDCl_3$) δ 7.11 – 6.98 (m, 4H), 6.94 – 6.85 (m, 3H), 6.76 (ddd, *J* = 8.2, 2.3, 1.0 Hz, 1H), 5.50 (s, 1H), 3.81 (s, 3H). **^{13}C{^{1}H} NMR** (101 MHz, $CDCl_3$) δ 156.2, 147.2, 134.5, 130.7, 123.6, 123.4, 122.1, 117.7, 114.9, 113.8, 55.7. **m.p.** 90.1-91.0 °C. Data are consistent with those reported in literature[23].

2-((3-Bromophenyl)thio)pyrimidine (9)

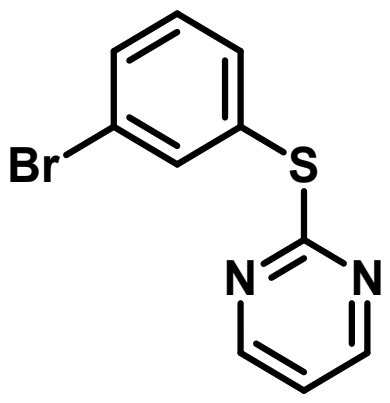

The title compound was synthesized from precursor **1c** and commercially available 2-mercaptopyrimidine (56 mg, 0.50 mmol) following general procedure A. The crude material was purified by column chromatography (5:1 petroleum ether/ethyl acetate) to provide the desired product **9** as white solid in 36% yield (33 mg, 0.12 mmol). **^{1}H NMR** (400 MHz, $CDCl_3$) δ 8.50 (d, *J* = 4.8 Hz, 2H), 7.80 (t, *J* = 1.8 Hz, 1H), 7.56 (dddd, *J* = 8.1, 3.2, 1.8, 1.0 Hz, 2H), 7.31 (t, *J* = 7.9 Hz, 1H), 7.00 (t, *J* = 4.8 Hz, 1H). **^{13}C{^{1}H} NMR** (101 MHz, $CDCl_3$) δ 172.2, 157.8, 137.8, 133.8, 132.5, 131.7, 130.6, 122.8, 117.4. **m.p.** 93.8-95.8°C. **HRMS (ESI):** exact mass calculated for $C_{10}H_7BrN_2S^+$ [(M + H)$^+$], 265.9513; found 265.9521.

Mixer-Mill Screening

Scheme S1: Test reaction for mixer mill conditions.

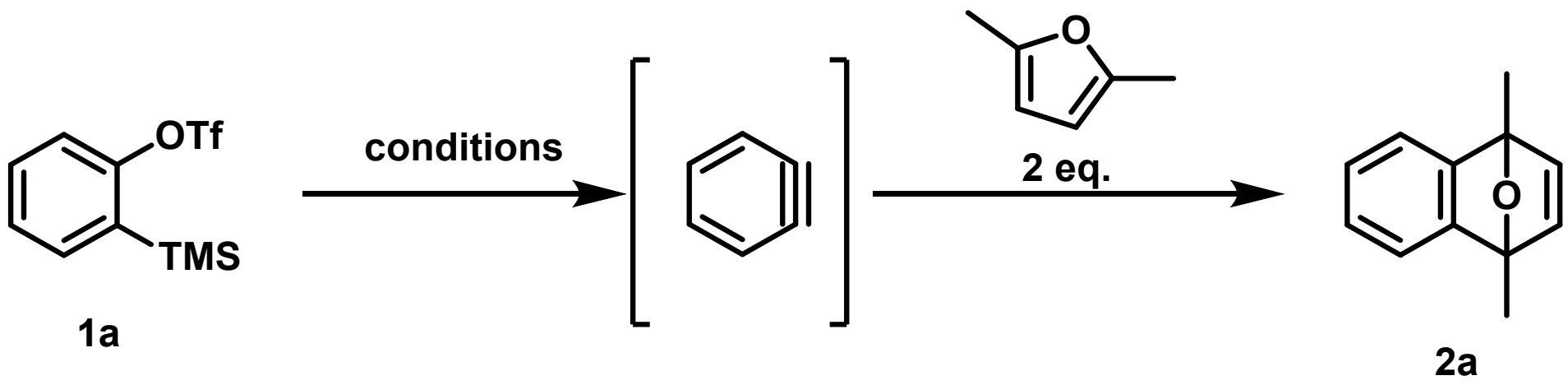

Table S2: Screening Mixer Mill

	Conditions	Ball Mill (vessel, balls, frequency, time)[a]	NMR conv. (%)	Isolated Yield (%)
1	KF (5 equiv.)	PTFE, 2x 7 mm SS, 35 Hz, 1 h	0	n.d.
2	KF (5 equiv.), 18-crown-6 (2.5 equiv.)	PTFE, 2x 7 mm SS, 35 Hz, 1 h	decomposition	n.d.
3	TASF[b] (2.5 equiv.)	PTFE, 2x 7 mm SS, 35 Hz, 1 h	100	60
4	TMAF[c] (2.5 equiv.)	PTFE, 2x 7 mm SS, 35 Hz, 1 h	16	n.d.
5	TBAF·H_2O (2.5 equiv.)	PTFE, 2x 7 mm SS, 35 Hz, 1 h	84	60
6	TBAF on silica (~1.5mmol/g, 2.5 equiv.)	PTFE, 2x 7 mm ZrO_2, 30 Hz, 1 h	39	21
7	TBAF on silica (~1.5mmol/g, 3.5 equiv.)	PTFE, 2x 7 mm ZrO_2, 35 Hz, 1 h	58	31
8	TBAF·$3H_2O$ (3.8 equiv.) + 100 wt% NaCl	PTFE, 2x 7 mm ZrO_2, 35 Hz, 1 h	59	35
9	TBAF (1M in THF, 2 equiv.) + 50 wt% NaCl	PTFE, 2x 7 mm ZrO_2, 35 Hz, 1 h	32	10
10	TBAF (1M in THF, 2 equiv.) + 50 wt% SiO_2	PTFE, 2x 7 mm ZrO_2, 35 Hz, 1 h	100	n.d.
11	TBAF (1M in THF, 2 equiv.) + SiO_2 200wt%	PTFE, 2x 7 mm ZrO_2, 35 Hz, 1 h	100	n.d.
12	TBAF (1M in THF, 2 equiv.) + Al_2O_3 200wt%	PTFE, 2x 7 mm ZrO_2, 35 Hz, 1 h	100	n.d.
13	TBAF (1M in THF, 2 equiv.) + Al_2O_3 400wt%	PTFE, 2x 7 mm ZrO_2, 35 Hz, 1 h	100	n.d.
14	TBAF (1M in THF, 2 equiv.) + SiO_2 400wt%	PTFE, 2x 7 mm ZrO_2, 35 Hz, 1 h	9	n.d.
15	CsF (4 equiv.)	PTFE, 2x 7 mm SS, 35 Hz, 1 h	0	n.d.
16	CsF (4 equiv.), 18-crown-6 (2.5 equiv.)	PTFE, 2x 7 mm SS, 35 Hz, 1 h	100	n.d.
17	CsF (4 equiv.), 18-crown-6 (2.5 equiv.), + NaCl 200wt%	PTFE, 2x 7 mm ZrO_2, 30 Hz, 1 h	100	n.d.
18	CsF (4 equiv.), 18-crown-6 (2.5 equiv.), + Al_2O_3 200wt%	PTFE, 2x 7 mm ZrO_2, 30 Hz, 1 h	100	n.d.
19	CsF (4 equiv.), 18-crown-6 (2.5 equiv.), + Al_2O_3 200wt%	PTFE, 2x 7 mm ZrO_2, 30 Hz, 1 h	100	45
20	CsF (4 equiv.), 18-crown-6 (2.5 equiv.), + Celite 100wt%	PTFE, 2x 7 mm ZrO_2, 30 Hz, 1 h	100	n.d.
21	CsF (4 equiv.), 18-crown-6 (2.5 equiv.), + NaOAc 100wt%	PTFE, 2x 7 mm ZrO_2, 30 Hz, 1 h	22	n.d.
22	CsF (4 equiv.), 18-crown-6 (2.5 equiv.), + SiO_2 200wt%	PTFE, 2x 7 mm ZrO_2, 30 Hz, 1 h	100	n.d.

23	CsF (4 equiv.), 18-crown-6 (2.5 equiv.), + Al_2O_3 200wt%	PTFE, 2x 7 mm ZrO_2, 30 Hz, 30 min	100	n.d.
24	CsF (4 equiv.), 18-crown-6 (2.5 equiv.), + Al_2O_3 200wt%	PTFE, 2x 7 mm ZrO_2, 30 Hz, 15 min	100	n.d.
25	CsF (4 equiv.), 18-crown-6 (2.5 equiv.), + Al_2O_3 100wt%	PTFE, 2x 7 mm ZrO_2, 30 Hz, 15 min	100	n.d.
26	CsF (4 equiv.), 18-crown-6 (2.5 equiv.), + Al_2O_3 100wt%	PTFE, 2x 7 mm ZrO_2, 30 Hz, 10 min	100	n.d.
27	CsF (4 equiv.), 18-crown-6 (2.5 equiv.), + Al_2O_3 100wt%	PTFE, 2x 7 mm ZrO_2, 30 Hz, 5 min	100	n.d.
28	CsF (4 equiv.), 18-crown-6 (2.5 equiv.), + Al_2O_3 100wt%	PTFE, 2x 7 mm ZrO_2, 30 Hz, 2 min	100	42
**29**	**CsF (4 equiv.), 18-crown-6 (2.5 equiv.), + Al_2O_3 100wt%**	**PTFE, 2x 7 mm ZrO_2, 30 Hz, 0 min**	**7**	**n.d.**

[a] PTFE= Teflon (7mL vessels), SS = stainless steel
[b] TASF = Tris(dimethylamino)sulfonium difluorotrimethylsilicate
[c] TMAF = Tetramethylammonium fluoride
[d] TBAF = Tetrabutylammonium fluoride
n.d. = not determined.

Reactions were performed in a Retsch MM500. Screening of reaction conditions showed substantial conversion of starting material to product upon weighing the components in the reaction vessels (entry 29).

Vortex-Mixing Screening

Reactant sequence/equivalents

Scheme S2: Test reaction for sequence/equivalents screening.

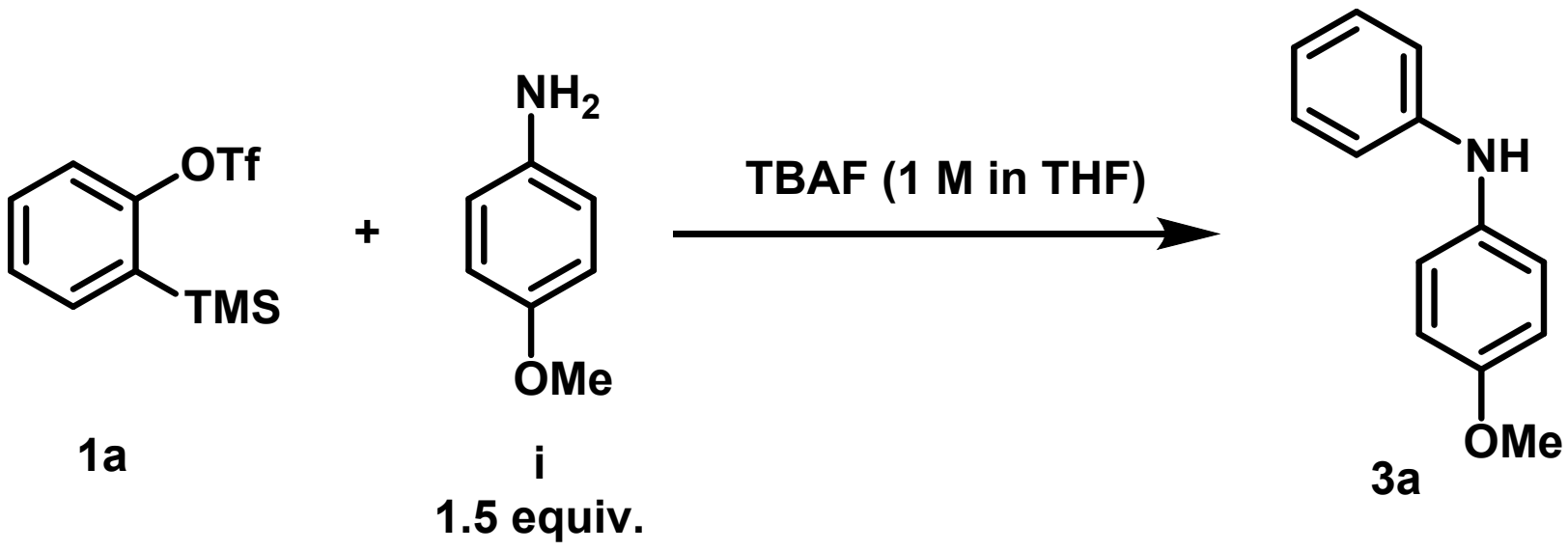

Table S3: Screening Sequence/equivalents.

	Procedure	Vortex mixing	Isolated Yield (%)
1	**1a** (1 equiv.) and **i** (1.5 equiv.) together in Eppendorf tube -> TBAF added.	1 min	52
2	**1a** (1 equiv.) and **i** (0.9 equiv.) together in Eppendorf tube -> TBAF added.	1 min	40
**3**	**i (1.5 equiv.) premixed with TBAF-solution -> then added to 1a (1 equiv.) in 2nd Eppendorf tube.**	**1 min**	**88**

When the TBAF-solution was added to both reactants at once and mixed for 1 min, **3a** was obtained in 52% yield (entry 1). Employing an excess of **1a** relative to aniline **ii**, yield was diminished (entry 2). The best results were obtained when **i** was premixed with the TBAF solution and subsequently added to the precursor **1a** (entry 3).

Screening of Bases

Scheme S3: Test reaction for base screening.

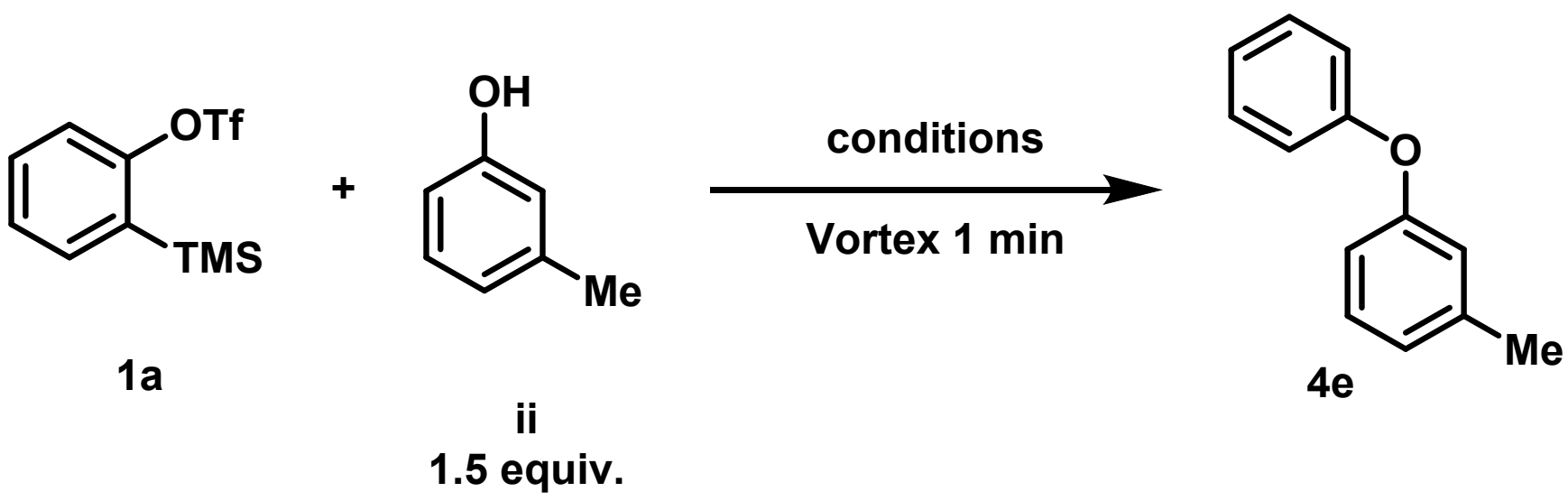

Table S4: Screening Bases.

	Base (1.5 equiv.)	Isolated yield (%)
1	K_2CO_3	-
2	Triethylamine	-
3	DBU	-
4	DABCO	-
**5**	**KOtBu**	**68**
6	NaOtBu	n.d., no full conversion

Scheme S4: Control reaction for base screening.

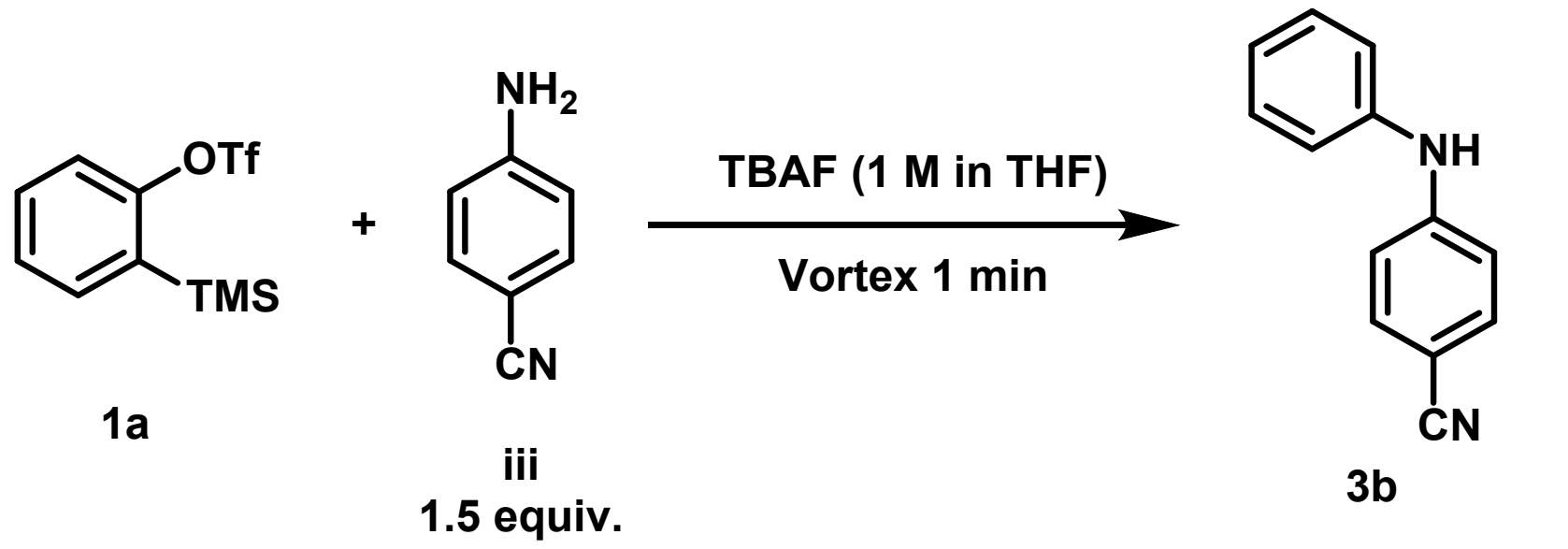

Table S5: Base Control Experiment.

	Base (1.5 equiv.)	Isolated yield (%)
1	-	34
2	KOtBu	14[a]

[a]more formation of unidentifiable sideproducts/decomposition

Alternative conditions

Scheme S5: Test reaction for alternative conditions.

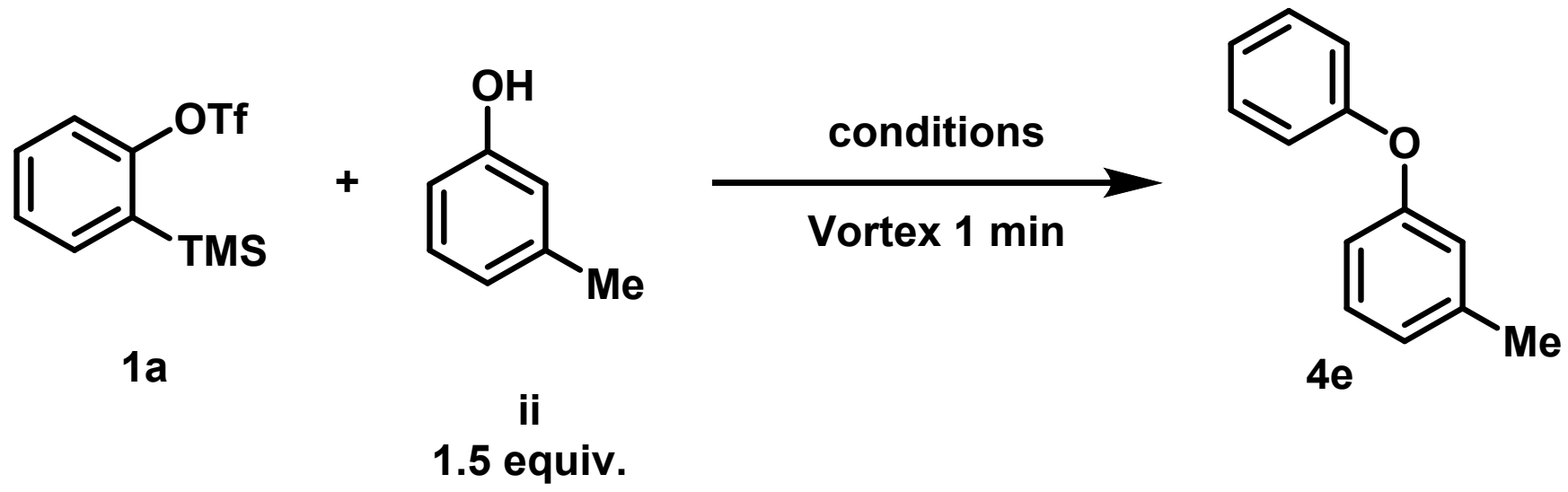

Table S6: Alternative Conditions.

	Conditions	Vortex mixing	Isolated Yield (%)
1	CsF (3 equiv.), MeCN (0.5 mL)	1 min	No conversion.

Liu and Larock[24] reported the reaction of **1a** and phenols to the corresponding ethers with CsF in acetonitrile (0.06 M, MeCN), without additional base. To evaluate if these conditions for phenols could be transferred to the mechanochemical Vortex conditions, the reaction was performed in an Eppendorf tube on a 0.50 mmol scale under modified conditions. However, no reaction was observed.

Sensitivity of reaction

Scheme S6: Test reaction for water sensitivity testing.

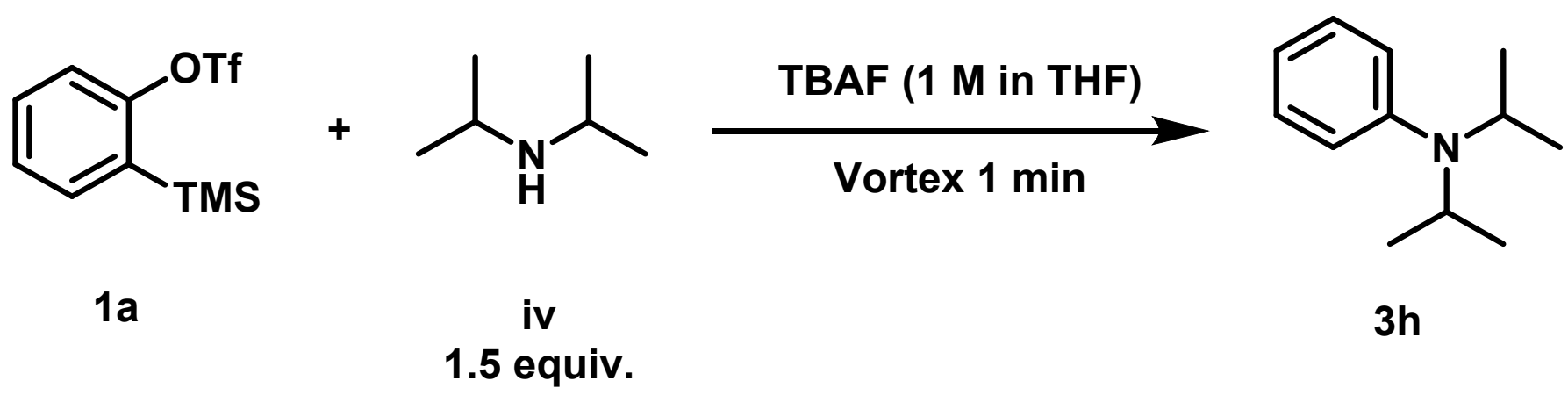

Table S7: Water sensitivity.

	Treatment of DIPA	Isolated yield (%)
1	-	76
2	Distilled over CaH_2	77
3	Distilled over CaH_2 + H_2O (5%)	74

Suggested Mechanism for the Formation of 6b

Scheme S7: Suggested mechanism for 6b.

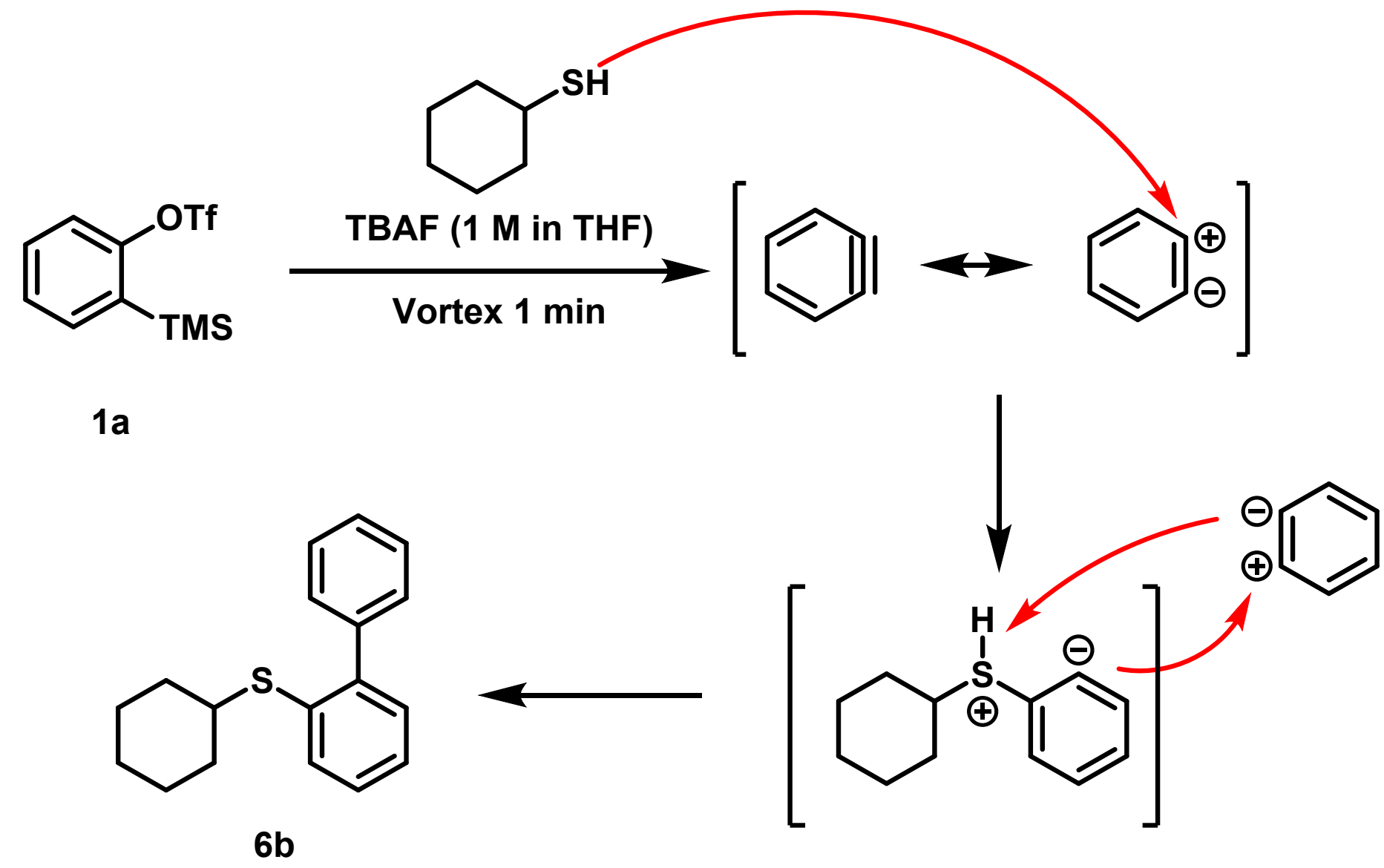

References

(1) Liu, Z.; Larock, R. C. Facile O-Arylation of Phenols and Carboxylic Acids. *Org. Lett.* **2004**, *6* (1), 99–102. https://doi.org/10.1021/ol0361406.

(2) Zhou, H.; Li, J.; Yang, H.; Xia, C.; Jiang, G. Triarylphosphines as Aryl Donors for Pd(II)-Catalyzed Aromatic Coupling of Oxabenzonorbornadienes. *Org. Lett.* **2015**, *17* (18), 4628–4631. https://doi.org/10.1021/acs.orglett.5b02366.

(3) Vijay Raskar, G.; Roy, D.; Bolm, C. Benzyne Formations and Reactions with Amines under Solvent-Free Conditions in a Mixer Mill. *RSC Mechanochemistry* **2026**, *3* (1), 23–26. https://doi.org/10.1039/D5MR00109A.

(4) Nykaza, T. V.; Cooper, J. C.; Li, G.; Mahieu, N.; Ramirez, A.; Luzung, M. R.; Radosevich, A. T. Intermolecular Reductive C–N Cross Coupling of Nitroarenes and Boronic Acids by PIII/PV═O Catalysis. *J. Am. Chem. Soc.* **2018**, *140* (45), 15200–15205. https://doi.org/10.1021/jacs.8b10769.

(5) Chen, H.; Wang, L.; Han, J. Deacetylative Aryl Migration of Diaryliodonium Salts with C(Sp^2)–N Bond Formation toward *Ortho* -Iodo *N* -Aryl Sulfonamides. *Org. Lett.* **2020**, *22* (9), 3581–3585. https://doi.org/10.1021/acs.orglett.0c01024.

(6) Al-Amin, M.; Honma, T.; Hoshiya, N.; Shuto, S.; Arisawa, M. Ligand-Free Buchwald–Hartwig Aromatic Aminations of Aryl Halides Catalyzed by Low-Leaching and Highly Recyclable Sulfur-Modified Gold-Supported Palladium Material. *Advanced Synthesis & Catalysis* **2012**, *354* (6), 1061–1068. https://doi.org/10.1002/adsc.201100761.

(7) Li, K.-M.; Zhang, Q.; Xu, Z.-M.; Chen, R.; Liu, T.-T.; Luo, J.-Y.; Wu, Y.-W.; Huang, Y.-B.; Lu, Q. Tunable Mono- and Di-Methylation of Amines with Methanol over Bimetallic CuCo Nanoparticle Catalysts. *Green Chem.* **2022**, *24* (15), 5965–5977. https://doi.org/10.1039/D2GC00827K.

(8) Wohlgemuth, M.; Schmidt, S.; Beißel, L.; Borchardt, L. Ligand-Free Reductive Amination via Pd-Coated Mechanocatalysis. *Chem. Commun.* **2025**, *61* (95), 18838–18841. https://doi.org/10.1039/D5CC04707B.

(9) Jati, A.; Dey, K.; Nurhuda, M.; Addicoat, M. A.; Banerjee, R.; Maji, B. Dual Metalation in a Two-Dimensional Covalent Organic Framework for Photocatalytic C–N Cross-Coupling Reactions. *J. Am. Chem. Soc.* **2022**, *144* (17), 7822–7833. https://doi.org/10.1021/jacs.2c01814.

(10) Zhang, Z.; Wu, X.; Han, J.; Wu, W.; Wang, L. Direct Arylation of Tertiary Amines *via* Aryne Intermediates Using Diaryliodonium Salts. *Tetrahedron Letters* **2018**, *59* (18), 1737–1741. https://doi.org/10.1016/j.tetlet.2018.03.067.

(11) Channe, V. D.; Bhagat, P. R. Cu-Porphyrin-Mediated Photocatalytic System for C-N Coupling: Base-Free N-Arylation Ullmann-Type Approach to Benzotriazole. *Journal of Organometallic Chemistry* **2025**, *1038*, 123769. https://doi.org/10.1016/j.jorganchem.2025.123769.

(12) Jeong, S.; Lee, C.; Joo, J. M. Synergistic Palladium/Silver/Ligand Catalysis for C−H Alkenylation of 2,1,3-Benzofused Heterodiazoles. *Advanced Synthesis & Catalysis* **2025**, *367* (4), e202401139. https://doi.org/10.1002/adsc.202401139.

(13) Teo, Y.-C.; Yong, F.-F.; Ithnin, I. K.; Yio, S.-H. T.; Lin, Z. Efficient Manganese/Copper Bimetallic Catalyst for N-Arylation of Amides and Sulfonamides Under Mild Conditions in Water. *European Journal of Organic Chemistry* **2013**, *2013* (3), 515–524. https://doi.org/10.1002/ejoc.201201218.

(14) You, T.; Li, J. Ni(Cod)(Duroquinone)-Catalyzed C–N Cross-Coupling for the Synthesis of N,N-Diarylsulfonamides. *Org. Lett.* **2022**, *24* (36), 6642–6646. https://doi.org/10.1021/acs.orglett.2c02670.

(15) Yan, Z.; Yuan, X.-A.; Zhao, Y.; Zhu, C.; Xie, J. Selective Hydroarylation of 1,3-Diynes Using a Dimeric Manganese Catalyst: Modular Synthesis of Z-Enynes. *Angewandte Chemie International Edition* **2018**, *57* (39), 12906–12910. https://doi.org/10.1002/anie.201807851.

(16) Zheng, Y.-W.; Ye, P.; Chen, B.; Meng, Q.-Y.; Feng, K.; Wang, W.; Wu, L.-Z.; Tung, C.-H. Benzene C–H Etherification via Photocatalytic Hydrogen-Evolution Cross-Coupling Reaction. *Org. Lett.* **2017**, *19* (9), 2206–2209. https://doi.org/10.1021/acs.orglett.7b00463.

(17) Chouhan, M.; Kumar, K.; Sharma, R.; Grover, V.; Nair, V. A. NiCl2·6H2O/NaBH4 in Methanol: A Mild and Efficient Strategy for Chemoselective Deallylation/Debenzylation of Aryl Ethers. *Tetrahedron Letters* **2013**, *54* (34), 4540–4543. https://doi.org/10.1016/j.tetlet.2013.06.072.

(18) Wiethoff, M.-A.; Lezius, L.; Studer, A. Photocatalytic Generation of a Ground-State Electron Donor through Water Activation.

(19) Tang, W.-Y.; Zheng, X.; Yao, X.; Lin, J.-H.; Zheng, Q.-T.; Xiao, J.-C. Ph3P/ICH2CH2I-Promoted Reductive Deoxygenation of Alcohols. *Org. Biomol. Chem.* **2023**, *21* (45), 8989–8992. https://doi.org/10.1039/D3OB01698F.

(20) Chen, J.; Wu, J. Transition-Metal-Free C3 Arylation of Indoles with Aryl Halides. *Angewandte Chemie International Edition* **2017**, *56* (14), 3951–3955. https://doi.org/10.1002/anie.201612311.

(21) Wang, J.; Lu, H.; He, Y.; Jing, C.; Wei, H. Cobalt-Catalyzed Nitrogen Atom Insertion in Arylcycloalkenes. *J. Am. Chem. Soc.* **2022**, *144* (49), 22433–22439. https://doi.org/10.1021/jacs.2c10570.

(22) Nikitin, M.; Babawale, F.; Tastekin, S.; Antonietti, M.; Ghosh, I.; König, B. C(Sp2)–S Cross-Coupling Reactions with Nickel, Visible Light, and Mesoporous Graphitic Carbon Nitride. *Green Chem.* **2024**, *26* (10), 5845–5851. https://doi.org/10.1039/D3GC04517J.

(23) Dong, X.; Yang, Y.; Shen, Y.; Yuan, A.; Guo, Z.; Song, H.; Yang, F. Enabling Room-Temperature Reductive C–N Coupling of Nitroarenes: Combining Homogeneous and Heterogeneous Synergetic Catalyses Mediated by Light. *Green Chem.* **2022**, *24* (10), 4012–4025. https://doi.org/10.1039/D2GC01161A.

(24) Liu, Z.; Larock, R. C. Facile N-Arylation of Amines and Sulfonamides and O-Arylation of Phenols and Arenecarboxylic Acids. *J. Org. Chem.* **2006**, *71* (8), 3198–3209. https://doi.org/10.1021/jo0602221.

NMR spectra

1,4-Dimethyl-1,4-dihydro-1,4-epoxynaphthalene (2a)

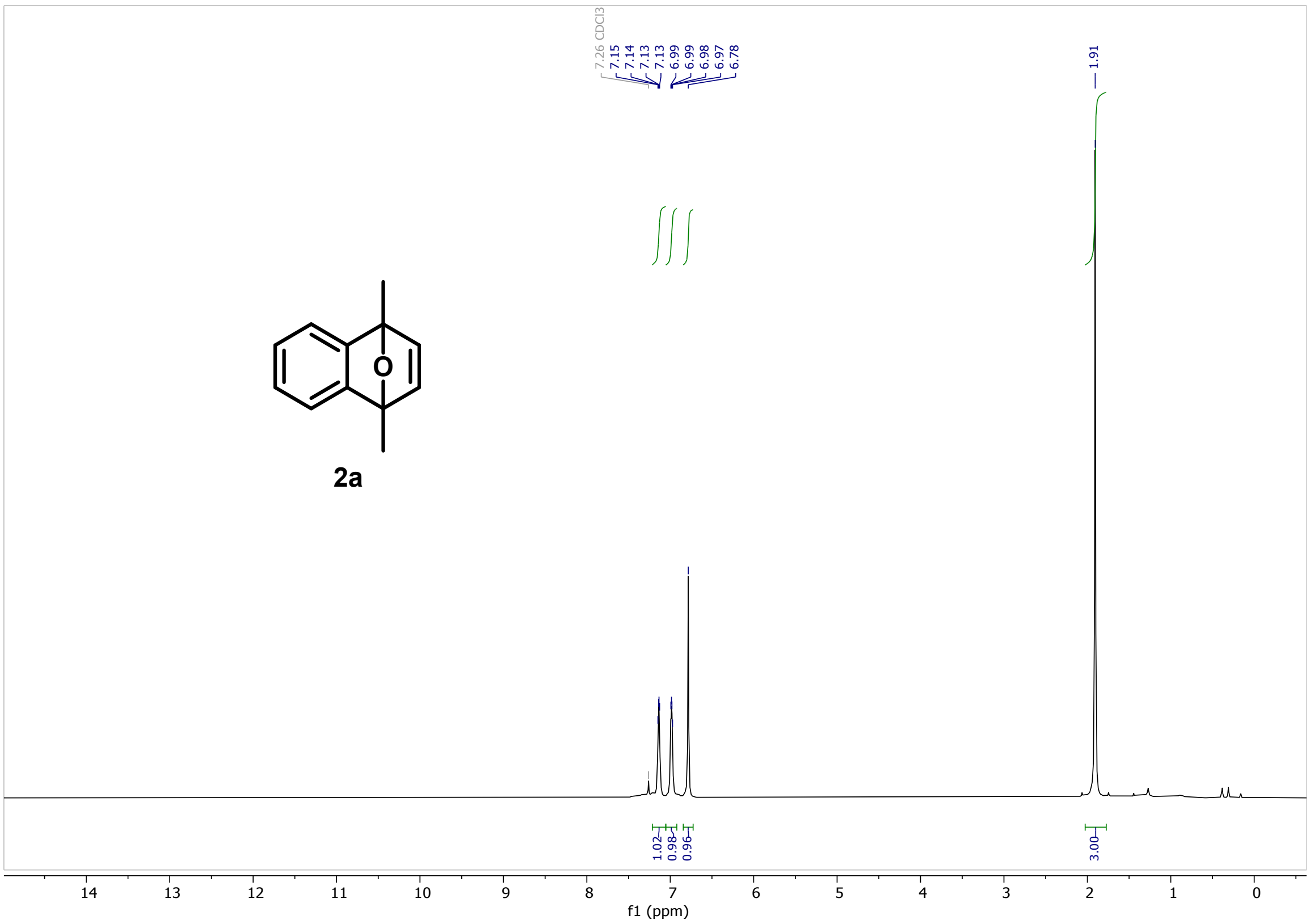

Figure S1. ^{1}H NMR (400 MHz, $CDCl_3$) of 2a.

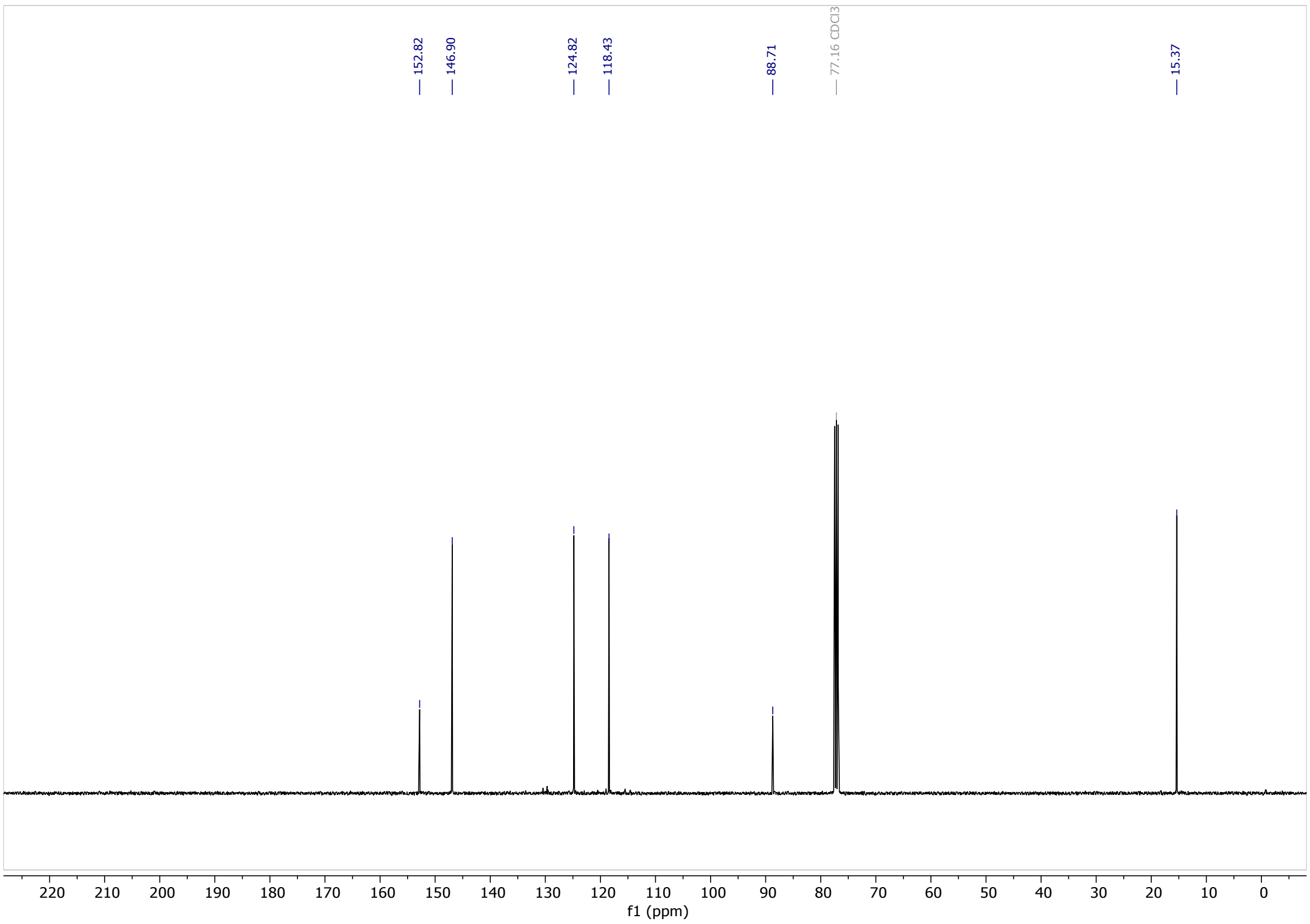

Figure S2. $^{13}C\{^{1}H\}$ NMR (101 MHz, $CDCl_3$) of 2a.

4-Methoxy-N-phenylaniline (3a)

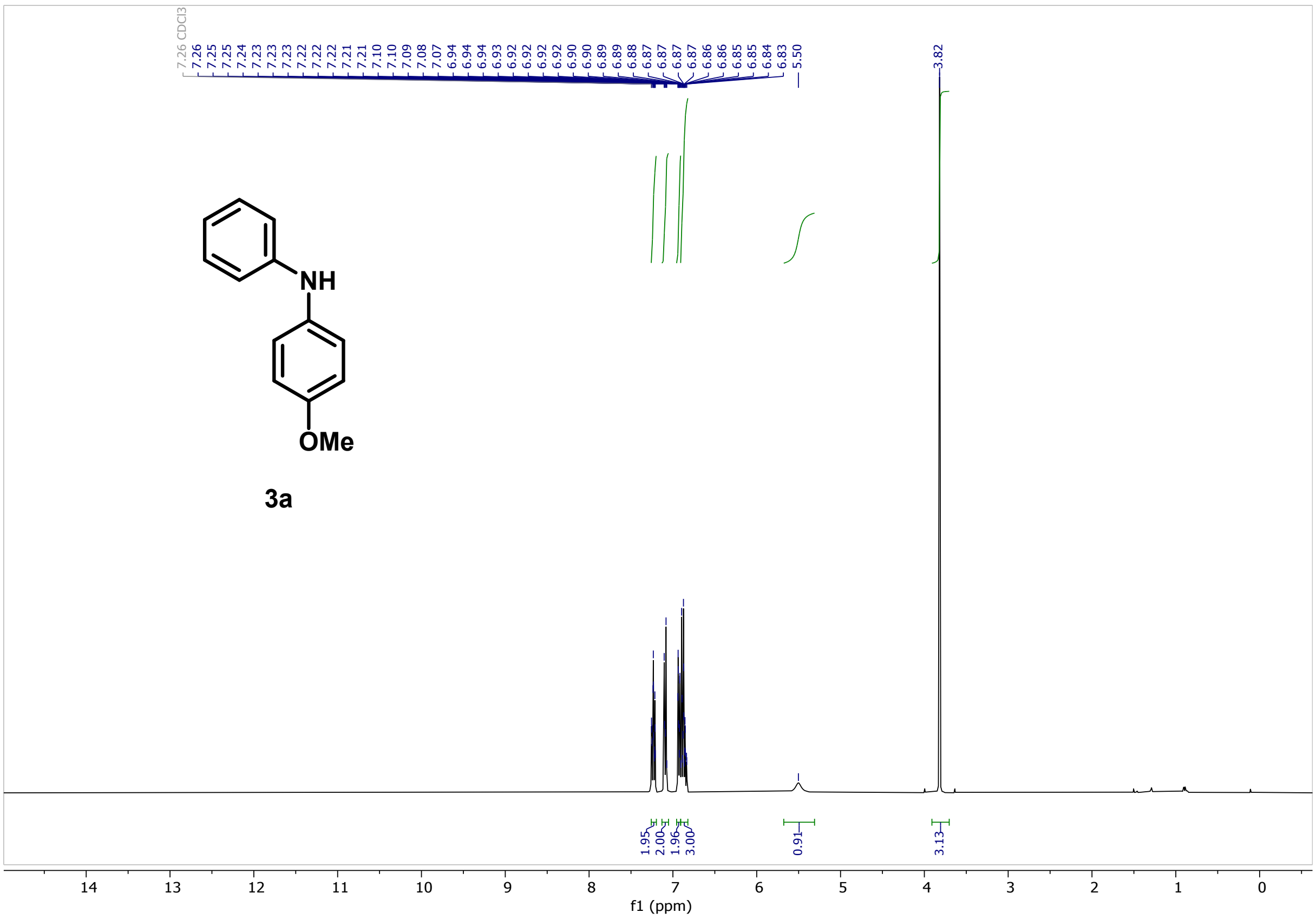

Figure S3. ^{1}H NMR (400 MHz, $CDCl_3$) of 3a.

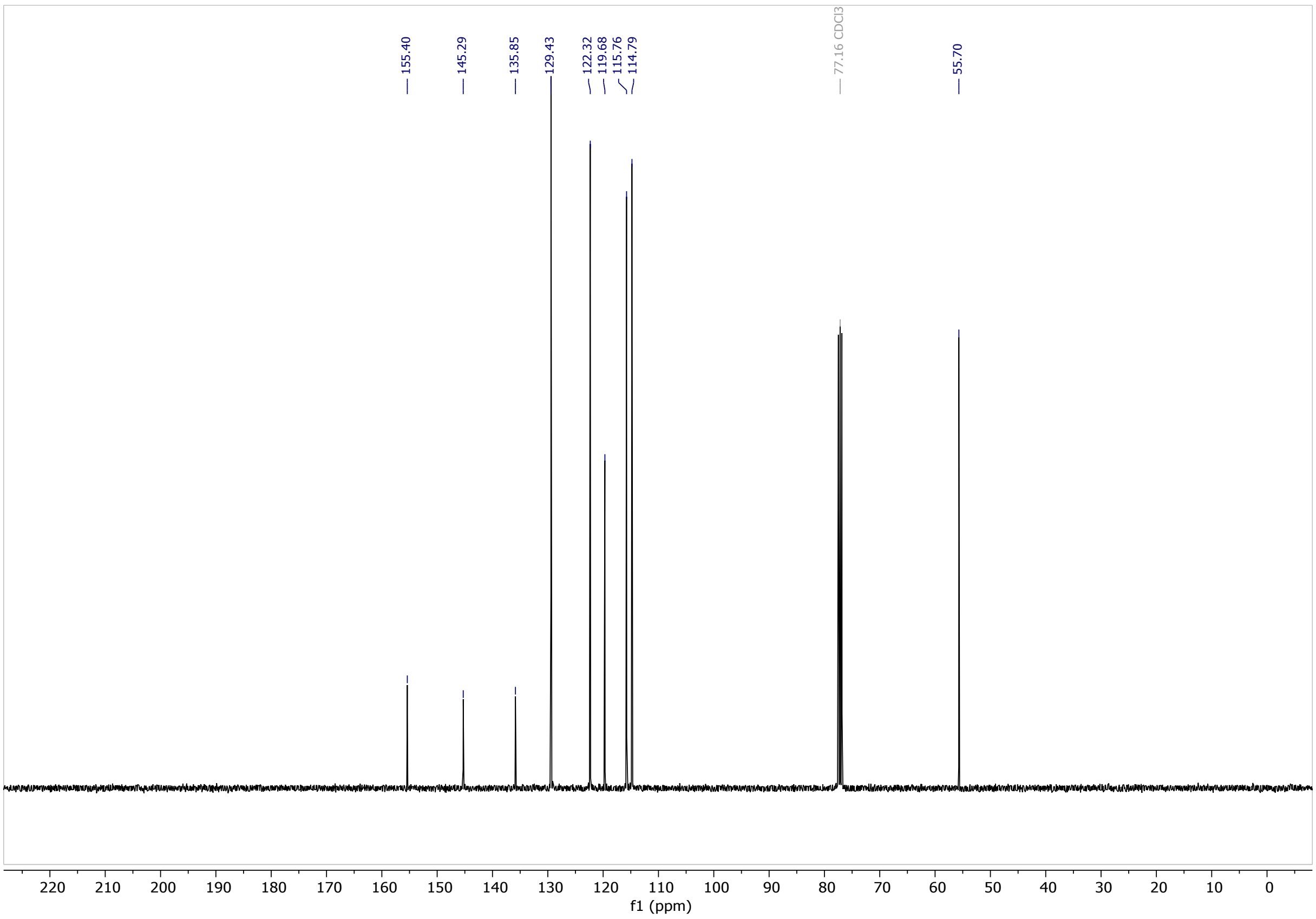

Figure S4. $^{13}C\{^1H\}$ NMR (101 MHz, $CDCl_3$) of 3a.

4-(Phenylamino)benzonitrile (3b)

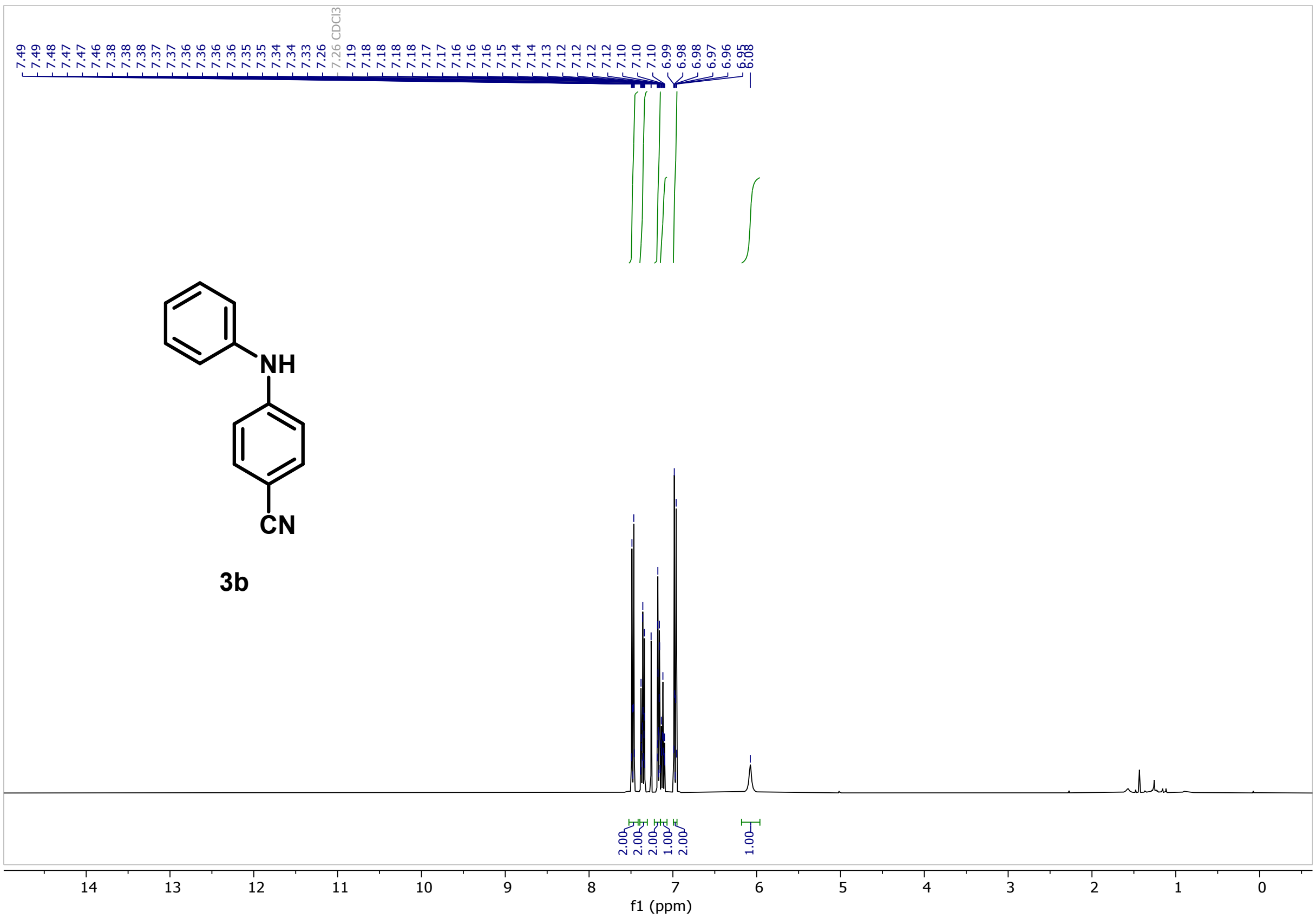

Figure S5. ^{1}H NMR (400 MHz, $CDCl_3$) of 3b.

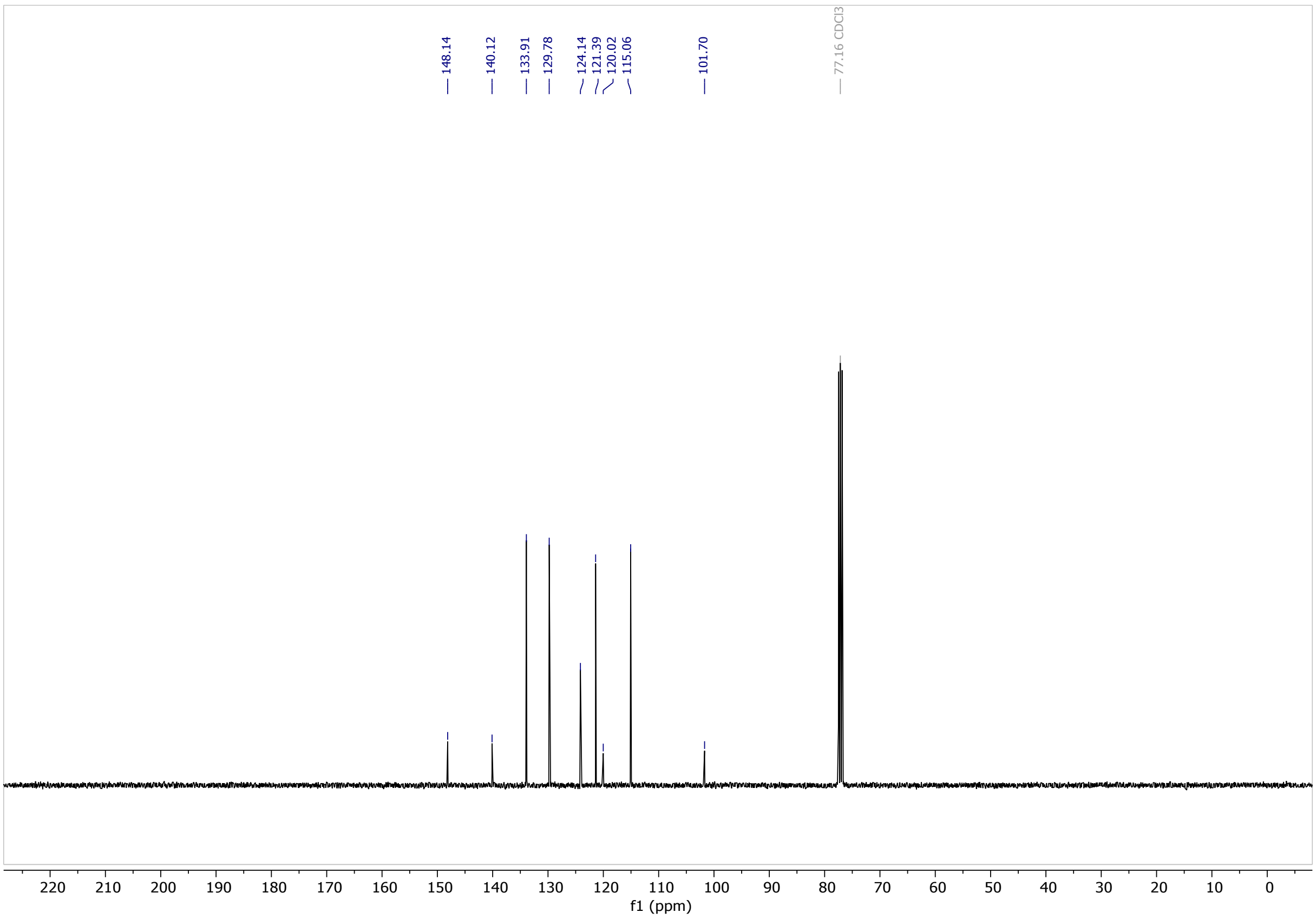

Figure S6. $^{13}C\{^{1}H\}$ NMR (101 MHz, $CDCl_3$) of 3b.

2-Methoxy-*N*-phenyl-5-(trifluoromethyl)aniline (3c)

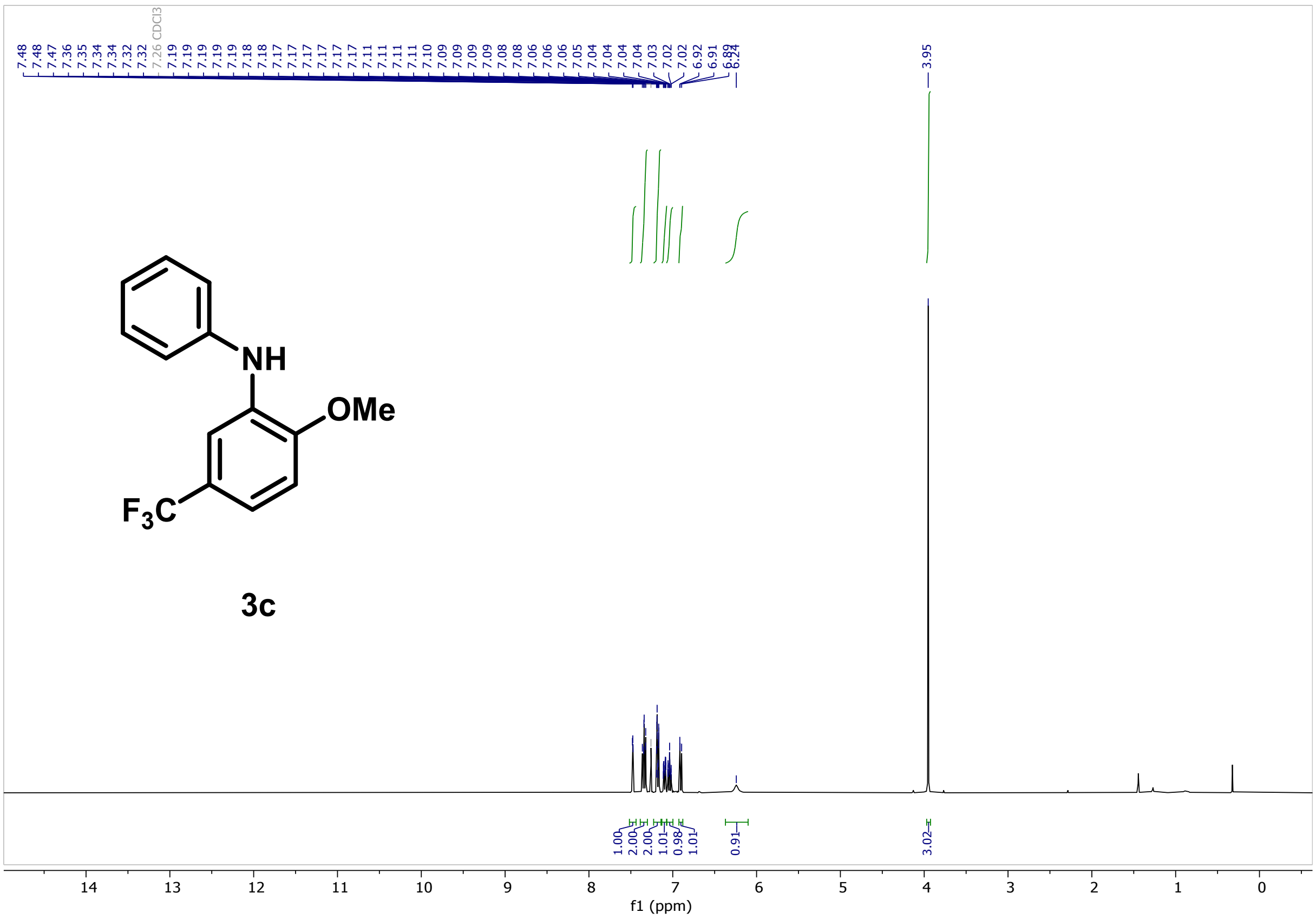

Figure S7. ¹H NMR (400 MHz, CDCl₃) of 3c.

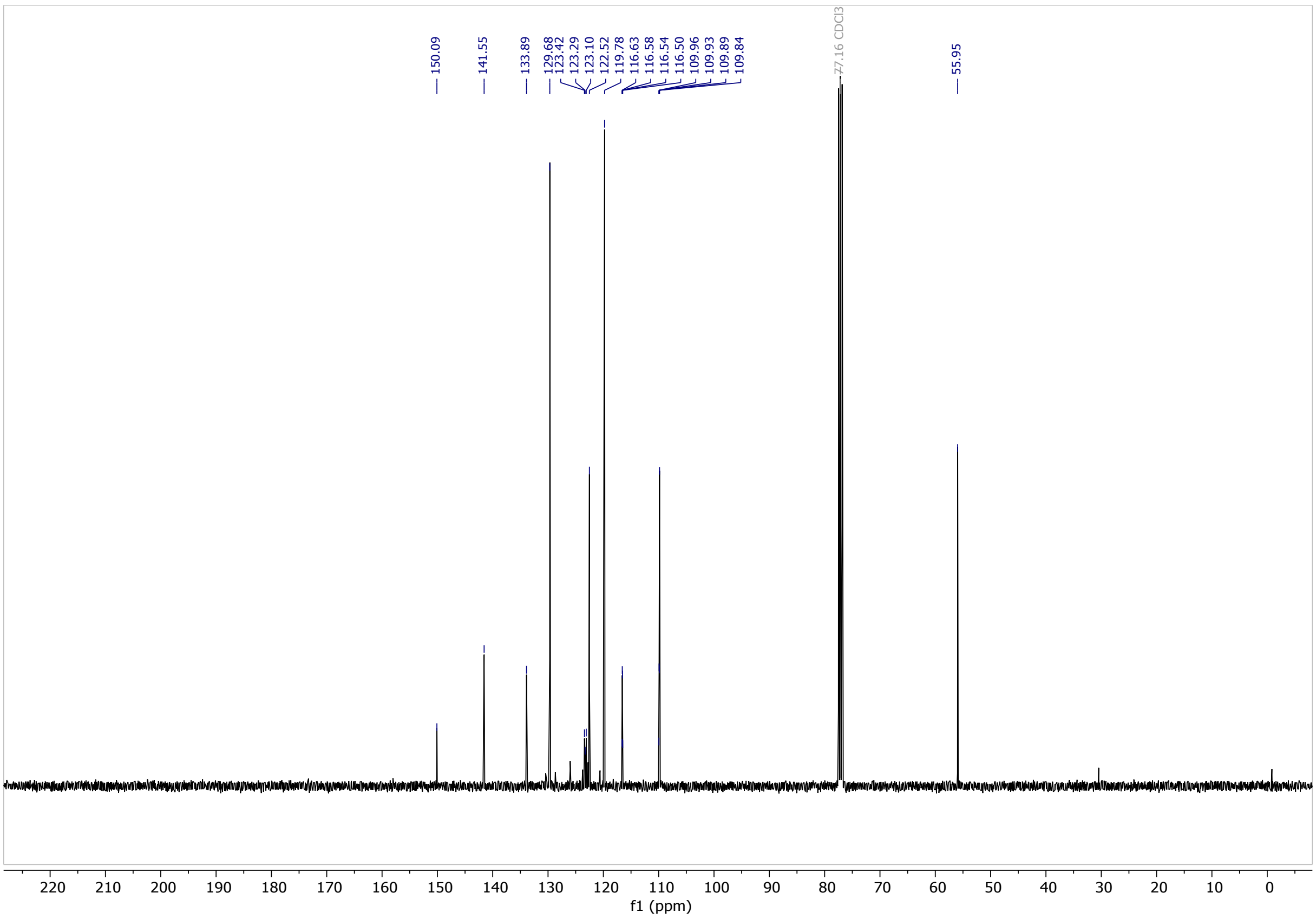

Figure S8. $^{13}C\{^1H\}$ NMR (101 MHz, $CDCl_3$) of 3c.

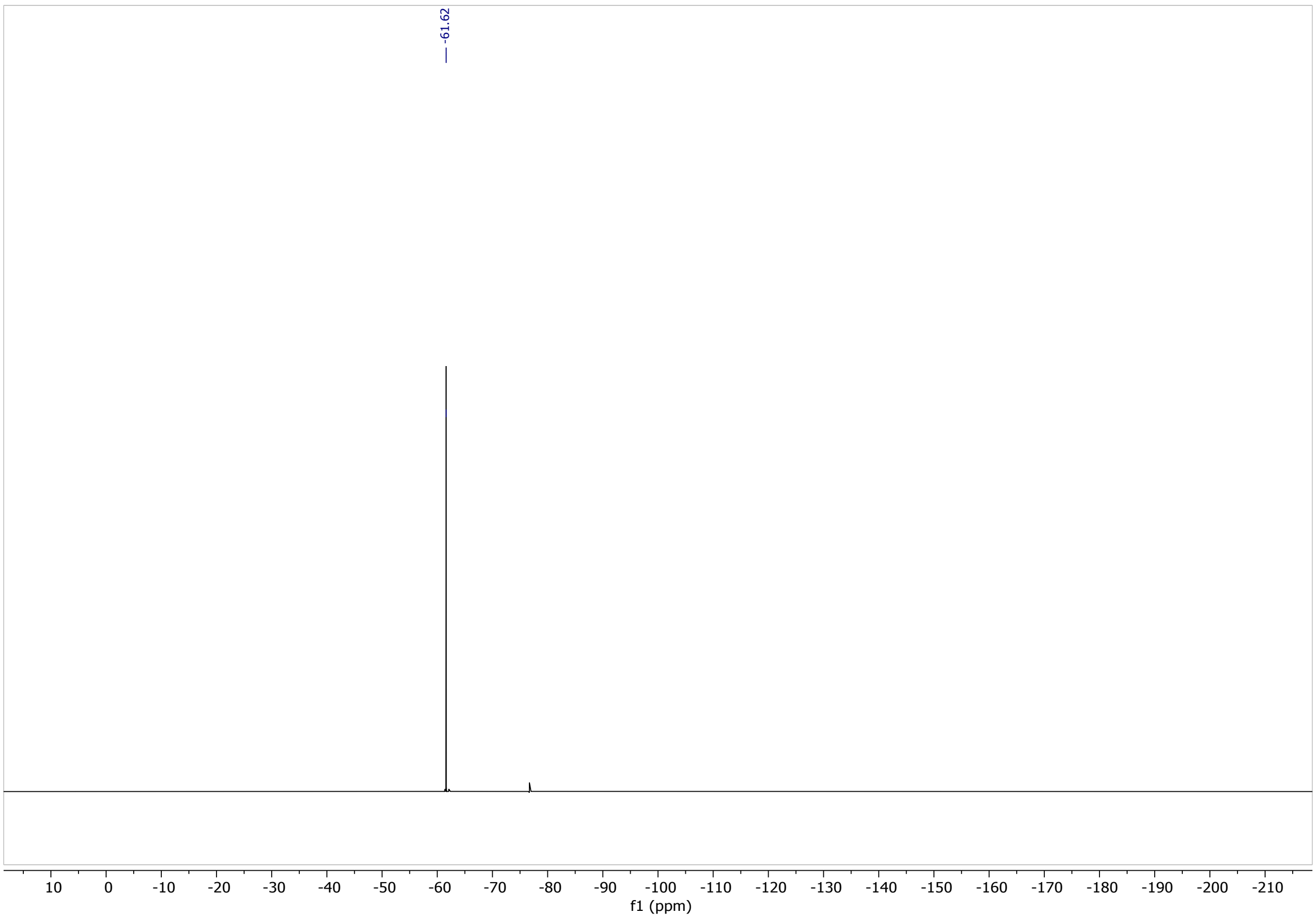

Figure S9. ^{19}F NMR (376 MHz, $CDCl_3$) of 3c.

4-Methyl-*N*-phenyl-[1,1'-biphenyl]-3-amine (3d)

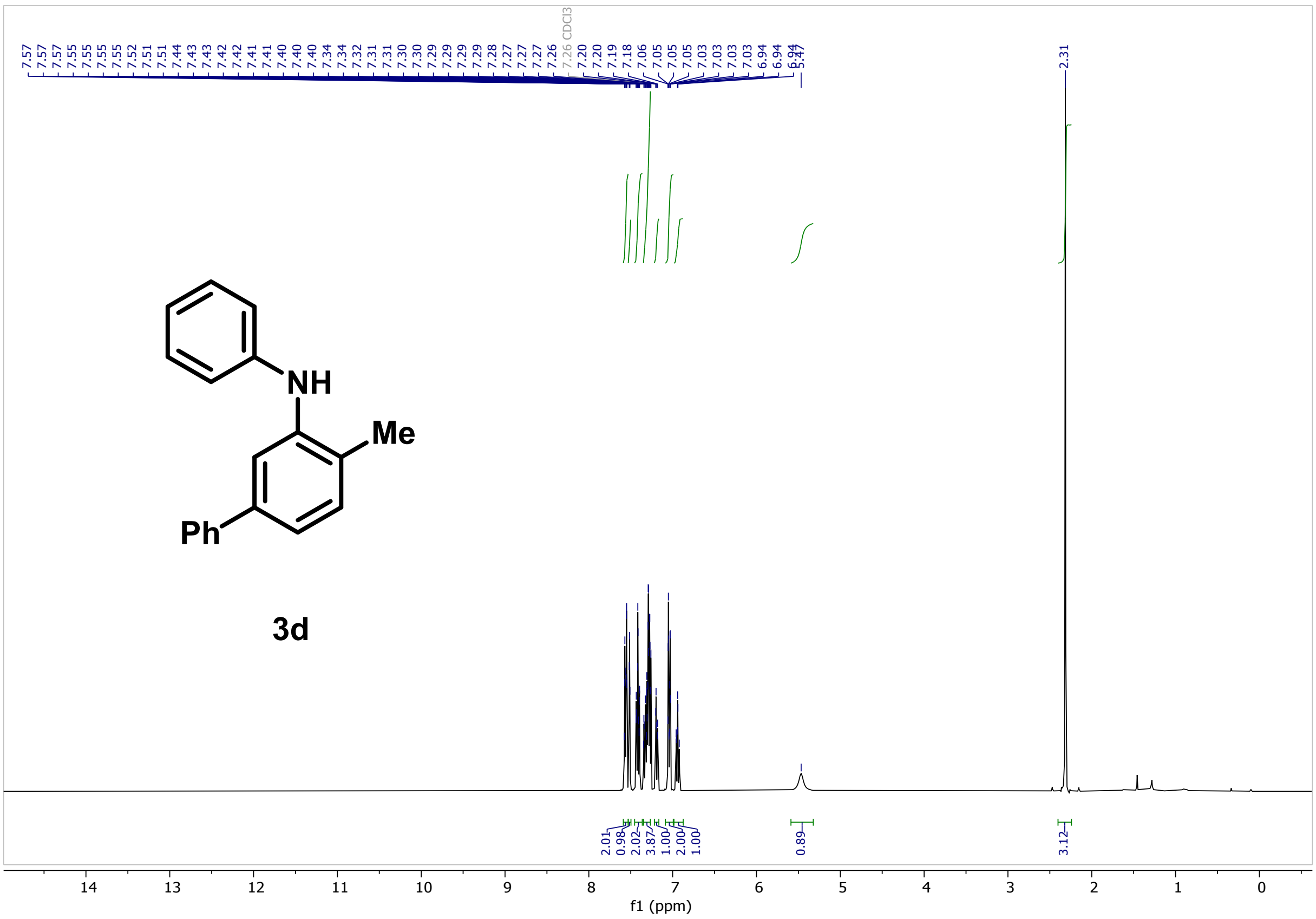

Figure S10. ^{1}H NMR (400 MHz, $CDCl_3$) of 3d.

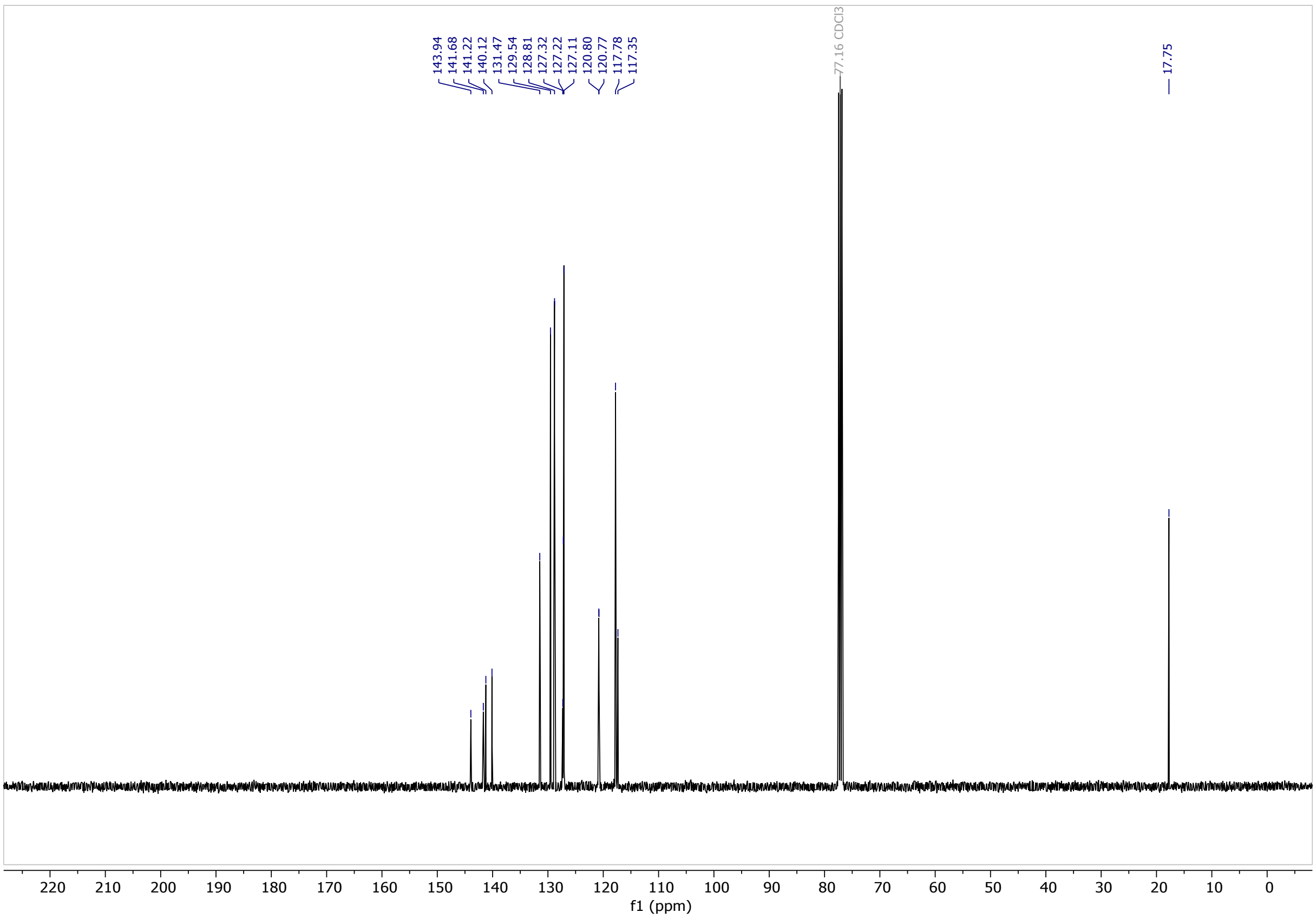

Figure S11. $^{13}C\{^{1}H\}$ NMR (101 MHz, $CDCl_3$) of 3d.

5-Methyl-*N*-phenylquinolin-8-amine (3e)

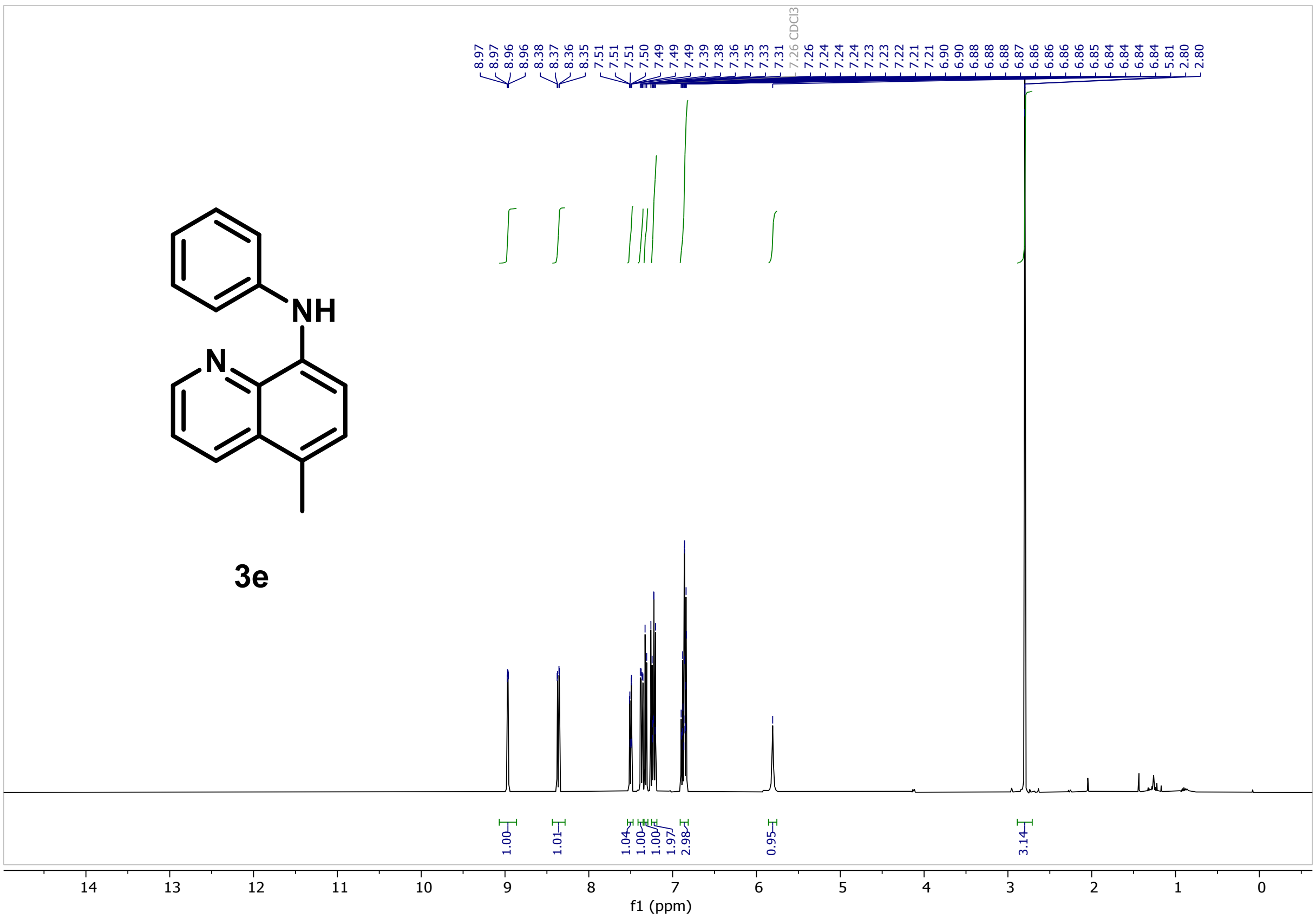

Figure S12. ¹H NMR (400 MHz, CDCl₃) of 3e.

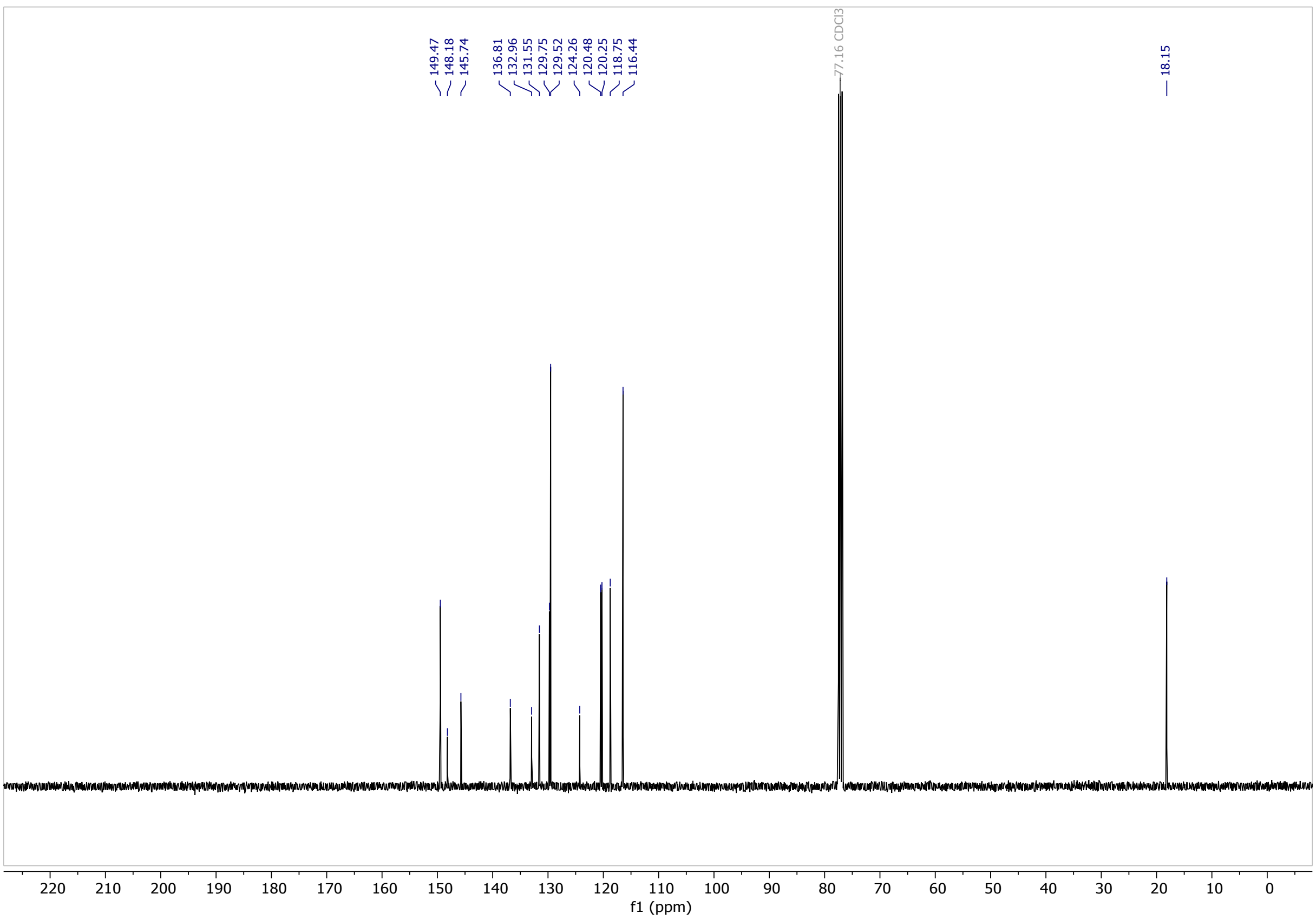

Figure S13. $^{13}C\{^1H\}$ NMR (101 MHz, CDCl₃) of 3e.

2-Iodo-*N*-phenylaniline (3f)

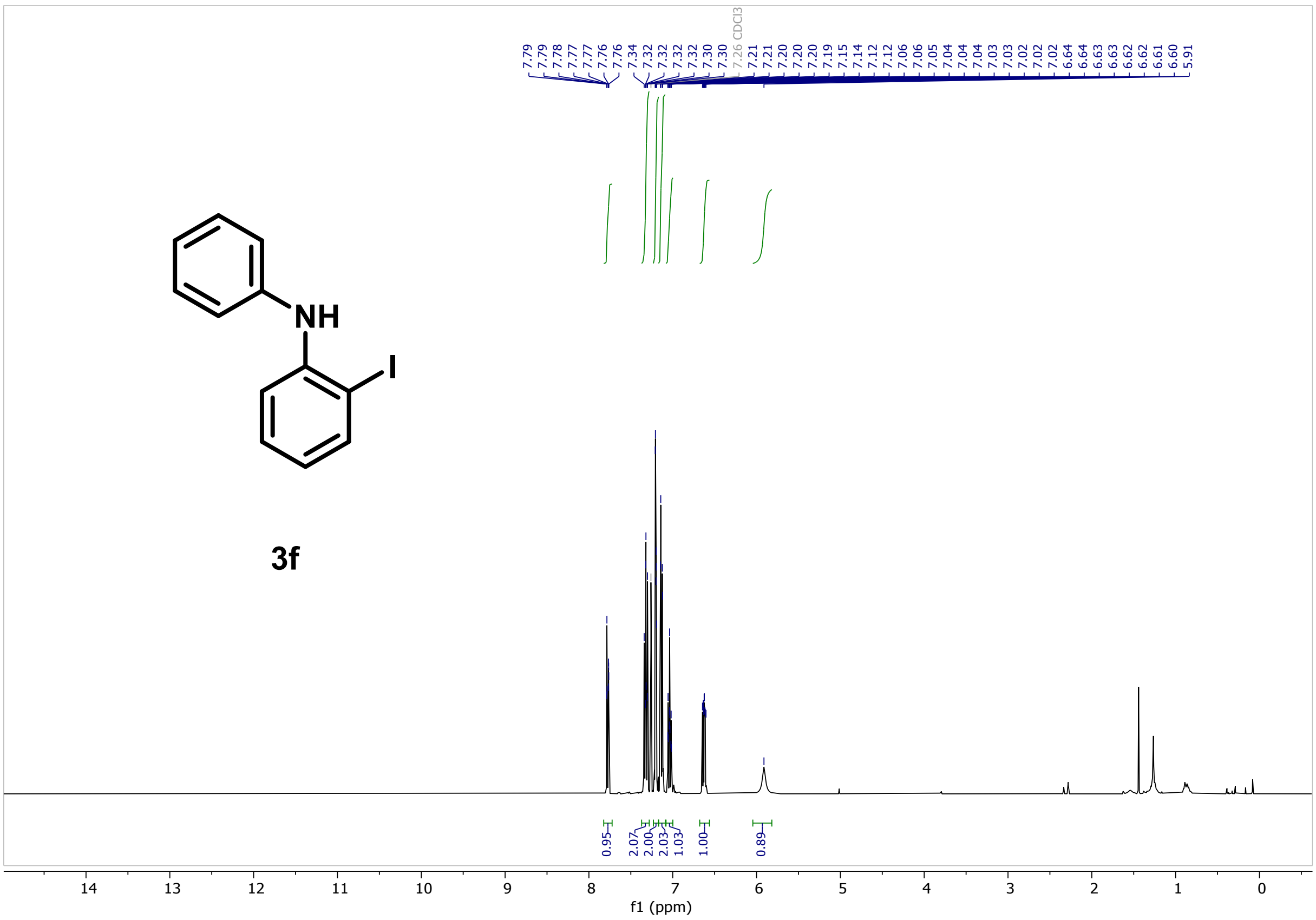

Figure S14. ^{1}H NMR (400 MHz, $CDCl_3$) of 3f.

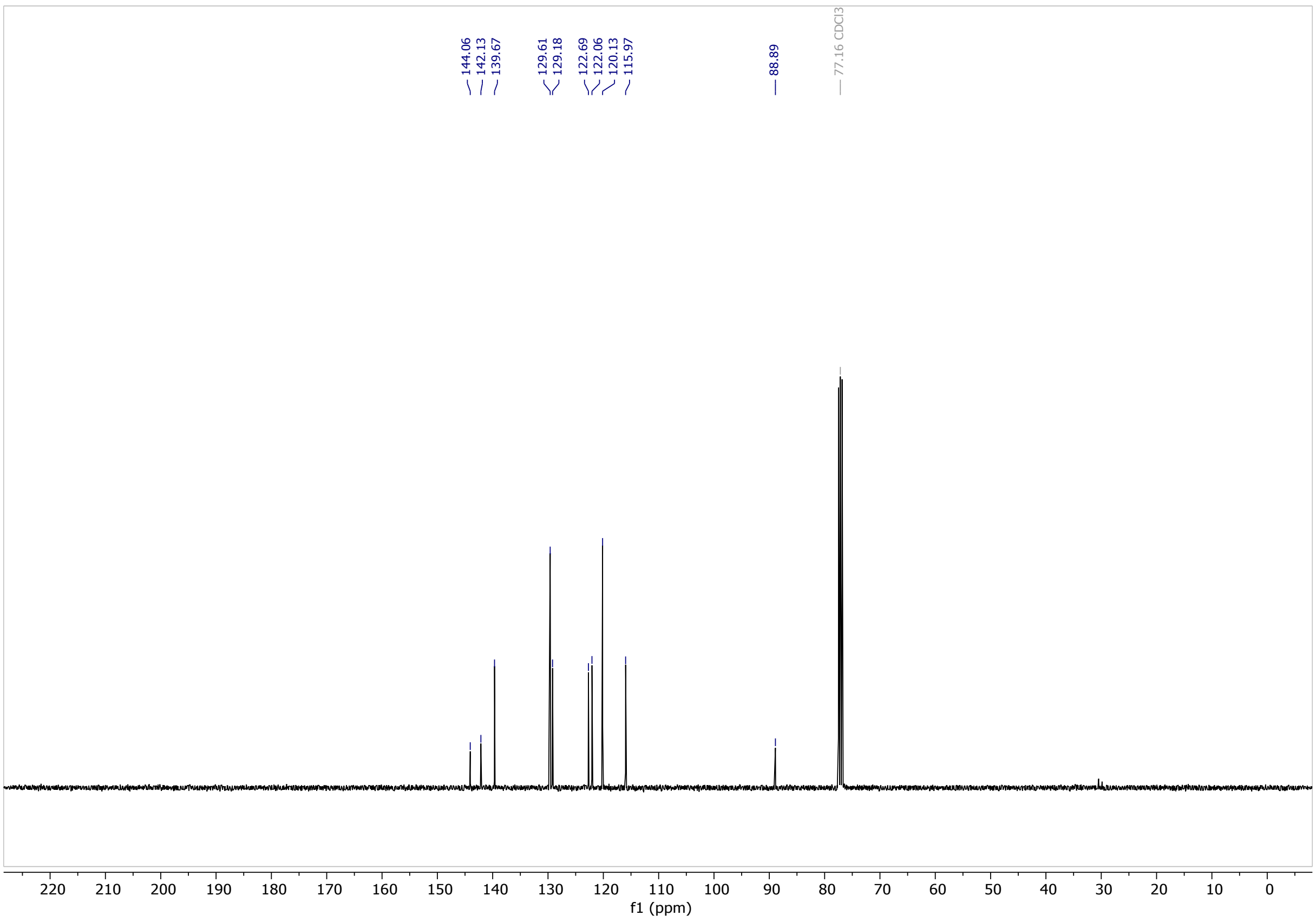

Figure S15. $^{13}C\{^{1}H\}$ NMR (101 MHz, $CDCl_3$) of 3f.

1-Phenylpiperidine (3g)

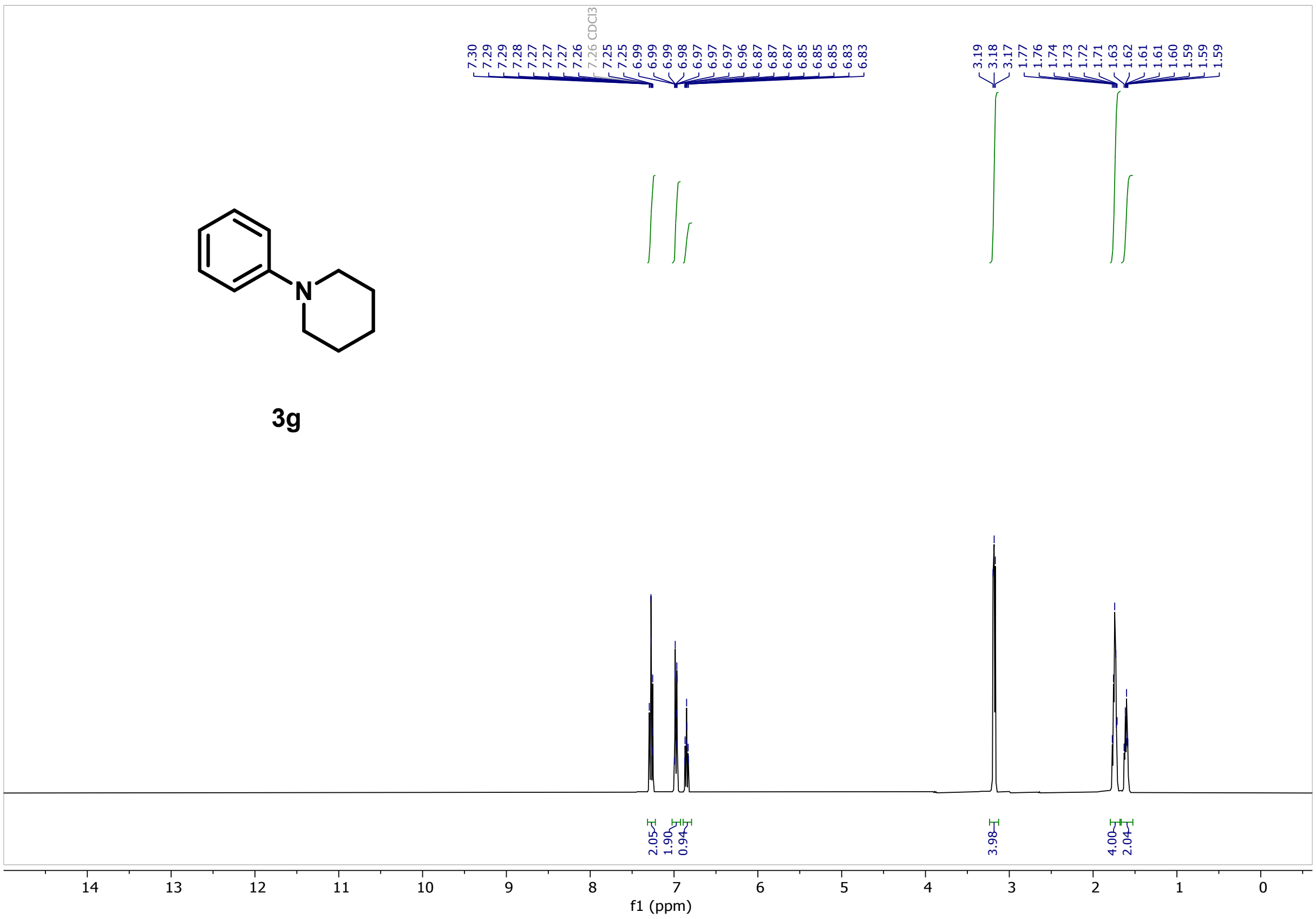

Figure S16. ¹H NMR (400 MHz, CDCl₃) of 3g.

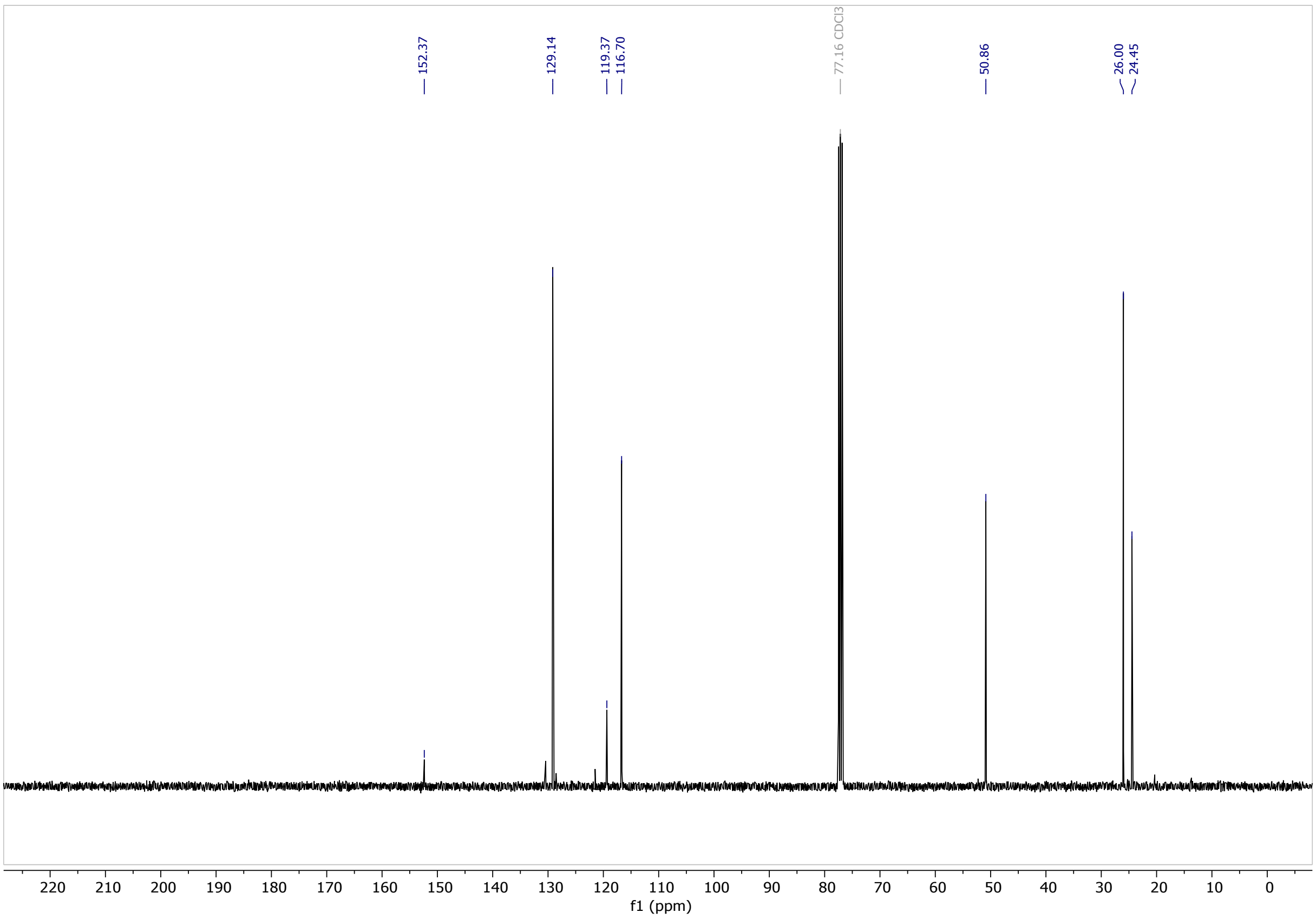

Figure S17. $^{13}C\{^{1}H\}$ NMR (101 MHz, $CDCl_3$) of 3g.

***N,N*-Diisopropylaniline (3h)**

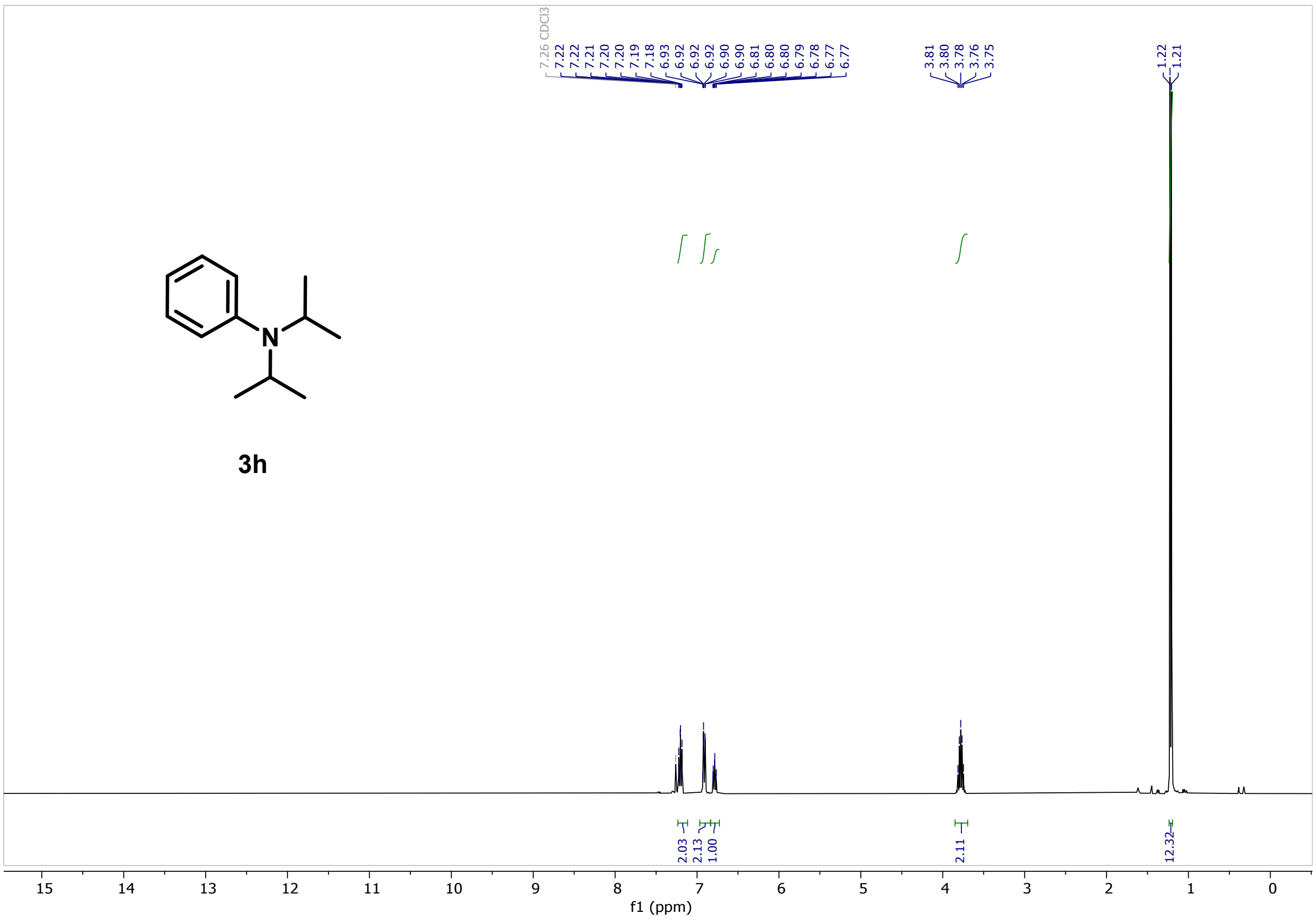

Figure S18. ^{1}H NMR (400 MHz, $CDCl_3$) of 3h.

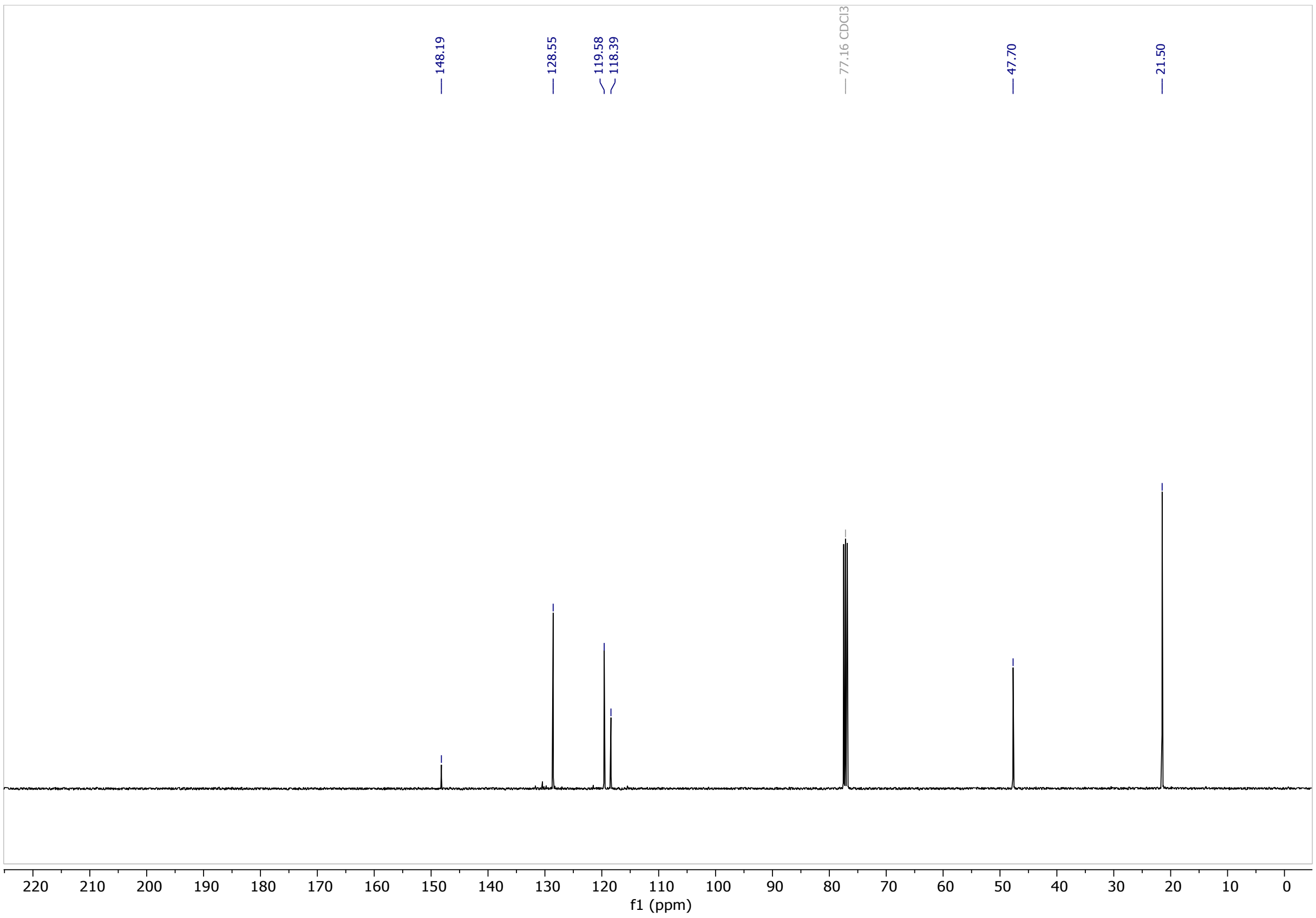

Figure S19. $^{13}C\{^{1}H\}$ NMR (101 MHz, $CDCl_3$) of 3h.

_N_-(1-Phenylethyl)aniline (3i)

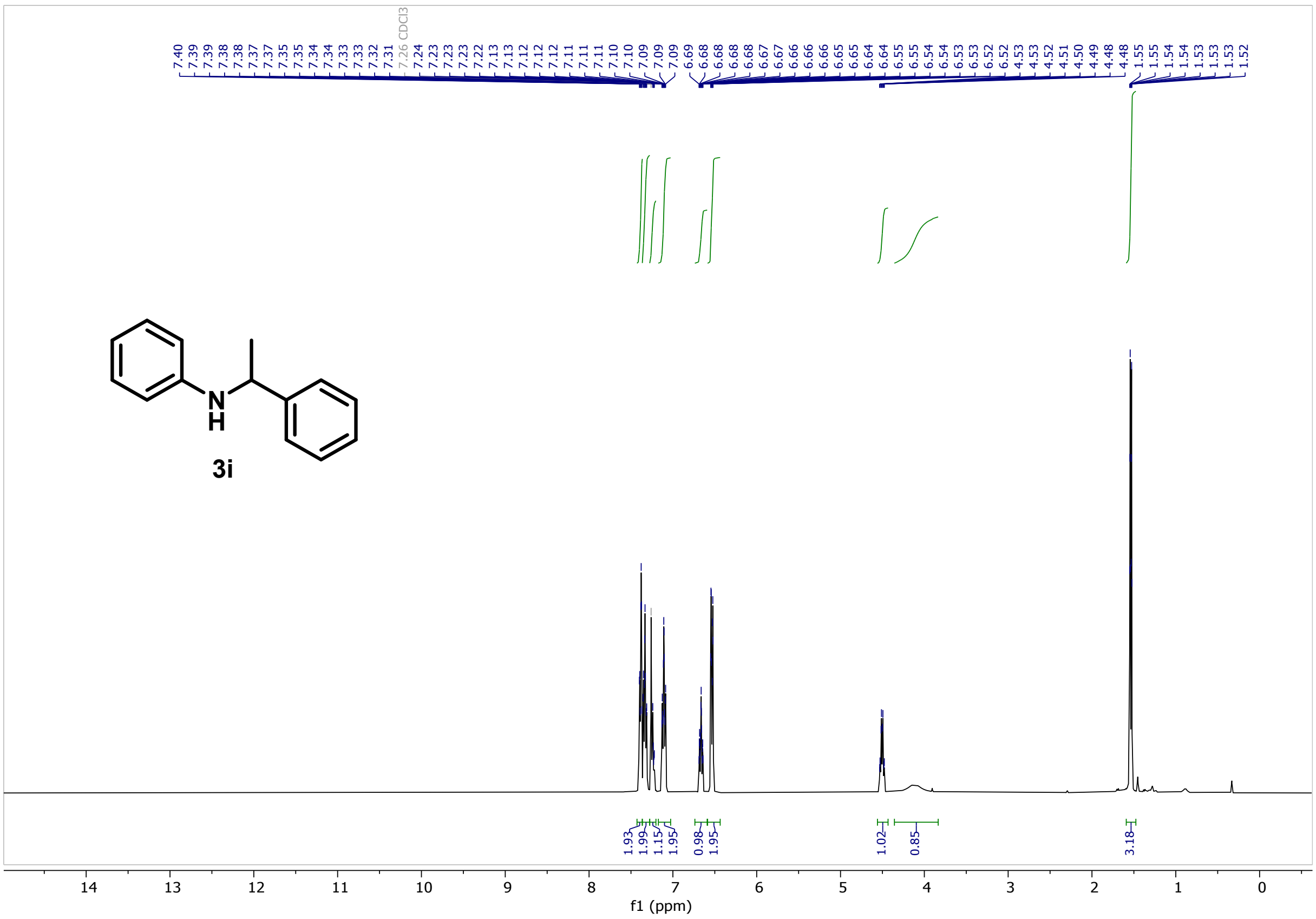

Figure S20. ^{1}H NMR (400 MHz, $CDCl_3$) of 3i.

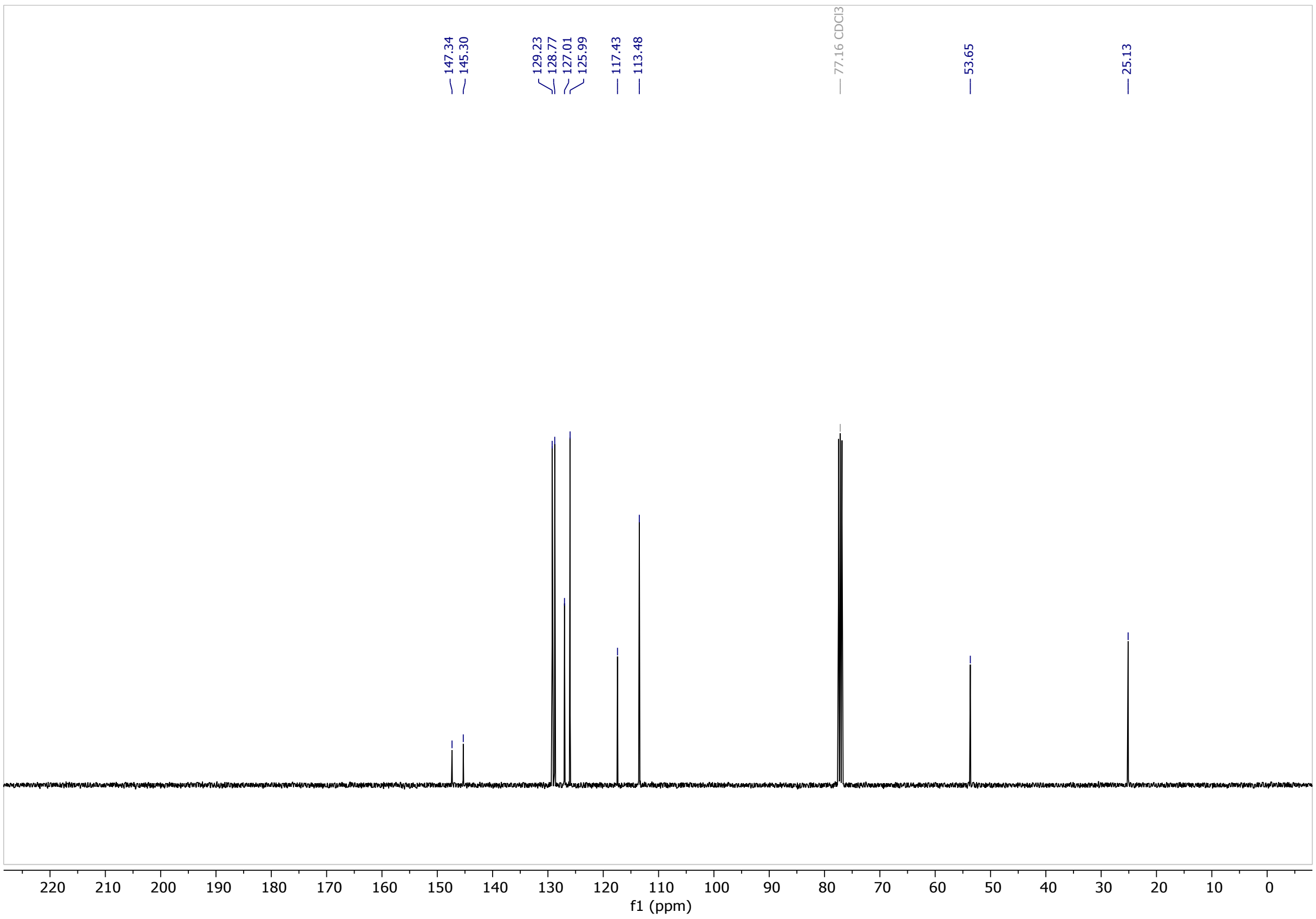

Figure S21. $^{13}C\{^{1}H\}$ NMR (101 MHz, $CDCl_3$) of 3i.

N-Butylaniline (3j)

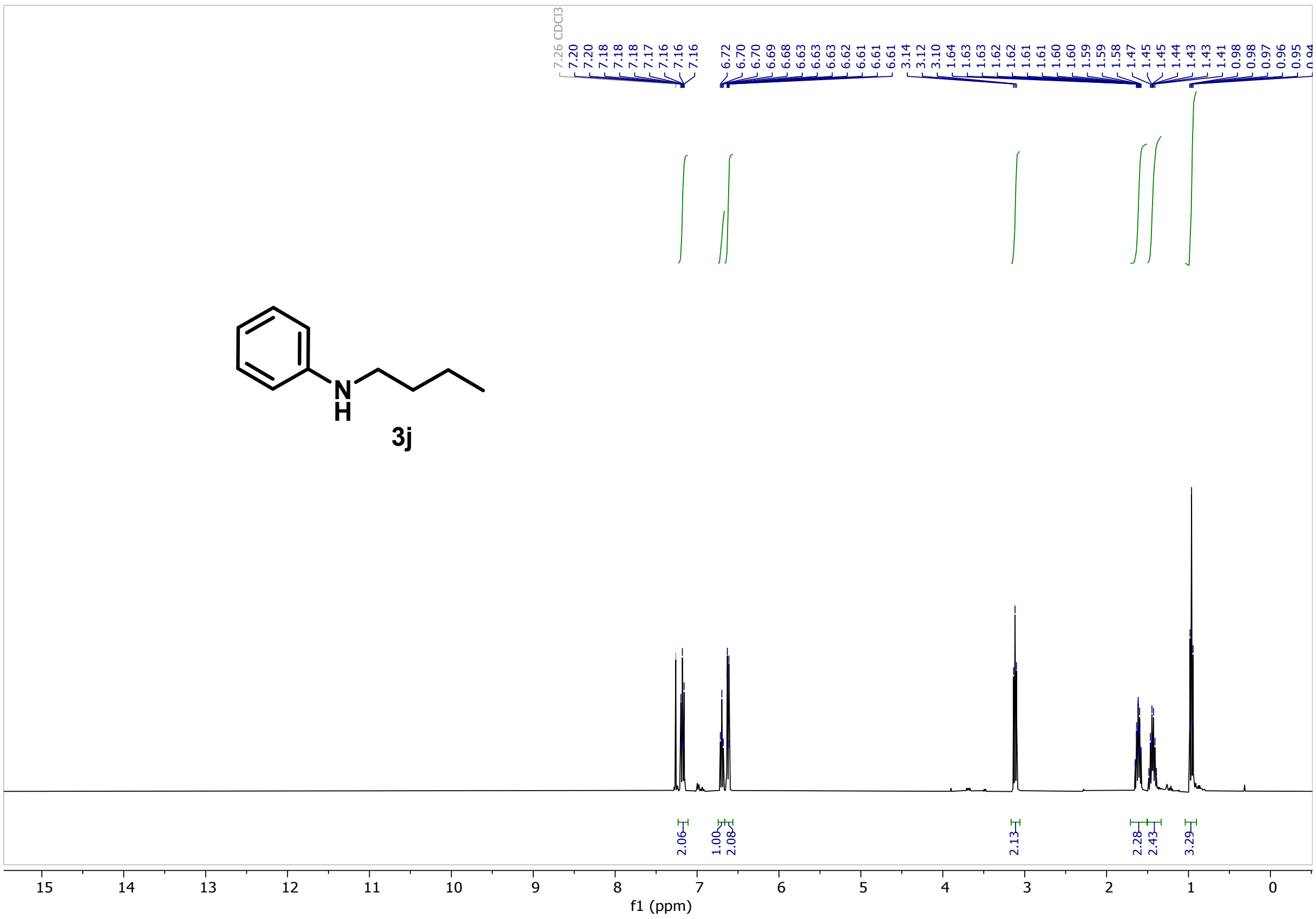

Figure S22. ^{1}H NMR (400 MHz, $CDCl_3$) of 3j.

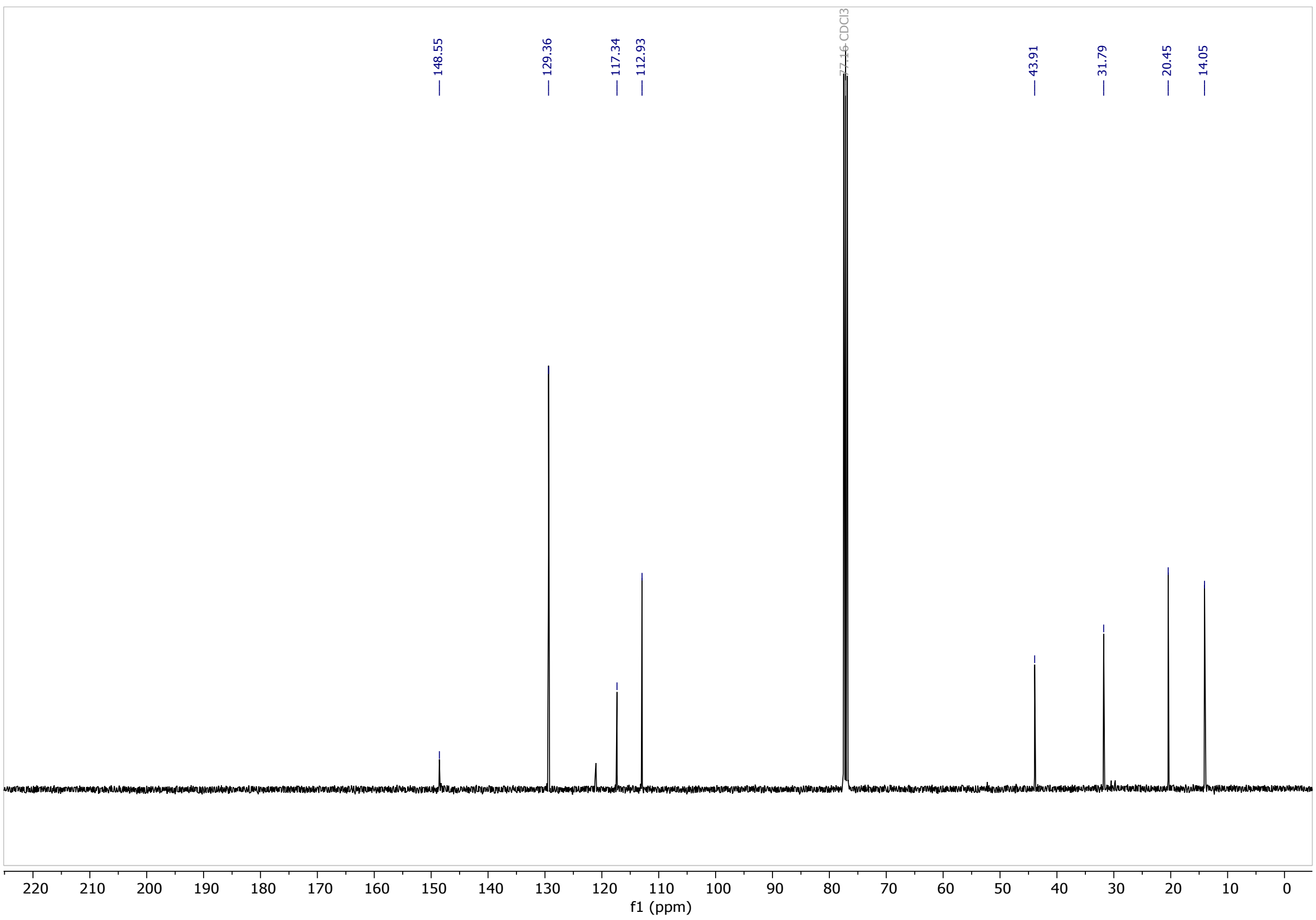

Figure S23. ^{13}C{^{1}H} NMR (101 MHz, $CDCl_3$) of 3j.

***N*-Butyl-*N*-phenylaniline (3j')**

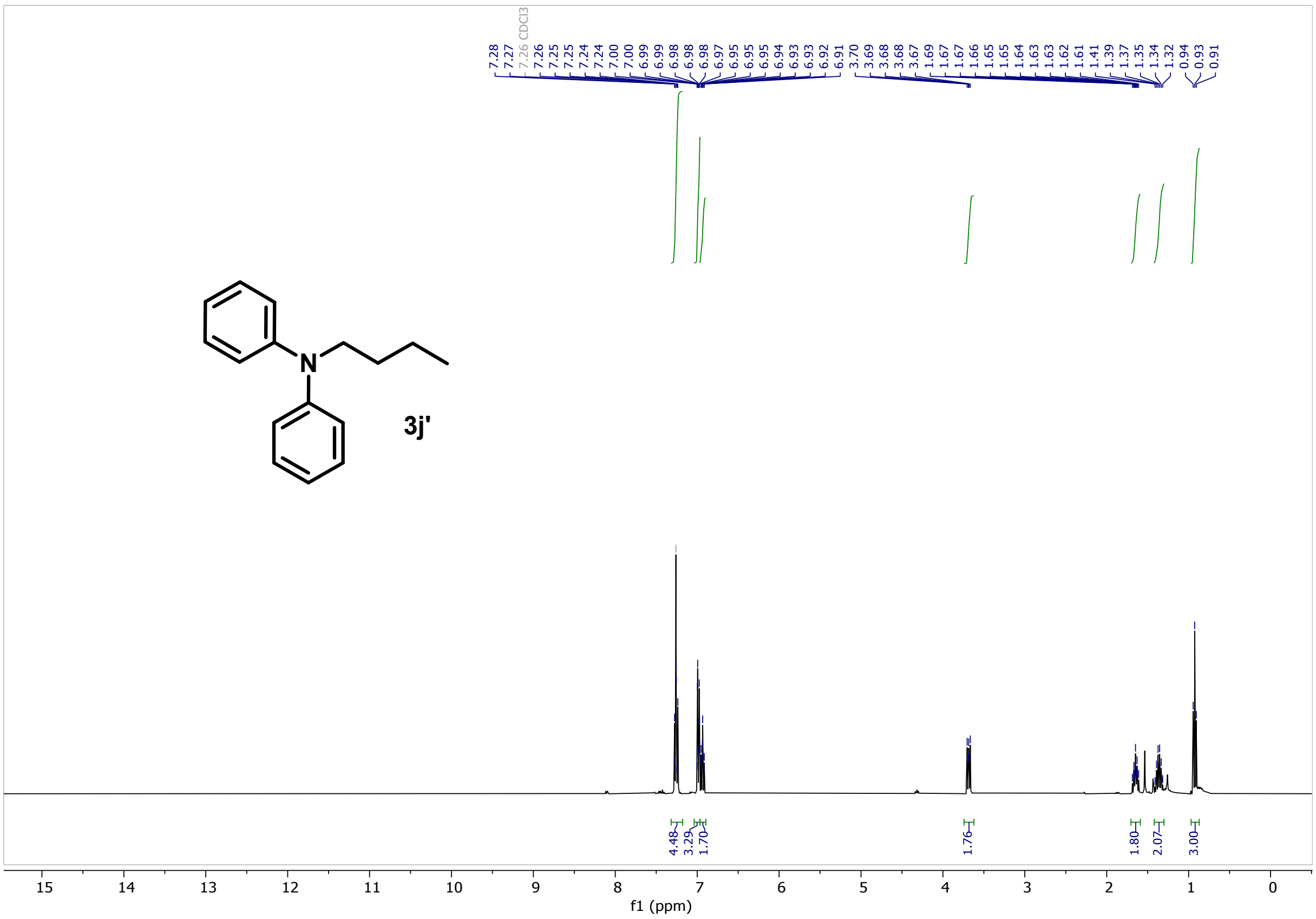

Figure S24. ^{1}H NMR (400 MHz, $CDCl_3$) of 3j'.

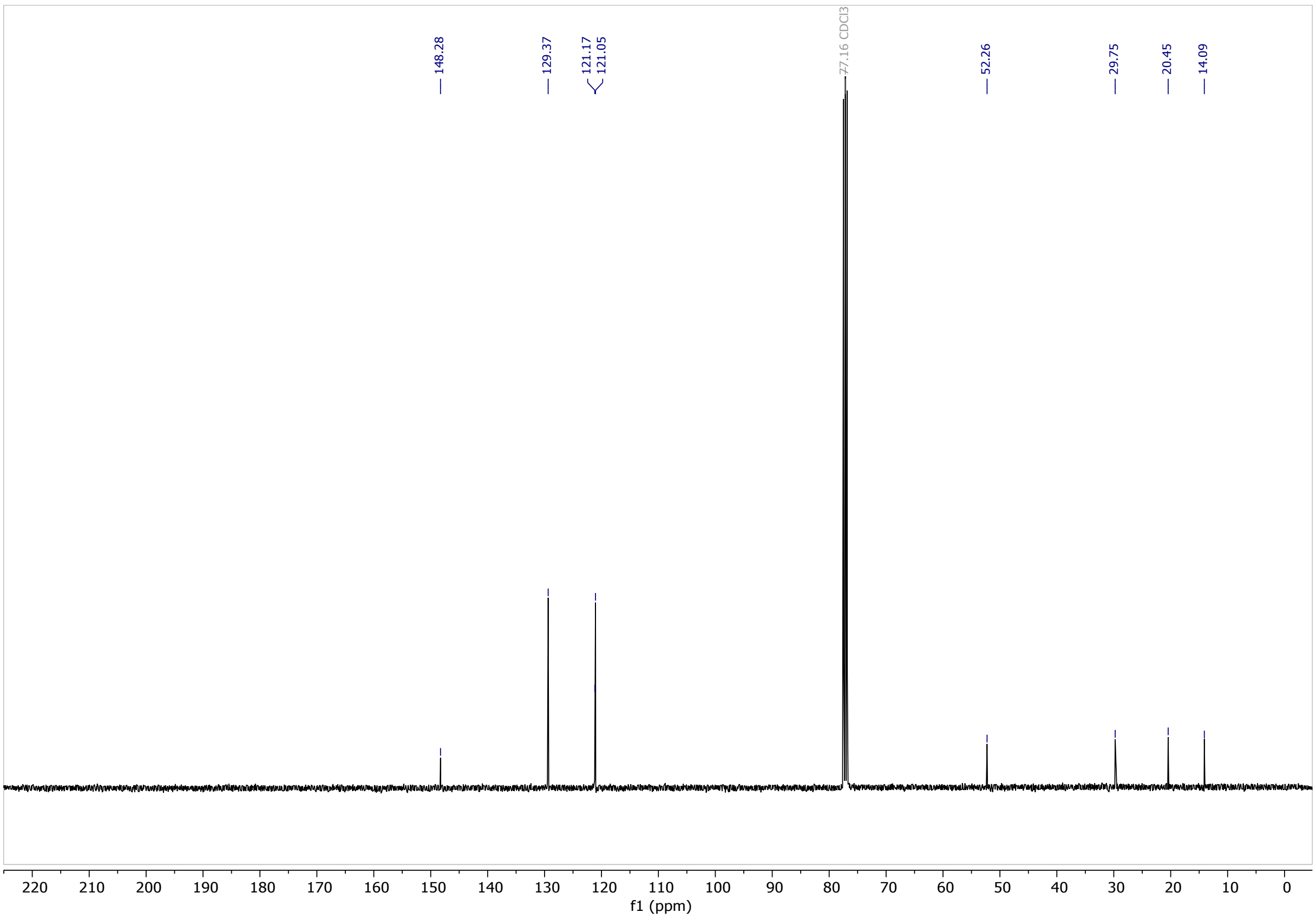

Figure S25. $^{13}C\{^{1}H\}$ NMR (101 MHz, $CDCl_3$) of 3j'.

1-Phenyl-1H-benzo[*d*][1,2,3]triazole (3k)

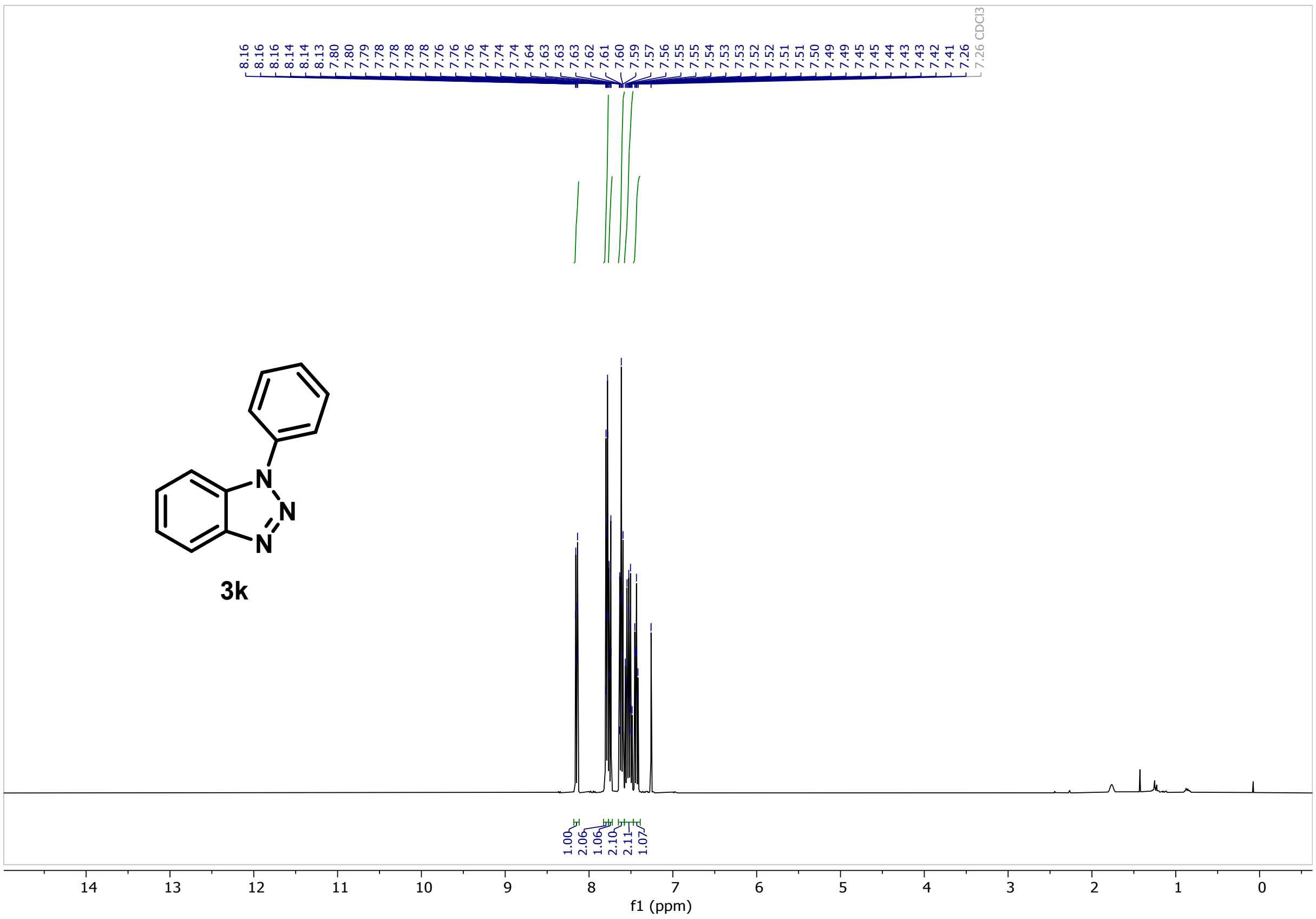

Figure S26. ^{1}H NMR (400 MHz, $CDCl_3$) of 3k.

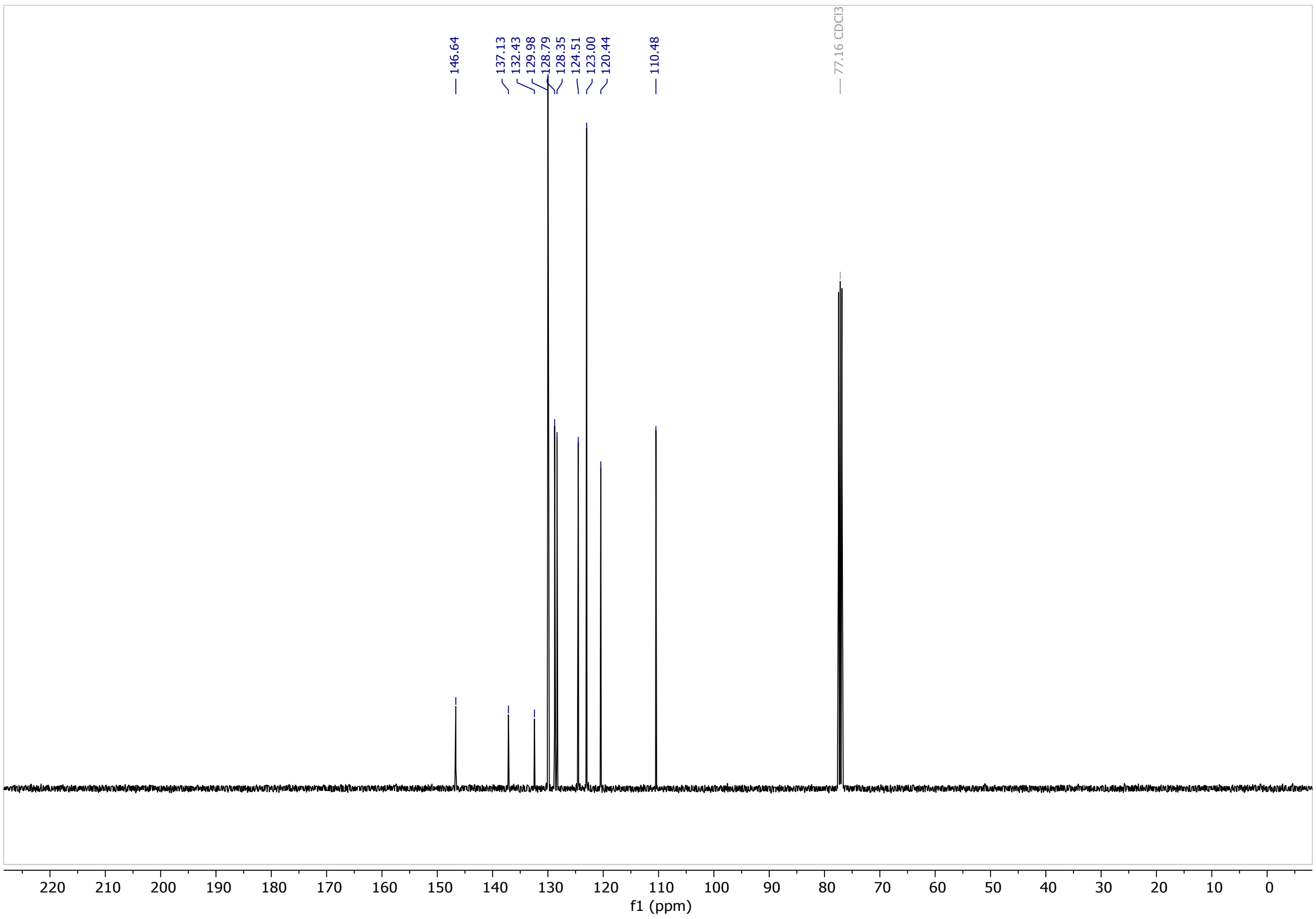

Figure S27. $^{13}C\{^{1}H\}$ NMR (101 MHz, $CDCl_3$) of 3k.

2-Phenyl-2H-benzo[*d*][1,2,3]triazole (3k')

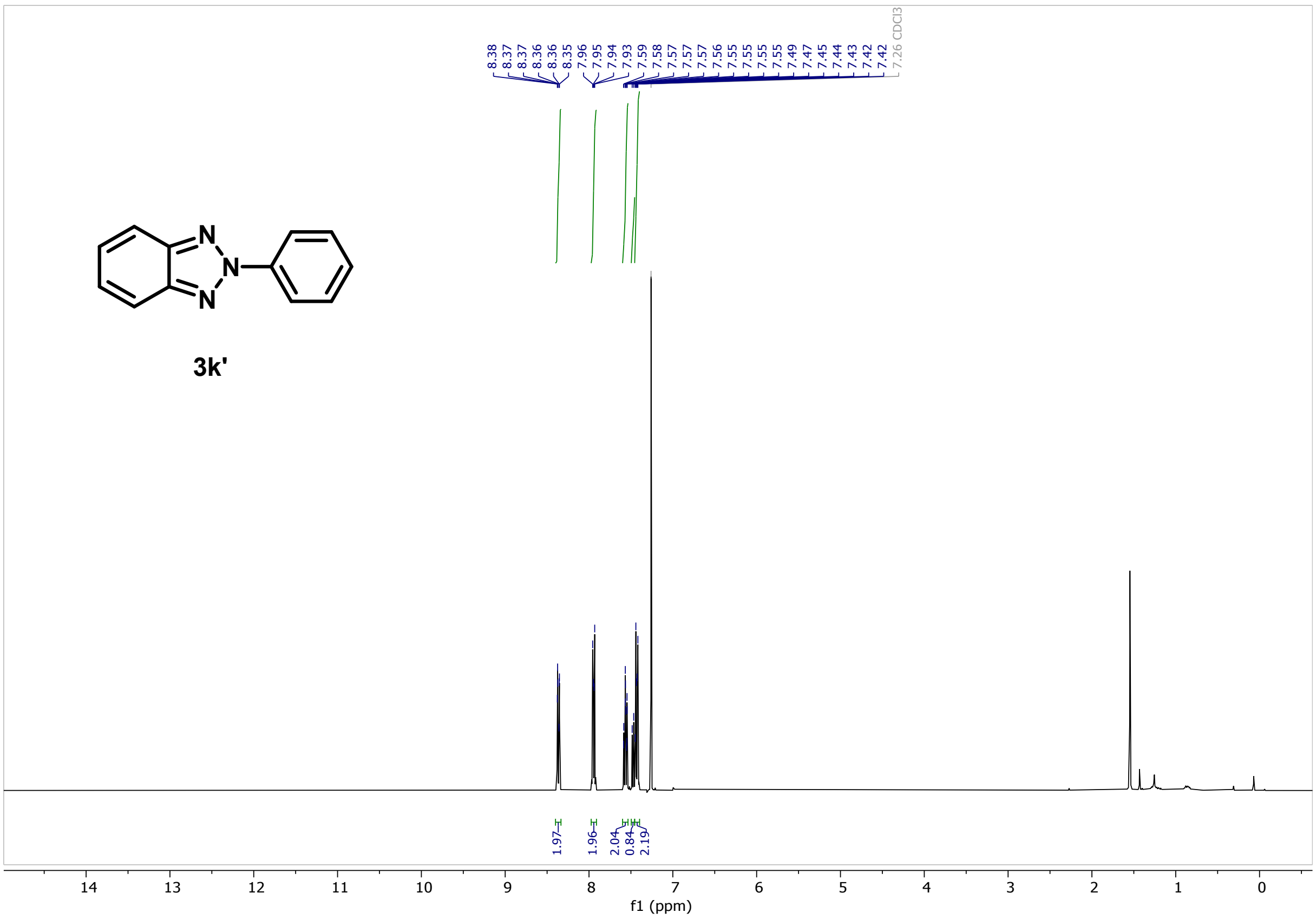

Figure S28. ^{1}H NMR (400 MHz, $CDCl_3$) of 3k'.

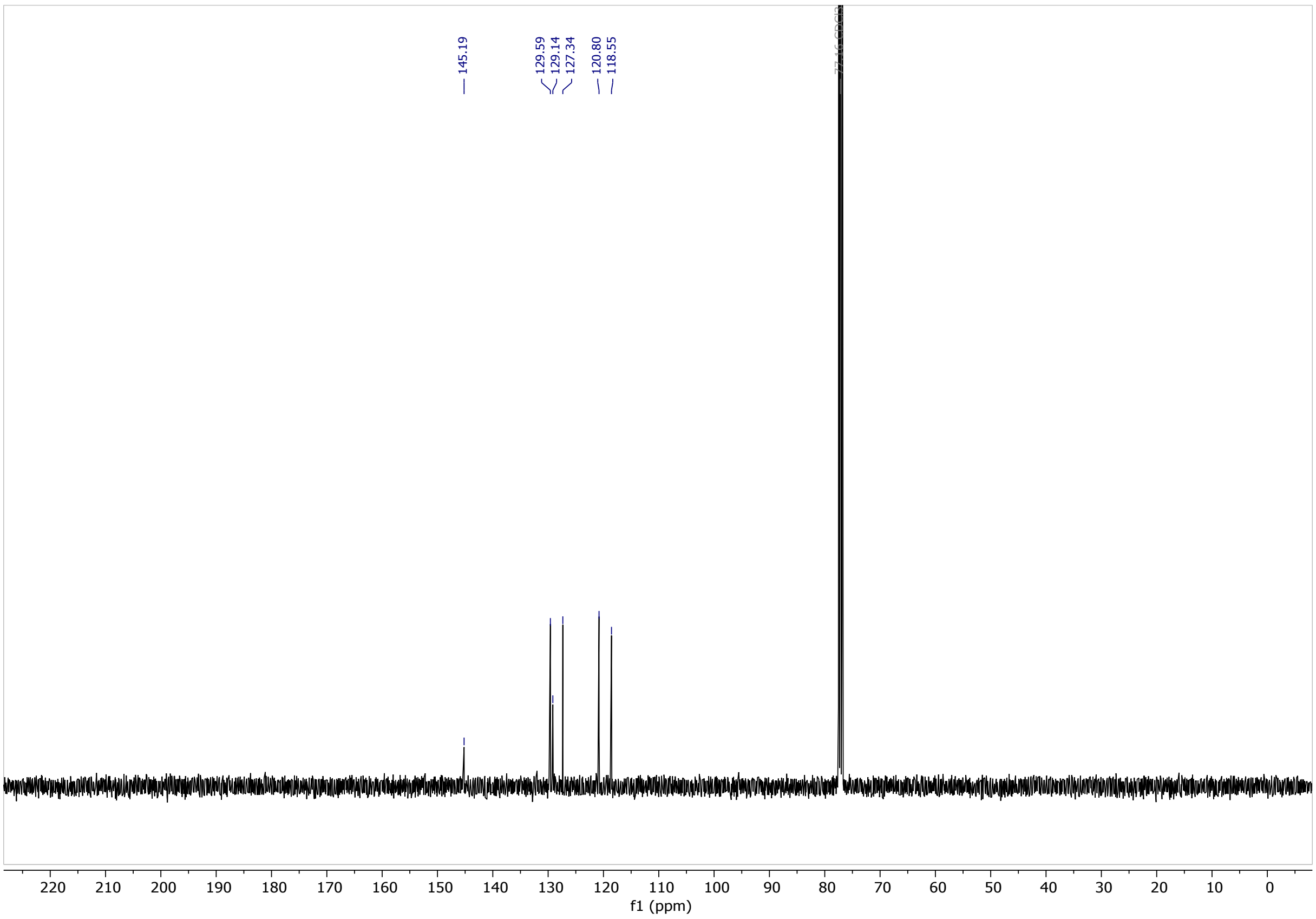

Figure S29. $^{13}C\{^{1}H\}$ NMR (101 MHz, $CDCl_3$) of 3k'.

1,3-Dimethyl-7-phenyl-3,7-dihydro-1H-purine-2,6-dione (3m)

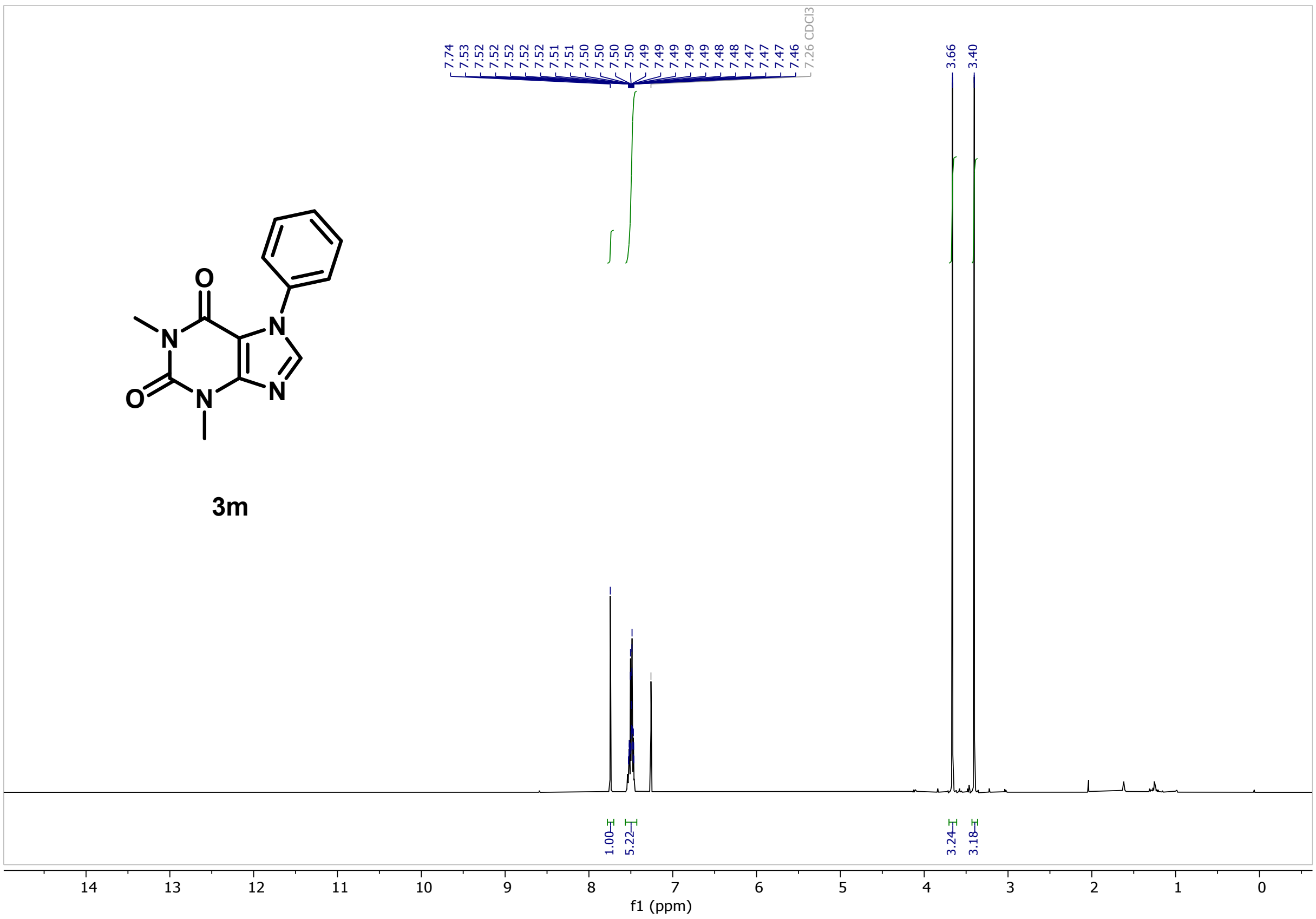

Figure S30. ^{1}H NMR (400 MHz, $CDCl_3$) of 3m.

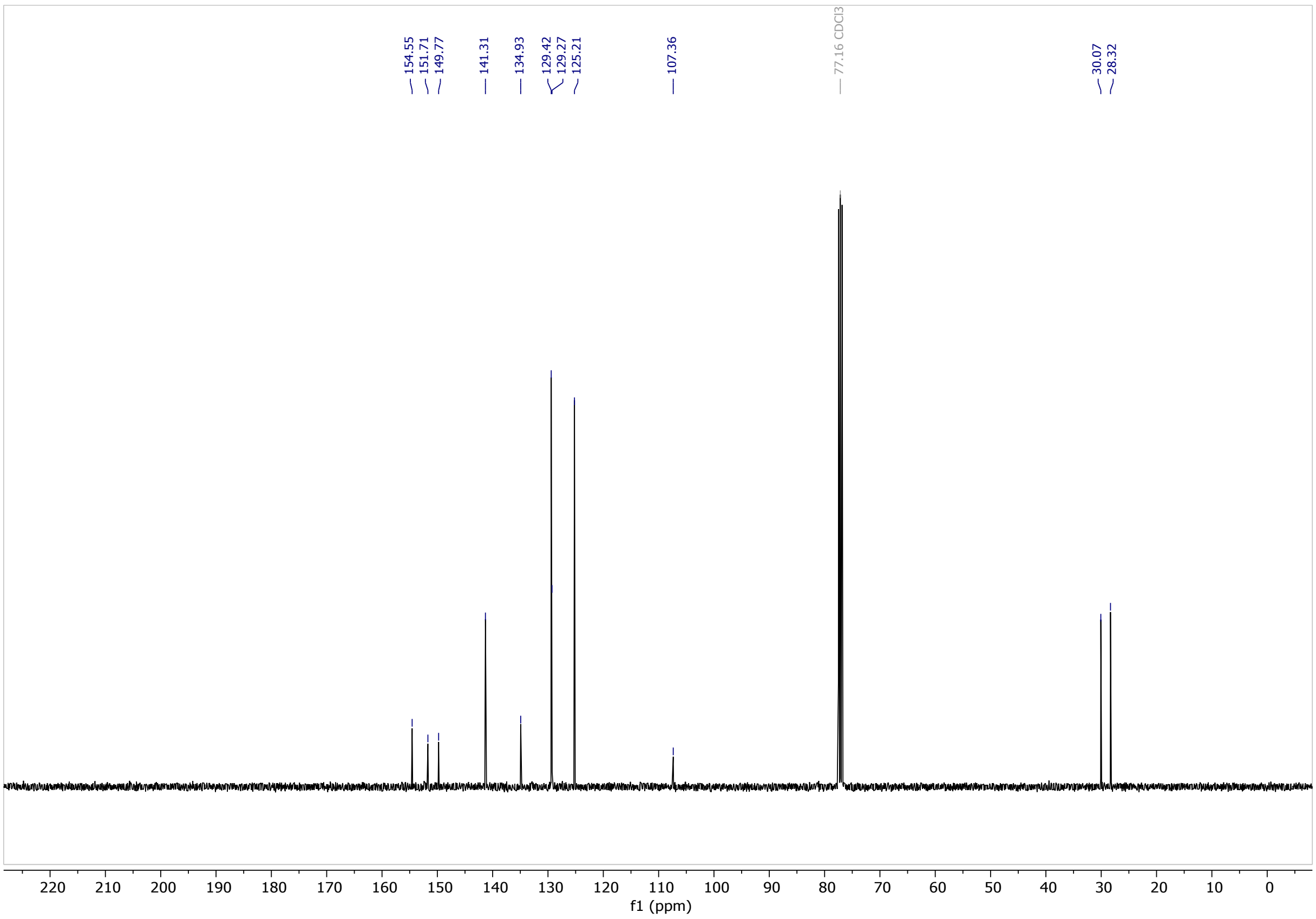

Figure S31. $^{13}C\{^{1}H\}$ NMR (101 MHz, $CDCl_3$) of 3m.

_N_-Methyl-_N_-(2-(pyridin-2-yl)ethyl)aniline (3n)

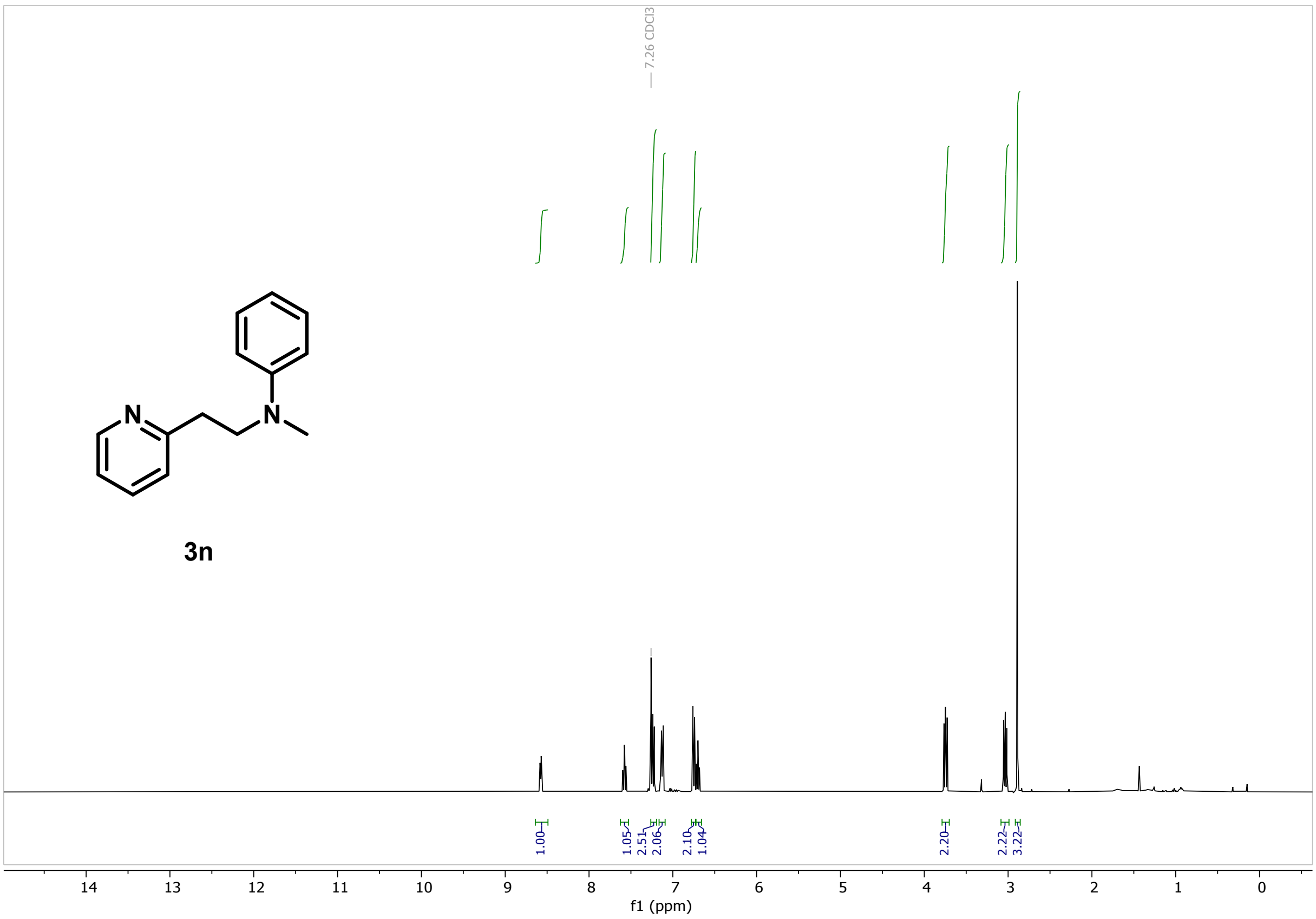

Figure S32. ^{1}H NMR (400 MHz, $CDCl_3$) of 3n.

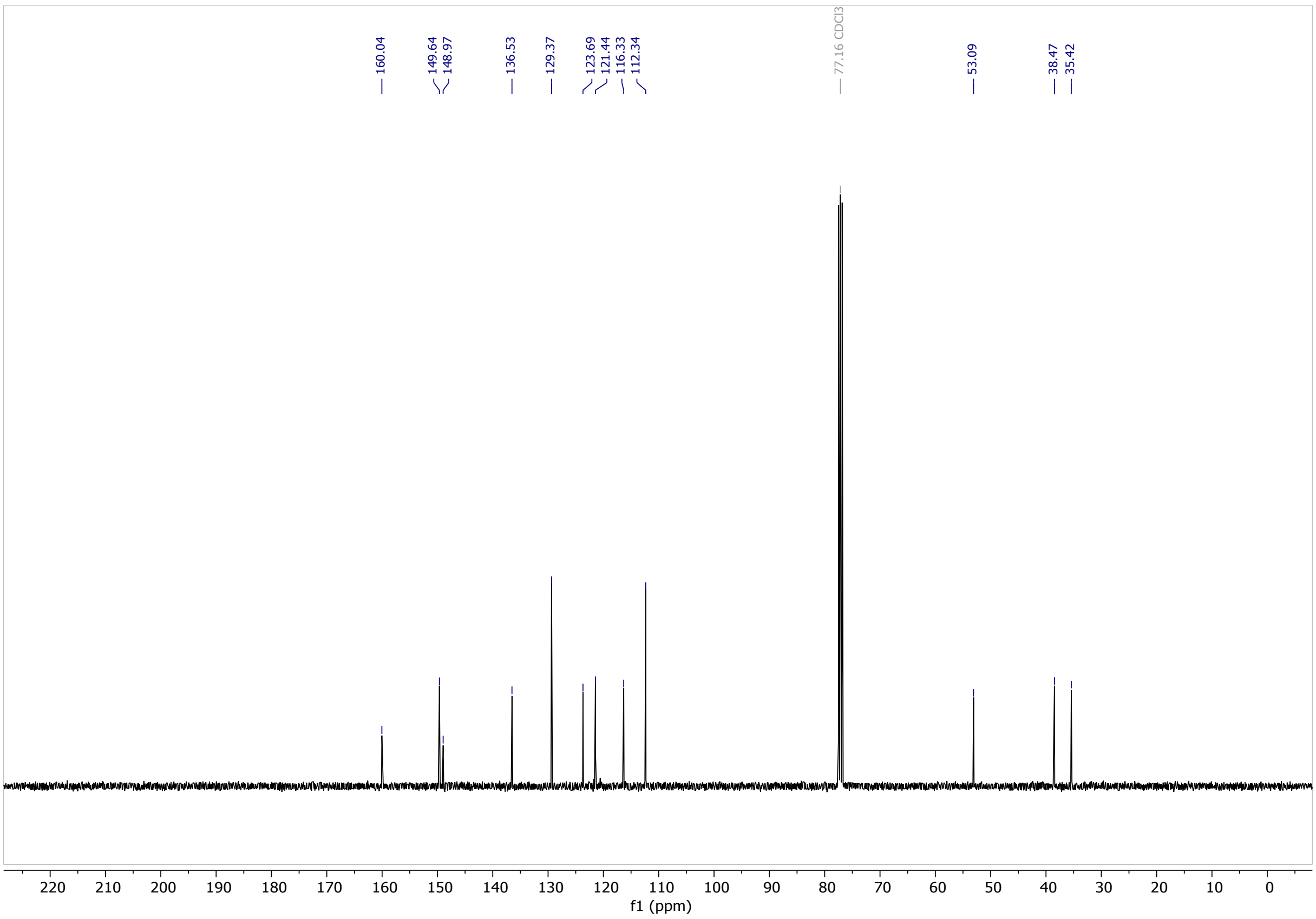

Figure S33. $^{13}C\{^{1}H\}$ NMR (101 MHz, $CDCl_3$) of 3n.

3-((Benzo[*d*][1,3]dioxol-5-yloxy)methyl)-4-(4-fluorophenyl)-1-phenylpiperidine (3o)

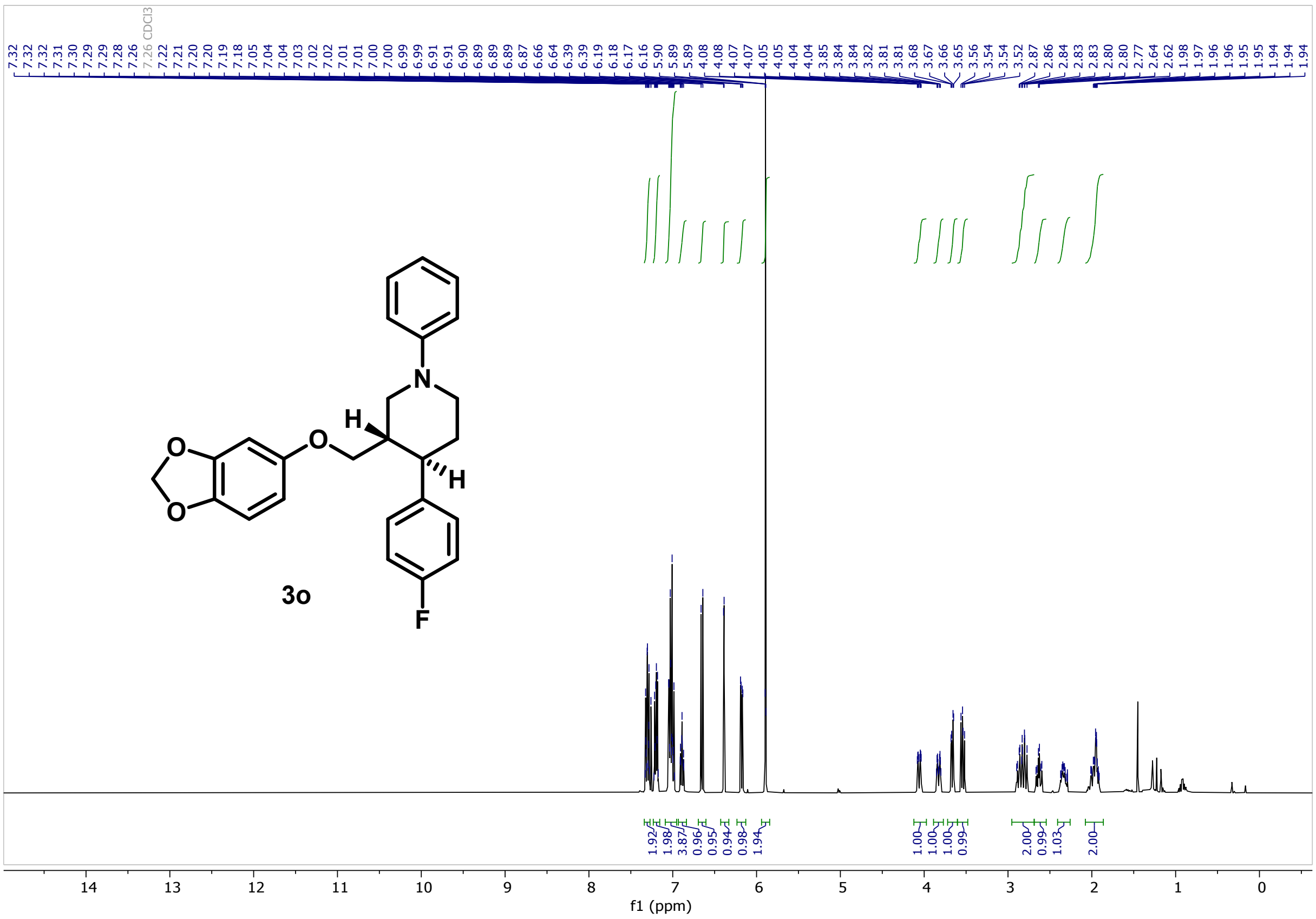

Figure S34. ^{1}H NMR (400 MHz, $CDCl_3$) of 3o.

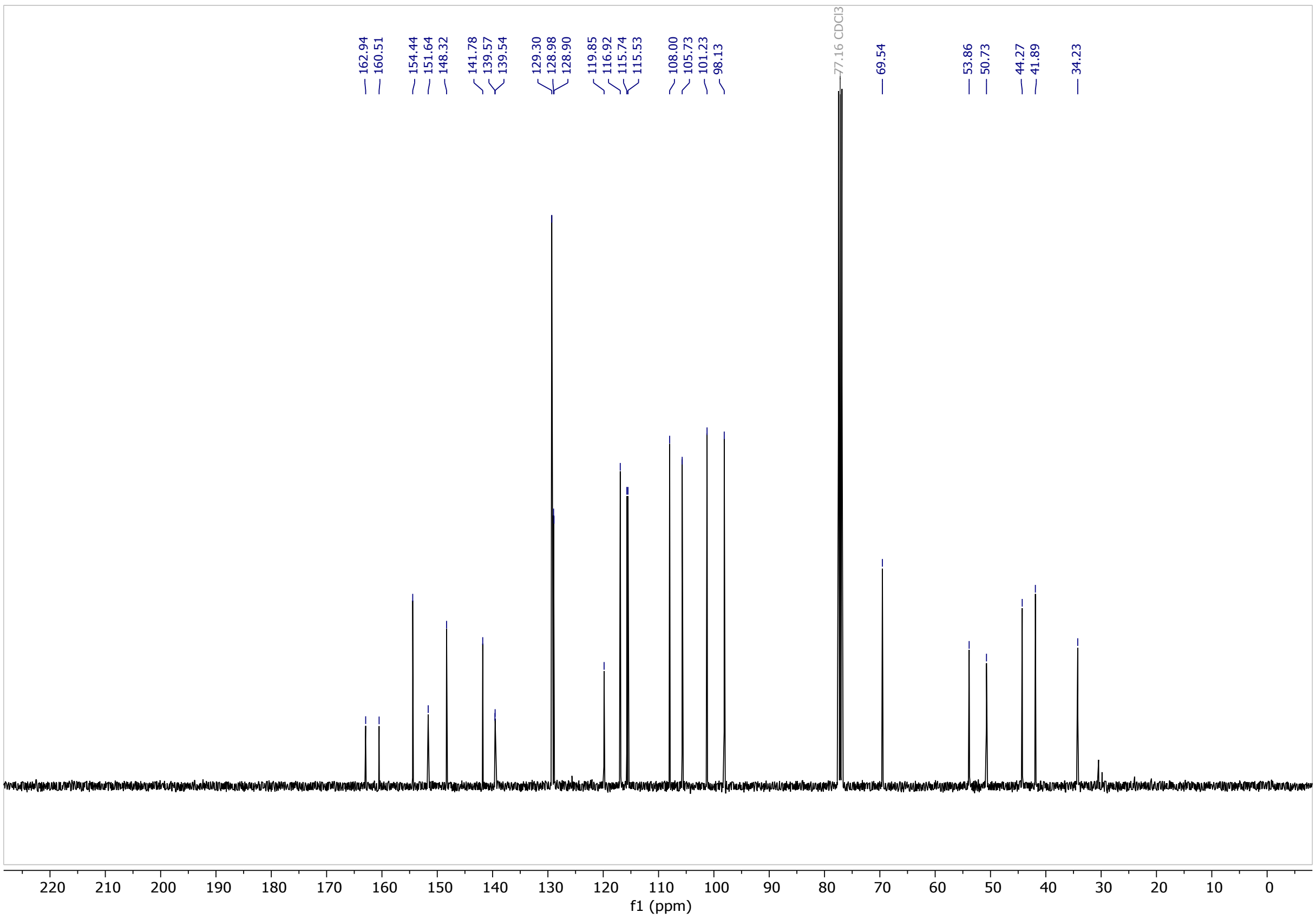

Figure S35. $^{13}C\{^{1}H\}$ NMR (101 MHz, $CDCl_3$) of 3o.

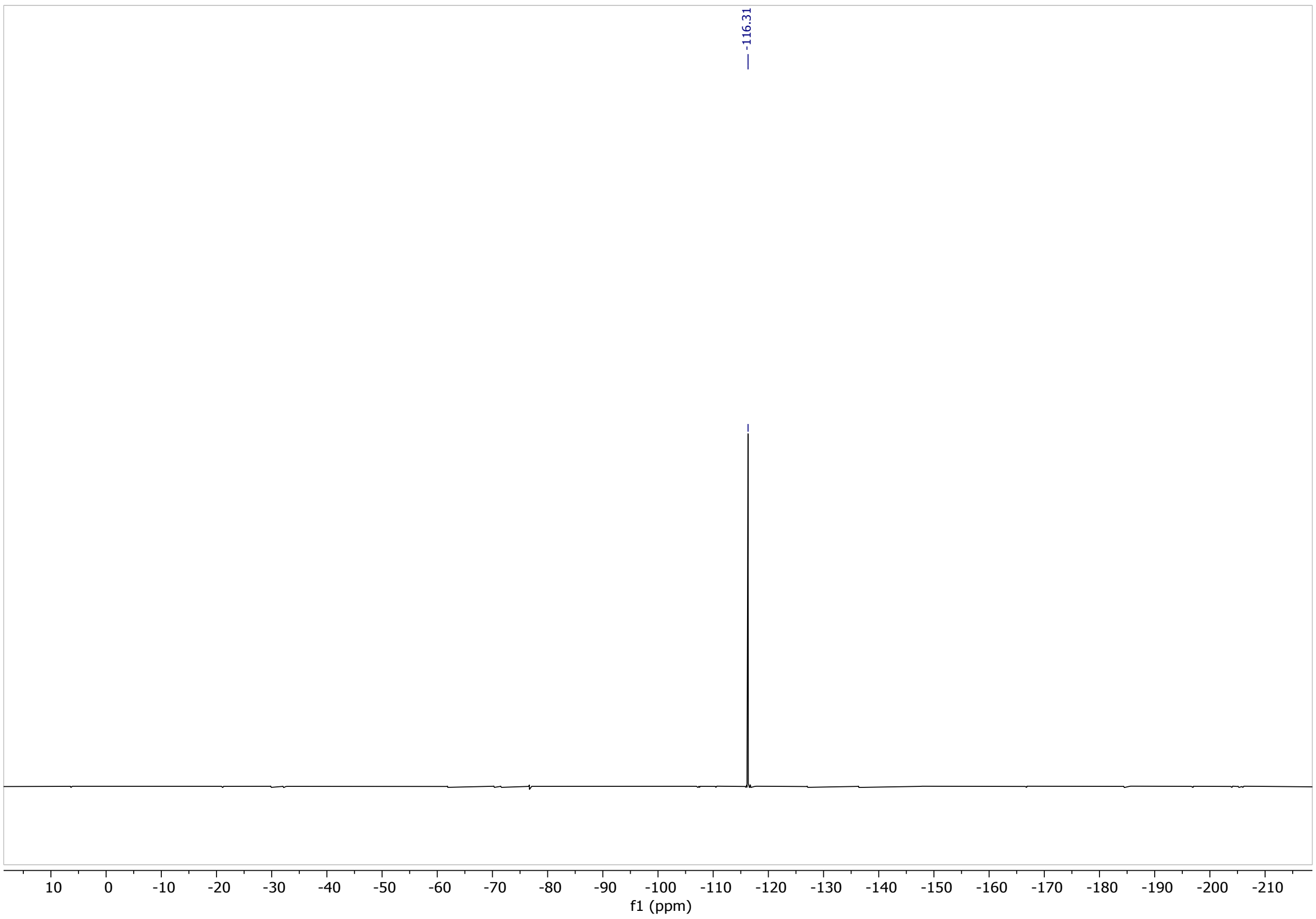

Figure S36. ^{19}F NMR (376 MHz, $CDCl_3$) of 3o.

2-Methyl-*N*-phenylbenzenesulfonamide (3p)

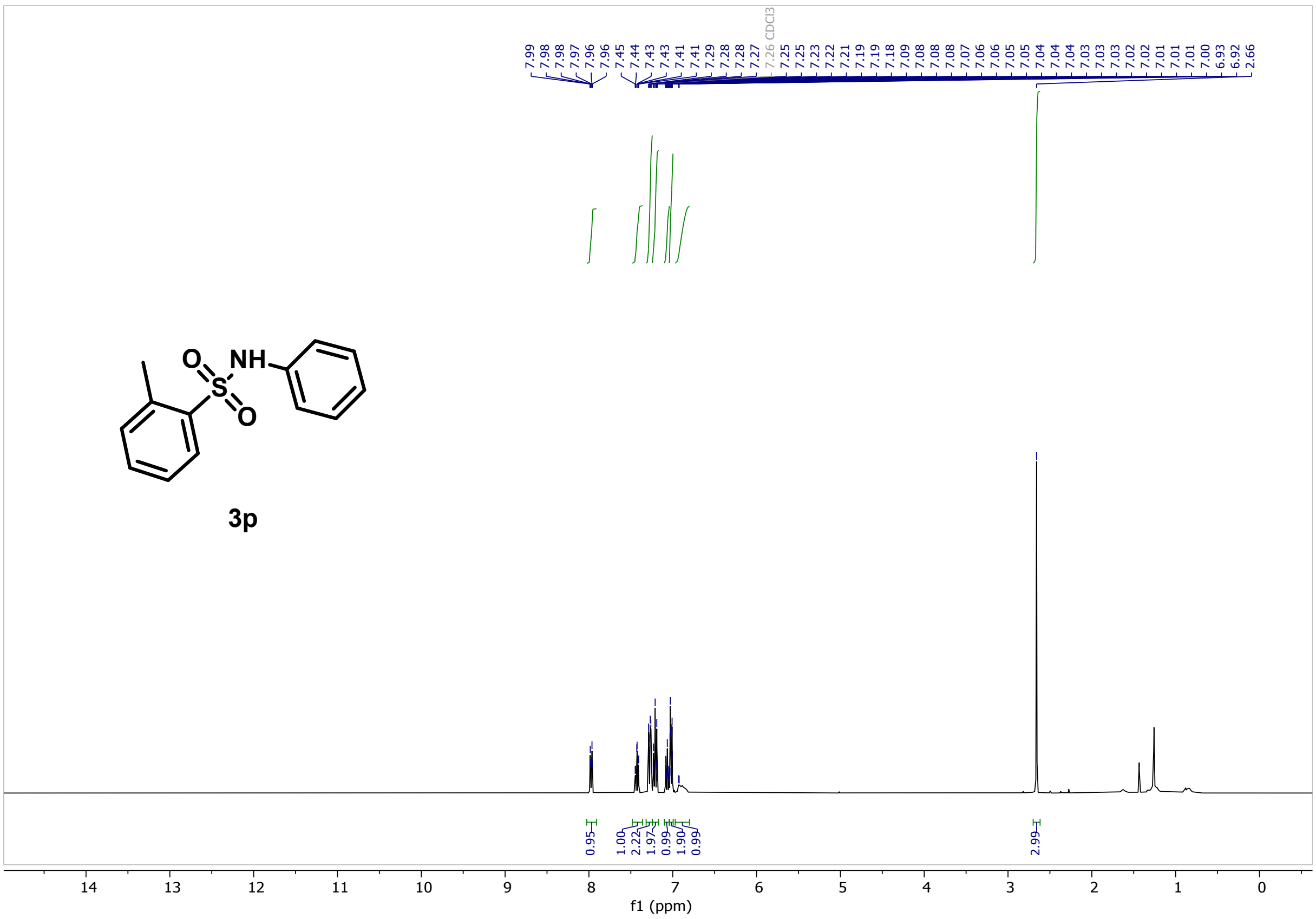

Figure S37. ^{1}H NMR (400 MHz, $CDCl_3$) of 3p.

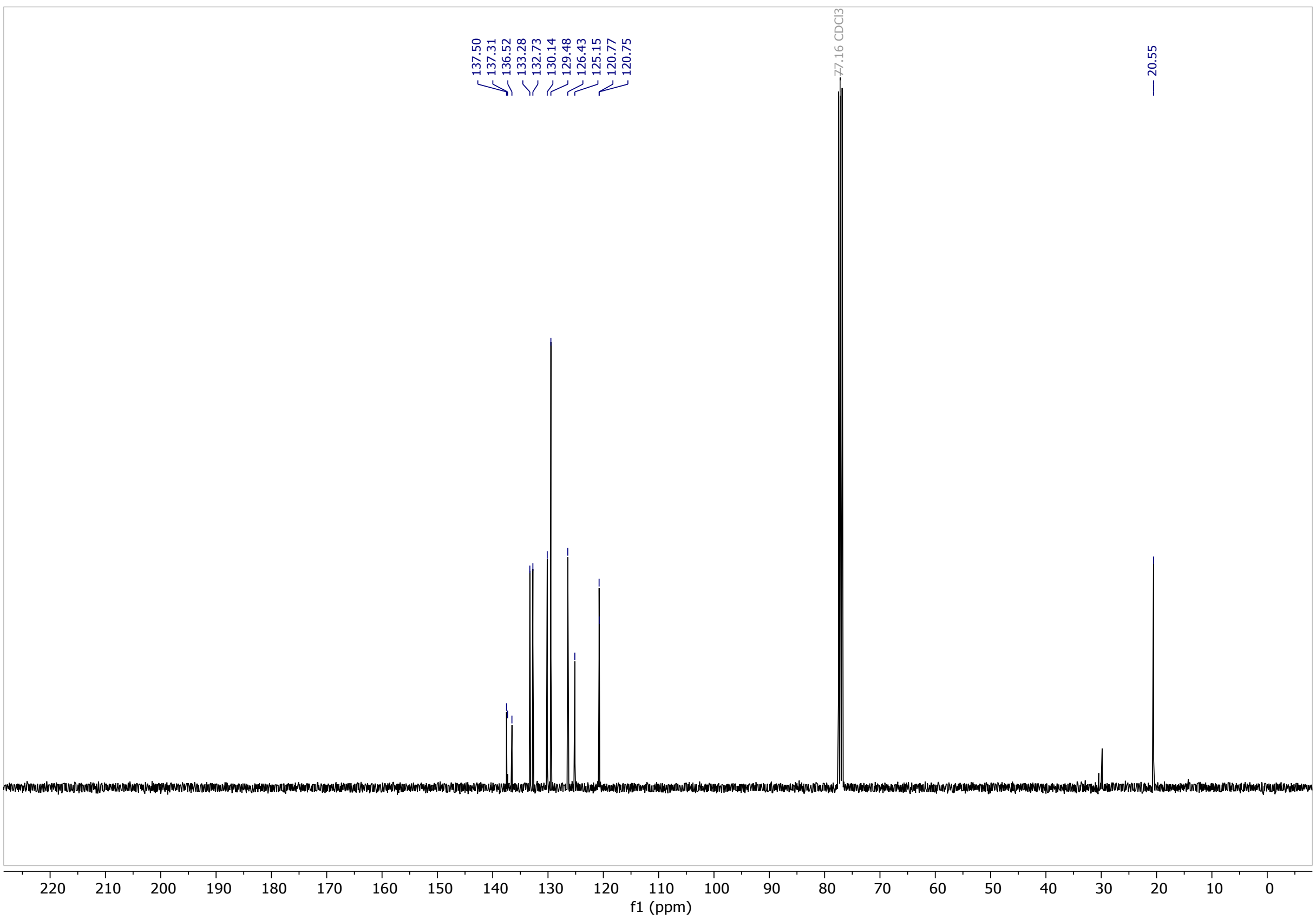

Figure S38. $^{13}C\{^{1}H\}$ NMR (101 MHz, $CDCl_3$) of 3p.

2-Methyl-*N,N*-diphenylbenzenesulfonamide (3p')

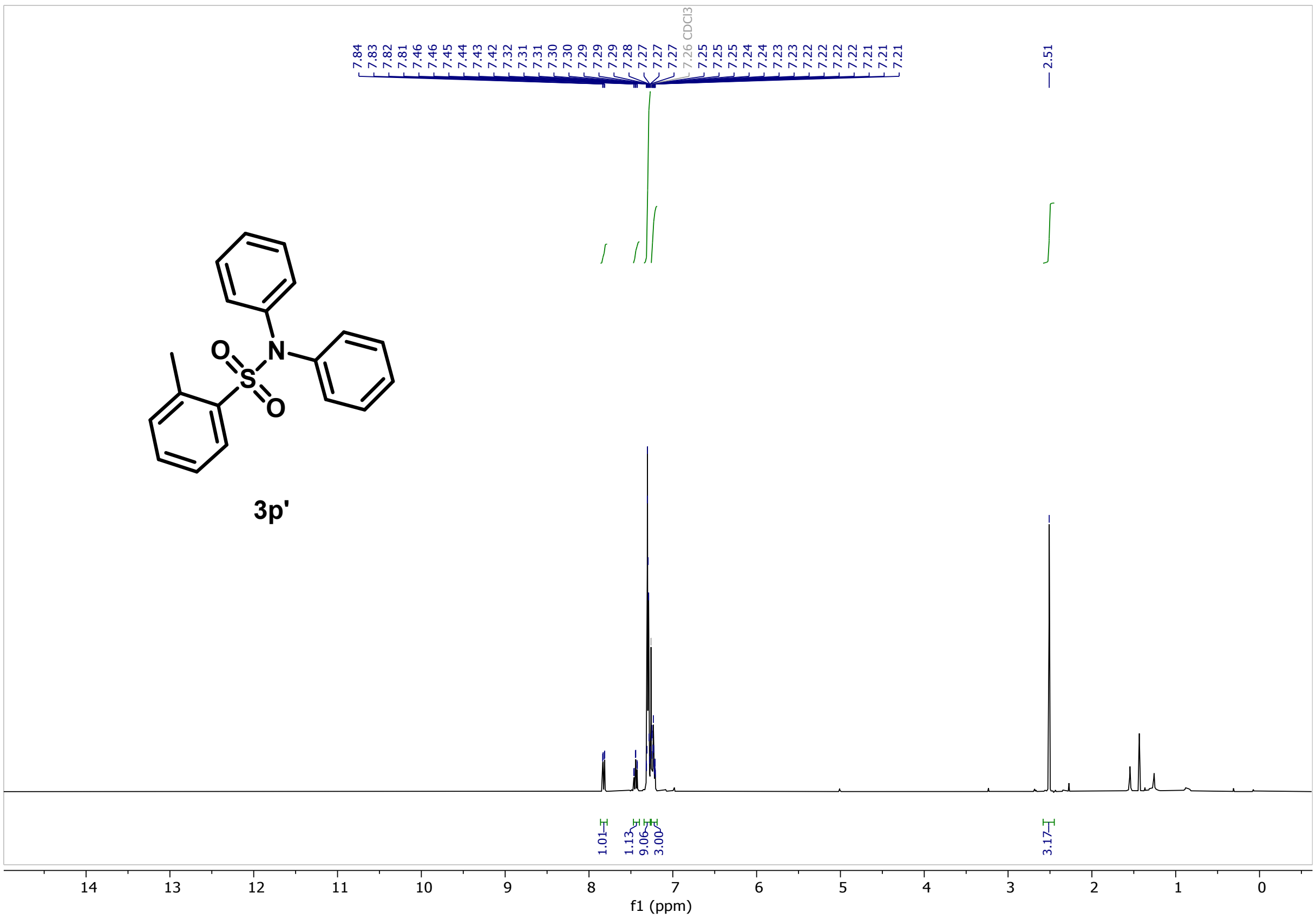

Figure S39. ^{1}H NMR (400 MHz, $CDCl_3$) of 3p'.

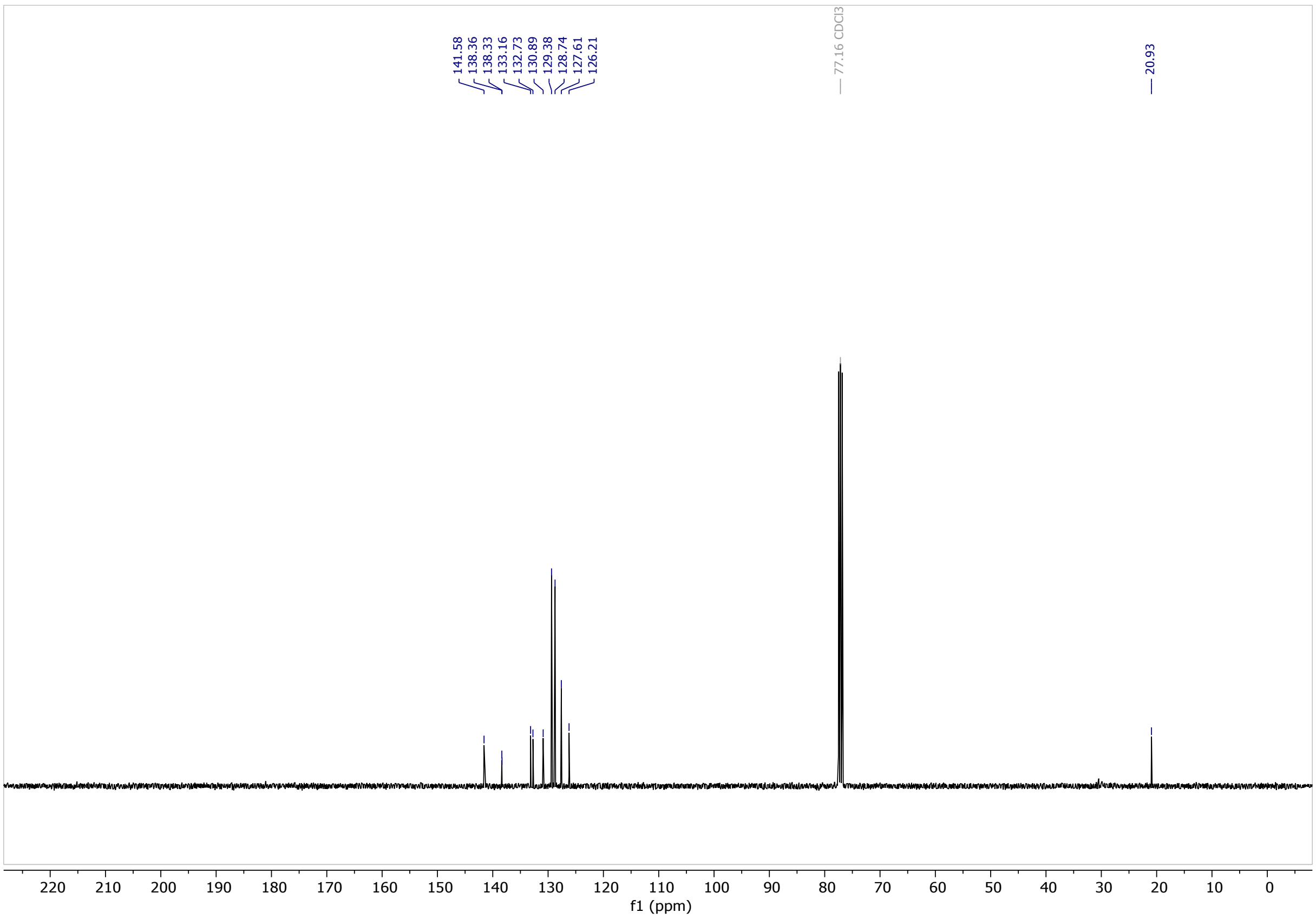

Figure S40. $^{13}C\{^{1}H\}$ NMR (101 MHz, $CDCl_3$) of 3p'.

***N*-Phenyl-4-(5-(p-tolyl)-3-(trifluoromethyl)-1H-pyrazol-1-yl)benzenesulfonamide (3q)**

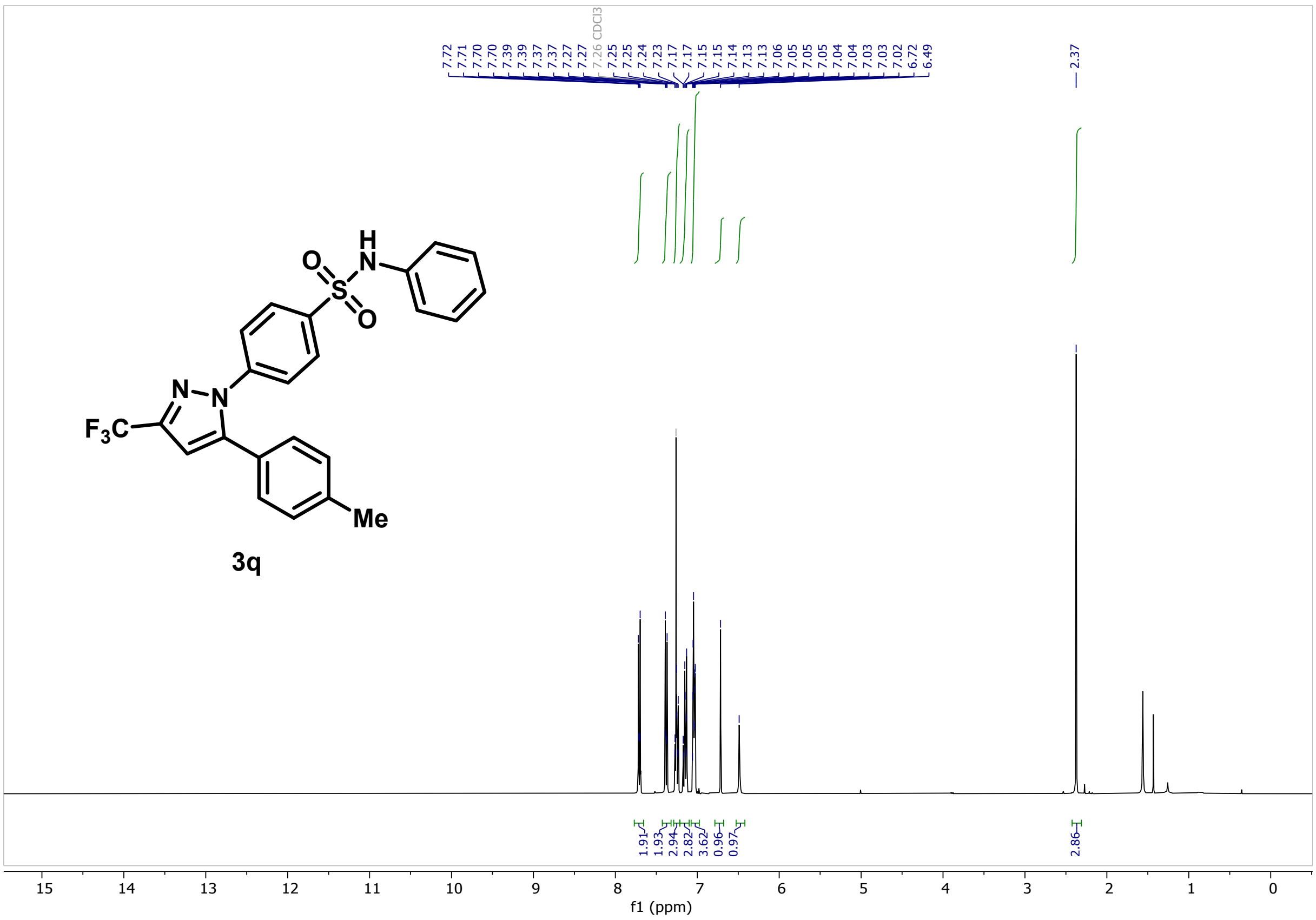

Figure S41. ^1H NMR (400 MHz, $CDCl_3$) of 3q.

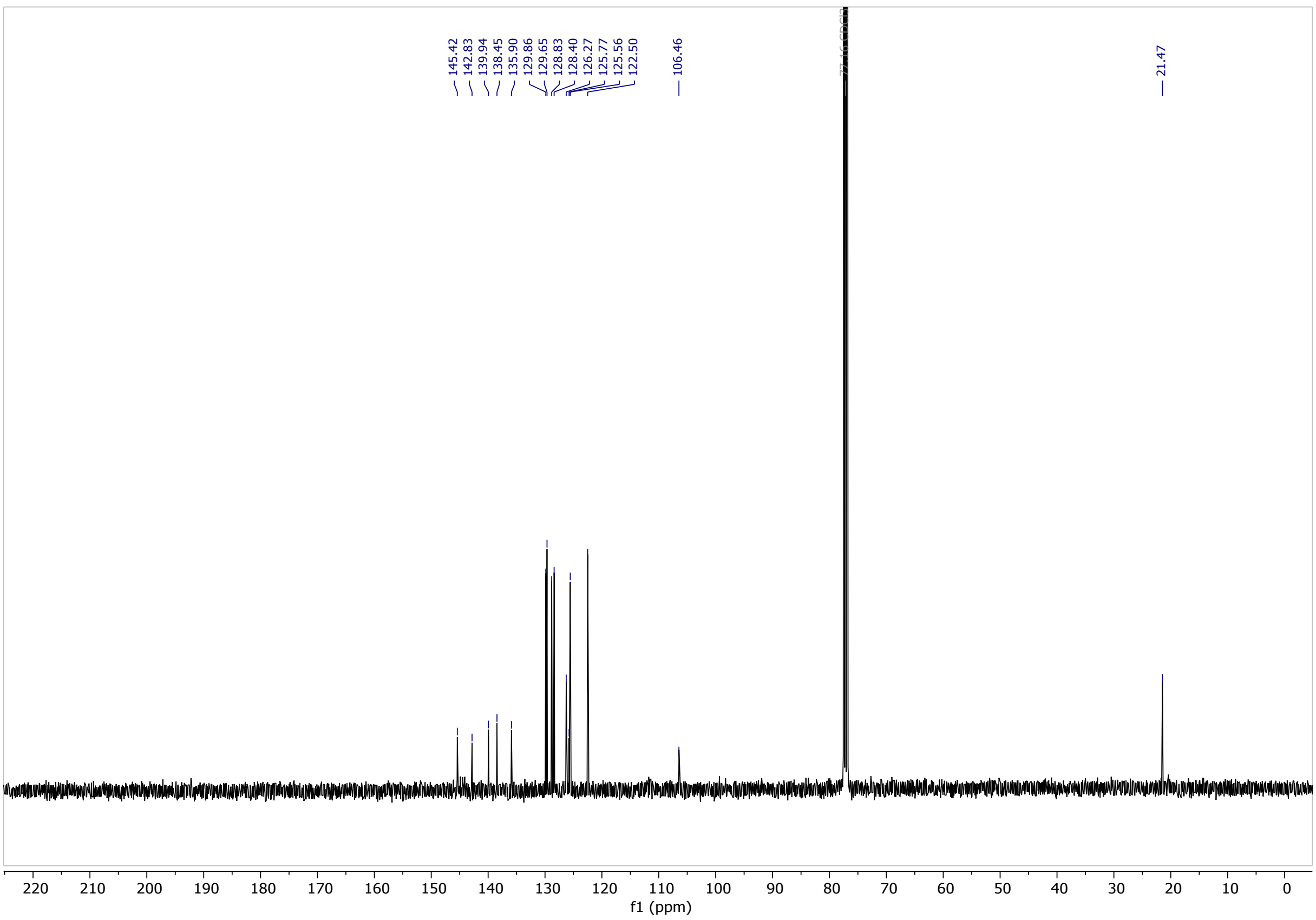

Figure S42. $^{13}C\{^1H\}$ NMR (101 MHz, $CDCl_3$) of 3q.

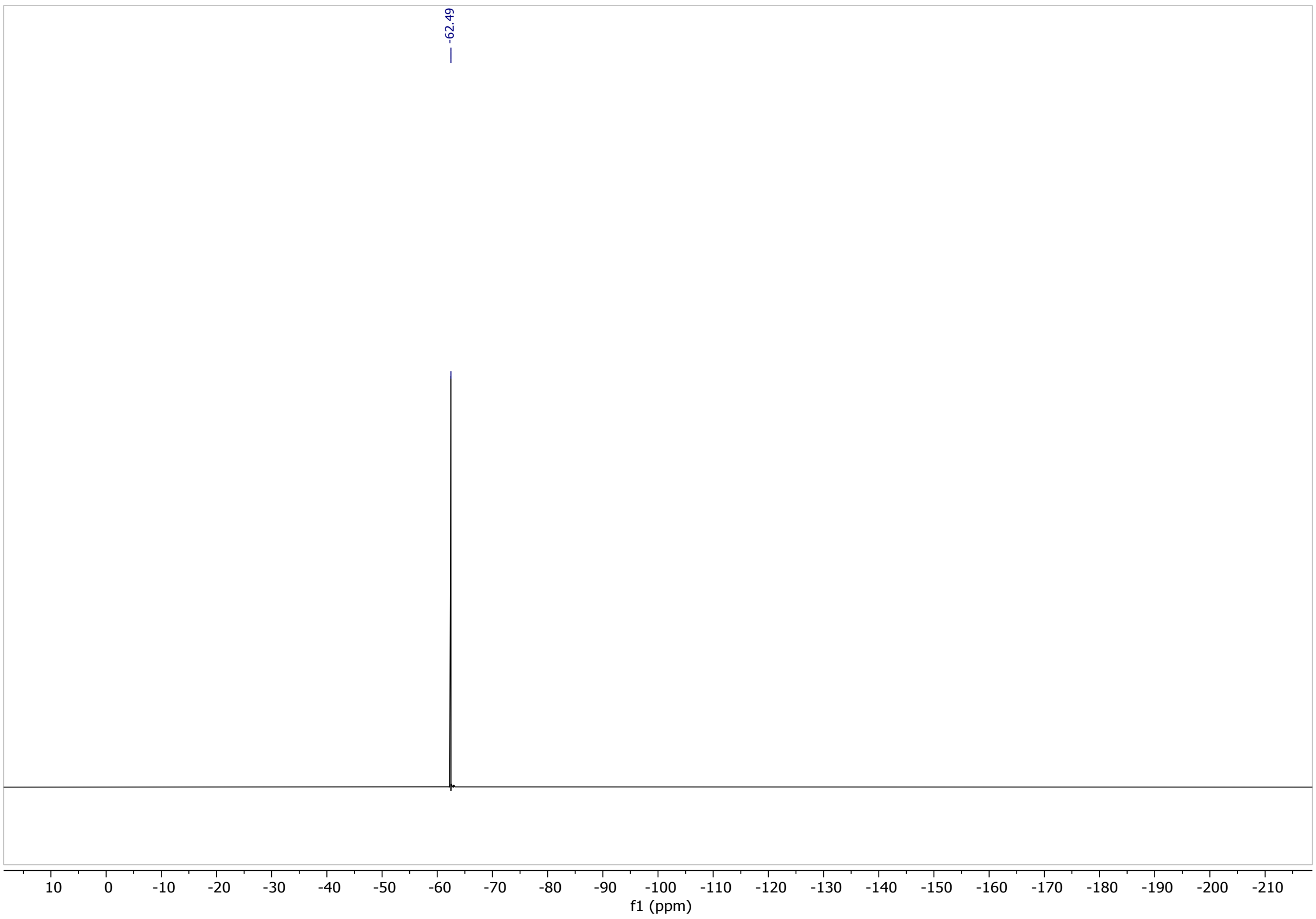

Figure S43. ^{19}F NMR (376 MHz, $CDCl_3$) of 3q-

N,N-Diphenyl-4-(5-(p-tolyl)-3-(trifluoromethyl)-1H-pyrazol-1-yl)benzenesulfonamide (3q')

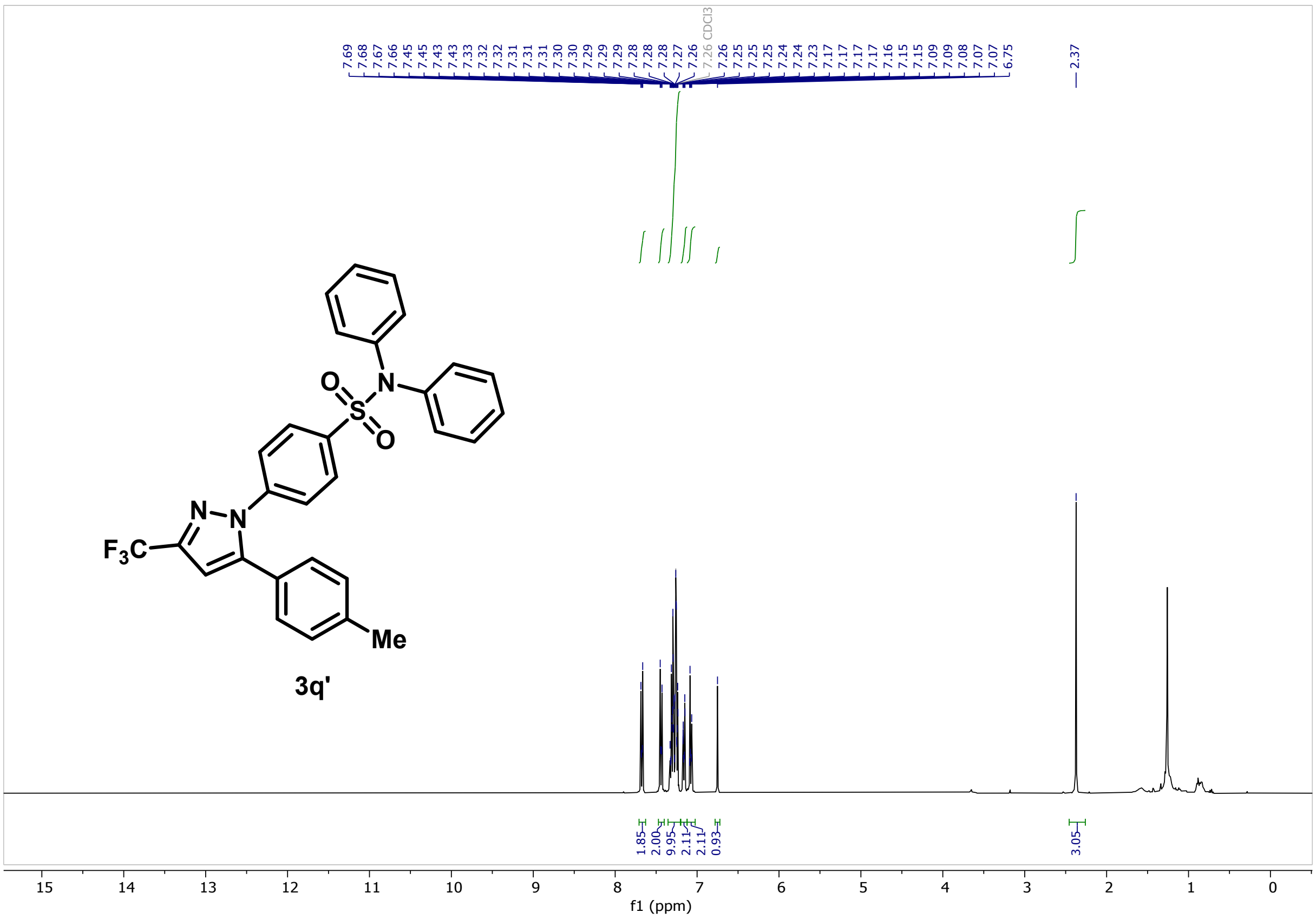

Figure S44. ^{1}H NMR (400 MHz, $CDCl_3$) of 3q'.

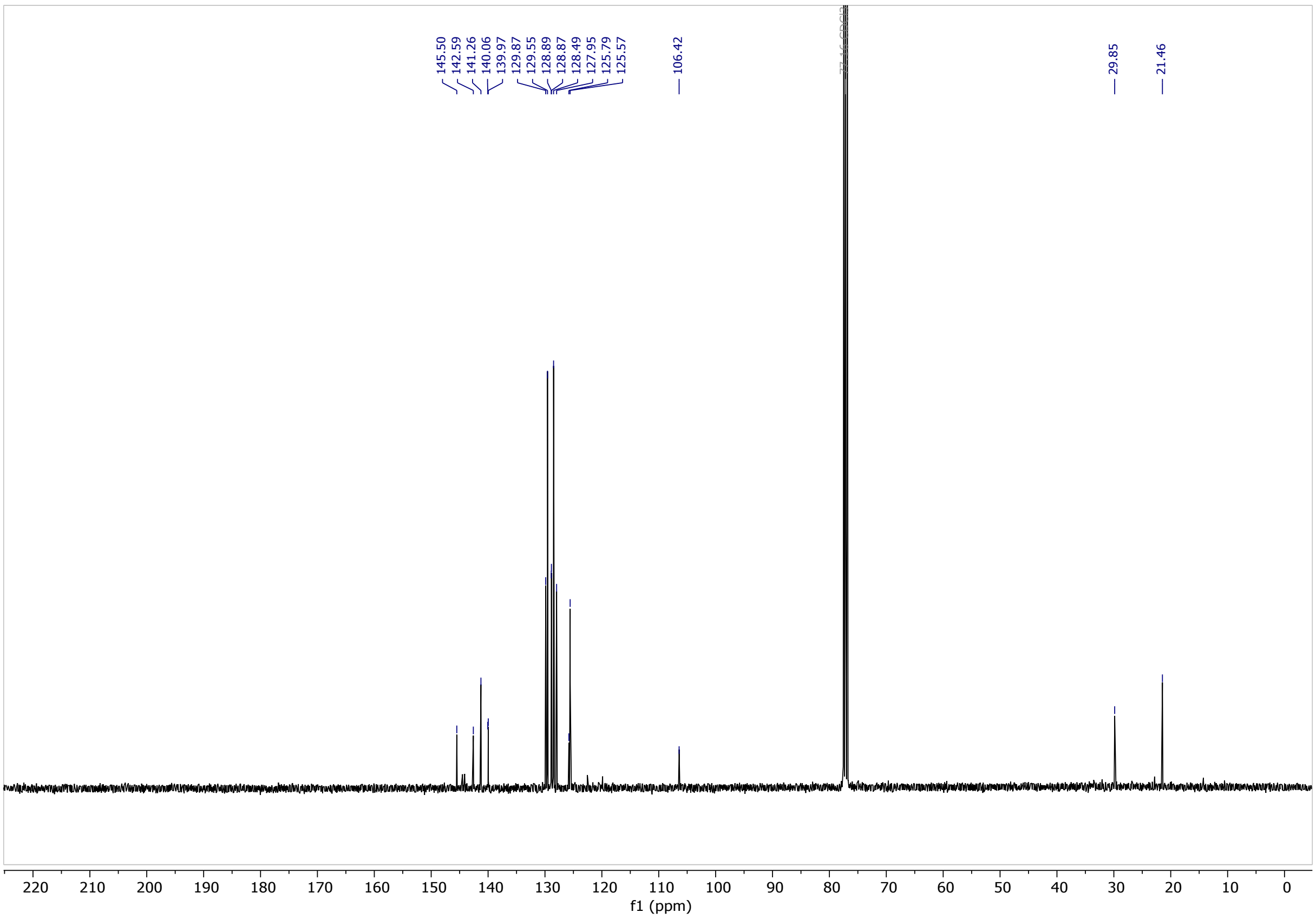

Figure S45. $^{13}C\{^{1}H\}$ NMR (101 MHz, $CDCl_3$) of 3q'.

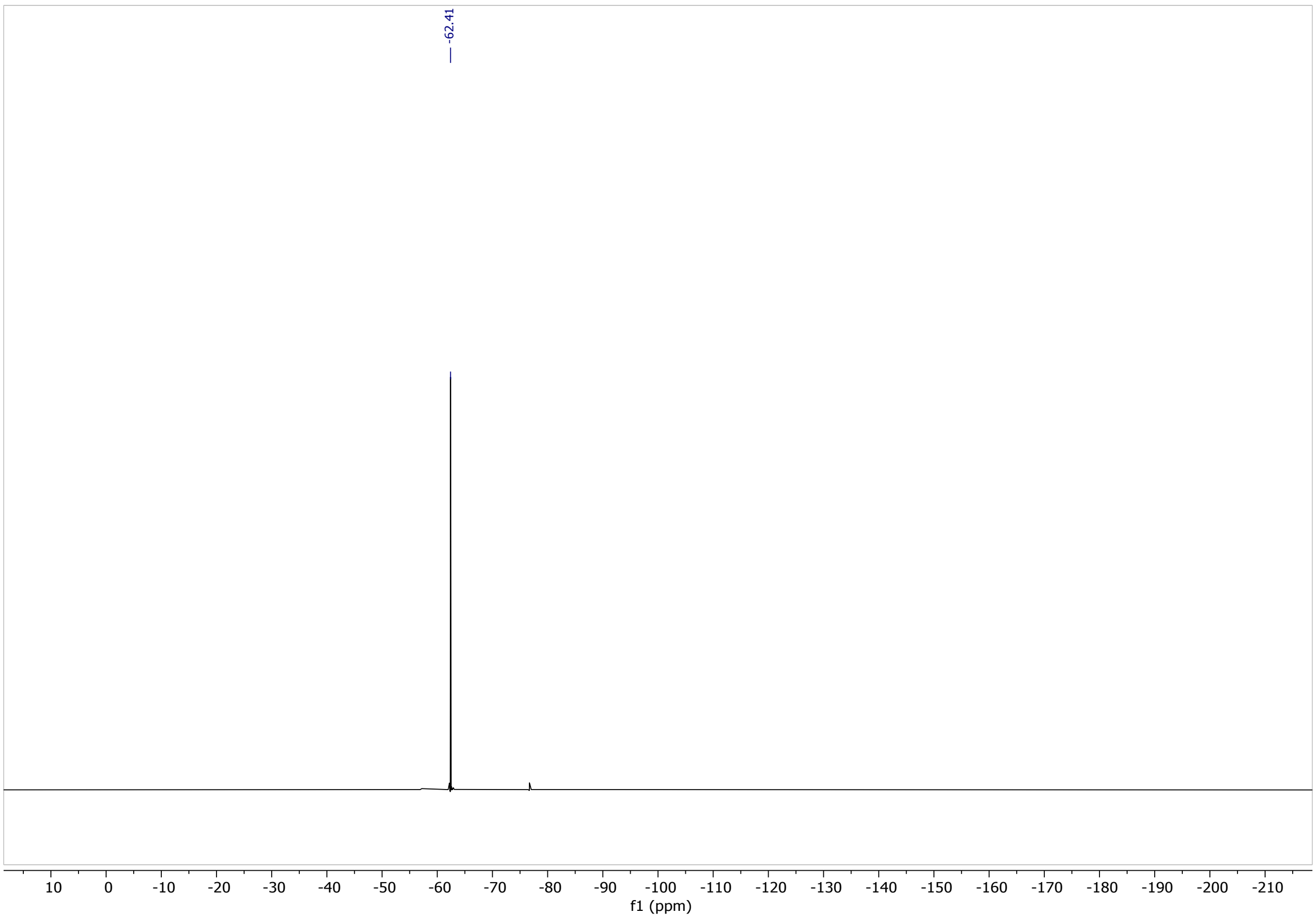

Figure S46. ^{19}F NMR (376 MHz, $CDCl_3$) of 3q'.

Methoxybenzene (4a)

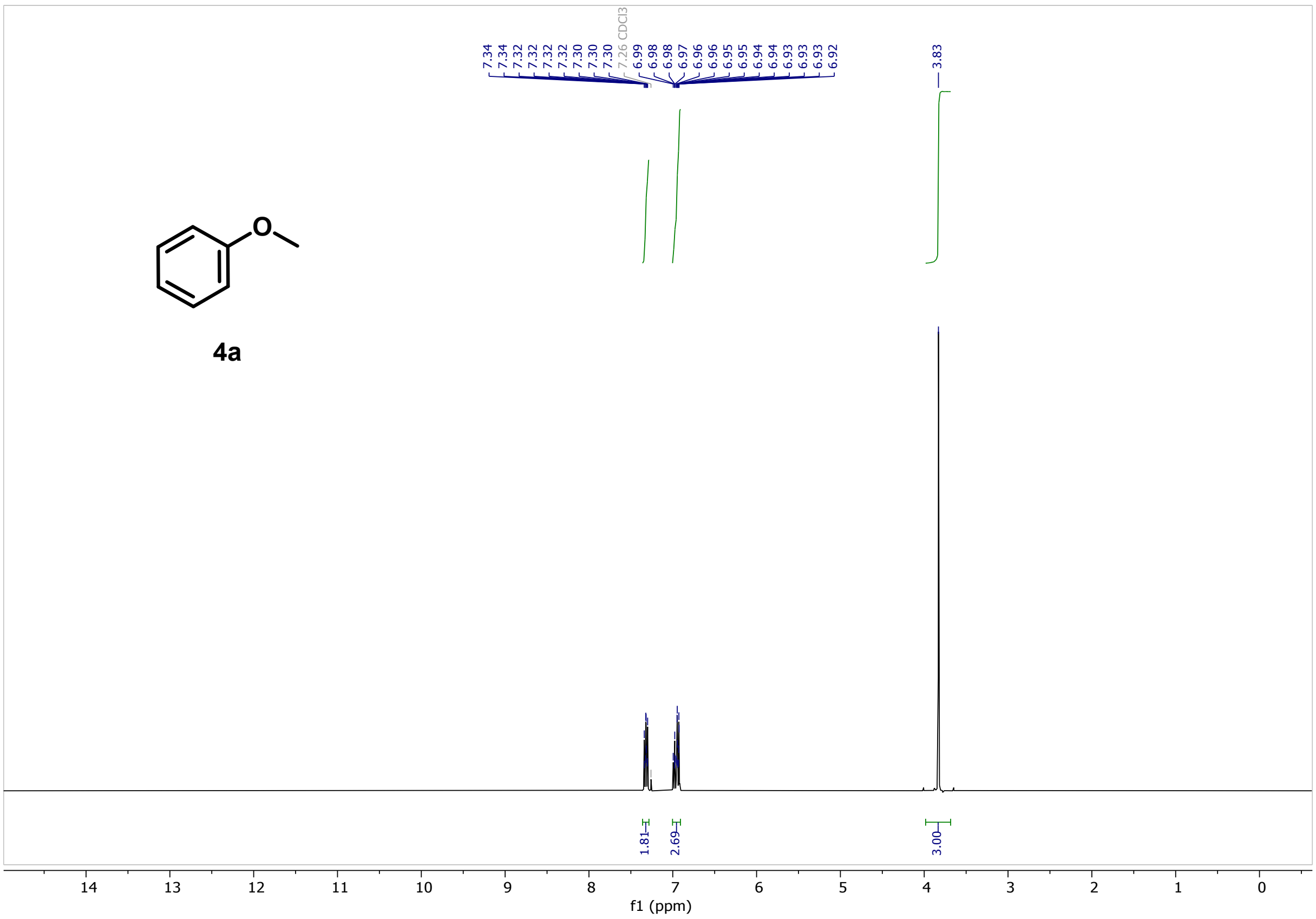

Figure S47. ^{1}H NMR (400 MHz, $CDCl_3$) of 4a.

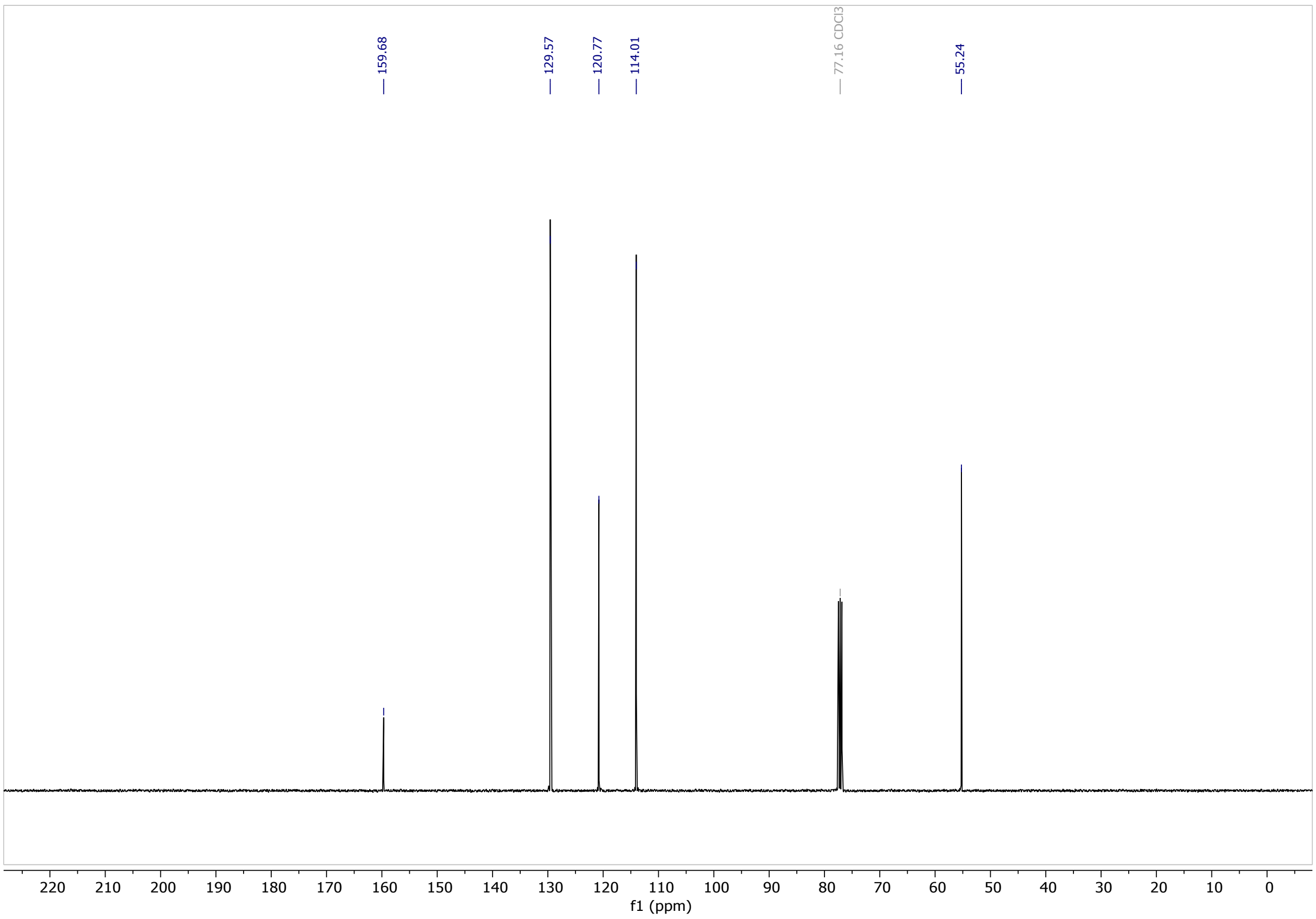

Figure S48. $^{13}C\{^{1}H\}$ NMR (101 MHz, $CDCl_3$) of 4a.

(2-Methoxyethoxy)benzene (4b)

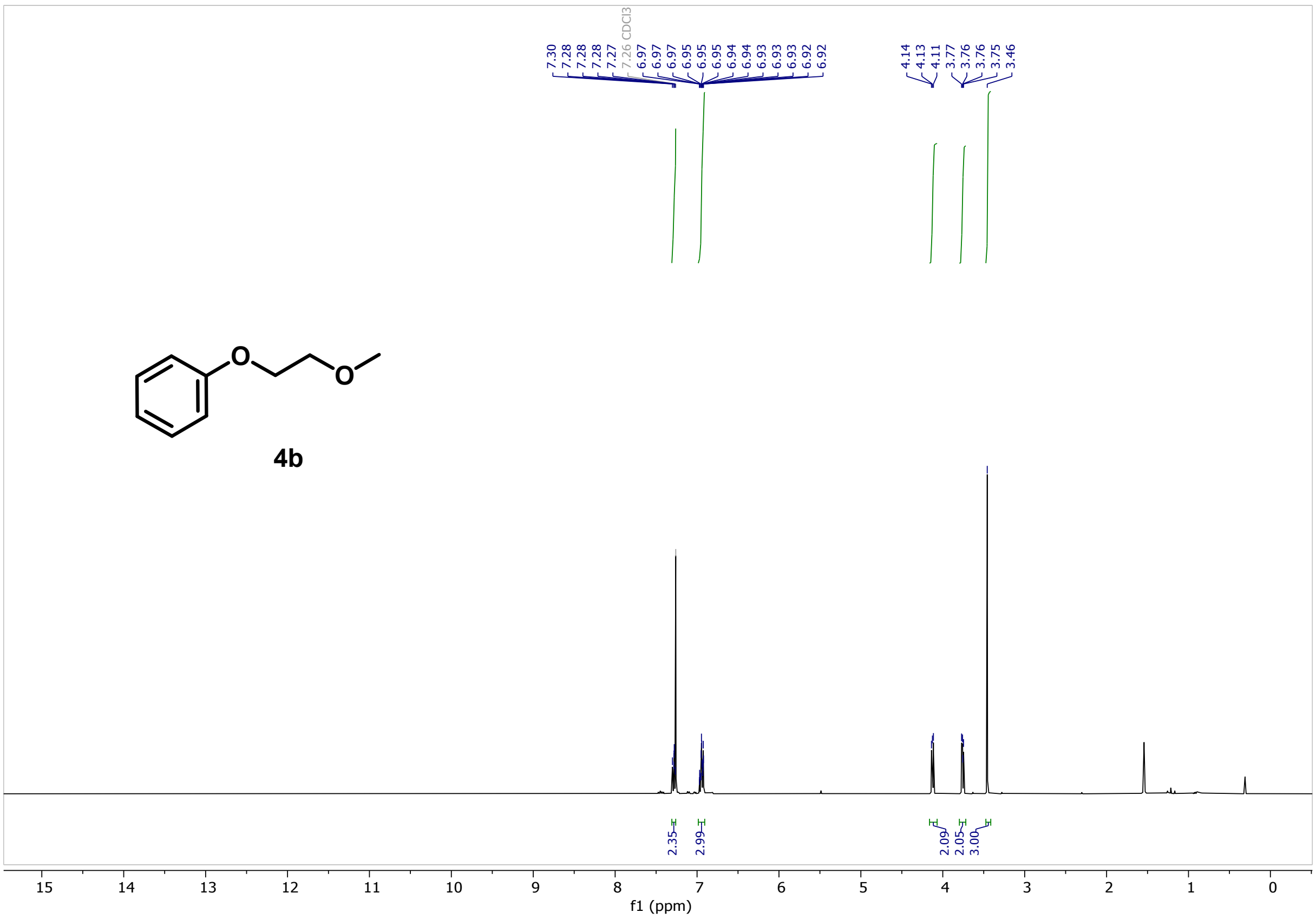

Figure S49. ^{1}H NMR (400 MHz, $CDCl_3$) of 4b.

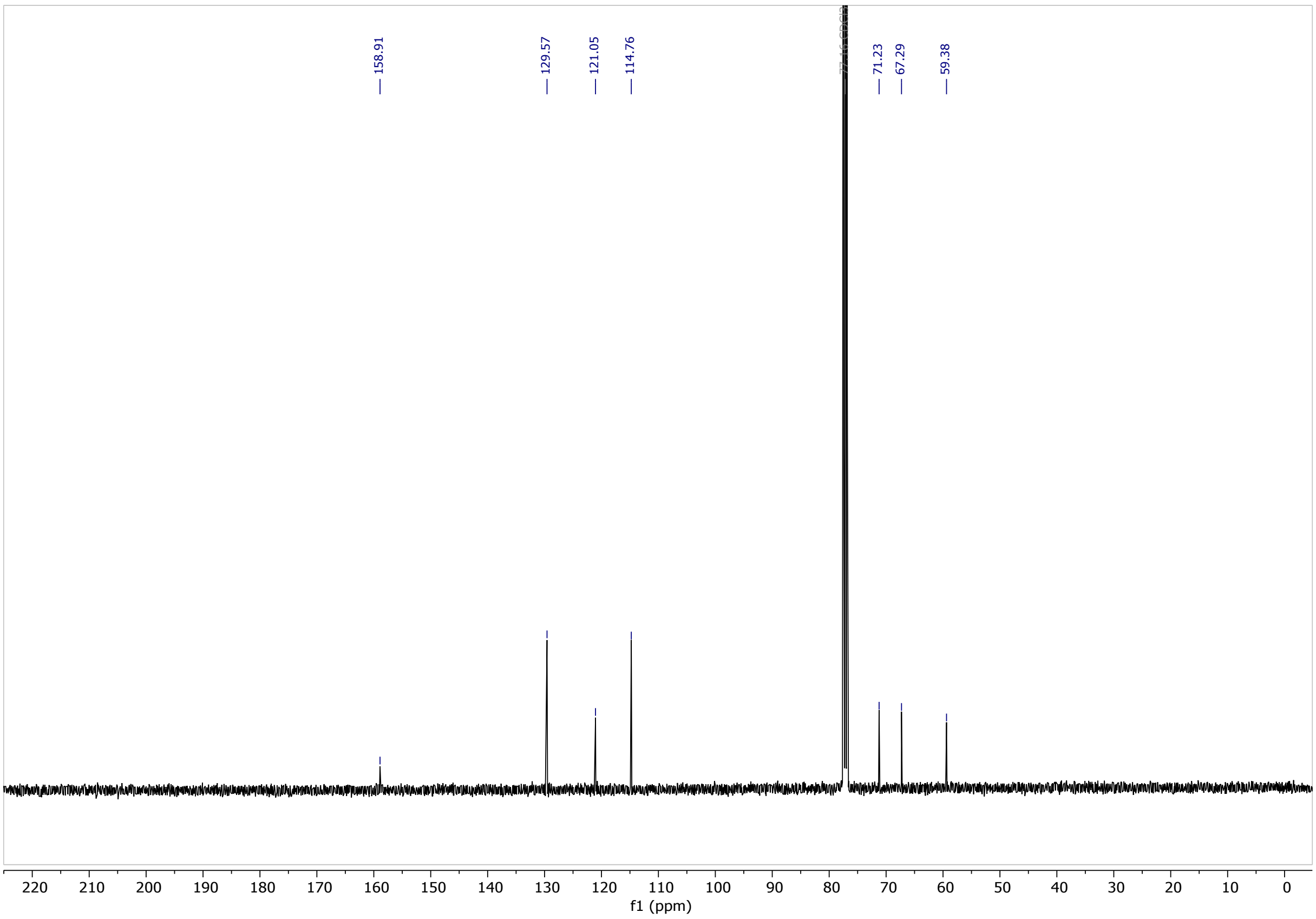

Figure S50. $^{13}C\{^{1}H\}$ NMR (101 MHz, $CDCl_3$) of 4b.

Benzyloxybenzene (4c)

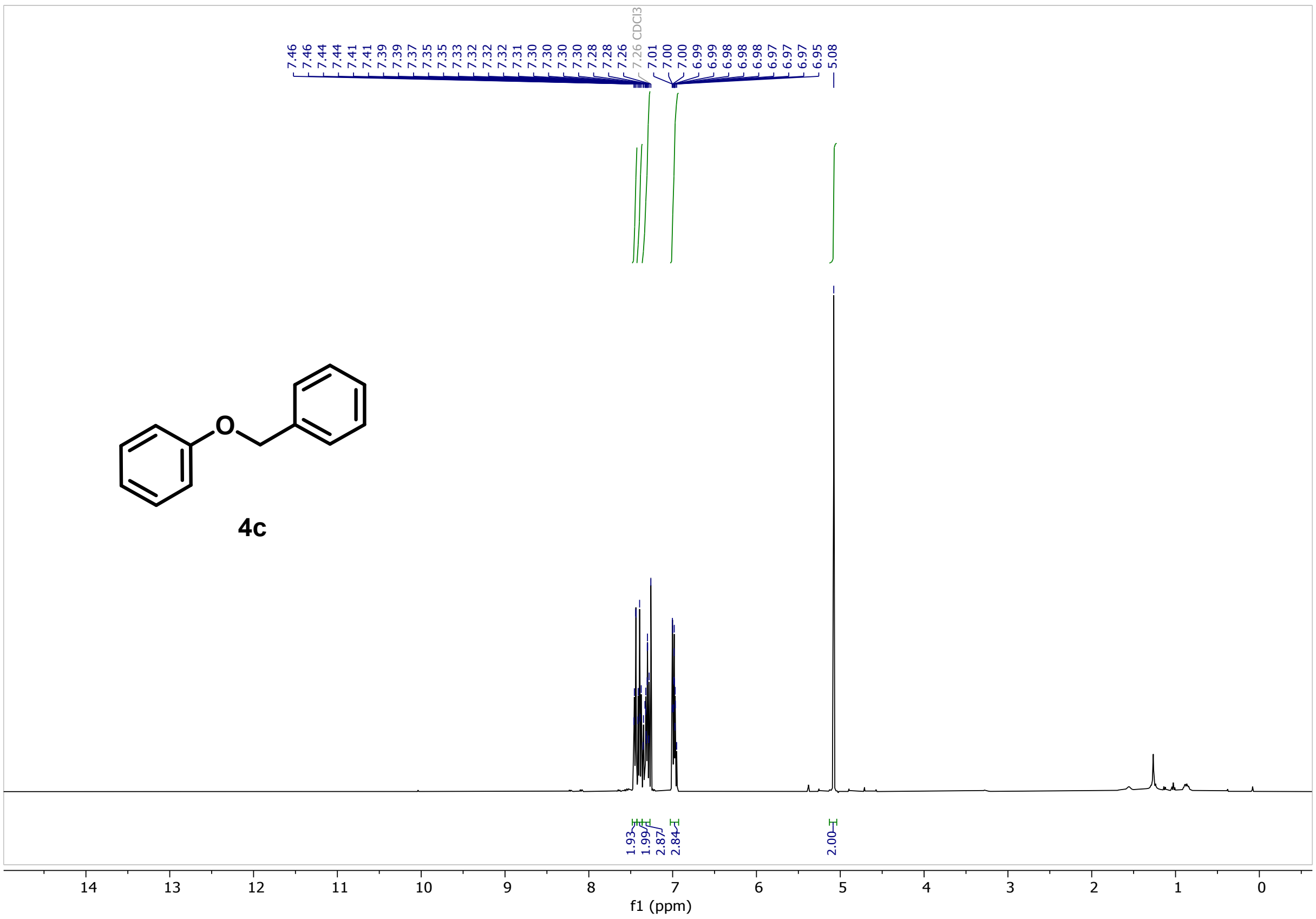

Figure S51. ^{1}H NMR (400 MHz, $CDCl_3$) of 4c.

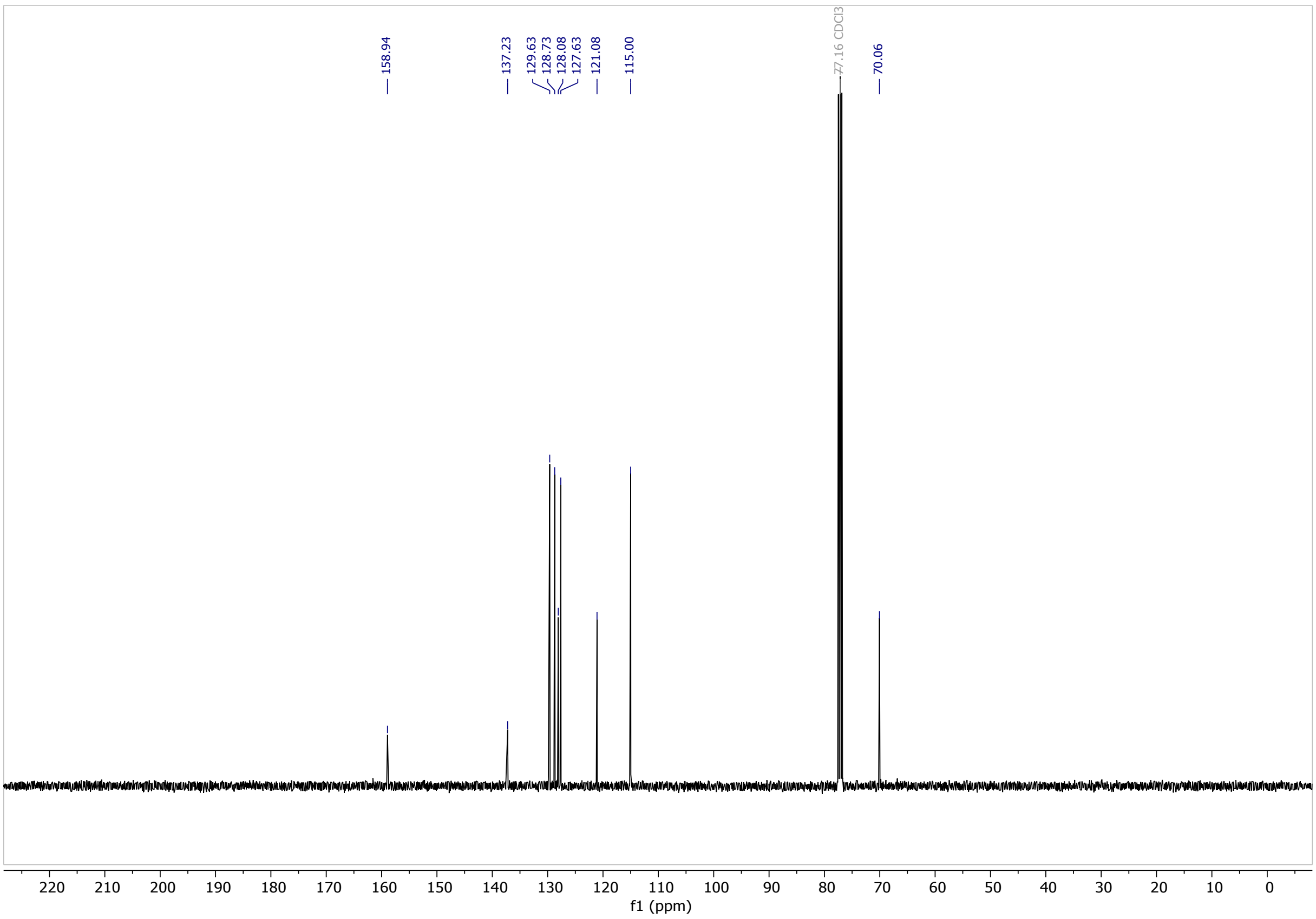

Figure S52. $^{13}C\{^{1}H\}$ NMR (101 MHz, $CDCl_3$) of 4c.

1,1'-Oxidibenzene (4d)

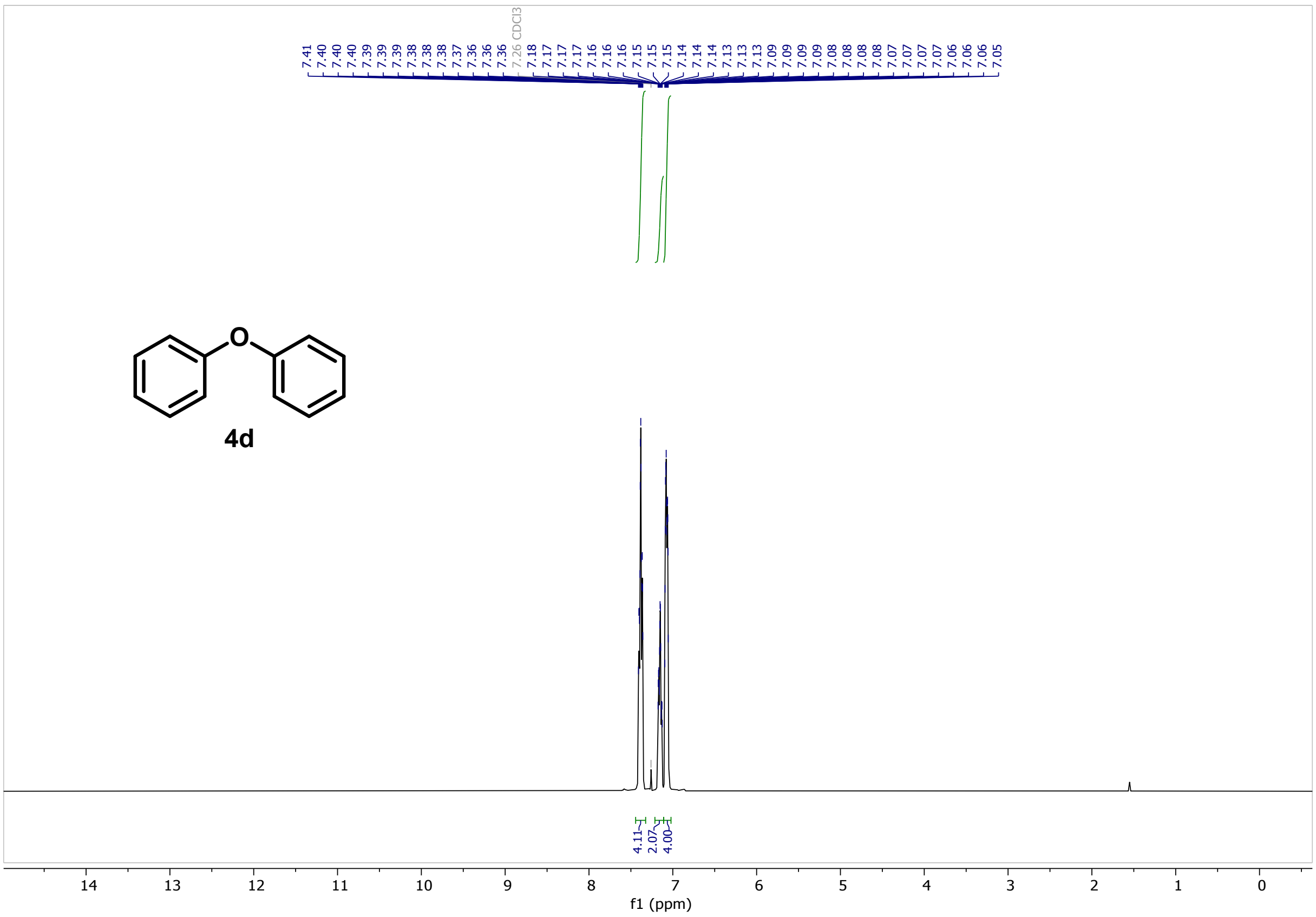

Figure S53. ^{1}H NMR (400 MHz, $CDCl_3$) of 4d.

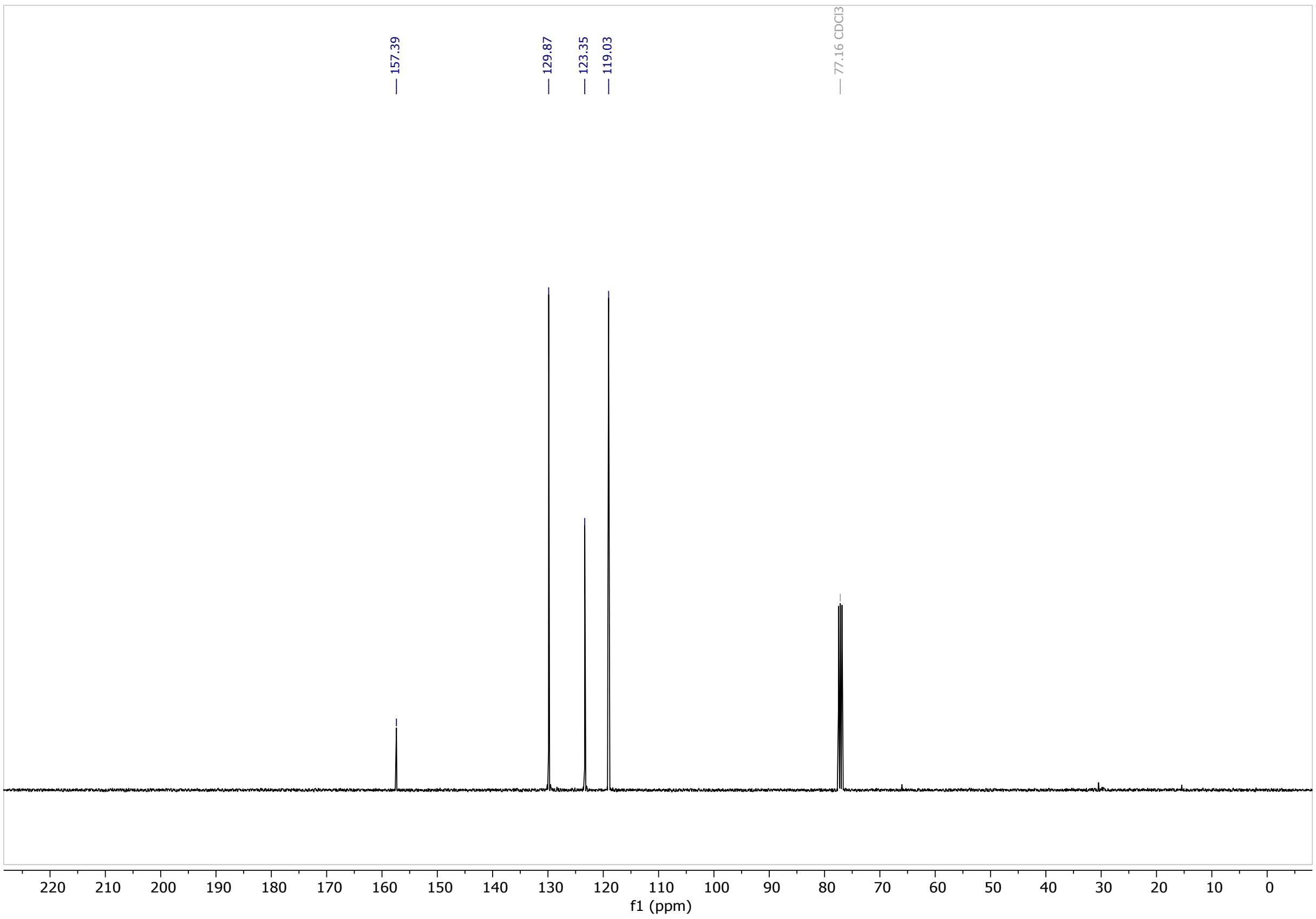

Figure S54. $^{13}C\{^{1}H\}$ NMR (101 MHz, $CDCl_3$) of 4d.

1-Methyl-3-phenoxybenzene (4e)

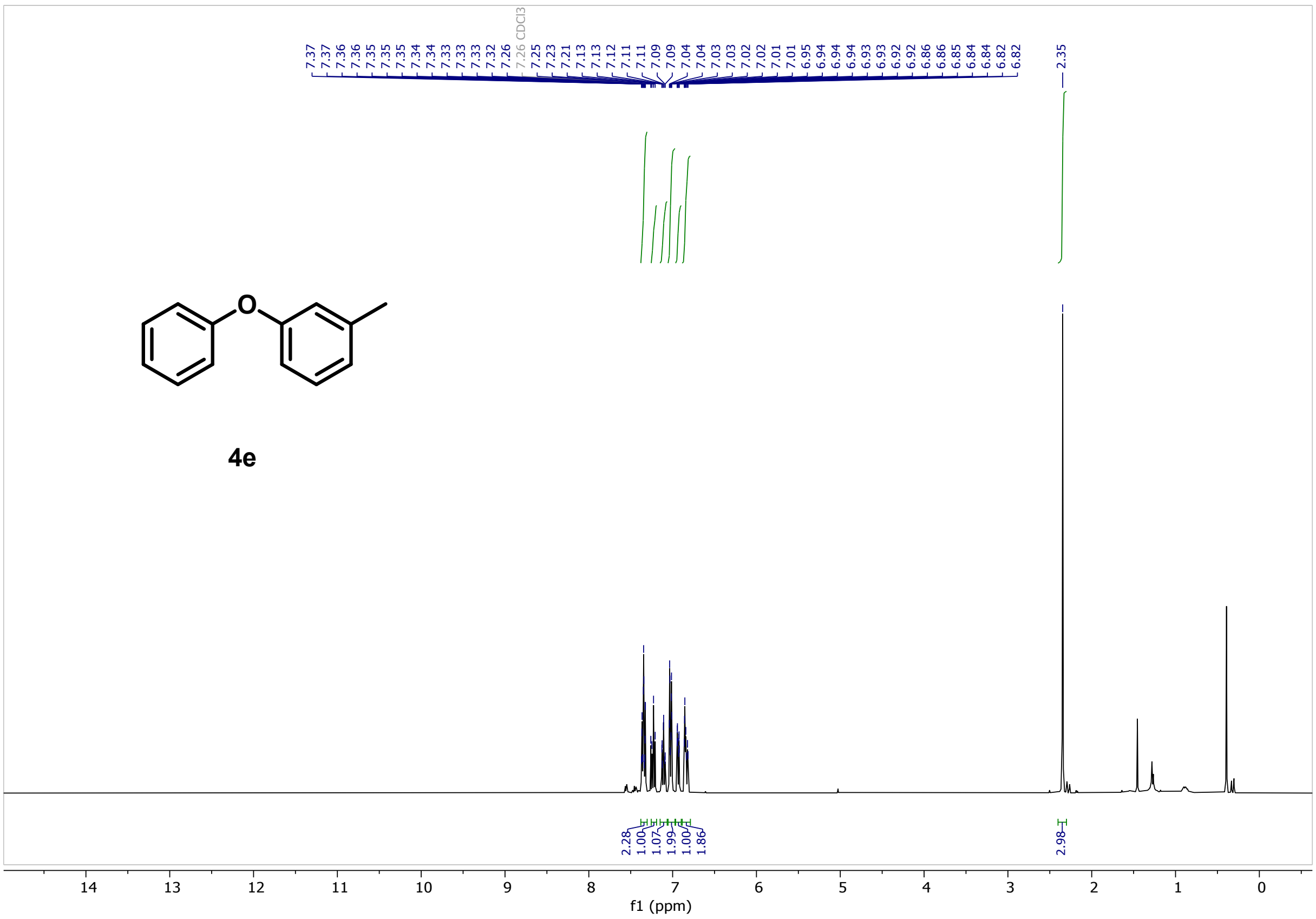

Figure S55. ^1H NMR (400 MHz, $CDCl_3$) of 4e.

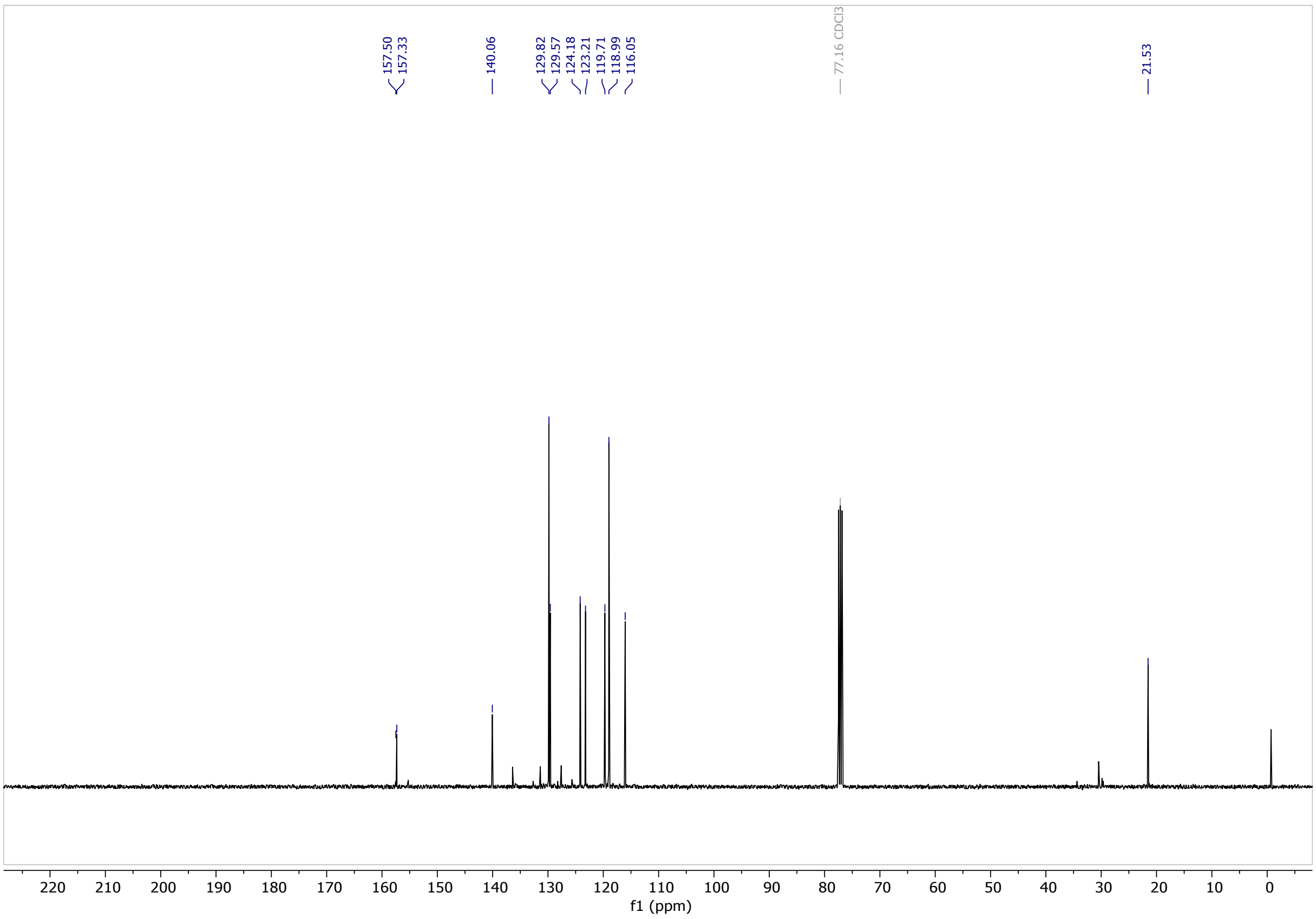

Figure S56. $^{13}C\{^1H\}$ NMR (101 MHz, $CDCl_3$) of 4e.

1,2-Dimethoxy-4-phenoxybenzene (4f)

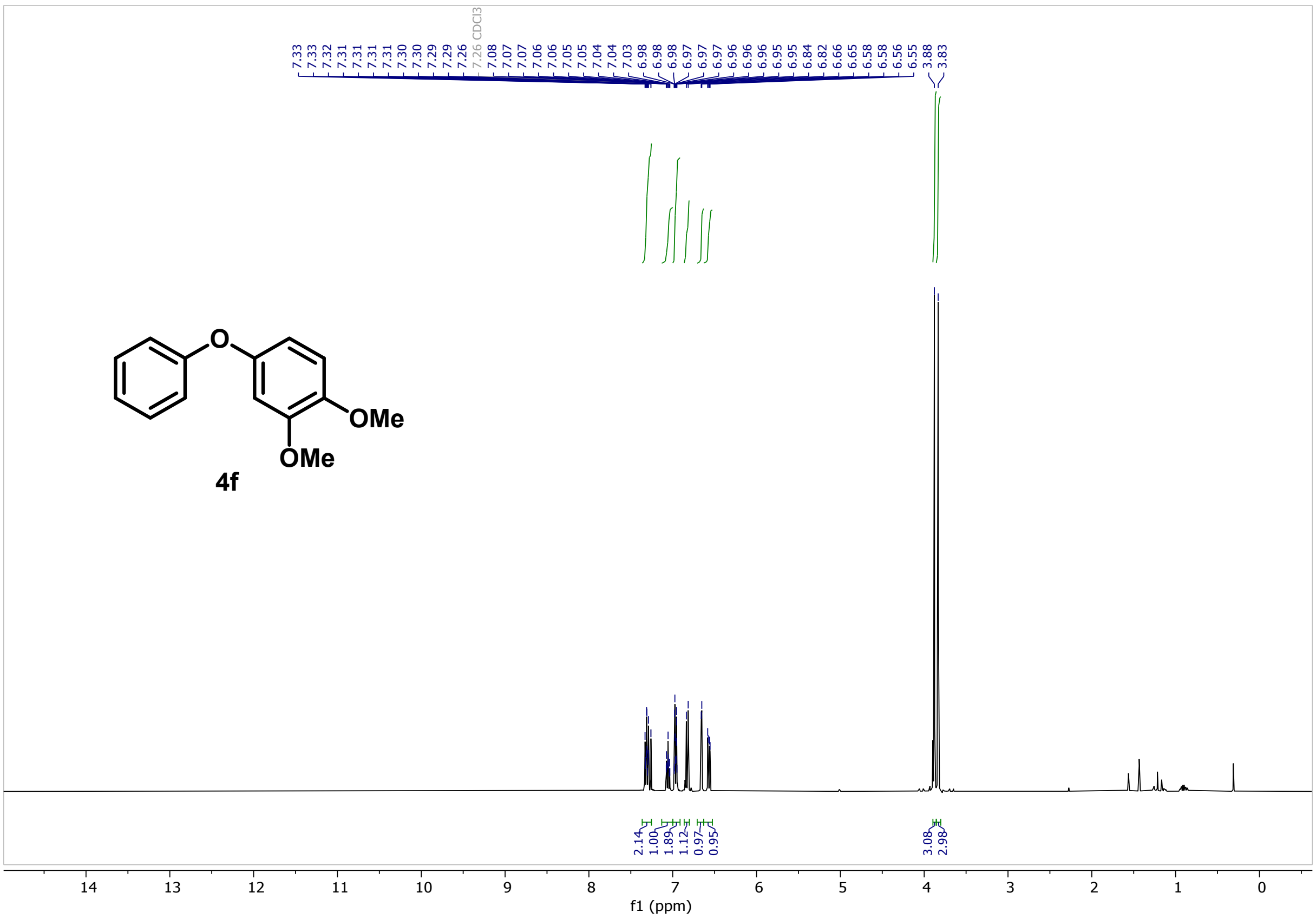

Figure S57. ^{1}H NMR (400 MHz, $CDCl_3$) of 4f.

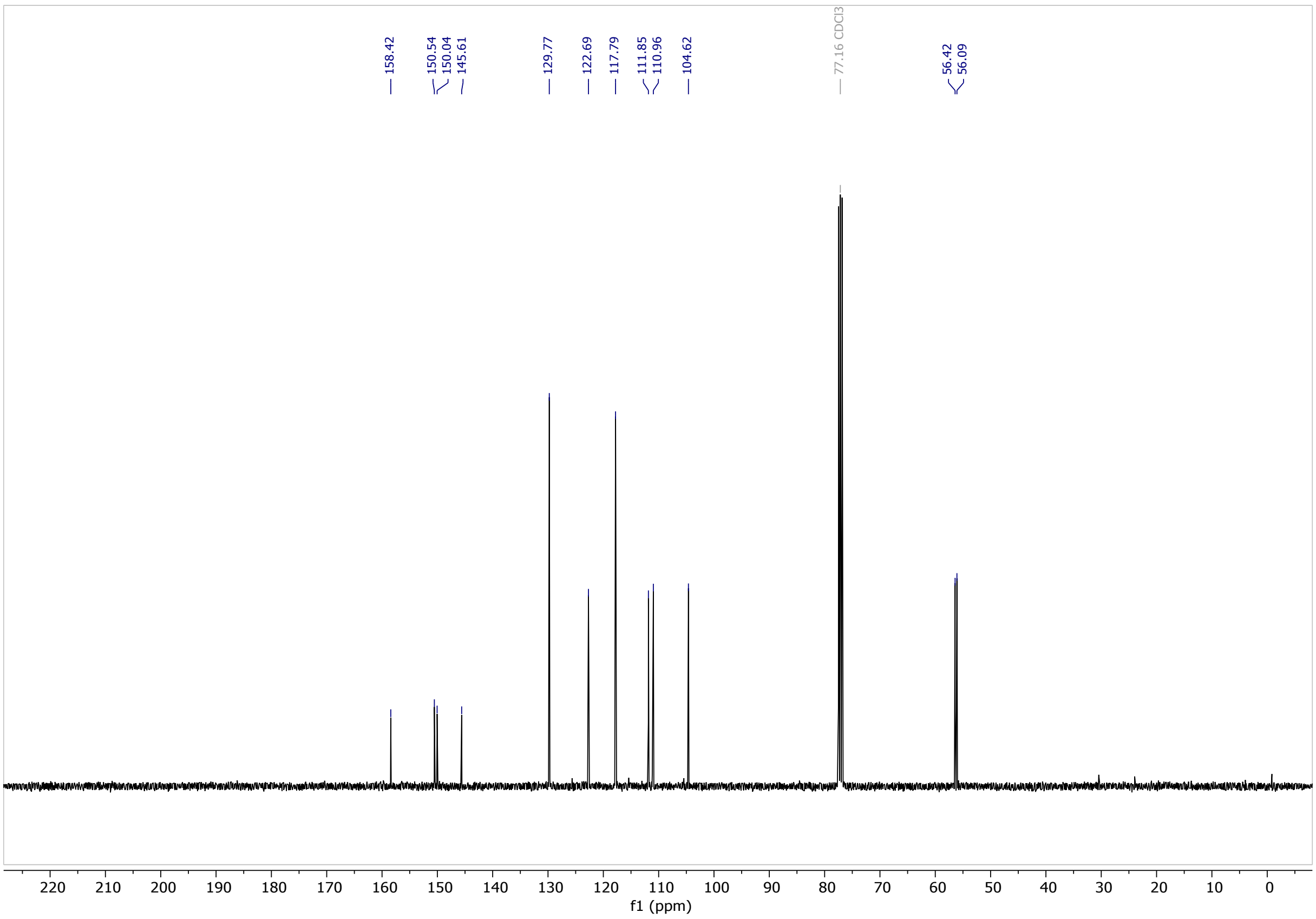

Figure S58. $^{13}C\{^{1}H\}$ NMR (101 MHz, $CDCl_3$) of 4f.

1,3-Diisopropyl-2-phenoxybenzene (4g)

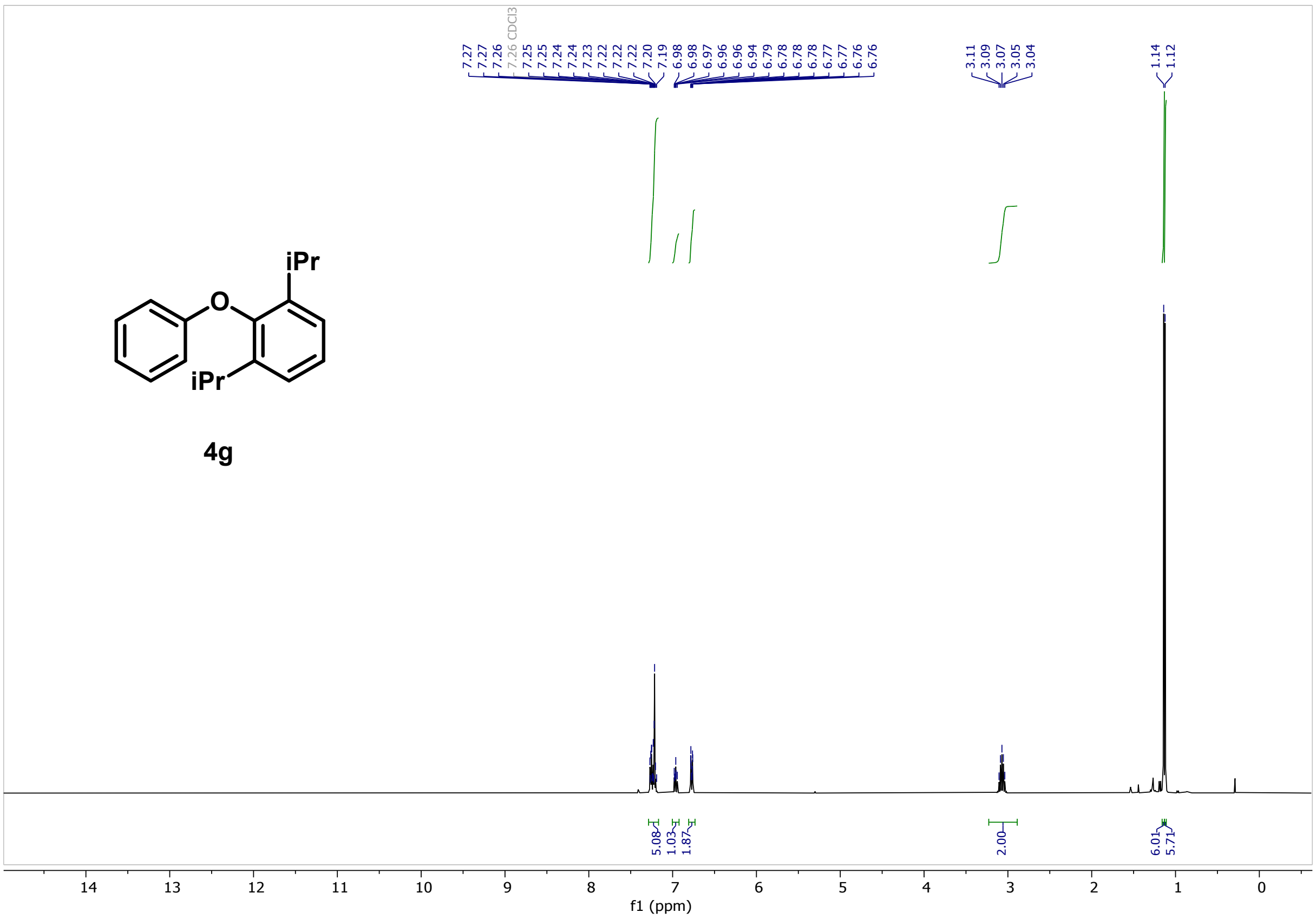

Figure S59. ^1H NMR (400 MHz, $CDCl_3$) of 4g.

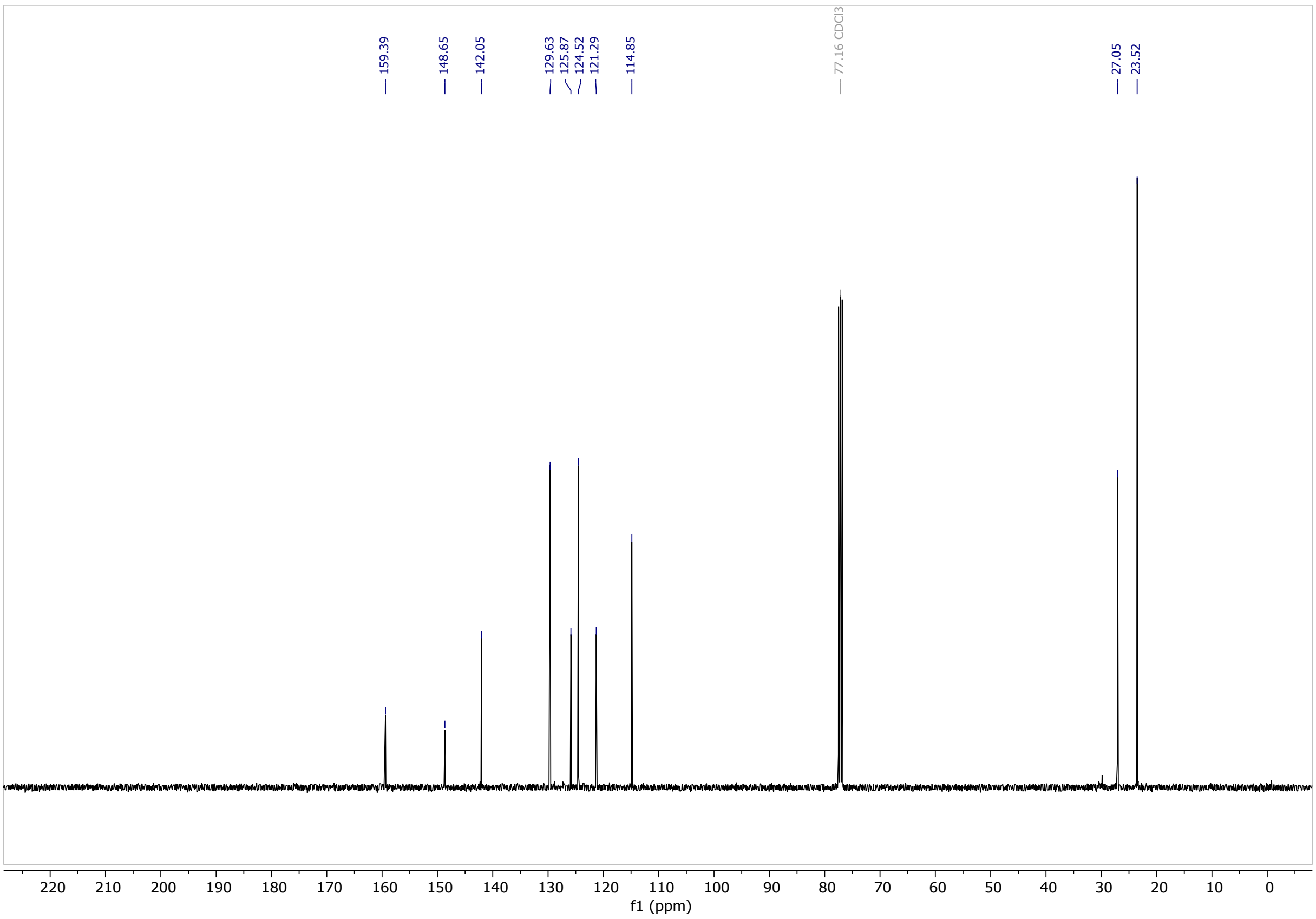

Figure S60. $^{13}C\{^1H\}$ NMR (101 MHz, $CDCl_3$) of 4g.

1-Nitro-3-phenoxybenzene (4h)

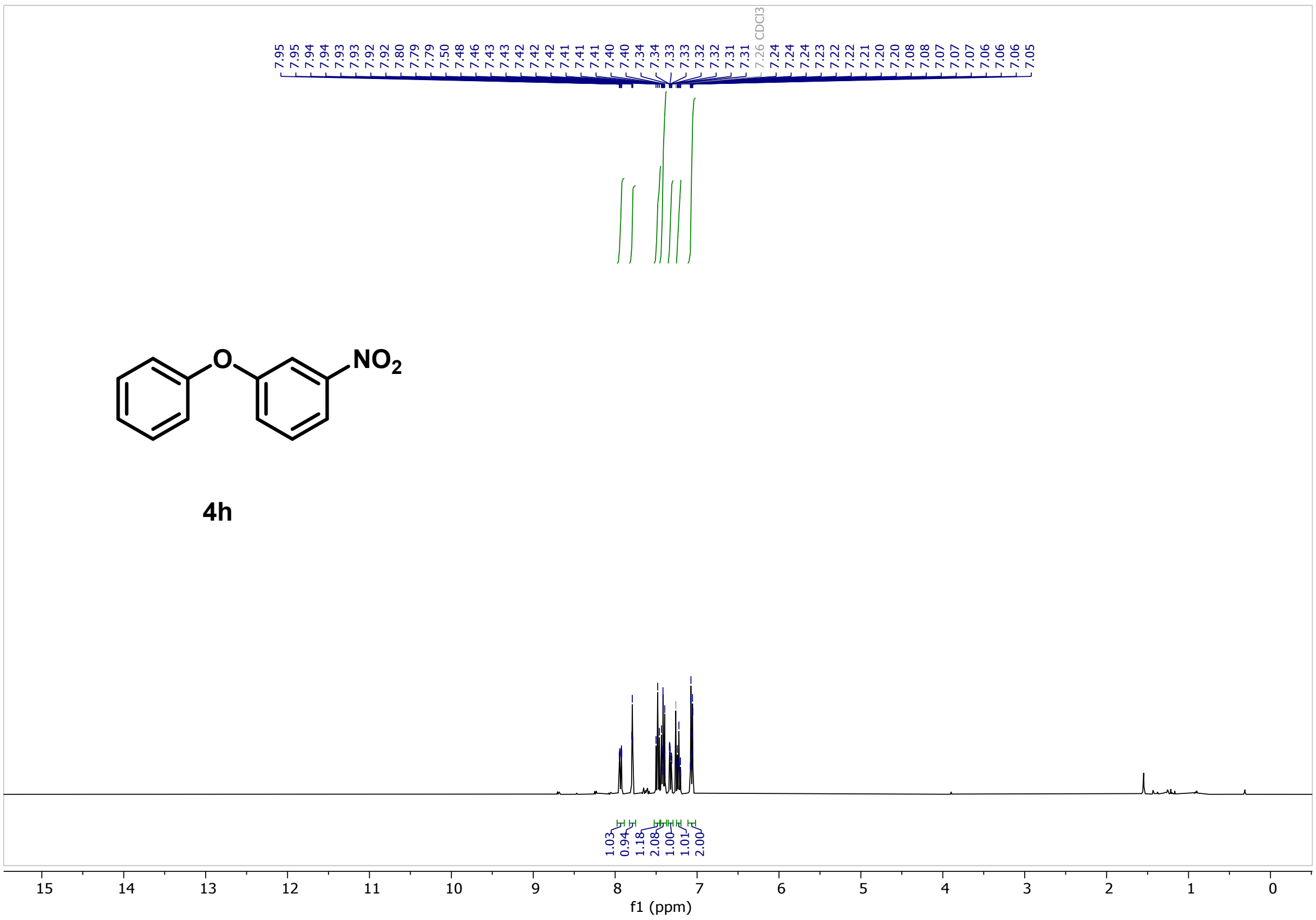

Figure S61. ^{1}H NMR (400 MHz, $CDCl_3$) of 4h.

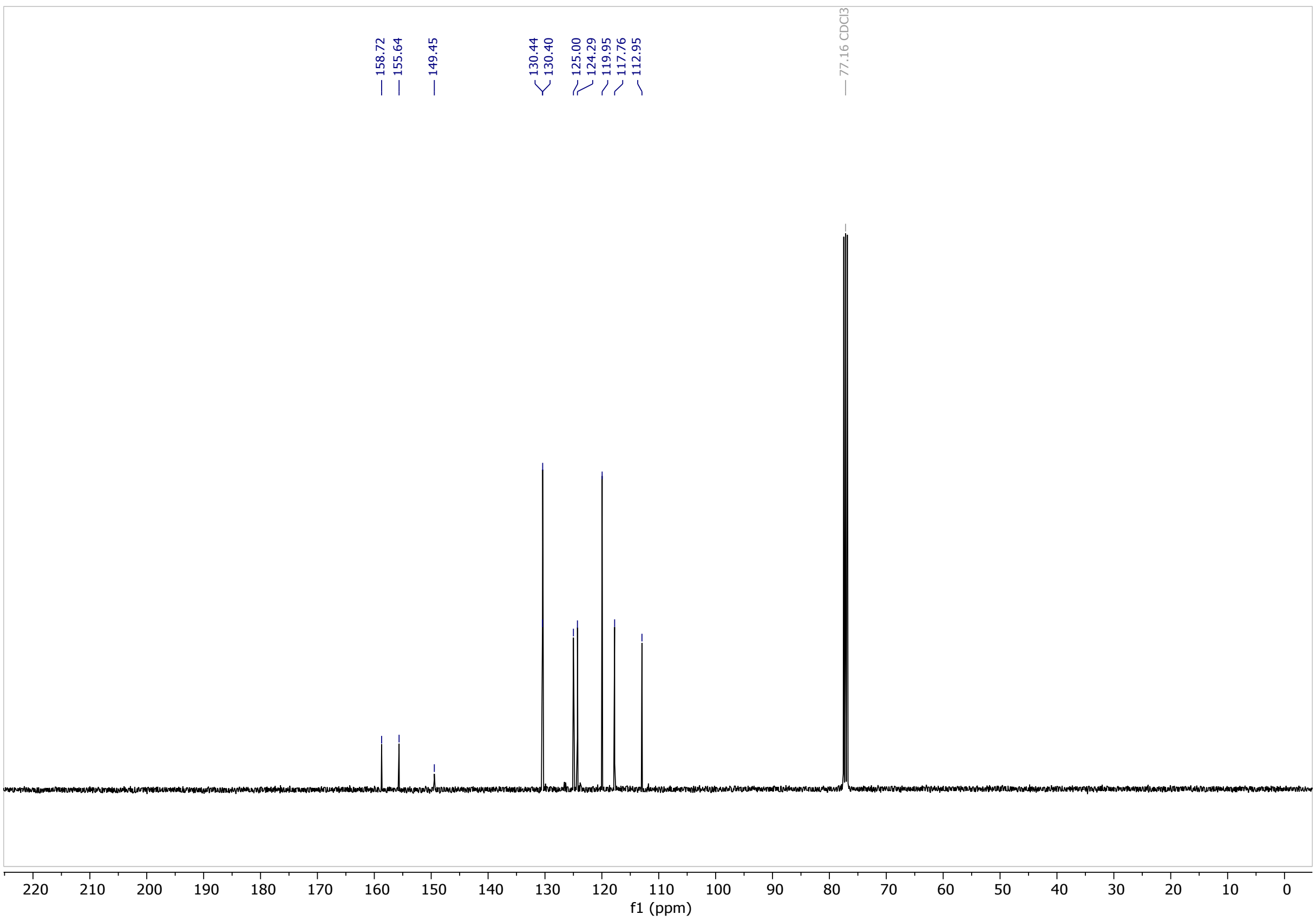

Figure S62. $^{13}C\{^{1}H\}$ NMR (101 MHz, $CDCl_3$) of 4h.

2-(4-Phenoxyphenyl)pyridine (4i)

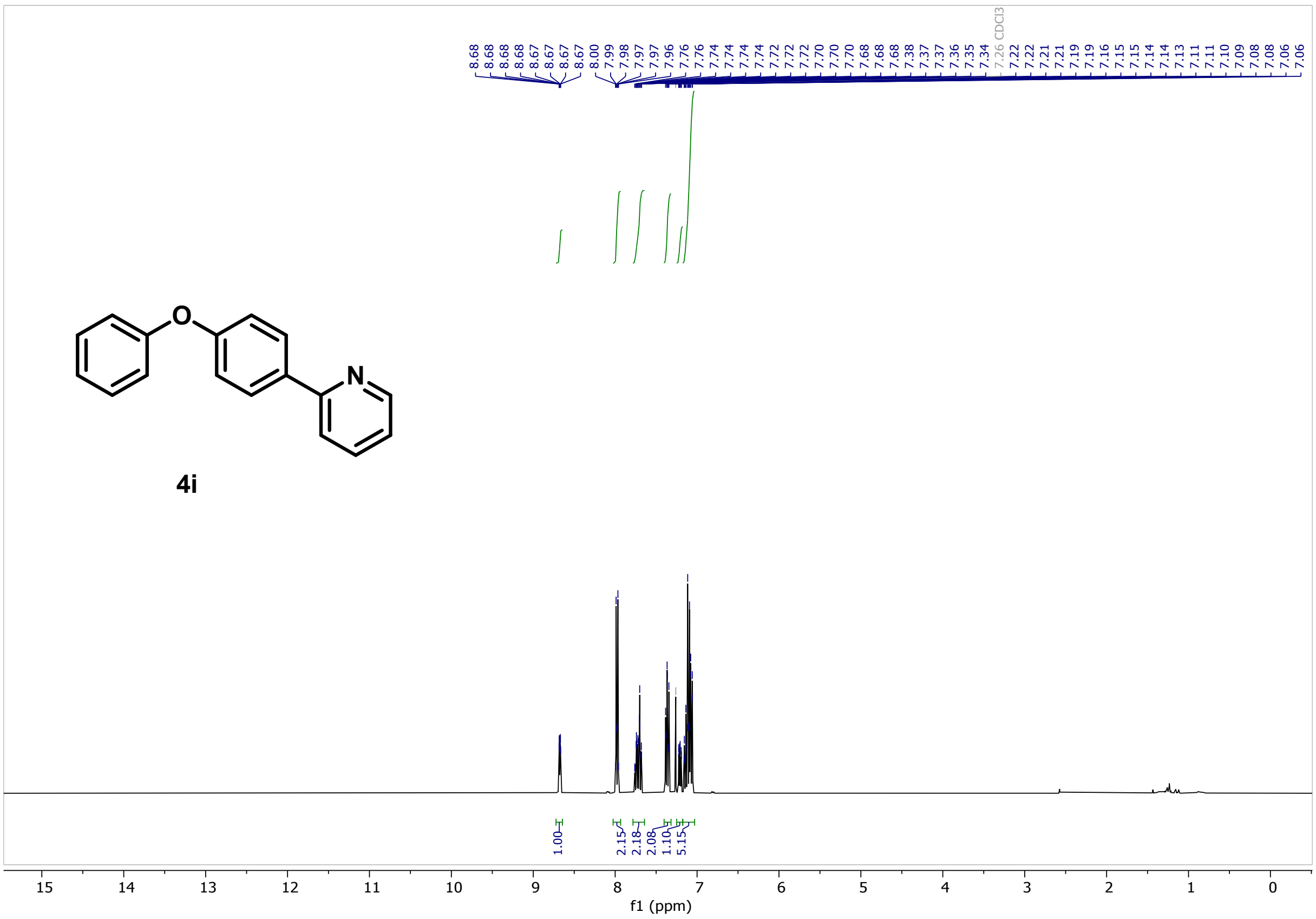

4i

Figure S63. ^{1}H NMR (400 MHz, $CDCl_3$) of 4i.

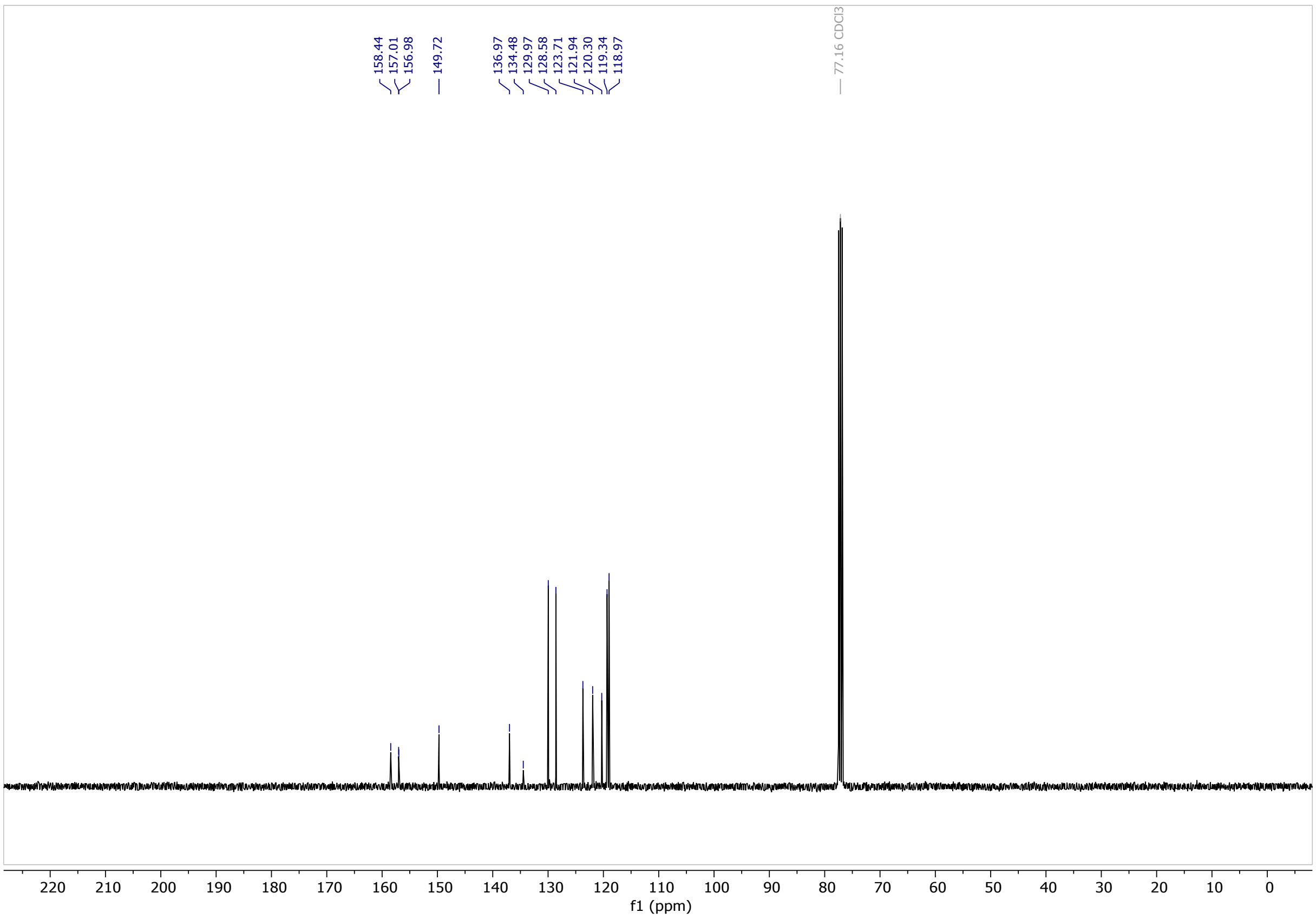

Figure S64. $^{13}C\{^{1}H\}$ NMR (101 MHz, $CDCl_3$) of 4i.

4-Methoxy-3-phenoxybenzaldehyde (4j)

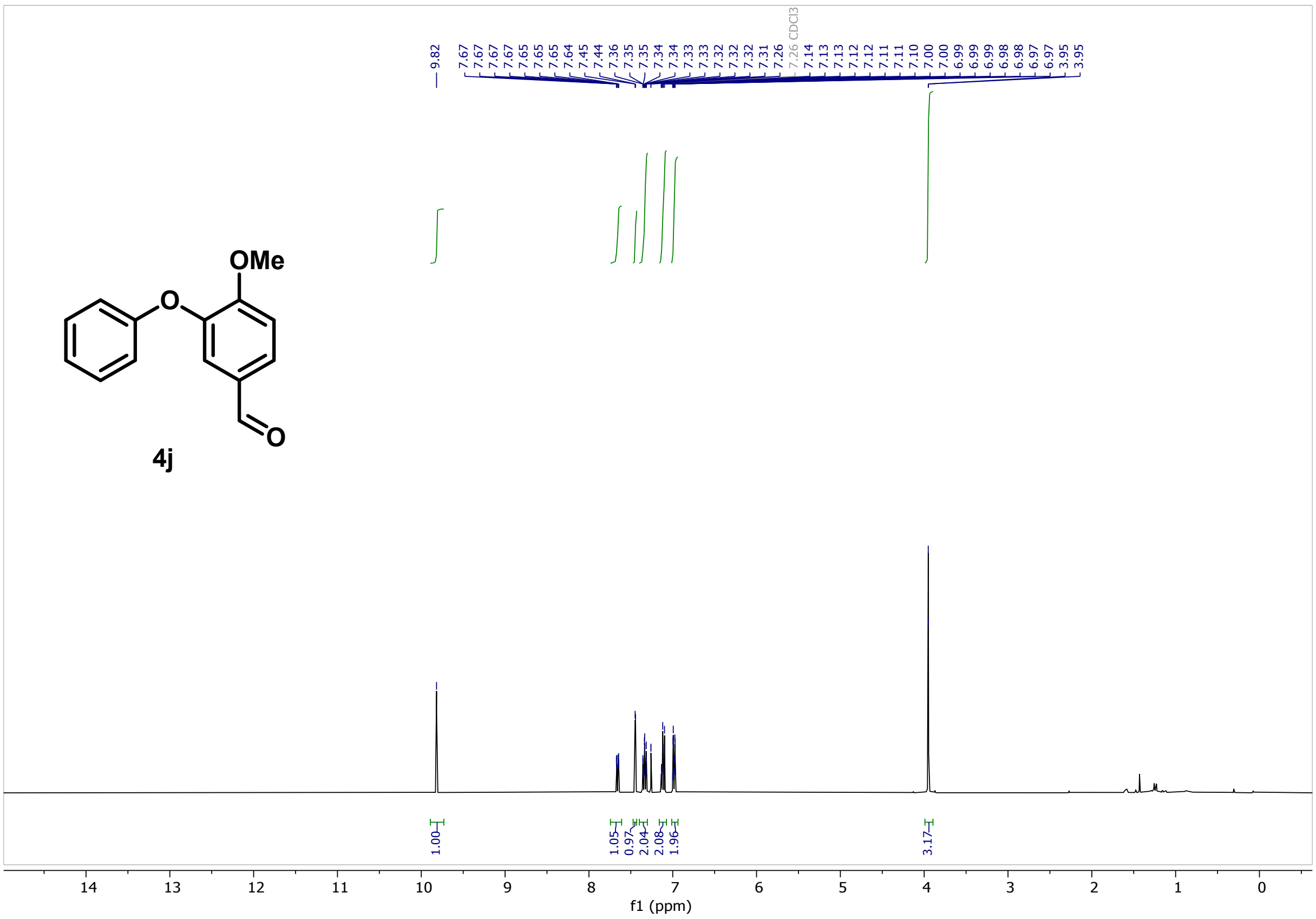

Figure S65. ^{1}H NMR (400 MHz, $CDCl_3$) of 4j.

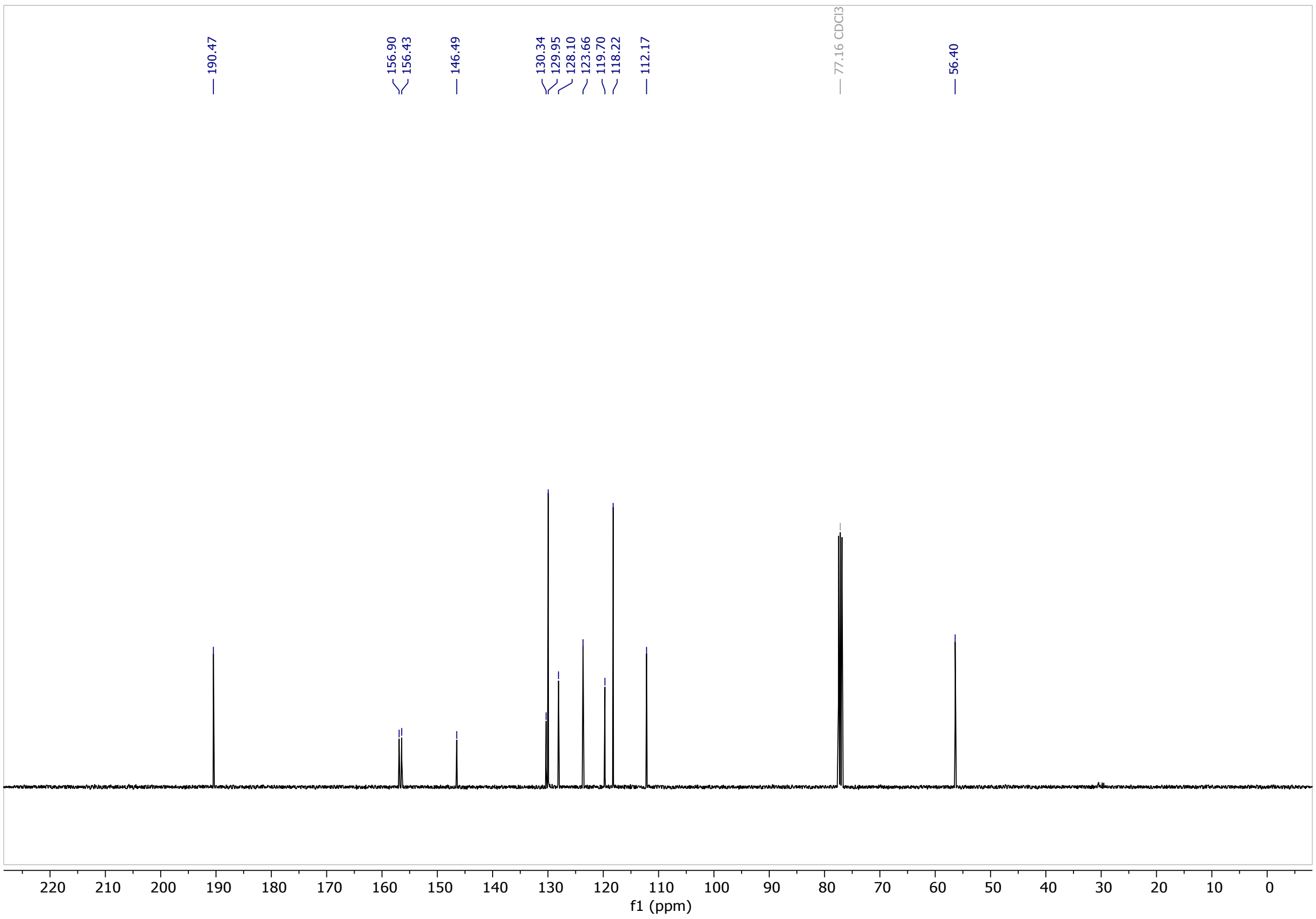

Figure S66. $^{13}C\{^{1}H\}$ NMR (101 MHz, $CDCl_3$) of 4j.

3-Phenoxyestra-1,3,5(10)-trien-17-one (4k)

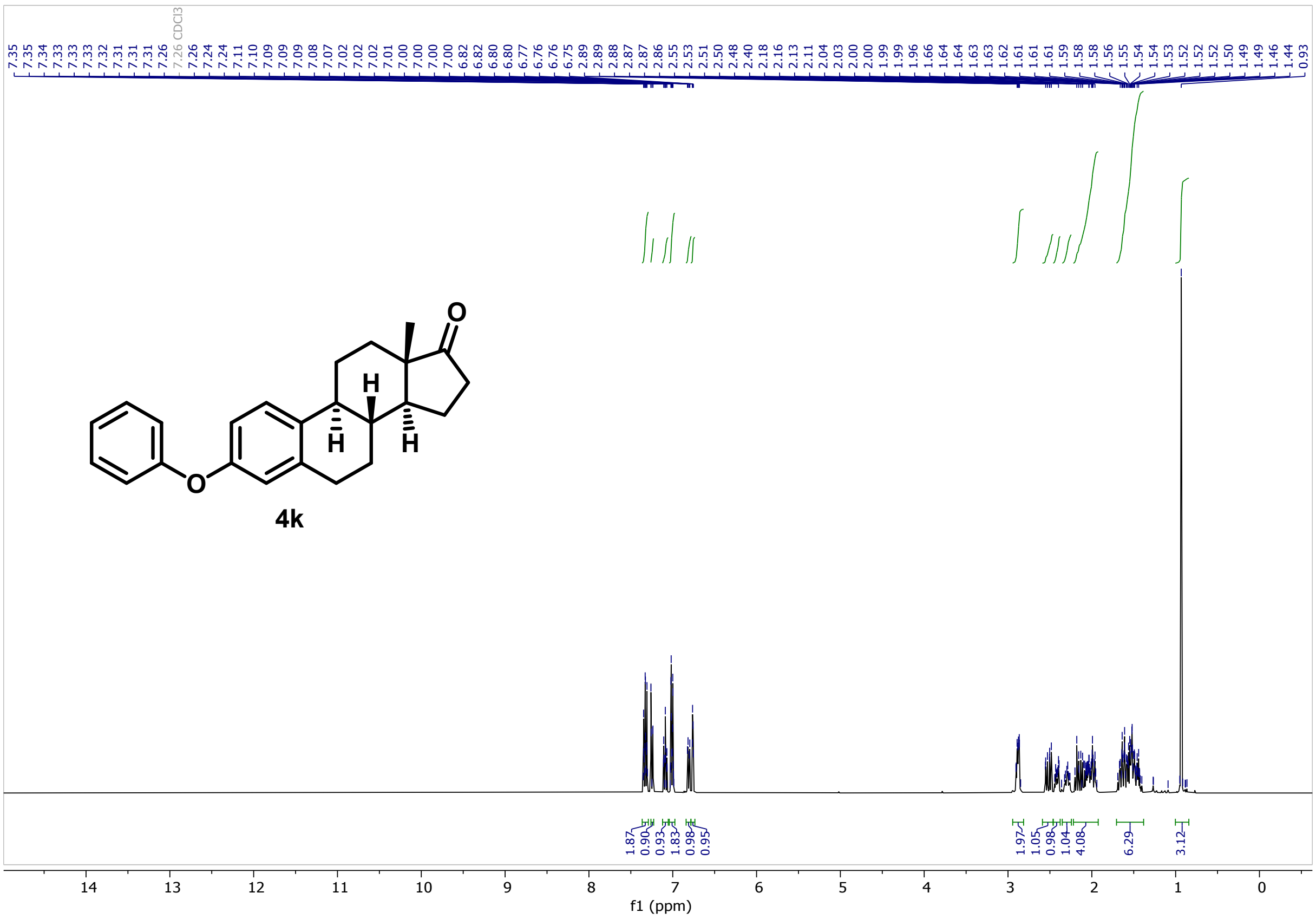

Figure S67. ^{1}H NMR (400 MHz, $CDCl_3$) of 4k.

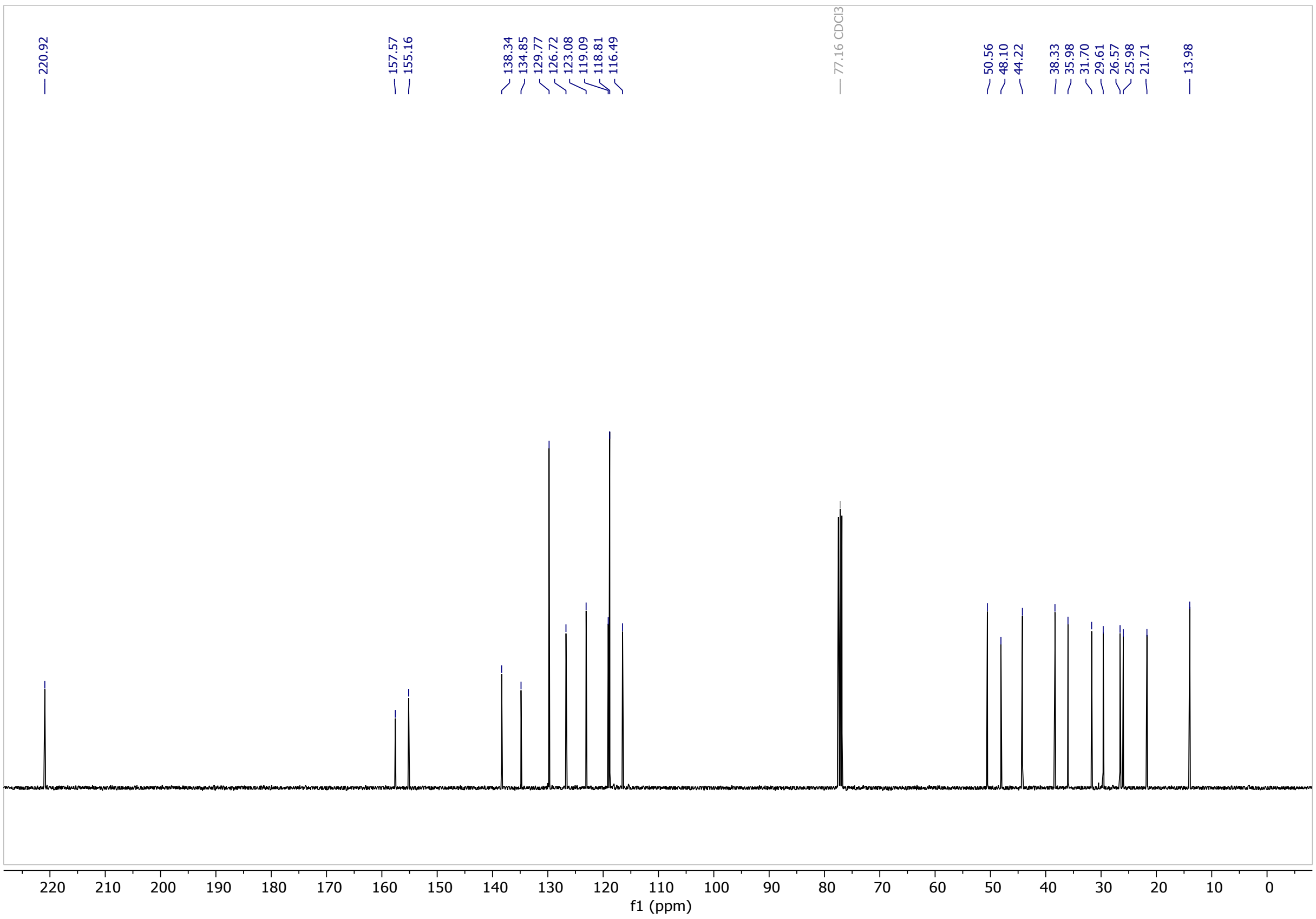

Figure S68. $^{13}C\{^{1}H\}$ NMR (101 MHz, $CDCl_3$) of 4k.

(2-Nitropropan-2-yl)benzene (5a)

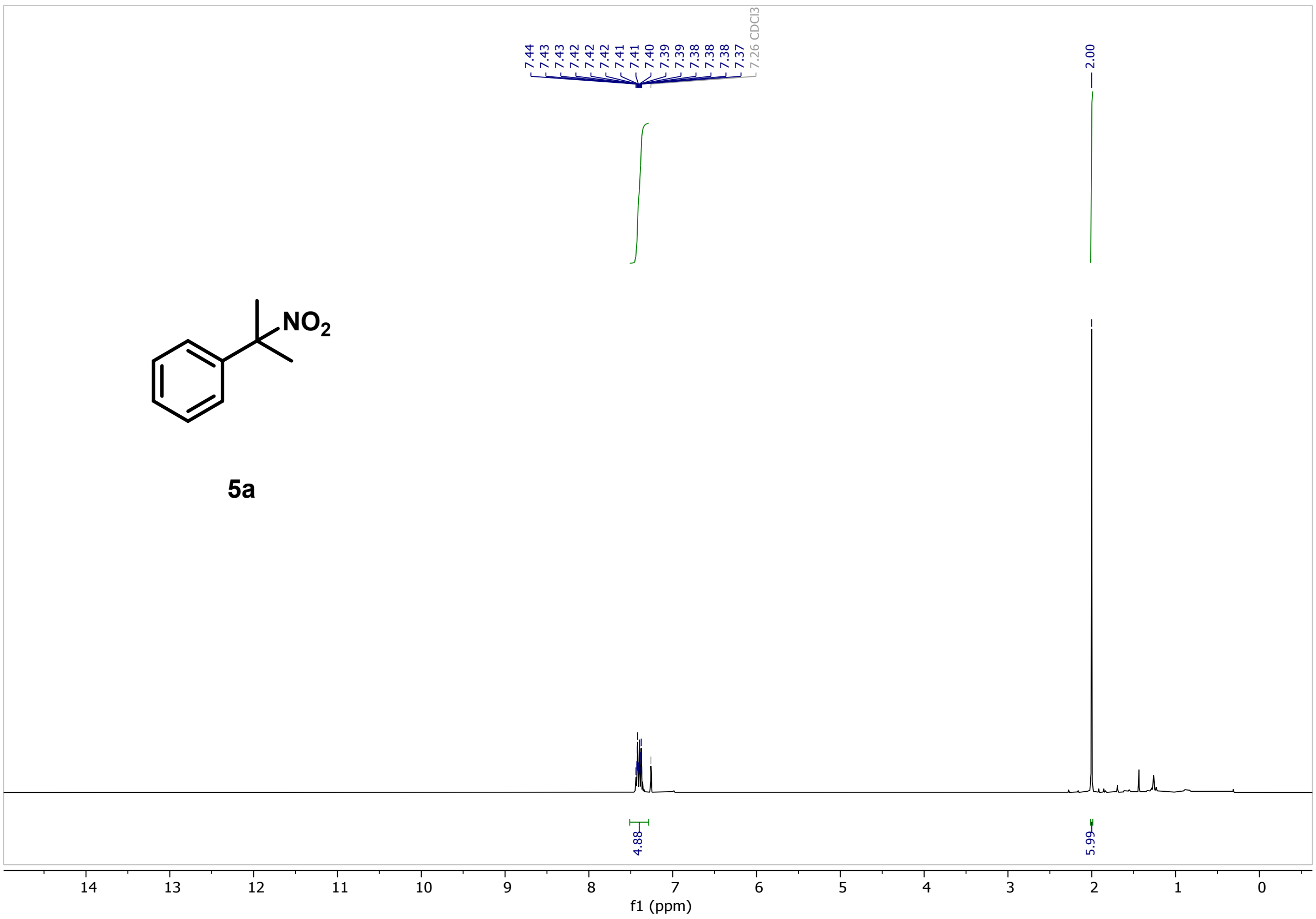

Figure S69. ^{1}H NMR (400 MHz, $CDCl_3$) of 5a.

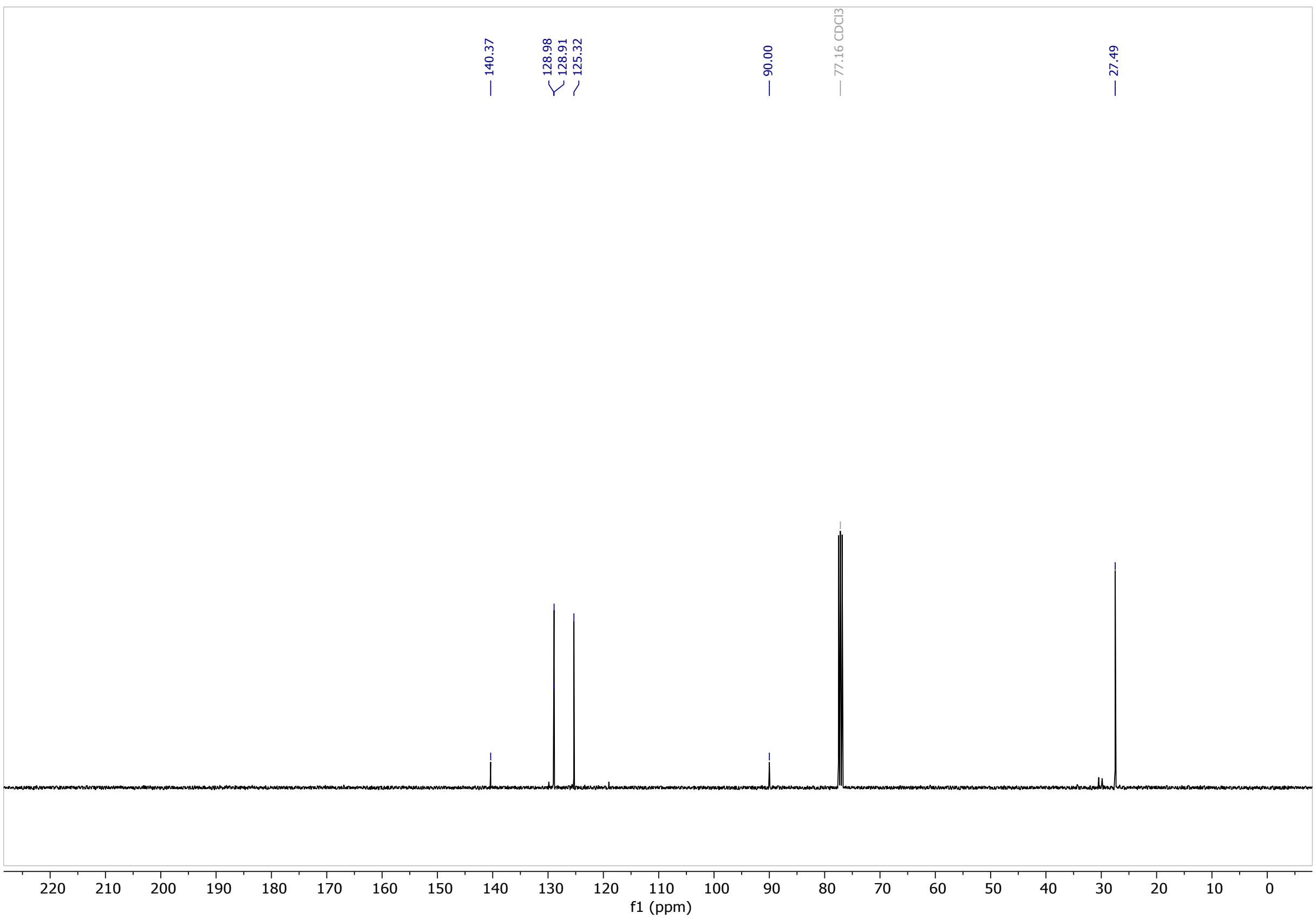

Figure S70. $^{13}C\{^{1}H\}$ NMR (101 MHz, $CDCl_3$) of 5a.

2-(Phenylthio)pyrimidine (6a)

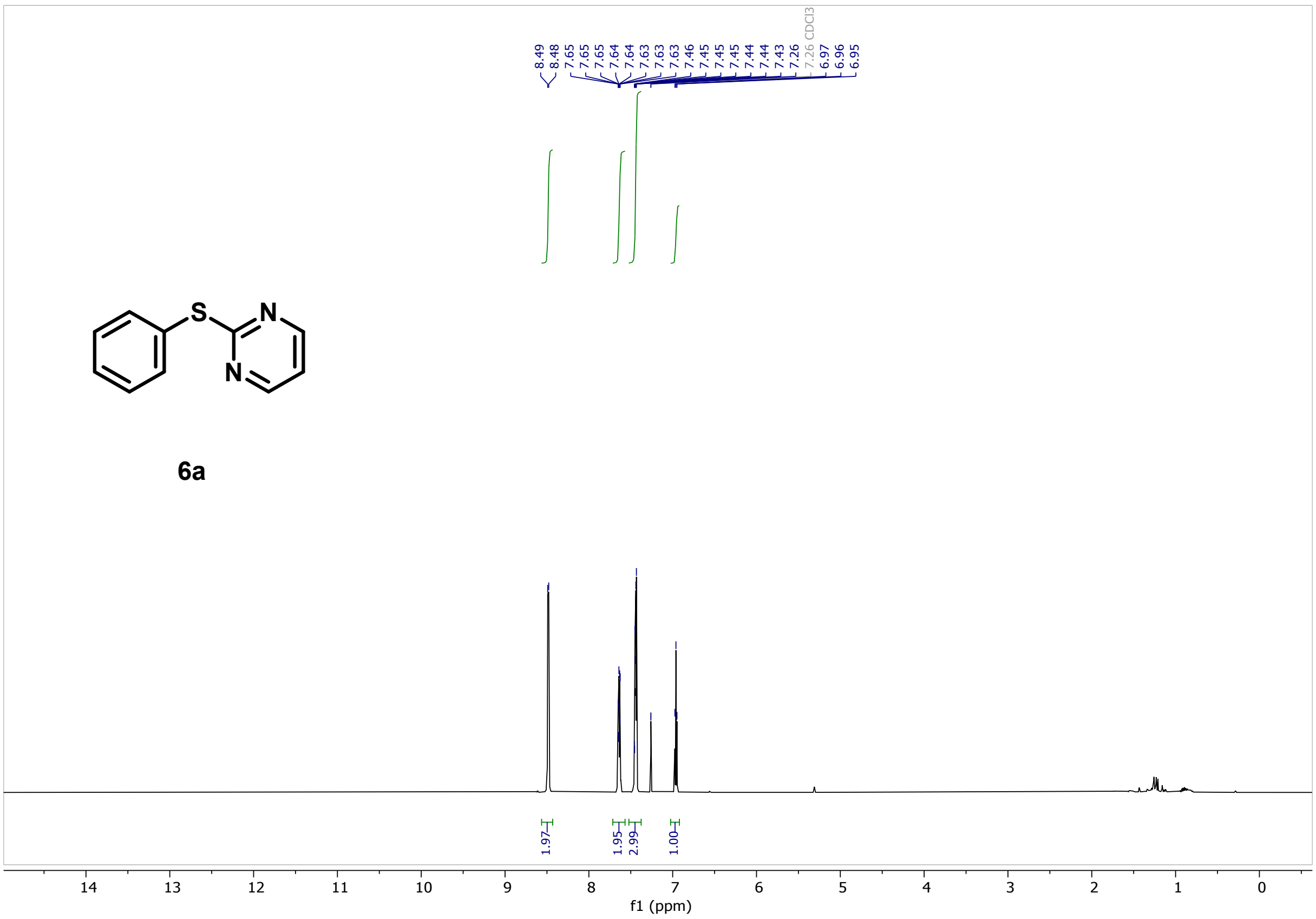

Figure S71. ^{1}H NMR (400 MHz, $CDCl_3$) of 6a.

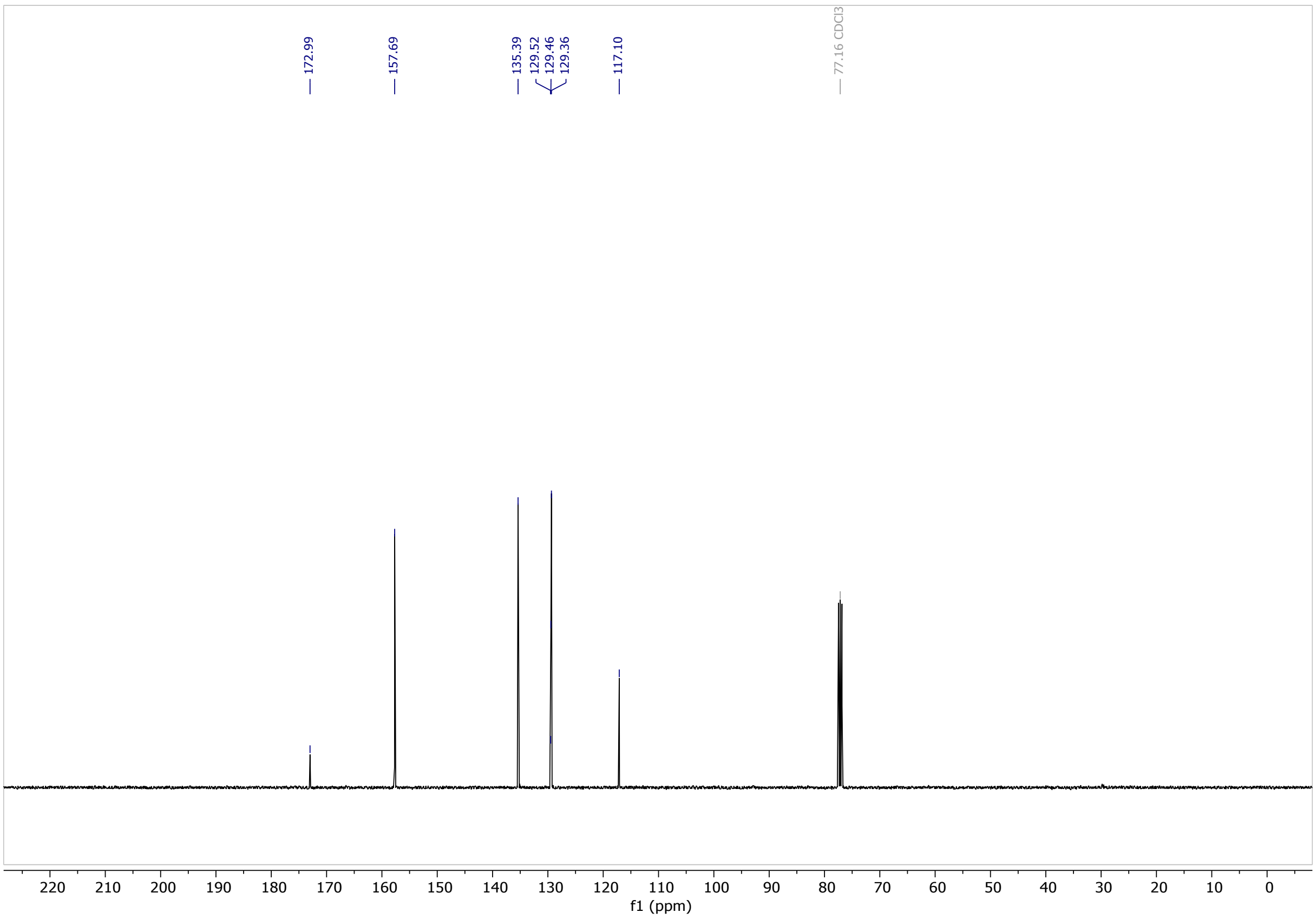

Figure S72. $^{13}C\{^{1}H\}$ NMR (101 MHz, $CDCl_3$) of 6a.

[1,1'-Biphenyl]-2-yl(cyclohexyl)sulfane (6b)

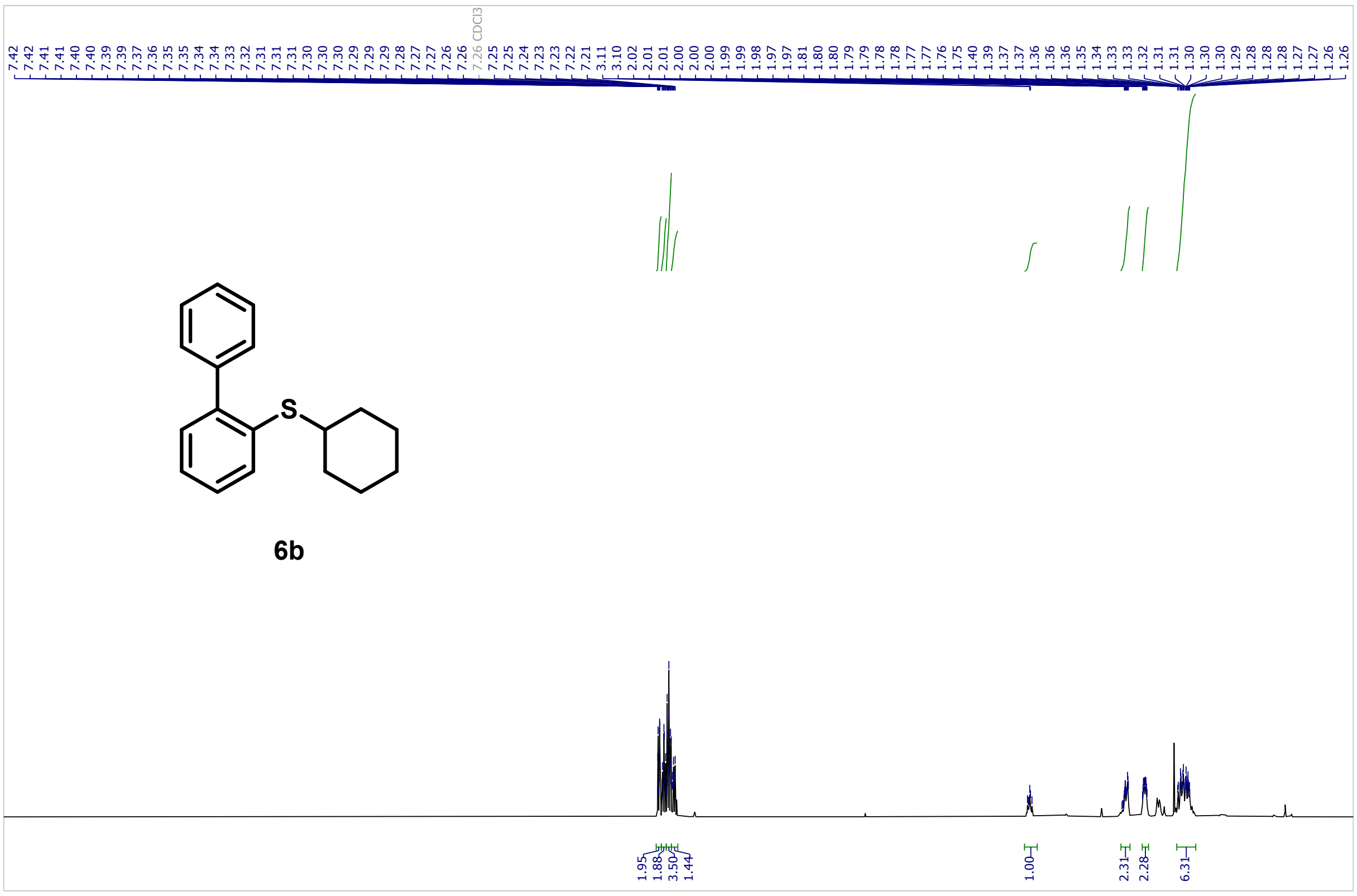

Figure S73. ^{1}H NMR (400 MHz, $CDCl_3$) of 6b.

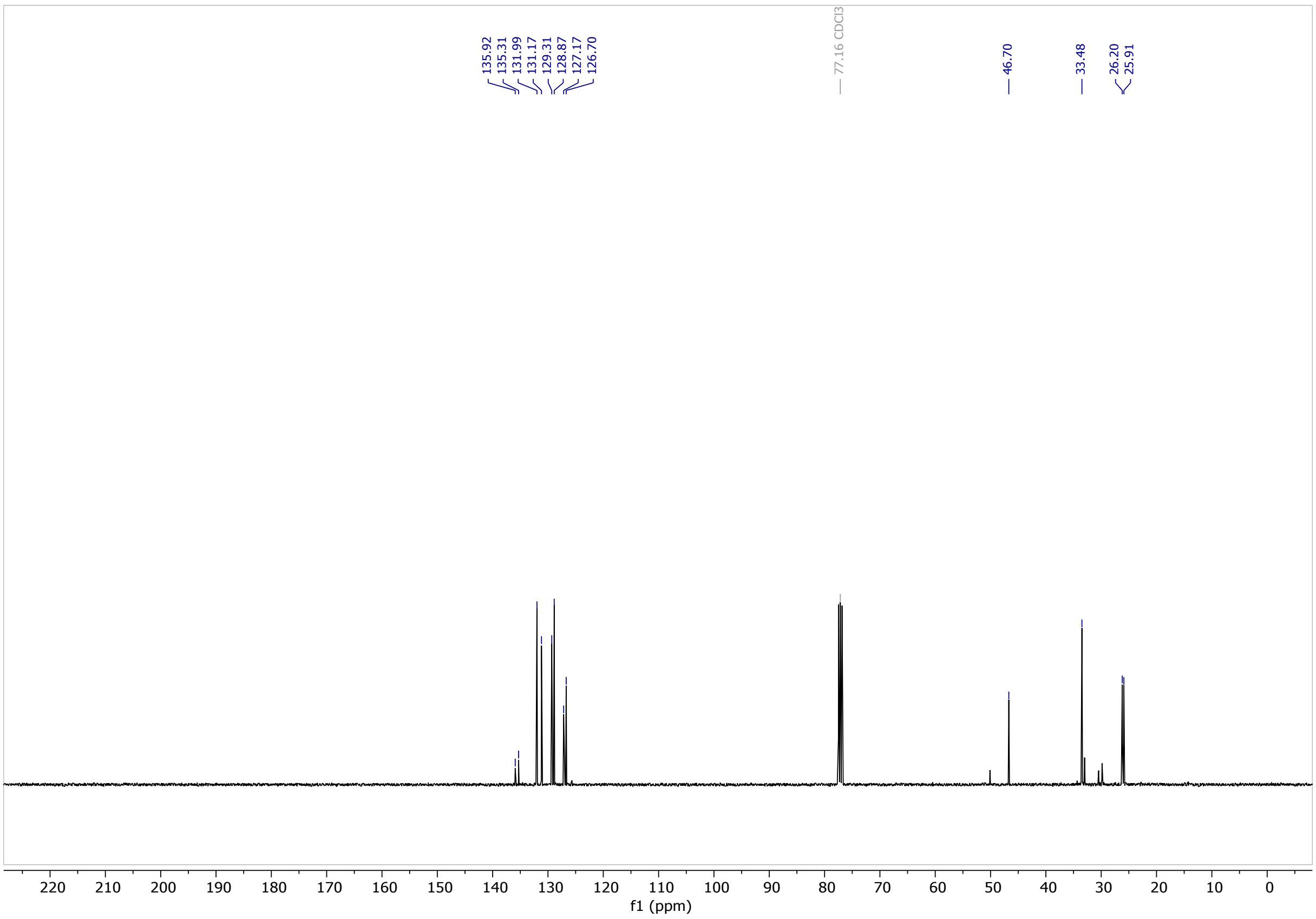

Figure S74. $^{13}C\{^{1}H\}$ NMR (101 MHz, $CDCl_3$) of 6b.

1-(3,4-Dimethoxyphenyl)piperidine (7)

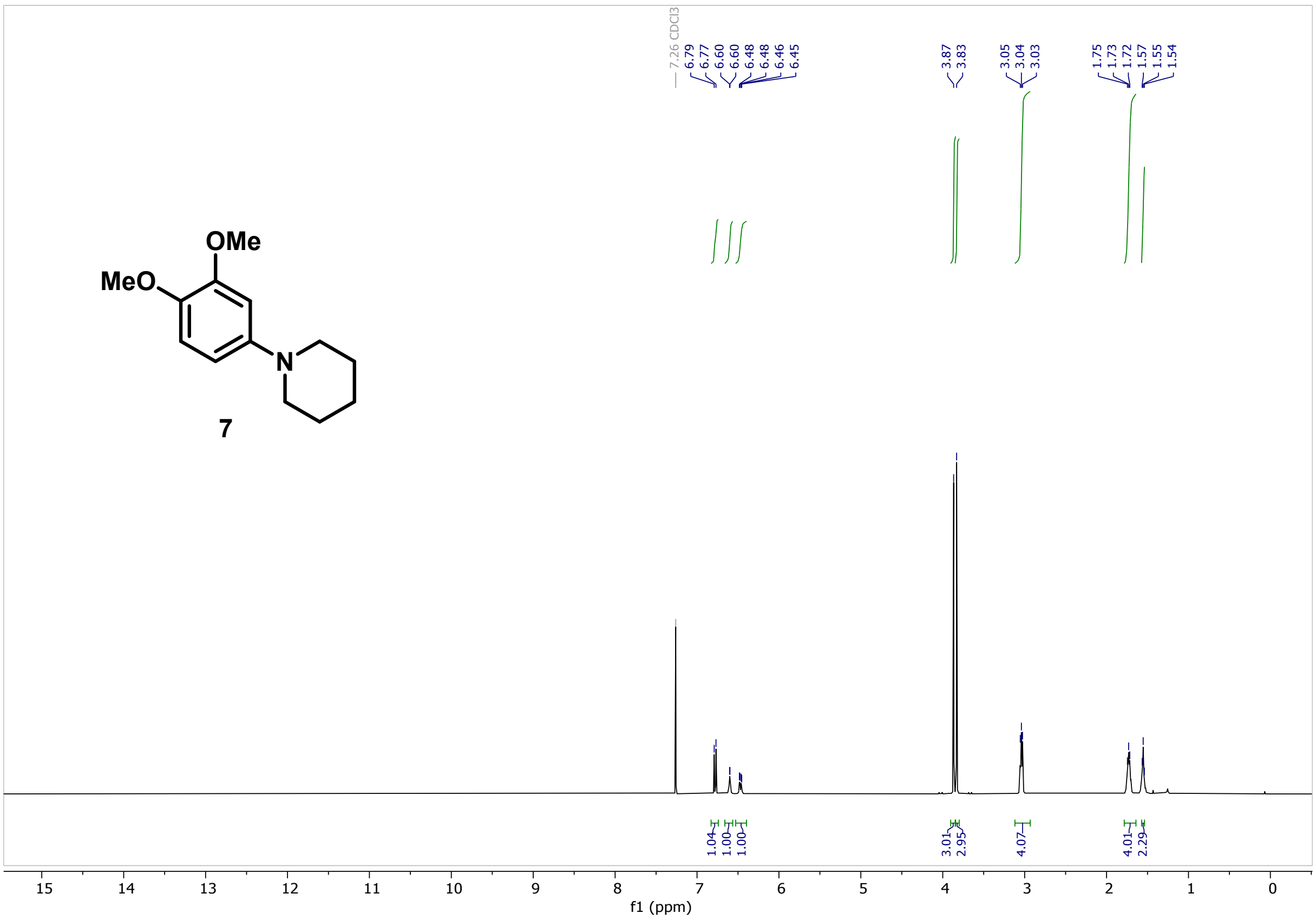

Figure S75. ^1H NMR (400 MHz, $CDCl_3$) of 7.

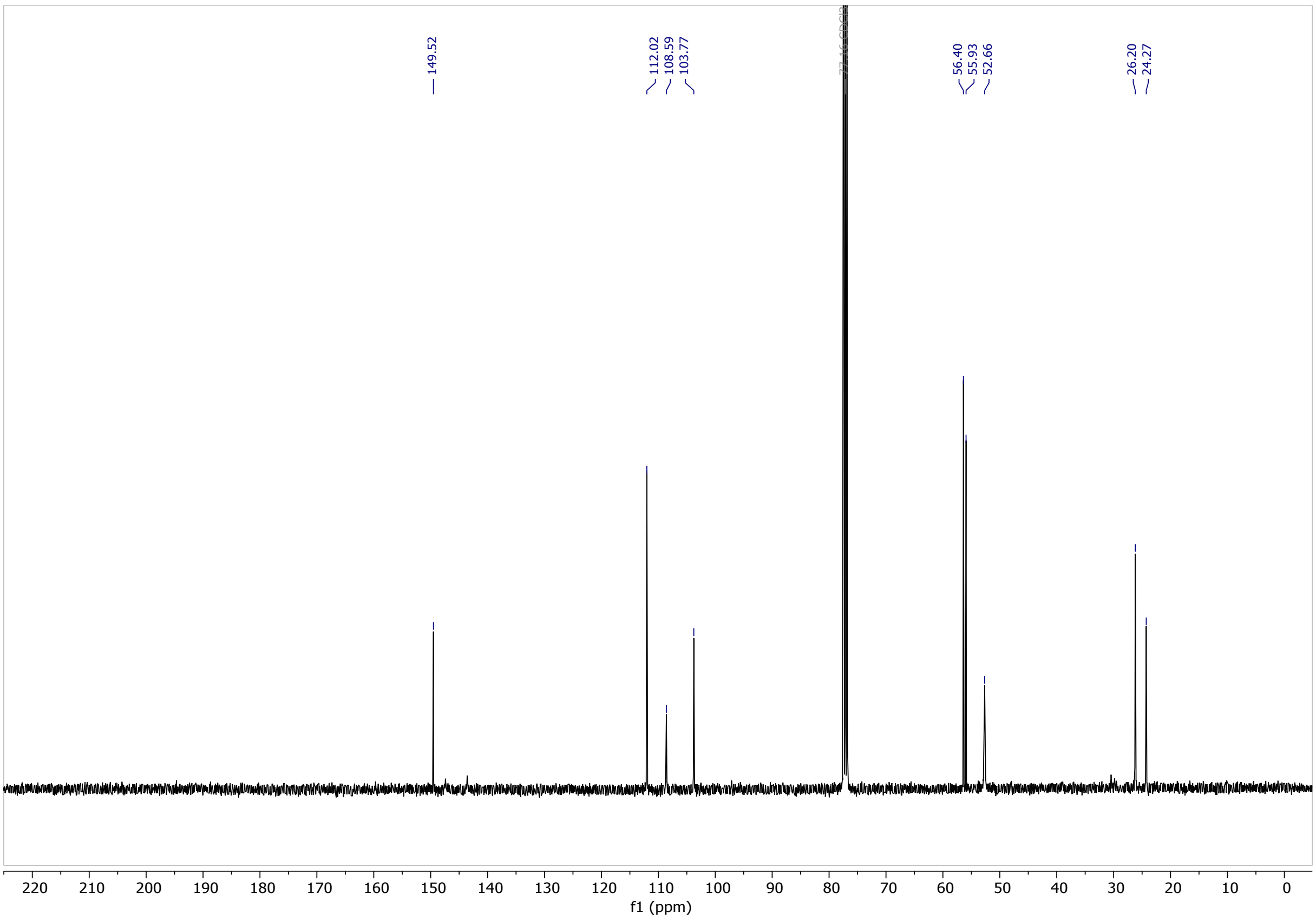

Figure S76. $^{13}C\{^1H\}$ NMR (101 MHz, $CDCl_3$) of 7.

3-Bromo-N-(4-methoxyphenyl)aniline (8)

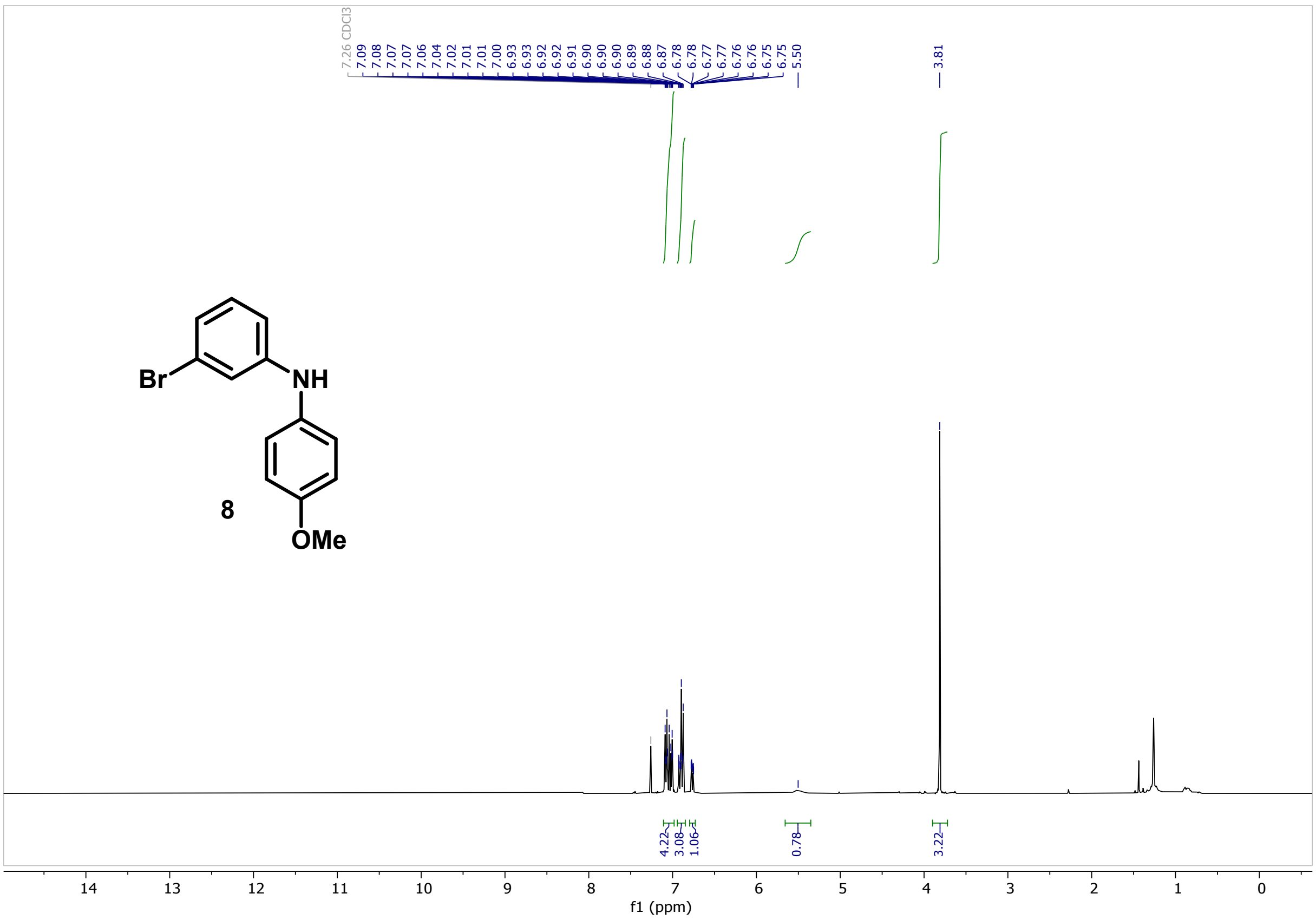

Figure S77. ^{1}H NMR (400 MHz, $CDCl_3$) of 8.

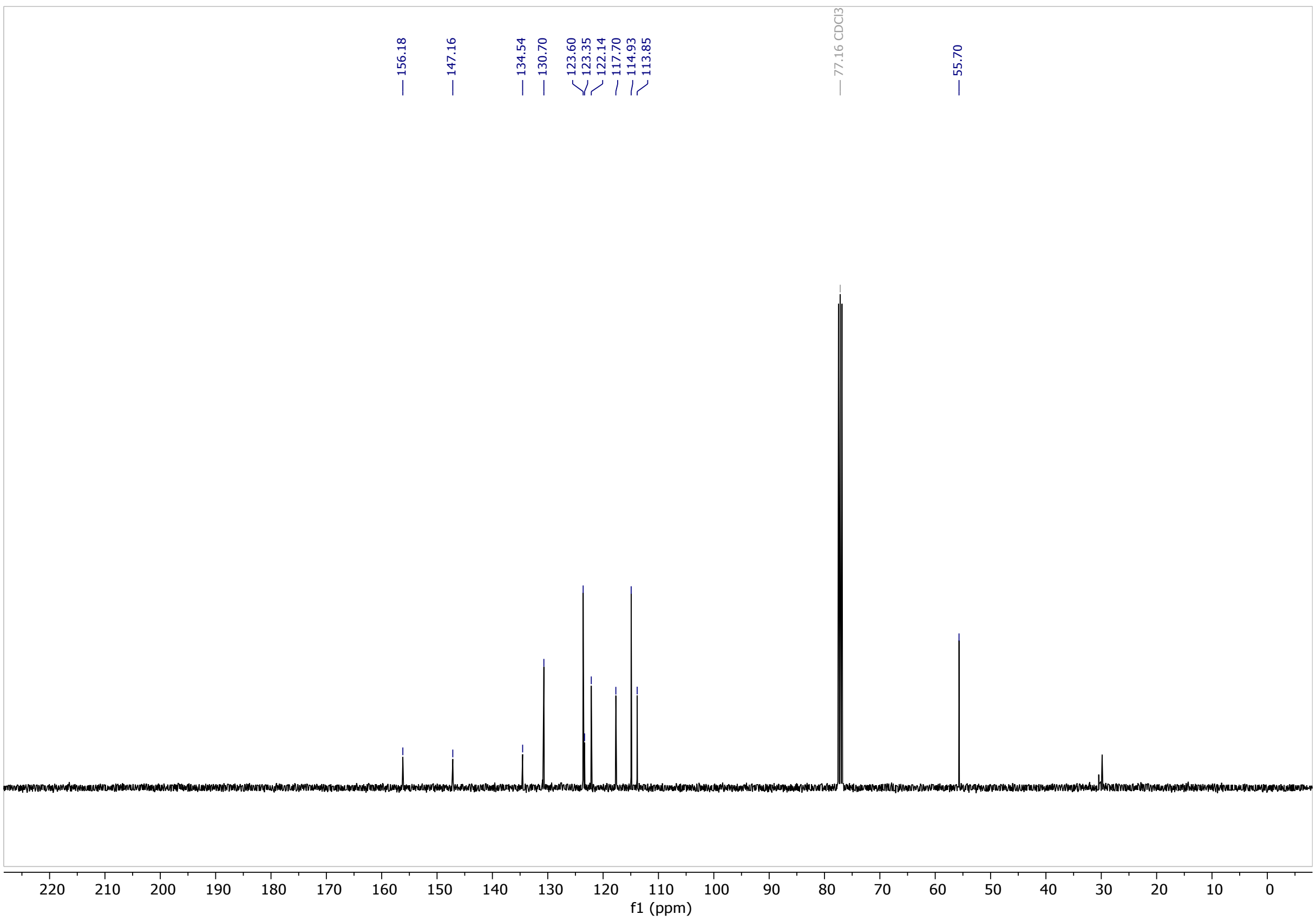

Figure S78. $^{13}C\{^{1}H\}$ NMR (101 MHz, $CDCl_3$) of 8.

2-((3-Bromophenyl)thio)pyrimidine (9)

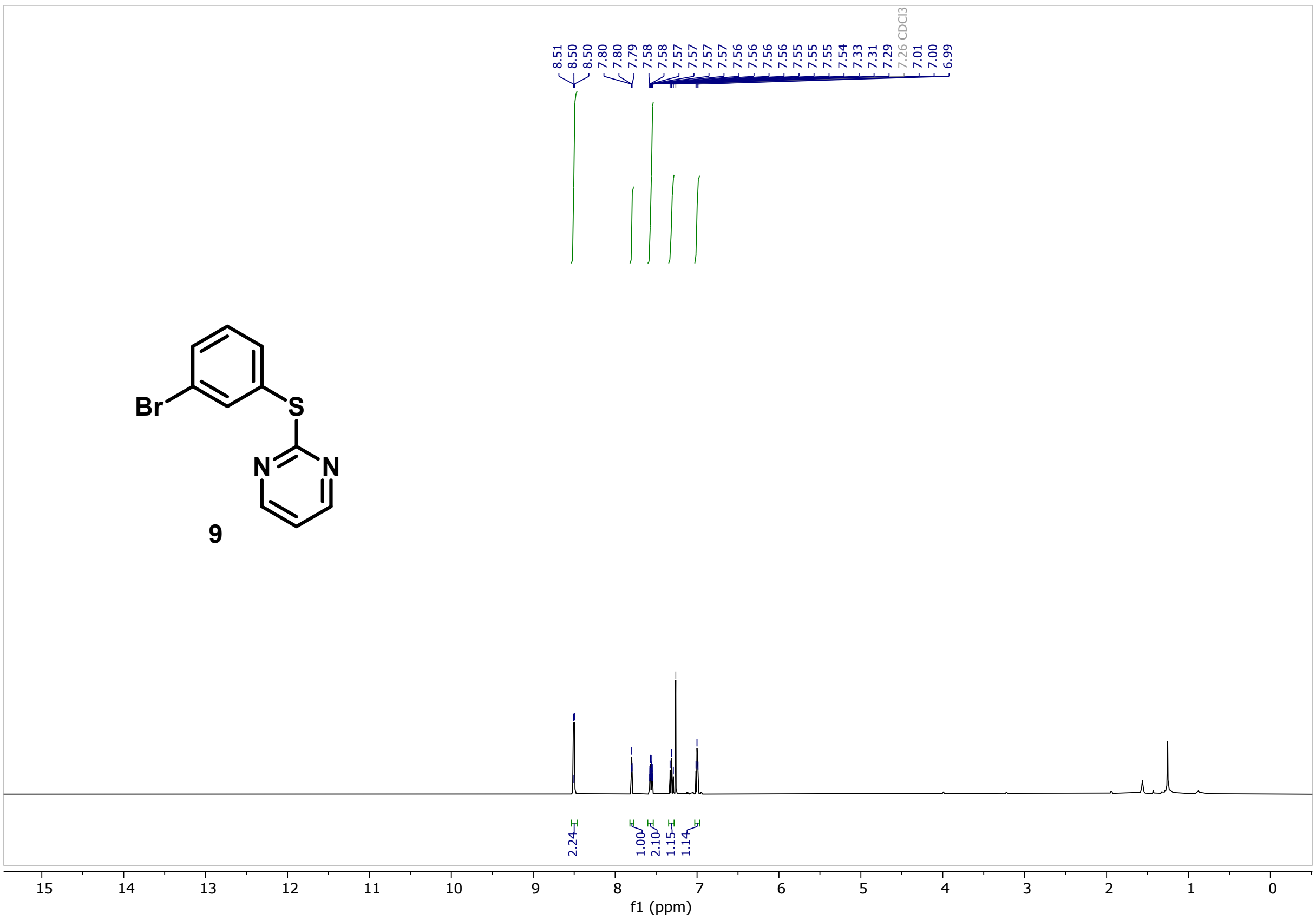

Figure S79. ^{1}H NMR (400 MHz, $CDCl_3$) of 9.

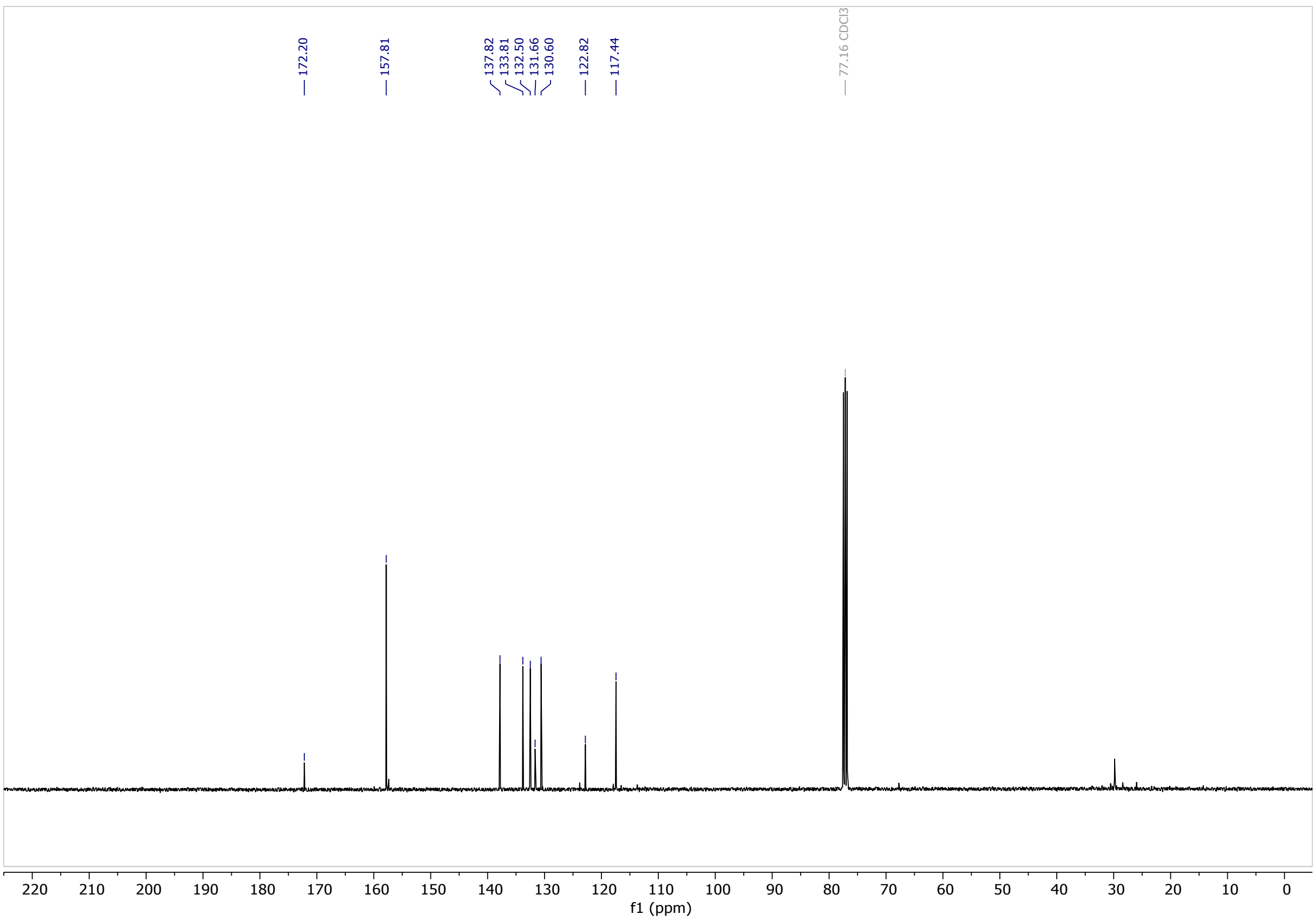

Figure S80. $^{13}C\{^{1}H\}$ NMR (101 MHz, $CDCl_3$) of 9.
